# Characterization of the TLR Family in *Branchiostoma lanceolatum* and Discovery of a Novel TLR22-Like Involved in dsRNA Recognition in Amphioxus

**DOI:** 10.3389/fimmu.2018.02525

**Published:** 2018-11-02

**Authors:** Jie Ji, David Ramos-Vicente, Enrique Navas-Pérez, Carlos Herrera-Úbeda, José Miguel Lizcano, Jordi Garcia-Fernàndez, Hector Escrivà, Àlex Bayés, Nerea Roher

**Affiliations:** ^1^Department of Cell Biology, Animal Physiology and Immunology, Institute of Biotechnology and Biomedicine (IBB), Universitat Autònoma de Barcelona, Bellaterra, Spain; ^2^Molecular Physiology of the Synapse Laboratory, Biomedical Research Institute Sant Pau (IIB Sant Pau), Barcelona, Spain; ^3^Department of Genetics, School of Biology and Institute of Biomedicine (IBUB), University of Barcelona, Barcelona, Spain; ^4^Department of Biochemistry and Molecular Biology, Institute of Neurosciences, Universitat Autònoma de Barcelona, Bellaterra, Spain; ^5^CNRS, Biologie Intégrative des Organismes Marins, BIOM, Sorbonne Université, Banyuls-sur-Mer, France

**Keywords:** toll-like receptor, TLR, evolution, amphioxus, Poly I:C, TLR22

## Abstract

Toll-like receptors (TLRs) are important for raising innate immune responses in both invertebrates and vertebrates. Amphioxus belongs to an ancient chordate lineage which shares key features with vertebrates. The genomic research on TLR genes in *Branchiostoma floridae* and *Branchiostoma belcheri* reveals the expansion of TLRs in amphioxus. However, the repertoire of TLRs in *Branchiostoma lanceolatum* has not been studied and the functionality of amphioxus TLRs has not been reported. We have identified from transcriptomic data 30 new putative TLRs in *B. lanceolatum* and all of them are transcribed in adult amphioxus. Phylogenetic analysis showed that the repertoire of TLRs consists of both non-vertebrate and vertebrate-like TLRs. It also indicated a lineage-specific expansion in orthologous clusters of the vertebrate TLR11 family. We did not detect any representatives of the vertebrate TLR1, TLR3, TLR4, TLR5 and TLR7 families. To gain insight into these TLRs, we studied in depth a particular TLR highly similar to a *B. belcheri* gene annotated as bbtTLR1. The phylogenetic analysis of this novel BlTLR showed that it clusters with the vertebrate TLR11 family and it might be more related to TLR13 subfamily according to similar domain architecture. Transient and stable expression in HEK293 cells showed that the BlTLR localizes on the plasma membrane, but it did not respond to the most common mammalian TLR ligands. However, when the ectodomain of BlTLR is fused to the TIR domain of human TLR2, the chimeric protein could indeed induce NF-κB transactivation in response to the viral ligand Poly I:C, also indicating that in amphioxus, specific accessory proteins are needed for downstream activation. Based on the phylogenetic, subcellular localization and functional analysis, we propose that the novel BlTLR might be classified as an antiviral receptor sharing at least partly the functions performed by vertebrate TLR22. TLR22 is thought to be viral teleost-specific TLR but here we demonstrate that teleosts and amphioxus TLR22-like probably shared a common ancestor. Additional functional studies with other lancelet TLR genes will enrich our understanding of the immune response in amphioxus and will provide a unique perspective on the evolution of the immune system.

## Introduction

There are two types of immunity in vertebrates. One is the innate immunity, which is genetically programmed to detect invariant features of invading microbes. The other is the adaptive immunity, which employs antigen receptors that are not encoded in the germ line but are generated *de novo* (1). The innate immune system is the first line of defense against infectious diseases (2). Immediately after infection, the innate response is activated to combat pathogens and synthesize inflammatory mediators and cytokines (3). However, the primary challenge of the innate immune system is how to discriminate a countless number of pathogens using a restricted number of receptors (2). As a response, a variety of receptors can recognize conserved motifs on pathogens (4). These conserved motifs are known as Pathogen-Associated Molecular Patterns (PAMPs) (5) and their recognition partners, are called Pattern Recognition Receptors (PRRs) (6).

Toll-like receptors (TLRs), among the most extensively studied PRRs, are type-I transmembrane proteins consisting of an ectodomain, a transmembrane (TM) domain and an intracellular Toll/interleukin-1 receptor (TIR) domain (7). The ectodomain, which functions as a PAMPs recognition domain, is arranged in tandem leucine-rich repeat (LRR), from one to many depending on the receptor type. The LRR contains a segment of 11 conserved residues with the consensus sequence LxxLxLxxNxL, where x can be any amino acid, L is a hydrophobic residue (leucine, valine, isoleucine, or phenylalanine) and N can be asparagine or cysteine (8). The TIR domain is present in the cytosol and is required for downstream signal transduction (9). Upon PAMP recognition, TLRs recruit TIR-domain containing adaptor proteins such as MyD88, TRIF, TIRAP/MAL, or TRAM, which initiate signal transduction pathways that culminate in the activation of NF-κB, IRFs, or MAP kinases regulating the expression of cytokines, chemokines, or type I interferons (IFN), which finally protect the host against infections (10).

TLRs are expressed in innate immune cells such as dendritic cells and macrophages as well as non-immune cells like fibroblast and epithelial cells (10). TLRs are largely divided into two subfamilies based on their subcellular localizations: cell surface or intracellular. Ten and twelve functional TLRs have been identified in humans and mice, respectively. Human TLR1, TLR2, TLR4, TLR5 and TLR6 are expressed on the cell surface and recognize mainly microbial membrane components such as lipids, lipoproteins and proteins. Human TLR3, TLR7, TLR8, TLR9 and murine TLR11, TLR12, TLR13, which are expressed in intracellular vesicles such as those in the endoplasmic reticulum (ER), endosomes, lysosomes and endolysosomes, and recognize nucleic acids (9, 11–13). Recently, the sequencing of the genome in five bony fish species has allowed the discovery of at least 16 TLR types in teleosts (14).

There are two structural types of TLRs according to the TLR ectodomain structure: sccTLRs and mccTLRs. The sccTLRs are characterized by the presence of a single cysteine cluster on the C-terminal end of LRRs (a CF motif), which is juxtaposed to the plasma membrane. Most TLRs found in deuterostomes have this domain organization. The mccTLRs are characterized by an ectodomain with two or more CF motifs and another cysteine cluster on the N-terminal side of the LRRs (NF motif). They are systematically found in protostomes but have also been identified in the invertebrate deuterostome *S. purpuratus* and the cnidarian *N. vectensis* (15). Both sccTLR and mccTLR share a common TLR structure: LRR+TM+TIR. According to the ectodomain architecture and phylogenetic criteria, vertebrate TLRs can be classified into six families: 1, 3, 4, 5, 7 and 11. TLR1 family includes TLR1/2/6/10/14/18/24/25 as well as TLR27; TLR3, 4 and 5 families only include TLR3, 4 and 5 itself; TLR7 family includes TLR7/8/9; TLR11 family includes two subfamilies: 11 (TLR11/12/16/19/20/26) and 13 (TLR13/21/22/23) (16, 17).

A variety of TLRs are capable of recognizing viruses. Among human TLRs, the envelope proteins from viruses are mainly recognized by TLR2 and TLR6. Viral nucleic acids are recognized by TLR3 (ssRNA or dsDNA), TLR7 (ssRNA), TLR8 (ssRNA), and TLR9 (dsDNA or CpG motifs) (18). In teleosts, it has been reported that Poly I:C could be recognized by different TLRs. Teleost TLR13 was firstly reported in Miiuy croaker (*Miichthys miiuy*) which showed cytoplasmic localization in HeLa cells. It could respond to both *Vibrio anguillarum* and Poly I:C injection *in vivo* and Poly I:C stimulation in leukocytes (19). In fugu (*Takifugu rubripes*), TLR3 localizes in the endoplasmic reticulum and recognizes relatively short dsRNA, whereas TLR22 recognizes long dsRNA on the cell surface (20). Grass carp (*Ctenopharyngodon idella*) TLR22 is expressed in many tissues and is highly abundant in the gills. Infection of grass carp with grass carp reovirus (GCRV), a dsRNA virus, induces a rapid up-regulation of TLR22 gene expression in the spleen (21). Japanese flounder (*Paralichthys olivaceus*) TLR22 is mainly expressed in peripheral blood leukocytes (PBL) and could be induced by both peptidoglycan and Poly I:C (22), whereas TLR3 gene expression in PBLs increased upon stimulation with Poly I:C and CpG ODN 1668 (23). Both TLR3 and TLR22 gene transcription had also been studied in large yellow croaker. Basal gene transcription was high in several immune organs and could be up-regulated after injection of Poly I:C in the anterior kidney (TLR22), spleen (TLR3 and 22), liver (TLR3) and blood (TLR3) (23). In the common carp (*Cyprinus carpio* L.), TLR22 was transcripted in almost all the tissues. When fish was challenged with Poly I:C or *Aeromonas hydrophila*, the transcription of this TLR was up-regulated in a variety of tissues (24). Overall, TLRs with immune function have been found from cnidarians to mammals which imply a conserved evolution. TLR3 is found both in mammals and teleost whereas TLR22 is present in many fish species and *Xenopus*, but absent from birds and other terrestrial animals (25). The origin of the TLRs involved in dsRNA virus recognition is still under study. The current hypothesis is that specific fish TLR duplication results from the fish specific Whole Genome Duplication (WGD) (26–28), but here we show that, in amphioxus, exists an ortholog of the TLR11 subfamily possessing TLR22 functional similarities, pointing out that a TLR22-like function was present in the ancestor of chordates.

Amphioxus belongs to an ancient chordate lineage which shares key anatomical and developmental features with vertebrates and tunicates (also known as urochordates) (29). All chordates have a similarly organized genome though amphioxus has relatively little duplication (30). Thus amphioxus, with its phylogenetic position diverging at the base of chordates and its genomic simplicity, is a good non-vertebrate model to understand the evolution of vertebrates (31). *Branchiostoma lanceolatum* (Mediterranean amphioxus) has been extensively studied together with other amphioxus species such as *Branchiostoma belcheri* (Asian amphioxus), *Branchiostoma japonicum* (Asian amphioxus) and *Branchiostoma floridae* (Florida amphioxus) (32). To date, genomic data have revealed that *B. floridae* has 48 TLRs (33). However, only one full-length TLR, annotated as bbtTLR1, was functionally characterized in *B. belcheri tsingtauense* until now. The experimental data supports the immunological function of this TLR that together with MyD88 is involved in the activation of NF-κB signaling pathway (34). Further studies of TLRs in amphioxus are required to better understand the ancestors and functional evolution of vertebrate TLRs.

In this study, we investigated the total number of TLR genes in *B. lanceolatum* and studied their phylogenetic and evolutionary relationships with vertebrate and invertebrate TLRs. We also examined the total number of TLR genes in *B. floridae* and *B. belcheri* according to our definition of a true TLR. We studied the basal gene expression of all the TLRs in adult amphioxus (*B. lanceolatum*). Moreover, we cloned the full length of a novel TLR in *B. lanceolatum* and we further investigated its subcellular localization and PAMP binding specificity using NF-κB luciferase assay in a mammalian expression system. Exhaustive phylogenetic analysis combined with functional data has allowed us to explore the evolution and function of this novel TLR compared with vertebrate TLRs.

## Materials and methods

### Sequence analysis: phylogeny and bioinformatics

To characterize the TLR repertoire of *B. lanceolatum*, we performed a search using the BbtTLR1 sequence (GenBank: DQ400125.2) and an unpublished transcriptome of *B. lanceolatum* derived from several adult tissues and embryonic stages. The transcriptome data were obtained from an exhaustive collection of 52 RNA-Seq datasets using the Illumina technology. From 15 embryonic stages, one pre-metamorphosis stage and 9 adult organs, a total of 4.2 billion Illumina reads with a volume of 871 Gbp were obtained. These embryonic stages are eggs, 32 cells, blastula, 7, 8, 10, 11, 15, 18, 21, 24, 27, 36, 50, and 60 hpf. The adult tissues are neural tube, gut, hepatic tissue, gills, epidermis, muscle, female and male gonads, and cirri. For the transcriptome assembly, Tophat2 was used mapping each strand-specific RNA-seq sample against the recently assembled *B. lanceolatum* genome. Gene models were built using Cufflinks and each annotation merged using Cuffmerge to produce a single collection of transcripts. The transcriptome was translated into predicted proteins using the TransDecoder suite v3.0.1. From the PFAM database v30.0, we downloaded the hidden Markov models profile collection (Pfam-A.hmm.gz) and extracted the two profiles for the protein domains that we were looking for, the TIR and the LRR domains. HMMER 3.1b was then used with the hmmsearch mode to identify the predicted proteins with these domains. Finally, a manually curated annotation was performed. Specific primers for each *B. lanceolatum* TLR were designed using NCBI primer designing tool (https://www.ncbi.nlm.nih.gov/tools/primer-blast/) and are shown in Supplementary Table 1.

To study the phylogenetic relationship of *B. lanceolatum* and vertebrate TLRs, we performed the maximum-likelihood analysis. *Drosophila melanogaster* Toll sequences and vertebrate TLR protein sequences were obtained from the National Center for Biotechnology Information (NCBI, https://www.ncbi.nlm.nih.gov/) and UniProt (http://www.uniprot.org/) (Supplementary Table 2). TLR sequences of *Lytechinus variegatus* (35) and *Saccoglossus kowalevskii* (36) were obtained from online repositories and a search similar to the one carried on in *B. lanceolatum* (Supplementary Data 1). In all the phylogenetic analysis, we only included the sequences that have a complete TIR domain. For full-length protein, sequences were aligned with MAFFT (37) choosing L-INS-i method which optimizes alignments for sequences containing hypervariable regions flanked by one alignable domain. For TIR domain, sequences were aligned with MAFFT choosing G-INS-i method which allows to align the entire region with a global conservation. The alignment was trimmed using TrimAL (38) with “Automated 1” mode. The phylogenetic reconstruction was done using IQ-TREE (39) and its built-in ModelFinder software (40). Branch support was calculated running 1,000 replicates of the SH-like approximate likelihood ratio test (SH-aLRT) (41) and ultrafast bootstrap (42).

The TLR sequences of *B. floridae* and *B. belcheri* were obtained from the databases of JGI (http://genome.jgi.doe.gov/Brafl1/Brafl1.home.html) and LanceletDB (http://genome.bucm.edu.cn/lancelet/index.php), respectively. The open reading frame was identified through sequence translation with ExPASy software (http://web.expasy.org/translate/). Transmembrane regions were predicted using TMHMM server v2.0 (http://www.cbs.dtu.dk/services/TMHMM/). The number of LRR domains was predicted using LRRfinder software (http://www.lrrfinder.com/lrrfinder.php). Full-length protein domain was predicted by the Simple Modular Architecture Research Tool (SMART) (http://smart.embl-heidelberg.de/). The single cysteine cluster TLRs (sccTLRs) and multiple cysteine cluster TLRs (mccTLRs) were characterized according to Leulier and Lemaitre (15). The first annotated sequence was selected according to the blastp software in NCBI. The molecular weight of BlTLR was calculated with ProtParam (http://web.expasy.org/protparam/). The sequence of BlTLR was examined for the presence of a signal peptide using SignalP (http://www.cbs.dtu.dk/services/SignalP/). N-linked glycosylation site was predicted with NetNGly 1.0 server (http://www.cbs.dtu.dk/services/NetNGlyc/). Multiple sequence alignment of BlTLR and fish TLR22 was performed by Clustal Omega (https://www.ebi.ac.uk/Tools/msa/clustalo/).

The phylogenetic analysis of three *Branchiostoma* species (*B. floridae, B. belcheri* and *B. lanceolatum*) TLRs was performed using TIR domain sequences. The TIR domain sequences of vertebrates, *S. kowalevskii* and *D. melanogaster* were included. All the TIR domain sequences were identified from the full-length protein using SMART software. Prior to the analysis, sequences were aligned with MAFFT choosing G-INS-i method. The alignment was trimmed using TrimAL with “Automated 1” mode. The phylogenetic analysis was done using IQ-TREE and its built-in ModelFinder software. Branch support was calculated running 1,000 replicates of the SH-like approximate likelihood ratio test and ultrafast bootstrap.

The phylogenetic analysis of BlTLR and BbtTLR1 was performed with the full-length protein using IQ-TREE software. The *D. melanogaster* Toll and the vertebrate TLR sequences were included in the analysis. The sequences were aligned with MAFFT choosing L-INS-i method. The alignment was trimmed using TrimAL with “Automated 1” mode. In the analysis, branch support was calculated running 1,000 replicates of the SH-like approximate likelihood ratio test and ultrafast bootstrap.

### Animals

*Branchiostoma lanceolatum* adults were collected in the bay of Argelès-sur-Mer, France (latitude 42° 32′ 53″ N and longitude 3° 03′ 27″ E) with a specific permission delivered by the Prefect of Region Provence Alpes Côte d'Azur. *B. lanceolatum* is not a protected species. Amphioxus were kept in the laboratory in 60-l glass tanks with ~50-l seawater and 5 cm height of sand on the bottom. Water temperature was maintained around 17°C and the salinity ranged between 40 and 45 PSU. The photoperiod was set to 14 h light/10 h dark. The animals were not fed with extra food during the experiment.

### RNA isolation, cDNA synthesis and RT-PCR

Total RNA was extracted from the whole animal using TRI reagent (Sigma-Aldrich) according to the manufacturer's protocol. The homogenization was performed with a Polytron homogenizer (Kinemetica). The quality of the RNA was assessed with a Bioanalyzer (Agilent Technologies) and the concentration was measured with a Nanodrop (Thermo scientific). The RNA was purified using an RNeasy micro kit (Qiagen) and DNAse treated according to manufacturer's instructions and stored at −80°C. The first-strand cDNA was synthesized with SuperScript III first-strand synthesis system (Thermo Fisher Scientific). RT-PCR reactions were performed with primers specific for each TLR under following conditions: initial denaturation at 94°C for 5 min, followed by 35 cycles of denaturation at 94°C for 45 s, annealing at 60°C for 45 s, and extension at 72°C for 50 s, and a final extension at 72°C, 7 min. Glyceraldehyde-3-phosphate dehydrogenase (GAPDH) was used as a reference gene. PCR products were separated in 1% agarose gel electrophoresis and stained with GelGreen Nucleic Acid Gel Stain (Biotium). Agarose gel imaging was performed with a GelDoc XR system (Bio-Rad). Six of the PCR products were purified and sequenced.

### Full-length cDNA cloning of BLTLR

A DNA BLAST search of NCBI database was conducted using BbtTLR1 sequence from *B. belcheri* (GenBank: DQ400125.2). We obtained a sequence (GenBank: AF391294.1) from *B. floridae* showing 82% identity. In addition, a DNA BLAST search using bbtTLR1 was performed in the genome scaffold of *B. lanceolatum* and we identified a short sequence (ContigAmph29716) showing 83% identity. The forward primer (Table 1) was designed based on the conserved region between bbtTLR1 *B. belcheri* and *B. floridae* sequence. The reverse primer (Table 1) was designed based on the ContigAmph29716 sequence. We cloned a fragment of around 2,000 bp by PCR using the cDNA prepared from the whole animal. The 5′-end was obtained by 5′ RACE (Invitrogen) using gene specific primers (Table 1). A fragment of ~600 bp was obtained. The 3′-end was obtained by 3′ RACE (Invitrogen) using gene specific primer (Table 1). A fragment of ~1,000 bp was obtained. Finally, a PCR amplification was carried out to obtain the full-length sequence with Expand high fidelity PCR system (Roche) using the full-length primers (Table 1) designed in the non-coding regions from both 5′ to 3′-ends. All the fragments were separated by electrophoresis and cloned into the pGEM-T Easy Vector (Promega). Sequencing was carried out using T7 and SP6 primers (Servei de Genòmica i Bioinformàtica, IBB-UAB).

**Table 1:** Primers used for cloning and RT-qPCR.

**Category**	**Primer**	**Sequence (5′-3′)**	**Product size (bp)**
Fragment	Forward	GGGACGATCCAGTCACGCTG	2,190
	Reverse	GACACCAACGGCTGCGCAG	
5′RACE	Reverse1	GAGTGAAGAACAGTGA	684
	Reverse2	GTCATTCCCTCCAAGGTTCAAAGAAGTC	
3′RACE	Forward	CGAAGACAGGCGATGGGTT	1,119
Full-length	Forward	AGAGAGAGAAAACTGCCAGCC	3,077
	Reverse	TTTCTGTCTCGACGGTCCTT	
RT-qPCR	Forward	TCACACGCTTTCTACGGCTT	122
	Reverse	AGGCTTAGGTCCAGTACGGT	
GAPDH	Forward	CCCCACTGGCCAAGGTCATCA	154
	Reverse	GCTGGGATGATATTCTGGTGGGC	

### LPS and poly I:C treatment *in vivo*

Adult amphioxi were treated with either 10 μg/ml bacterial lipopolysaccharide (LPS) from *Escherichia coli* O111:B4 strain (Sigma-Aldrich) or 10 μg/ml Poly I:C, a synthetic analog of dsRNA viruses (Invivogen) by bath immersion. The stocks of LPS and Poly I:C solution were prepared in PBS (Sigma-Aldrich) and diluted to the indicated working concentrations with sterile seawater. Seawater sterilization was performed with 0.22 μm sterile filter. PBS prepared in seawater (1% v/v) was used as a control. Three, 6, 12 and 24 h after immersion, 3 animals from each group were sampled separately. The animals were frozen in liquid nitrogen immediately and stored in −80°Cuntil use. Total RNA was prepared from the whole animal and the first-strand cDNA was synthesized for RT-qPCR analysis.

### RT-qPCR analysis

RT-qPCR was carried out to analyze the relative transcription level of BlTLR after LPS and Poly I:C treatments. The analysis was performed in the CFX384 Touch Real-Time PCR Detection System (Bio-Rad) using the iTaq universal SYBR green supermix kit (Bio-Rad) following the manufacturer's protocol. The RT-qPCR primers (Table 1) were designed to detect the transcription level of BlTLR. GAPDH gene was used as a reference gene. 10^−1^ and 10^−2^-fold cDNA dilutions were used for BlTLR and GAPDH gene expression analysis, respectively. Each PCR mixture consisted of 5 μl of SYBR green supermix, 0.5 μM of primers, 2.5 μl of diluted cDNA, and 1.5 μl sigma water in a final volume of 10 μl. All samples were run in triplicate using the following steps: initial denaturation at 95°C for 3 min, 39 cycles of 95°C for 10 s and 60°C for 30 s, and finally, 95°C for 10 s, increase every 0.5°C for 5 s from 65 to 95°C. The relative transcription levels were calculated using the 2^−ΔΔ*CT*^ method (43). All the data were analyzed using GraphPad software and significant differences were analyzed by one-way analysis of variance (ANOVA) using the value of ΔCt (normalize each technical repeat's gene-specific Ct value by subtracting from it the reference gene Ct value) (44).

### Plasmids

To study the subcellular localization of BlTLR in HEK293 cell, the coding sequence was cloned into pIRES2-EGFP vector (BD Biosciences Clontech, 6029-1) with two HA-tags (YPYDVPDYA) at 3′ end (named BlTLRHA) using XhoI and EcoRI as restriction sites. For testing the specific ligand binding of BlTLR, the ectodomain and transmembrane domain (amino acids 1-774) of BlTLR fused with human TLR2 cytoplasmic region (amino acids 611-784; NCBI: NP_001305716.1) was cloned into pIRES2-EGFP vector (named chimeric BlTLR) between SacII and EcoRI restriction sites. The eukaryotic expression vector pIRES2-EGFP was purchased from BD Biosciences. The NF-κB-dependent luciferase reporter vector (pNFκB) and the Renilla luciferase vector (pRenilla) were provided by Dr. José Miguel Lizcano. All the plasmids were confirmed by sequencing and agarose gel electrophoresis digested with the corresponding restriction enzymes. All the plasmids were purified at large scale using NucleoBond Maxi endotoxin-free plasmid isolation kit (Fisher Scientific) and stored at −20°C until use.

### Cell culture, transient transfection and stable cell lines

HEK293 cells were grown in complete medium: DMEM (Life Technologies, 31885) supplemented with 10% (v/v) FBS (Gibco) and 1% (v/v) penicillin and streptomycin (Gibco) at 37°C and 5% CO_2_. Plasmids were transiently transfected in HEK293 cells using linear polyethylenimine (PEI, CliniScience) at a ratio of 3:1 (μg PEI: μg plasmid). HEK293 cell lines stably expressing BlTLRHA and chimeric BlTLR were generated by Geneticin selection (Invitrogen, G418). In brief, 24 h after transient transfection, the culture medium was substituted with selective medium containing 1 mg/ml G418. Selective medium was refreshed every 2–3 days until the G418-resistant foci could be identified and all non-transfected cells (control) were dead (around 2 weeks). The colonies were picked and expanded in selective culture medium containing 1 mg/ml G418 for the following 2 weeks. Then, HEK293 stable cell lines were isolated via GFP-positive cell sorting (FACSJazz) in order to enrich the stable cell line. Finally, the HEK293 stable cells lines were cultured in DMEM complete medium at 37°C and 5% CO_2_.

### Flow cytometry

To assess the transient transfection efficiency of plasmid BlTLRHA in HEK293 cells, flow cytometry was performed using a FACS Canto (Becton Dickinson, USA). In brief, HEK293 cells were seeded on 6-wells plate (Thermo Scientific) at 50% density. The cells were transfected with empty vector (pIRES2-EGFP) and BlTLRHA plasmid using PEI as described above. Non-transfected cells were used as negative control. Cells were detached using TrypLE (Gibco) and re-suspended in PBS for cytometry analysis at 24, 48 and 72 h after transfection. The cytometer was set to detect the GFP signal and a total 10,000 events were recorded. The raw data were analyzed with Flowing software (Finland) and GraphPad software. Flow cytometry was also used to assess the percentage of transfected cells when setting up the stable cells lines BlTLRHA and chimeric BlTLR.

### Western blot analysis

HEK293 cells were transiently transfected with empty vector (pIRES2-EGFP) and BlTLRHA plasmid as described above. Cells were lysed in 200 μl cell lysis buffer (250 mM sacarose, 150 mM Tris, 5 mM EDTA, 125 mM DTT, 5% SDS, 2.5% bromophenol blue and 7.5% β-mercaptoethanol in water) and detached on ice using a cell scraper (BD Falcon) at 24, 48 and 72 h after transfection. The lysed cells were subjected to sonication for 10 s and centrifugation. After heating at 100°C for 5 min, the cell extracts were loaded into 10% SDS-PAGE and then transferred to PVDF membranes (EMD Millipore) using a Mini-protean Tetra (Bio-Rad). After 1 h blocking in 5% (w/v) BSA (Sigma-Aldrich) in TBST (50 mM Tris, 150 mM NaCl and 0.1% Tween 20), membranes were incubated with 1 μg/μl mouse anti-HA primary antibody (Covance, MMS-101P) overnight at 4°C, followed by incubation with a secondary HRP-conjugated antibody for 1 h at room temperature (RT). Proteins were visualized with a GelDoc system (Bio-Rad) by adding the SuperSignal West Pico chemiluminescent substrate (Thermo Fisher Scientific).

### Immunofluorescence and confocal microscopy

HEK293 cells were seeded (50% density) on 24 × 24 mm cover glasses (Labbox) coated with Poly-D-lysine hydrobromide (Sigma-Aldrich). The BlTLRHA plasmid was transiently transfected as described above. Cells were washed 3 times with DMEM at 48 h after transfection. For non-permeabilization, cells were blocked with 2% BSA in DMEM for 10 min at 37°C, and then incubated with mouse anti-HA primary antibody (1/500 diluted in DMEM) for 1 h at 37°C. Cells were washed 3 times with DMEM and fixed with 4% paraformaldehyde (PFA, Sigma-Aldrich) for 15 min at RT. After PBS washing, for transient transfection, fixed cells were incubated with anti-mouse Alexa Fluor 555 secondary antibody (Invitrogen) at 1:1,000 dilution for 2 h at RT; for stable transfection, cells were incubated with 5 μg/ml wheat germ agglutinin (WGA) conjugated with Alexa Fluor 647 for 10 min at RT before applying the secondary antibody at 1:1,000 dilution for 2 h at RT. For permeabilization, cells were washed with DMEM for 3 times and fixed with 4% PFA for 15 min at RT. After 3 washes with PBS, for transient transfection, cells were permeabilized with 0.2% Triton X-100 (Sigma-Aldrich) for 15 min at RT; for stable transfection, cells were incubated with 5 μg/ml WGA for 10 min at RT and then permeabilized with 0.1% Tween (Sigma-Aldrich) for 10 min at RT or the freeze and thaw method according to Mardones and González (45). After that, cells were blocked with 2% BSA in PBS for 1 h at RT, incubated with mouse anti-HA primary antibody (1/1,000 dilution) overnight at 4°C, followed by incubation with secondary anti-mouse AlexaFluor 555 antibody (Invitrogen) at 1:1,000 dilution for 2 h at RT. For both methods, cover glasses with cells were placed on SuperFrost Plus slides (Thermo scientific) covered with Fluoroshield with DAPI mounting medium (Sigma-Aldrich). Confocal imaging was performed using a Leica SP5 confocal microscope with a 63 × oil objective. The images were analyzed with Fiji software (46).

### Ligand stimulation and NF-κB luciferase reporter assay

Human TLR1-9 agonist kit (tlrl-kit1hw) and murine TLR13 agonist (tlrl-orn19) were purchased from Invivogen. HEK293 stable cell lines were used to minimize the deviation among different experiments. The stable cell lines were transfected with 0.5 μg/ml pNFκB and 0.05 μg/ml pRenilla (0.5 ml per well) using PEI. Renilla was used as internal control to normalize the differences in the reporter due to different transfection efficiencies. Twenty-four hours after transfection, cells were treated with indicated concentrations of ligands (Supplementary Table 3) for 16 h. As a positive control, 20 ng/ml human TNFα (Sigma-Aldrich) was used. The experiment was performed in triplicate. Luciferase activity assay was performed with the Dual-luciferase reporter assay system (Promega) using the Victor3 (PerkinElmer) according to the manufacturer's instructions. Briefly, after removing the growth medium from the well, cells were washed with PBS (2X). One hundred μl of passive lysis buffer (PLB) were added to each well. Then, the NF-κB-dependent firefly luciferase reporter was measured by adding 100 μl of luciferase assay reagent II (LAR II). After quantifying the firefly luminescence, the reaction was quenched. The Renilla luciferase reaction was initiated by adding 100 μl Stop & Glo Reagent to the same well and the Renilla luminescent signal was detected. The luciferase activity was expressed as the ratio of NF-κB-dependent firefly luciferase activity divided by Renilla luciferase activity.

## Results

### The TLR family in *B. lanceolatum*

**Figure 1:**
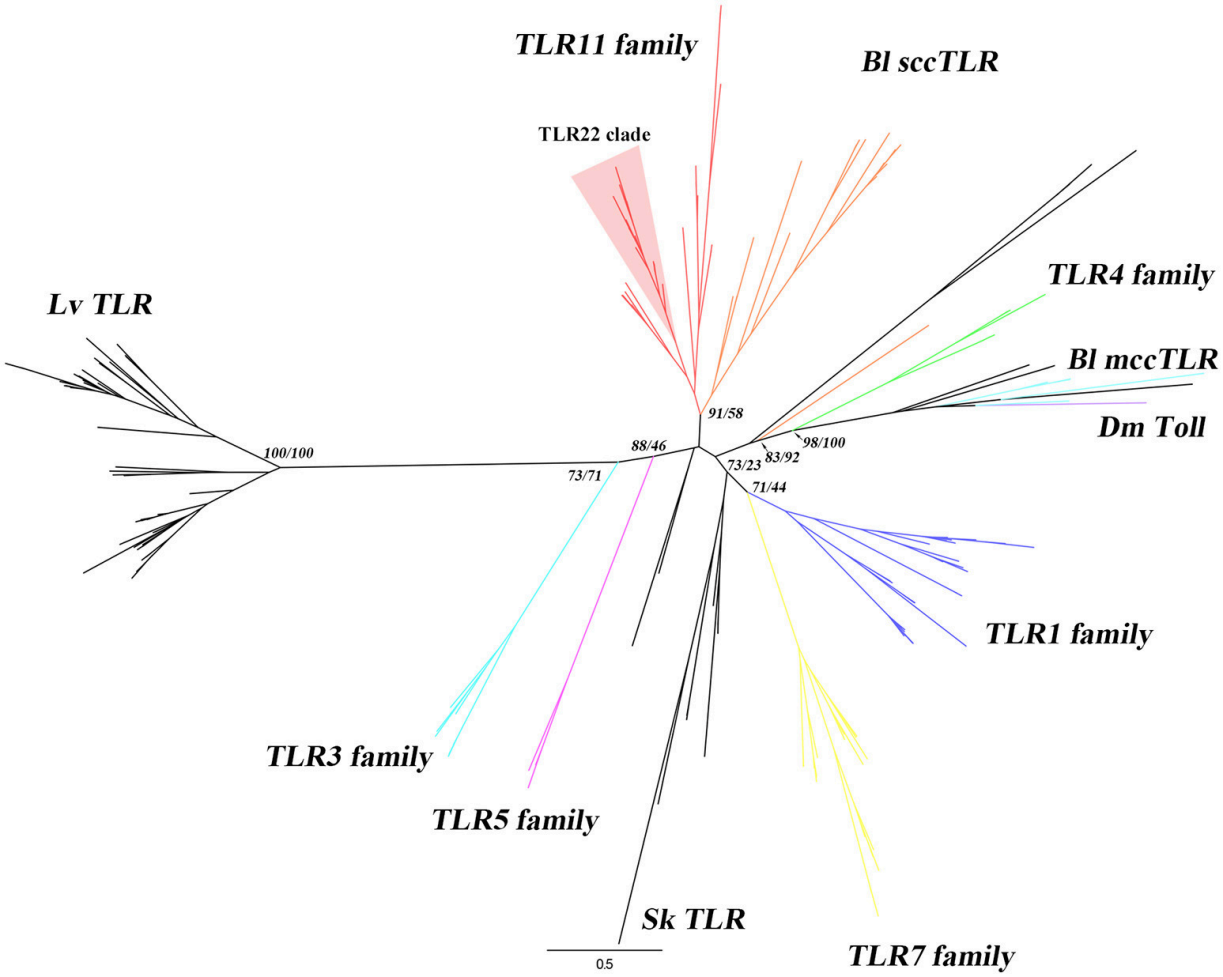
Phylogenetic analysis of *B. lanceolatum* TLRs. The phylogenetic tree was constructed using maximum-likelihood (IQ-TREE) with the full-length protein sequences. TLR sequences of *B. lanceolatum, S. kowalevskii, L. variegatus*, representative vertebrates and *D. melanogaster* Toll were used. Three TLR sequences (Bl10262, Bl22164 and Bl08928c) with incomplete TIR domain were removed from the analysis. Sequences were aligned with MAFFT choosing L-INS-i method and the alignments were trimmed using TrimAL with “Automated 1” mode. The best evolutionary model was established by ModelFinder according to BIC. The branch labels (numbers) are SH-aLRT support (%)/ultrafast bootstrap support (%) at the tree nodes. The tree was generated in FigTree. Dm Toll, Bl mccTLRs, Bl sccTLRs, Sk TLR, Lv TLR and 6 vertebrate TLR families (highlighted in different colors) are shown. TLR22 clade is shown with a red background. The detailed tree with all node supports can be found in Supplementary Figure 2.

A search for TIR and LRR domains was performed and proteins with both domains were selected as candidates. Then, these candidates were manually curated and a list of putative TLRs was obtained (Supplementary Data 2). Despite there are TLR-related molecules lacking extracellular LRR domains reported in some species of *Hydra* and coral (15), we only considered those sequences with at least one LRR domain, one TM domain and one TIR domain to obtain our final list of true TLR candidates. Using this rule, we obtained 30 TLRs. In order to understand the evolution of TLR of *B. lanceolatum*, we performed a phylogenetic analysis with representative vertebrate and invertebrate TLR sequences. Other authors had used either the full-length protein or the TIR domain to study the TLR evolution (32, 46–48). Therefore, we used full-length protein to perform the phylogenetic analysis when the sequences were complete, or TIR domain when there were incomplete or truncated sequences. The phylogenetic analysis of *B. floridae, B. belcheri* and *B. lanceolatum* using TIR domain sequences showed that there are two major clusters of TLRs (mccTLRs and sccTLRs) in *Branchiostoma*. However, we obtained a single clade with almost all the *Branchiostoma* sequences, clustered with vertebrate TLR3, 5 and 7 families (Supplementary Figure 1). This approach did not allow the identification of inter-taxa relationships between vertebrate and *Branchiostoma* TLR families. Roach et al. predicted that a strong selective pressure for specific PAMPs recognition maintains a largely unchanged repertoire of TLR recognition in vertebrates (16). Thus, we did phylogenetic analysis using the highly refined full-length TLR sequences of *B. lanceolatum* to better understand the evolutionary relationships with vertebrate TLRs. The phylogenetic analysis showed that the vertebrate TLRs were grouped into six clusters (TLR1, TLR3, TLR4, TLR5, TLR7 and TLR11 families) with high branch support within their own clusters confirming the reliability of the tree (Figure 1 and Supplementary Figure 2). Twenty *B. lanceolatum* sequences formed a strongly supported clade distinct from the mccTLR sequences and grouped with the TLR11 family. One TLR (Bl19922) is not clustered with any TLRs, probably because it is an N-terminal truncated sequence. Moreover, six *B. lanceolatum* TLRs, which were identified as mccTLR (invertebrate type) were clustered separately from the main vertebrate branch (Figure 1 and Supplementary Table 4).

**Figure 2:**
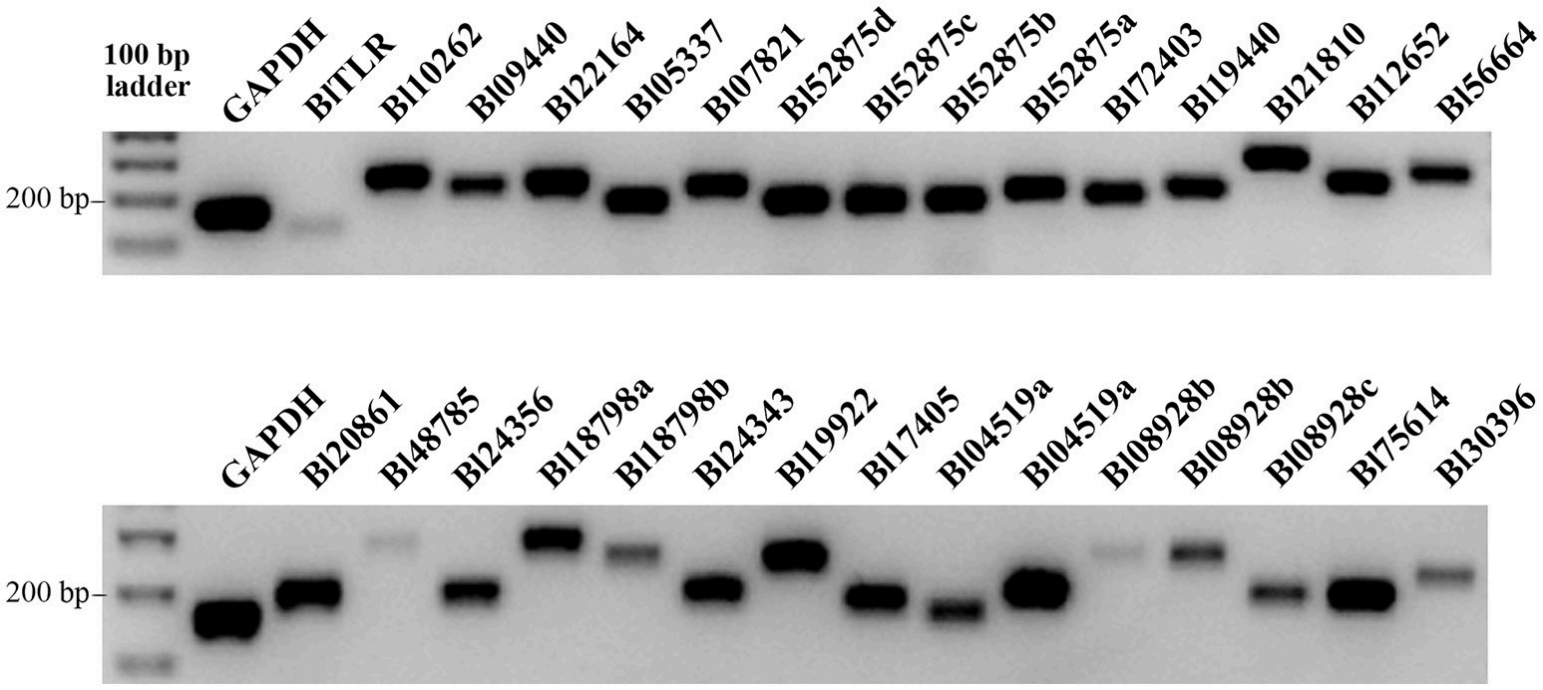
Expression of TLR genes in *B. lanceolatum*. The cDNA used in all amplifications was prepared from whole animals. RT-PCR reactions were accomplished using equal number of cycles, the PCR products were loaded equally on two 1% agarose gels and GAPDH was used as a reference gene. Images were taken with the same exposure time using a Geldoc.

The transcription of the 30 TLRs in *B. lanceolatum* was confirmed by RT-PCR analysis in adult animals. Each primer pair was designed based on the nucleotide sequences reconstructed from transcript sequences of *B. lanceolatum*. We found gene transcription in basal conditions for all the 30 TLRs. The TLRs with gene ID of BlTLR, Bl48785, Bl18798b, Bl08928b and Bl30396 showed a weak transcription while others were strongly expressed (Figure 2). Five of the genes were sequenced using specific primers confirming the identity of these genes (data not shown).

To better understand the *Branchiostoma* TLR evolution, we compared the domain structure of *B. lanceolatum, B. belcheri* and *B. floridae*. Therefore, we identified a total number of 30 TLRs in *B. lanceolatum*, 22 TLRs in *B. floridae* and 37 TLRs in *B. belcheri* (Supplementary Tables 4–6) according to the common TLR pattern. We also discriminated sccTLR and mccTLR in these three species according to the domain structure and phylogenetic analysis (Supplementary Figure 1). There are 3 mccTLRs in *B. floridae*, 5 mccTLRs in *B. belcheri* and 6 mccTLRs in *B. lanceolatum*. In addition, the mccTLRs found in the three *Branchiostoma* species consistently blast with invertebrate type TLRs (Supplementary Tables 4–6). We also studied the number of LRR from each TLR using LRRfinder software. The results showed that the LRR number of TLRs in the three species ranges from 1 to 25.

### Identification and characterization of a novel BLTLR

We focused on the amphioxus TLR11 family described in section The TLR Family in *B. lanceolatum* and specifically in a *B. lanceolatum* TLR sequence (BlTLR) because it was highly similar to the published bbtTLR1 (GenBank: DQ400125.2). This *B. belcheri* gene was annotated as TLR1 based on phylogenetic and functional data (34). Nonetheless, our phylogenetic analysis pointed out that BlTLR was a clear TLR11 family member. TLR11 family includes several teleost specific members (e.g., TLR19 or TLR22) that are not present in mammalian genomes and it is of great interest to know whether they are present in a more basal organism. To begin, we cloned the full-length of this novel BlTLR (GenBank: MG437061) and its 5′ and 3′-UTRs were obtained based on three orthologous found in the *Branchiostoma* genus. The length of the novel BlTLR cDNA is 3,772 bp, containing a 227 bp long 5′UTR, a 2,913 bp ORF (which encodes a putative 970 amino acid-long protein), and a 616 bp long 3′UTR with a putative polyadenylation signal (AATAAA) 17 nucleotides upstream of the poly(A) tail (Supplementary Figure 3). SMART domain analysis predicted that the BlTLR protein has the following domains: a C-terminal TIR domain (from residue 800 to 947), a transmembrane (TM) domain (from residue 752 to 774), a N-terminal signal peptide (first 27 residues), 21 tandem extracellular leucine-rich repeats (LRRs), a leucine rich repeat C-terminal domain (LRRCT) and a LRR N-terminal domain (LRRNT). The domain diagram of BlTLR was made with IBS software (49) and shown in Supplementary Figure 4. The LRRs are flanked by one LRRCT and one LRRNT domain. The BlTLR has only one LRRCT like most of the TLRs found in deuterostomes (sccTLRs). The highly conserved consensus sequence (LxxLxLxxNxL) of each LRR was identified with the LRRfinder (Supplementary Figures 3, 4). Ten potential N-linked glycosylation sites were predicted by NetNGly 1.0: N^101^-N^114^-N^154^-N^163^-N^276^-N^375^-N^393^-N^522^-N^573^-N^632^. The deduced molecular weight of BlTLR protein is 111.3 kDa and the full-length protein showed 78.8% identity with the bbtTLR1 of *B. belcheri*. Three conserved boxes were identified in TIR domain of BlTLR (Supplementary Figure 3). Box 1 and 2 are involved in binding downstream signaling molecules while box 3 is involved in the localization of the receptor through interactions with cytoskeletal elements (50). Importantly, a key residue in box 2 (Proline 681 in human TLR2 sequence) involved in MyD88 signaling was substituted by Ala in the BlTLR sequence (51).

### Expression analysis of BLTLR after LPS and poly I:C treatment

**Figure 3:**
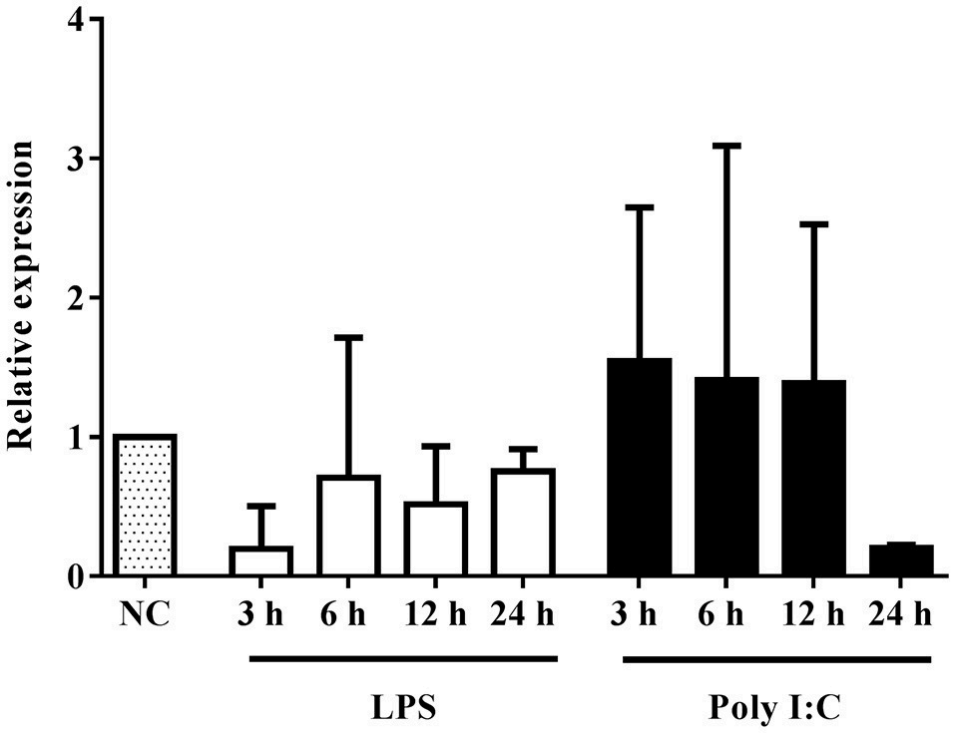
Expression of BlTLR gene after LPS or Poly I:C treatment. Animals previously immersed in 10 μg/ml LPS or 10 μg/ml Poly I:C, were collected at 3, 6, 12 and 24 h. The untreated animals were used as a control and assigned a value of 1 in the histogram. GAPDH was used as a reference gene. The bars indicate mean expression of 3 individual animals ± S.D. Significant differences of mean values were analyzed according to one-way ANOVA followed by Tukey's test.

We performed RT-qPCR to investigate the expression profile of the BlTLR in response to PAMP administration. This approach is often used to identify which family a putative TLR belongs to. Two representative PAMPs of bacterial and viral infection (LPS and Poly I:C, respectively) were used to challenge amphioxus *in vivo*. Amphioxi were immersed in 10 μg/ml LPS or 10 μg/ml Poly I:C to mimic the natural infection route. The gene transcription of BlTLR was analyzed by RT-qPCR in a time course at 3, 6, 12 and 24 h post-immersion (Figure 3). However, no significant differences in gene expression were observed in any of the LPS or Poly I:C-treated groups, indicating that 10 μg/ml LPS or Poly I:C administered by immersion within this time frame could not significantly induce up- or down-regulation of the BlTLR gene in adult amphioxus.

### Subcellular localization of BLTLR in HEK293 cells

**Figure 4:**
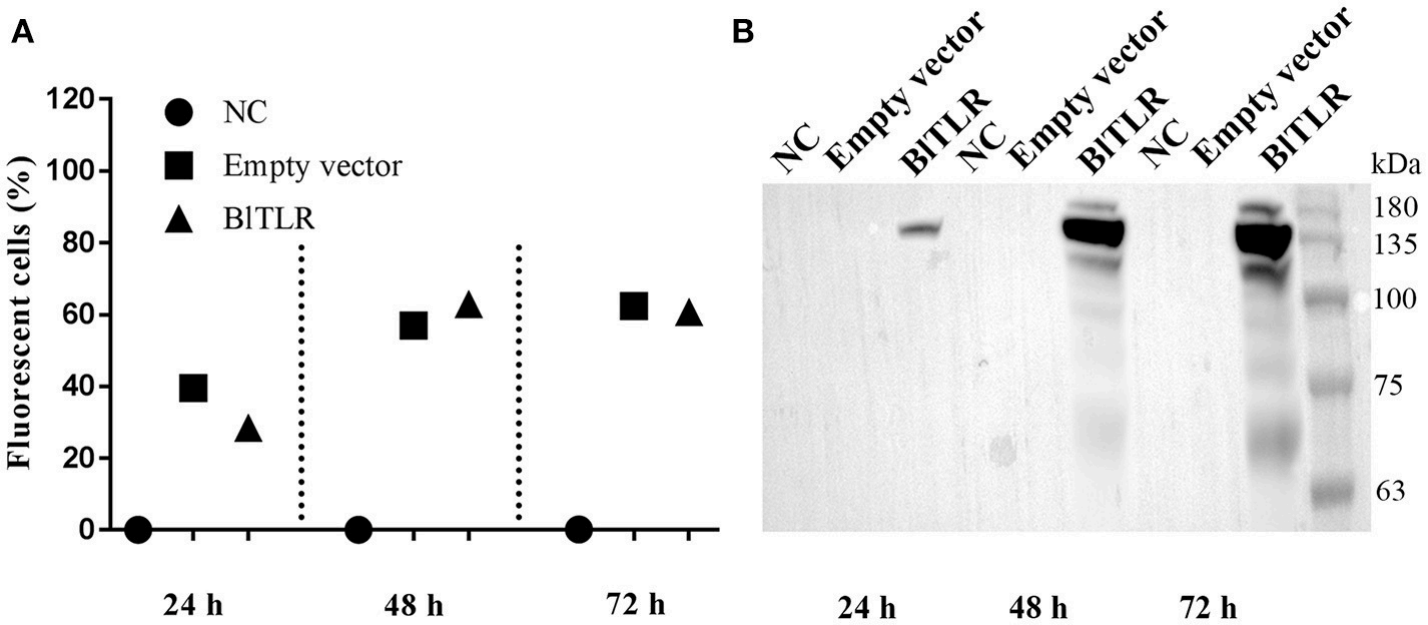
BlTLR expression in HEK293 cells. **(A)** HEK293 cells transfected with an empty vector (pIRES2-EGFP) and a vector expressing BlTLR were analyzed at 24, 48 and 72 h post-transfection by flow cytometry. Non-transfected cells (NC) were used as a control. Transfection efficiency was evaluated as the percentage of GFP positive cells. **(B)** Non-transfected cells, cells transfected with the empty vector and the vector expressing BlTLR with HA tag were analyzed at 24, 48 and 72 h post-transfection by western blot. Protein molecular weight standards (Niborlab) are shown on the right side.

We used HEK293 cells because these cells could be efficiently transfected and they have been extensively used for the study of TLR subcellular localization. Cells were transiently transfected with empty vector and the vector expressing the full-length BlTLR. Flow cytometry analysis showed that cells were successfully transfected at 24, 48 and 72 h and the transfection efficiency at 48 and 72 h (both around 60%) was higher than at 24 h (around 30%) post-transfection (Figure 4A). Western blot analysis confirmed that the BlTLR protein was properly expressed in HEK293 cells, and it was not degraded by intracellular proteases. The BlTLR protein was detected at 24, 48 and 72 h post-transfection (Figure 4B). The transcription levels were much higher at 48 and 72 h than at 24 h which agrees with the cytometry results. The molecular weight of BlTLR protein was around 135 kDa which is slightly bigger than the theoretical one (111.27 kDa). This may be due to post-translational modifications such as glycosylation, phosphorylation, ubiquitination, ubiquitin-like modifications or S-nitrosylation among others.

**Figure 5:**
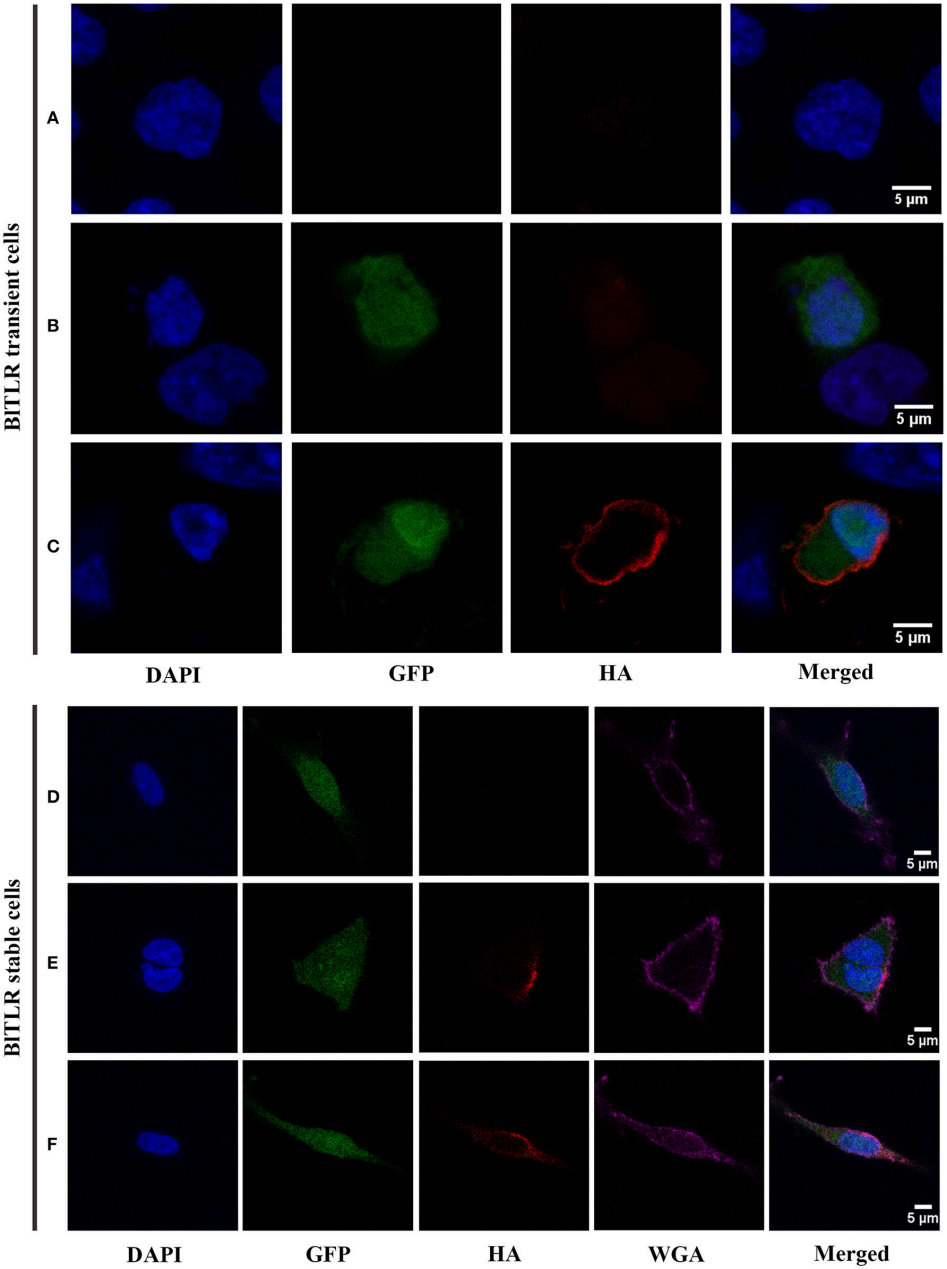
Subcellular localization of BlTLR in HEK293 cells. Confocal images showing HEK293 cells transiently transfected **(A–C)** or stably transfected **(D–F)** with BlTLR. **(A)** Not transfected cells; **(B)** Cells transfected with BlTLR and non-permeabilized; **(C)** Cells transfected with BlTLR and permeabilized with 0.2% Triton X-100. **(D)** BlTLR stable cells not permeabilized; **(E)** BlTLR stable cells permeabilized using freeze and thaw protocol; **(F)** BlTLR stable cells permeabilized with 0.1% Tween-20. Nuclei are stained with DAPI (in blue). Transfected cells were GFP labeled (in green). BlTLR was detected with anti-HA antibody and AF555-conjugated anti-mouse IgG (in red). Plasma membrane was stained with WGA AF647 conjugated (in purple). Figures were analyzed with Fiji software.

To explore the subcellular localization of BlTLR, we overexpressed the HA-tagged BlTLR in HEK293 cells and we visualized the localization using immunofluorescence and confocal microscopy. We did not observe the HA-tagged BlTLR in both transient and stable transfected cells when the cells were not permeabilized (Figures 5B,D). Non-transfected cells were used as a control (Figure 5A). This result indicates that, first, BlTLR might be an intracellular protein; second, BlTLR might localize on the plasma membrane but could not be detected in non-permeabilized cells due to the HA-tag location at the C-terminal. To further understand the localization of BlTLR, we performed the assay with a plasma membrane marker (WGA) and different permeabilization methods. Interestingly, when the cells were permeabilized using different permeabilization methods (from weak to strong), we found that BlTLR was mainly localized on the plasma membrane in both transient and stable transfected cells (Figures 5C,E,F).

### BLTLR could respond to poly I:C in HEK293 cells

**Figure 6:**
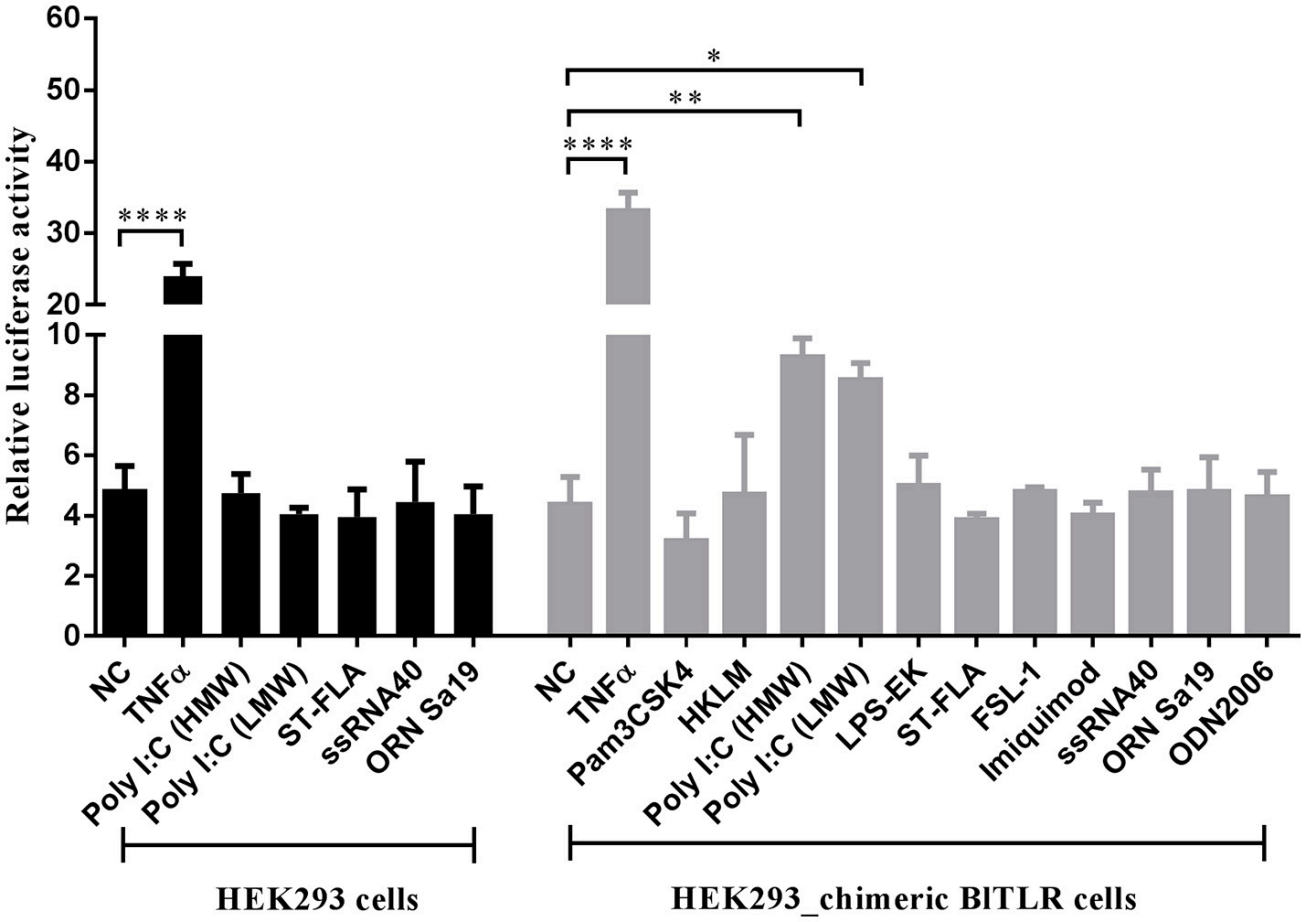
HEK293 cells expressing chimeric BlTLR induce the activation of NF-κB in response to Poly I:C. HEK293 chimeric BlTLR stable cells were treated with 11 different ligands (gray columns). Non-transfected HEK293 cells were treated with five potential ligands (black columns). Non-treated cells (NC) and cells treated with human TNFα (20 ng/ml) were used as negative and positive controls, respectively. The luciferase activity was expressed as the ratio of NF-κB-dependent firefly luciferase activity divided by Renilla luciferase activity. Bars represented mean ± S.D. Significant differences of mean values were analyzed according to one-way ANOVA followed by Tukey's test. ^*^P < 0.05; ^**^P < 0.01; ^****^P < 0.0001.

Mammalian TLRs can transactivate the transcription factor NF-κB in response to ligand binding. Usually, each TLR has a restricted PAMPs preference (Supplementary Table 3) and the NF-κB reporter assay allows functional discrimination between TLRs. To shed light on the role of novel BlTLR in PAMPs recognition, a HEK293 cell line stably expressing BlTLR was generated. However, the BlTLR stable cells could not activate the NF-κB promoter stimulated by any of the tested PAMPs (data not shown). To further study the receptor activity, we design a chimeric receptor fusing the ectodomain of BlTLR with the TIR domain of human TLR2 and we generated a stable cell line. This approach has been used before to ensure a correct downstream signaling avoiding the differences in the set of adaptors and accessory proteins between vertebrates and non-vertebrates (34, 52). The chimeric BlTLR stable cells responded to Poly I:C (LMW and HMW) which usually binds to TLR3 or TLR22. Conversely, other ligands, including Pam2CSK4 for TLR1/2, HKLM for TLR2, LPS for TLR4, flagellin for TLR5, FSL-1 for TLR2/6, imiquimod for TLR7, ssRNA for TLR8, ODN2006 for TLR9, ORN Sa19 for TLR13 (mouse) failed to induce NF-κB transactivation (Figure 6). Human recombinant TNFα was used as a positive control since it is a well-known NF-κB activator. In order to confirm that the up-regulation of luciferase activity is due to the Poly I:C recognition by the chimeric BlTLR but not by endogenous TLRs, we performed the luciferase assay using chimeric BlTLR stable cells and HEK293 cells without chimeric BlTLR. The NF-κB luciferase activity was up-regulated in chimeric BlTLR stable cells with respect to HEK293 cells treated with Poly I:C (LMW and HMW; Figure 6).

Our results showed that the novel BlTLR localized at the plasma membrane and responded to Poly I:C. These characteristics are only compatible with TLR22, thus we postulated that the novel receptor is a TLR22-like receptor. The alignment of BlTLR with 12 teleost TLR22 sequences showed that BlTLR had 27.8–30.8% of identity with fish TLR22 (Supplementary Table 7).

To further explore the phylogenetic relationship of BlTLR, BbtTLR1 and vertebrate TLRs, phylogenetic trees were constructed based on full-length protein sequence using maximum-likelihood analysis (Supplementary Figure 5). As expected, *D. melanogaster* Tolls clustered independently and all the vertebrate TLRs clustered into six clades. Furthermore, BlTLR clustered in the same TLR11 family clade together with BbtTLR1. The SH-aLRT support (%) and ultrafast bootstrap support (%) are 94.6 and 94 (Supplementary Figure 5). This result indicates that BlTLR is very likely to be a member of TLR11 family and could be identified as TLR11, 12, 13, 19, 20, 21 22, or 23. Overall, all the results strongly support the identification of the novel receptor that carries the TLR22 function (BlTLR22-like).

## Discussion

TLRs play crucial roles in the innate immune system by recognizing PAMPs from pathogens in vertebrates. In addition, TLRs have multiple functions ranging from developmental signaling to cell adhesion in protostomes (48). The study of TLRs may help to understand the role of TLR-mediated responses which could increase our range of strategies to treat infectious diseases and manipulate immune responses by drug intervention (53). From the evolutionary point of view, TLRs are conserved across invertebrates to vertebrates and absent from non-animal phyla (plants and fungi). However, there are vast structural and functional divergences in TLRs between invertebrates and vertebrates (15). In vertebrates, humans and mice have 10 and 12 TLRs, respectively and at least 16 TLRs have been identified in teleost; in urochordates, *Ciona intestinalis* has only two TLRs (54) whereas *Ciona savignyi* has between 8 and 20 (16); but in cephalochordates, *B. floridae* has an expansion of 48 TLRs according to Huang et al. (33). This expansion of TLRs in invertebrate deuterostomes remains to be understood by a comprehensive and thorough study of TLRs evolution. Amphioxus is a good model to study the invertebrate-chordate to vertebrate transition and the evolution of vertebrates. Therefore, studying TLR functions in such organism could improve our understanding of the ancestral innate immune system of vertebrates.

In this study, we identified 30 TLRs in *B. lanceolatum*, 22 TLRs in *B. floridae* and 37 TLRs in *B. belcheri* according to the basic TLR structure: “LRR+TM+TIR.” Differences in the total number of *B. floridae* TLRs between Huang et al. and our data probably reflects discrepancies in the consensus of what is the basic structure of TLRs. Our stringent rule includes only those putative receptors with a TIR domain, a transmembrane domain and at least one LRR domain, known as true TLRs (15). Our available transcriptomic data maybe do not include all the possible TLRs. Probably the total number of TLRs in the 3 species of lancelet should be similar. Among them, we identified 6 mccTLRs in *B. lanceolatum*, 3 mccTLRs in *B. floridae* and 5 mccTLRs in *B. belcheri*. This finding is different from the observation by Huang et al. concerning amphioxus TLR family: it has a high rate of domain combination acquisition and therefore a high number of TLRs (prediction of 36 sccTLRs and 12 mccTLRs) (33). Importantly, Bányai and Patthy provided evidence to dispute that the rate of protein innovation is exceptionally high in lancelets. They surmised these high rates are likely due to gene prediction errors (55). This might be the reason why there are less TLRs found in our study than the genomic prediction. Interestingly, if we remove 3 mccTLR sequences in *B. floridae* from our list, the total number of TLRs would be the same as reported by Tassia et al. which identified 19 TLRs (56). Moreover, the RT-PCR analysis showed that all the 30 TLRs of *B. lanceolatum* were truly expressed in adult animals. Our work shows that amphioxus and vertebrates share a conserved TLR framework in terms of protein structure. On the other hand, amphioxus TLRs maintain some features of invertebrates, such as the mccTLRs which are mainly found in protostomes (15). The function of remaining TLRs in PAMPs recognition remains unclear and needs further investigation.

We cloned the full-length sequence of BlTLR22-like from Mediterranean amphioxus (*B. lanceolatum*). The full-length protein showed the highest identity (78.8%) with bbtTLR1 of *B. belcheri* that was annotated by the authors as a TLR1 based on the expression analysis after PAMPs injection *in vivo* (34) but the authors did not study the subcellular localization or the direct ligand specificity. The domain analysis of BlTLR22-like protein sequence showed that it has a complete vertebrate-like ectodomain including a LRRCT, 21 LRRs and a LRRNT. The ectodomain forms a horseshoe structure to bind the specific PAMPs including the LRRCT that is responsible for dimerization which is necessary for complete ligand binding (57–59). The full-length protein sequences of BlTLR22 are highly similar to the TLR22 of many fish species, suggesting that they may have similar ligand recognition, intracellular signal transduction pathway mechanisms and localization.

In mammals, TLRs can be divided into two main groups according to localization: on the cell surface or in intracellular compartments (60). Among human TLRs, the ones located at the plasma membrane (TLR1, 2, 4, 5 and 6) recognize microbial pathogenic components of the cell wall, while the others (TLR3, 7, 8 and 9) located intracellularly in endosomes or lysosome recognizing nucleic acids (4). However, the above ligand recognition pattern in non-mammalian organisms may be not always as in mammals. For instance, mouse TLR13 recognizes a conserved 23S ribosomal RNA (rRNA) from bacteria in the endolysosomal compartment (11). In teleost, TLR13 of *M. croaker* could respond to Poly I:C both *in vivo* and *in vitro* and is localized in the cytoplasm of HeLa cells (19). Fugu TLR22 recognizes long-sized dsRNA on the cell surface whereas TLR3 resides in the endoplasmic reticulum and recognizes relatively short-sized dsRNA (20). TLR22 of grass carp (*C. idella*) recognizes Poly I:C stimulation in CIK (*C. idella* kidney) cell line and is localized on the cell membrane (21). In our study, immunofluorescence and confocal microscopy showed that BlTLR22-like is mainly localized on the plasma membrane.

In mammals, TLRs can recognize specific PAMPs with high levels of sensitivity (61). To test B1TLR22-like ligand specificity, we performed different assays with commercially available mammalian TLRs ligands, using NF-κB activity as a reporter. We could not observe significant differences of NF-κB activation in HEK293 cells expressing BlTLR22-like. There are different possible explanations but apart from problems with protein expression levels, intracellular degradation or incorrect trafficking, the two most likely reasons could be: (1) BlTLR22-like could not directly recognize PAMPs and the recognition process might require the assistance of other proteins that are specific for amphioxus and are not present in a mammalian system. For instance, *D. melanogaster* Tolls do not bind any PAMPs directly (62) and mammalian TLR4 cannot recognize LPS without the assistance of MD2 and CD14 (63) or; (2) BlTLR22-like has a TIR domain that interacts with a species specific adaptor protein not present in mammalian cells. This hypothesis could be supported by the fact that P681 (human TLR2), extremely important to activate MyD88 signaling pathways in mammals (51), was not present in BlTLR22-like neither in BbtTLR1 (Supplementary Figure 3). Thus, we could hypothesize that the absence of this Pro in the TLR22 sequence (Ala in BlTLR22-like) explains why the TIR domain of BlTLR22-like cannot activate MyD88 dependent signaling pathway in HEK293 unless we combine the ectodomain of TLR22 with the human TLR2 TIR domain. To test this hypothesis, we designed a chimeric protein containing the ectodomain and transmembrane domain of BlTLR22-like fused to the human TLR2 TIR domain and we tested whether it could respond to ligand stimulation when stably transfected in HEK293 cells. Indeed, the cells expressing chimeric BlTLR22-like activated significantly the NF-κB reporter in response to both LMW and HMW Poly I:C. The magnitude of the stimulation is similar to other published data. For instance, Ji et al. characterized the activation of IFN and NF-κB pathways by a teleost TLR19, and they found similar fold changes (around 2-fold change) as in our data (2.12 ± 0.1-fold change Poly I:C HMW and 1.95 ± 0.09-fold change Poly I:C LMW) (64). Other authors also have obtained similar fold-changes in the NF-κB reporter assay (65, 66). On the other hand, Voogdt et al. showed an extremely high activation of the NF-κB signaling pathway after flagellin stimulation but the main difference with our approach is that they used cells stably expressing NF-κB reporter (67). Poly I:C is a specific ligand of vertebrate TLR3 including many fish species (20, 23, 65, 68), of *M. croaker* TLR13 (19) and of different fish TLR22 (20–22, 24, 69).

The phylogenetic analysis of BlTLR22-like protein sequence and representative vertebrate TLR protein sequences revealed that BlTLR22-like clusters with the vertebrate TLR11 family. Interestingly, the phylogenetic analysis of *B. floridae* TIR domain and vertebrate TLRs has indicated that 33 variable-type TLRs show a paraphyletic relationship with the vertebrate TLR11 lineage (33). The TLR11 family is represented in humans only by a pseudogene and the major divisions of the TLR11 family are clearly very ancient (16). Moreover, BlTLR22-like has a single domain structure of the ectodomain which should be classified into TLR13 subfamily (Supplementary Table 8) according to the ectodomain architecture analysis of vertebrate TLRs (17). Taken together with its plasma membrane localization and functional analysis, we could further confirm the annotation of this TLR as an ortholog of vertebrate TLR11s, carrying a TLR22-like function and probably share a common ancestor with the fish specific TLR22. Overall, we provide evidence suggesting that TLR22 function may be an ancient and evolutionarily conserved antiviral response which emerged in Chordates.

## Data availability statements

The datasets for this manuscript are not publicly available yet because the paper in which all these data are presented is in the final review process. Once the paper is published all the transcriptomic and genome data will be freely available, but until then requests to access the datasets should be directed to Dr. Jordi Garcia-Fernàndez (jordigarcia@ub.edu) and Dr. Hector Escrivà (hescriva@obs-banyuls.fr).

## Ethics statement

All experimental procedures were approved by the Human and Animal Experimentation Ethics Committee of the Universitat Autònoma de Barcelona and were done in strict accordance with the recommendations of the European Directive (2010/63/EU) on the protection of animals used for scientific purposes.

## Author contributions

JJ performed all the experiments and phylogenetic analysis. DR-V and AB helped to design the phylogenetic analysis. EN-P, CH-Ú and JG-F provided the TLR gene candidates from the transcriptome of *B. lanceolatum* and assisted with the phylogenetic analysis. JL provided the reporter assay plasmids and helped in the reporter assay design. HE provided the amphioxus and partial sequence of BlTLR from the genome scaffold of *B. lanceolatum*. NR and JJ designed the experiments. JJ and NR prepared all the tables and figures and wrote the manuscript. All authors contributed to the correction of the final manuscript.

### Conflict of interest statement

The authors declare that the research was conducted in the absence of any commercial or financial relationships that could be construed as a potential conflict of interest.

## Supplementary material

Supplementary Figure 1 Phylogenetic analysis of *B. floridae, B. belcheri* and *B. lanceolatum* TLRs. The phylogenetic tree was constructed by maximum-likelihood method (IQ-TREE) using TIR domain sequences of *B. floridae, B. belcheri, B. lanceolatum, S. kowalevskii* and representative vertebrate TLRs. *D. melanogaster* Toll sequence was used as an outgroup to root the tree. Sequences were aligned by MAFFT with G-INS-i method and the best evolutionary model was established by ModelFinder according to BIC. Branch support was calculated running 1,000 replicates of the SH-aLRT and ultrafast bootstrap and they are represented as percentages at the tree nodes. The tree was generated in FigTree. Outgroup, mccTLRs and six vertebrate TLR families (by colors) were shown in the figure. BlTLR is indicated by a red arrow.

Supplementary Figure 2 Complete phylogenetic analysis of *B. lanceolatum* TLRs. The phylogenetic tree was constructed by IQ-TREE using full-length protein sequences. This tree is a more detailed version of the tree shown in Figure 1. All the values of SH-aLRT support and ultrafast bootstrap support are shown at the tree nodes. Outgroup, mccTLRs and 6 vertebrate TLR families (highlighted in different colors) are shown. The red arrow indicates BlTLR. Additional information about the sequences can be found in Supplementary Table 2, Supplementary Datas 1, 2.

Supplementary Figure 3 Nucleotide and deduced amino acid sequences of BlTLR. Predicted transcription start site (TSS) is marked with a curved arrow. TATA box is boxed with a rectangle. The putative STAT5 and APIB transcription factor binding sites have a thick underline. The start codon (ATG), the stop codon (TAA) and the polyadenylation signal sequence (AATAAA) are in bold. The predicted signal peptide and the transmembrane region are underlined. The potential N-linked glycosylation sites are underlined and in bold. LRRCT domain predicted by LRRfinder is double underlined. The TIR domain predicted by SMART is underlined and highlighted in gray. The consensus sequence of LRR domain predicted by LRRfinder is highlighted in gray. The three consensus sequences of Toll/interleukin-1 receptor homology domain were boxed and underlined in gray: box 1(FDAFISY), box 2 (GYKLC—RD—PG) and box3 (a conserved W surrounded by basic residues).

Supplementary Figure 4 Predicted domain architecture of BlTLR protein. The domain structure was predicted using the SMART program. Signal peptide (SP), leucine-rich repeat N-terminal domain (LRRNT), leucine-rich repeat (LRR), leucine rich repeat C-terminal domain (LRRCT), Transmembrane domain (TM) and Toll/interleukin-1 receptor (TIR) domain are indicated in figure. Figure was prepared with IBS software.

Supplementary Figure 5 Phylogenetic analysis of BlTLR. The phylogenetic tree was constructed by maximum-likelihood method (IQ-TREE) using full-length protein sequences. BlTLR, BbtTLR1 and representative vertebrate TLR sequences were used in the analysis. *D. melanogaster* Toll was used as an outgroup to root the tree. Sequences were aligned with MAFFT choosing L-INS-i method and the alignments were trimmed using TrimAL with “Automated 1” mode. The best evolutionary model was established by ModelFinder according to BIC. One-thousand replicates of the SH-aLRT support and ultrafast bootstrap support are represented as percentages at the tree nodes. The tree was generated in FigTree. Outgroup and six vertebrate TLR families (by colors) are shown in figure. BlTLR is indicated by a red arrow.

Supplementary Table 1 Primers used for RT-PCR analysis.

Supplementary Table 2 Vertebrate and invertebrate protein sequences used in the phylogenetic analysis.

Supplementary Table 3 TLR ligands used in this study.

Supplementary Table 4 TLRs in *B. lanceolatum*.

Supplementary Table 5 TLRs in *B. floridae*.

Supplementary Table 6 TLRs in *B. belcheri*.

Supplementary Table 7 Protein sequence identity of BlTLR and fish TLR22.

Supplementary Table 8 Ectodomain architecture of vertebrate TLRs and BlTLR.

Supplementary Data 1 TLR sequences of *L. variegatus* and *S. kowalevskii* used in the phylogenetic analysis. The TIR domain of each TLR is highlighted in yellow.

Supplementary Data 2 Identified DNA and putative protein sequences of TLRs in *B. lanceolatum*. The TIR domain of each TLR is highlighted in yellow.
