# Supplementary figures and images for: Characterization of the TLR Family in Branchiostoma lanceolatum and Discovery of a Novel TLR22-Like Involved in dsRNA Recognition in Amphioxus
Source: Front Immunol. 2018 Nov 2;9:2525. doi: 10.3389/fimmu.2018.02525 (PMC6224433; doi:10.3389/fimmu.2018.02525)

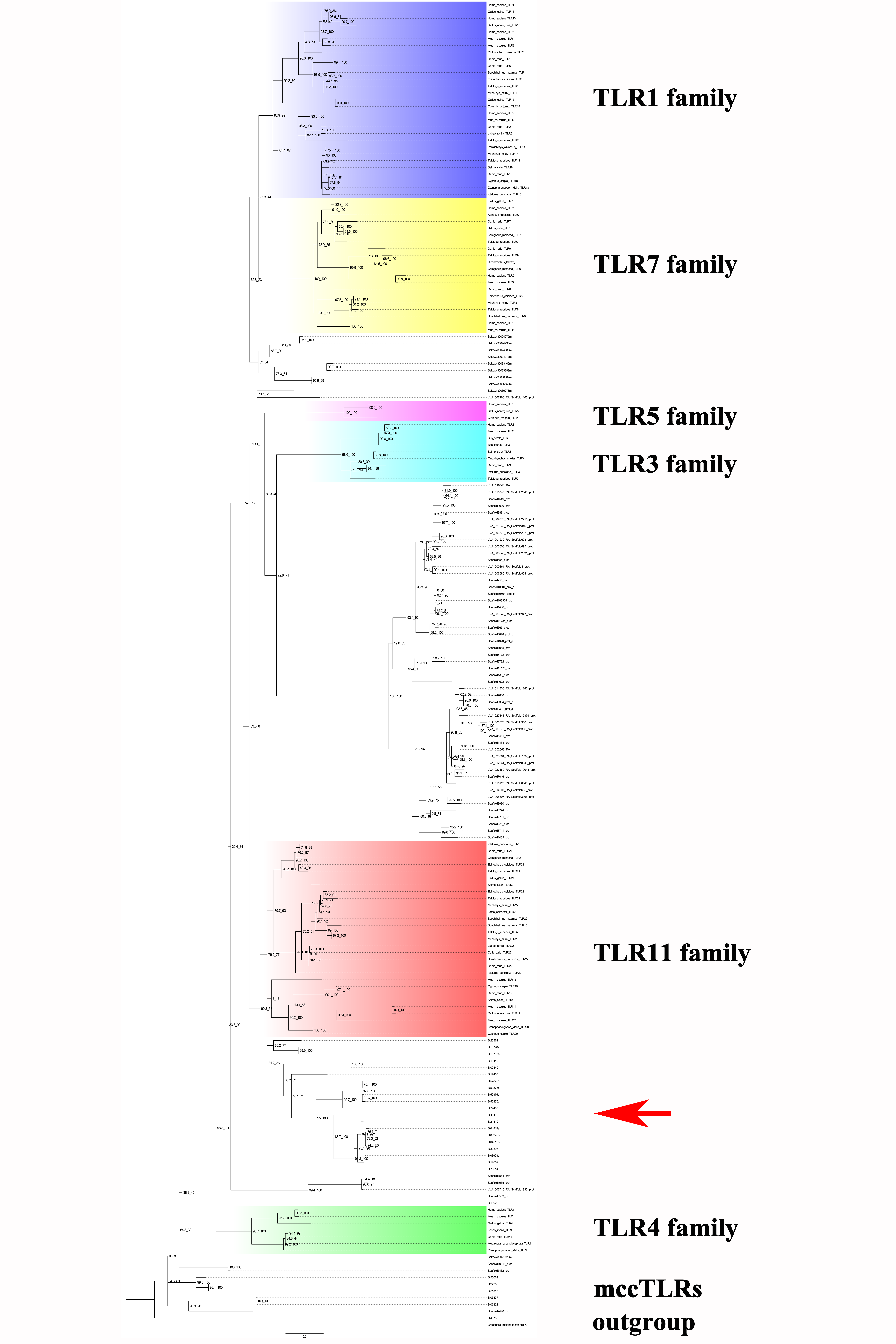

Supplement: Supplementary Figure 2 — Complete phylogenetic analysis of B. lanceolatum TLRs. The phylogenetic tree was constructed by IQ-TREE using full-length protein sequences. This tree is a more detailed version of the tree shown in Figure 1. All the values of SH-aLRT support and ultrafast bootstrap support are shown at the tree nodes. Outgroup, mccTLRs and 6 vertebrate TLR families (highlighted in different colors) are shown. The red arrow indicates BlTLR. Additional information about the sequences can be found in Supplementary Table 2, Supplementary Datas 1, 2. [file Image_2.TIF]

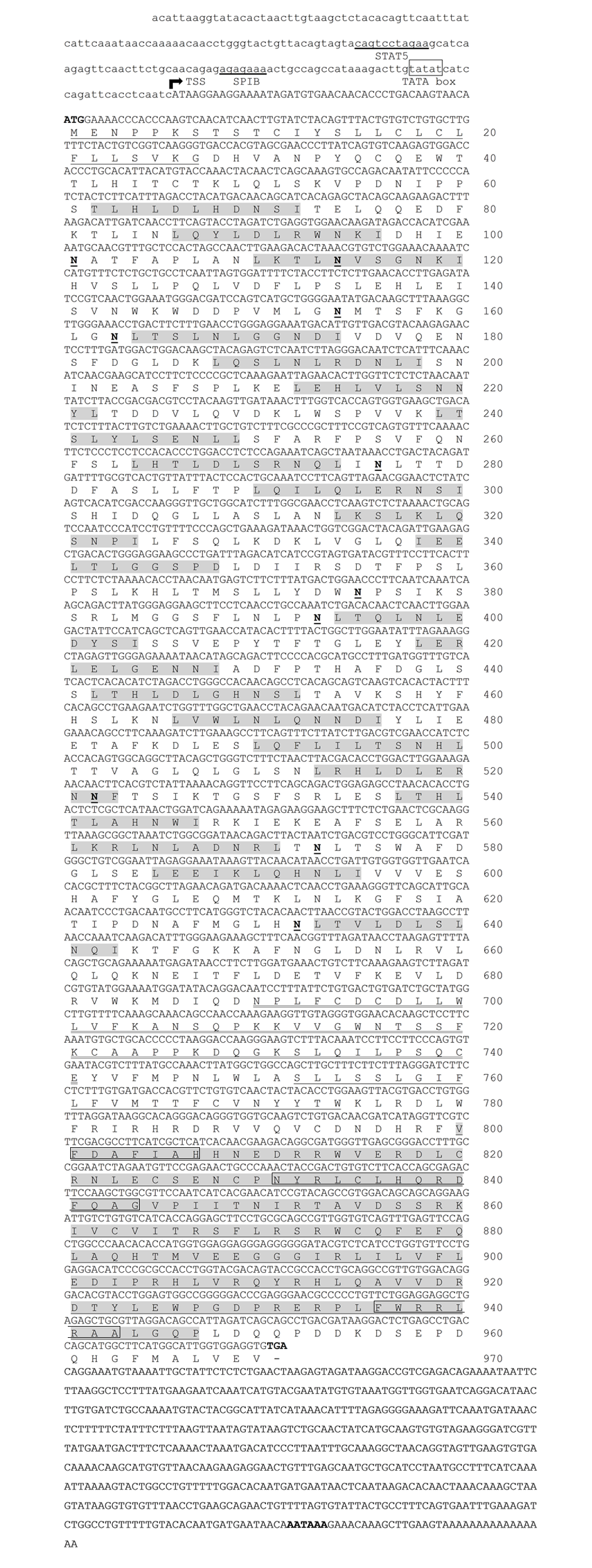

Supplement: Supplementary Figure 3 — Nucleotide and deduced amino acid sequences of BlTLR. Predicted transcription start site (TSS) is marked with a curved arrow. TATA box is boxed with a rectangle. The putative STAT5 and APIB transcription factor binding sites have a thick underline. The start codon (ATG), the stop codon (TAA) and the polyadenylation signal sequence (AATAAA) are in bold. The predicted signal peptide and the transmembrane region are underlined. The potential N-linked glycosylation sites are underlined and in bold. LRRCT domain predicted by LRRfinder is double underlined. The TIR domain predicted by SMART is underlined and highlighted in gray. The consensus sequence of LRR domain predicted by LRRfinder is highlighted in gray. The three consensus sequences of Toll/interleukin-1 receptor homology domain were boxed and underlined in gray: box 1(FDAFISY), box 2 (GYKLC—RD—PG) and box3 (a conserved W surrounded by basic residues). [file Image_3.TIF]

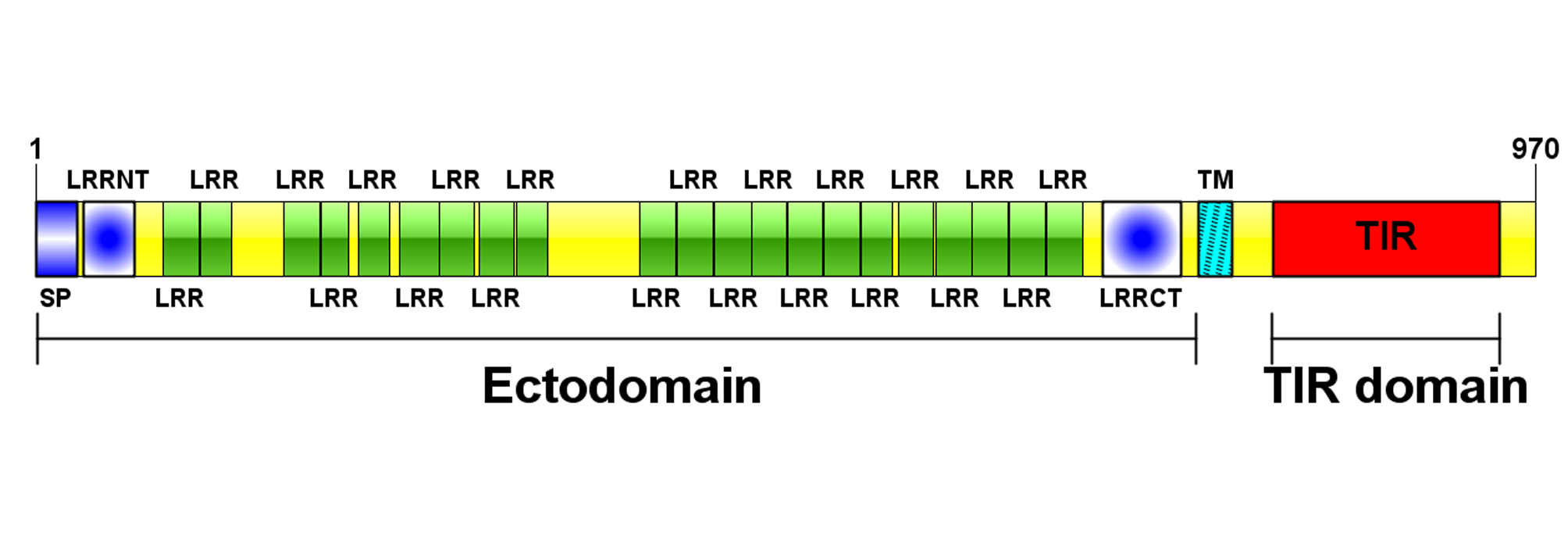

Supplement: Supplementary Figure 4 — Predicted domain architecture of BlTLR protein. The domain structure was predicted using the SMART program. Signal peptide (SP), leucine-rich repeat N-terminal domain (LRRNT), leucine-rich repeat (LRR), leucine rich repeat C-terminal domain (LRRCT), Transmembrane domain (TM) and Toll/interleukin-1 receptor (TIR) domain are indicated in figure. Figure was prepared with IBS software. [file Image_4.TIF]

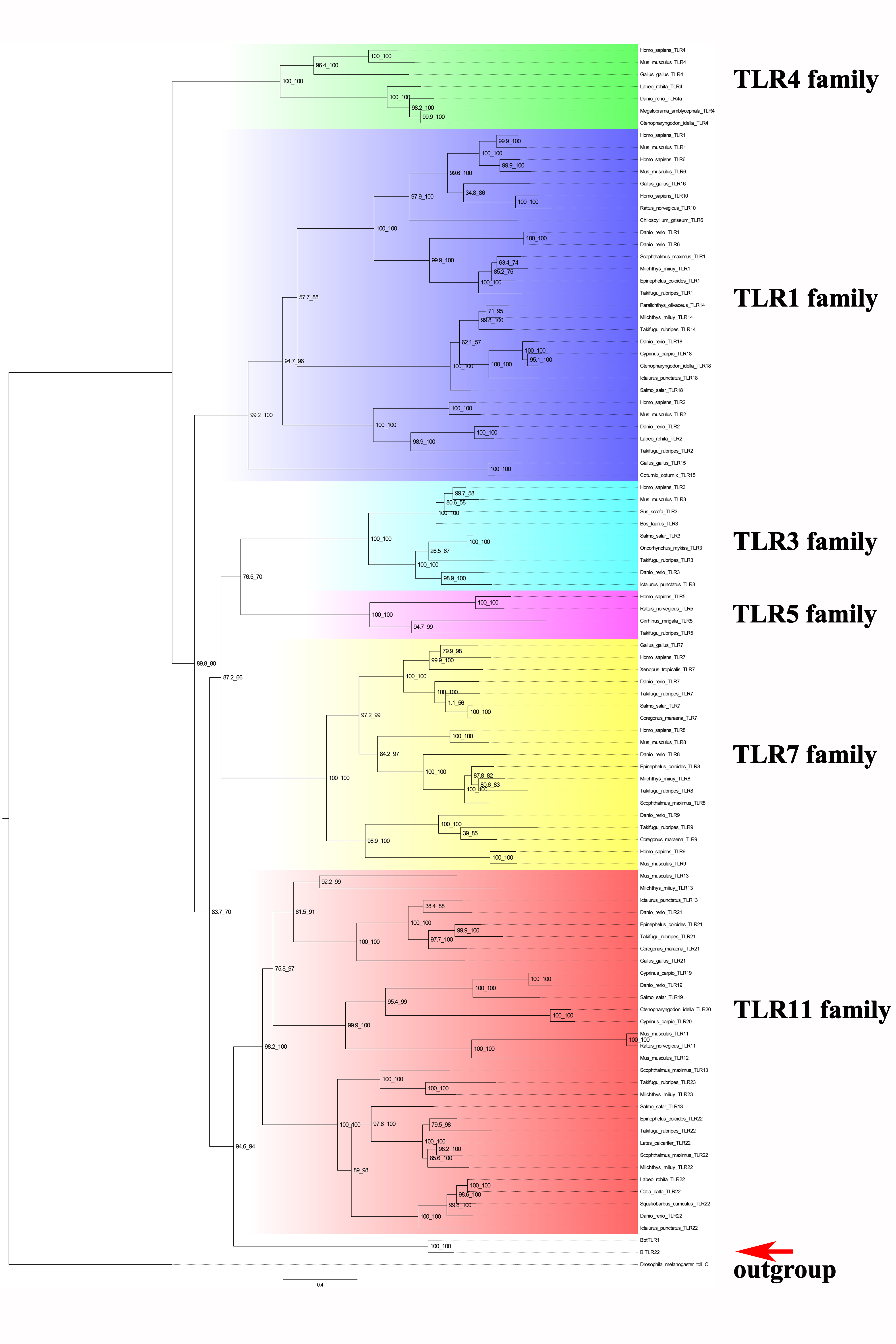

Supplement: Supplementary Figure 5 — Phylogenetic analysis of BlTLR. The phylogenetic tree was constructed by maximum-likelihood method (IQ-TREE) using full-length protein sequences. BlTLR, BbtTLR1 and representative vertebrate TLR sequences were used in the analysis. D. melanogaster Toll was used as an outgroup to root the tree. Sequences were aligned with MAFFT choosing L-INS-i method and the alignments were trimmed using TrimAL with “Automated 1” mode. The best evolutionary model was established by ModelFinder according to BIC. One-thousand replicates of the SH-aLRT support and ultrafast bootstrap support are represented as percentages at the tree nodes. The tree was generated in FigTree. Outgroup and six vertebrate TLR families (by colors) are shown in figure. BlTLR is indicated by a red arrow. [file Image_5.TIF]
