# Supplementary material for: Characterization of the TLR Family in Branchiostoma lanceolatum and Discovery of a Novel TLR22-Like Involved in dsRNA Recognition in Amphioxus
Source: Front Immunol. 2018 Nov 2;9:2525. doi: 10.3389/fimmu.2018.02525 (PMC6224433; doi:10.3389/fimmu.2018.02525)
Supplement: Supplementary Table 1 — Primers used for RT-PCR analysis. [file Table_1.DOCX]

***Supplementary Tables***

**Characterization of the TLR family in *Branchiostoma lanceolatum* and discovery of a novel TLR22-Like involved in dsRNA recognition in amphioxus**

**Jie Ji^1^, David Ramos-Vicente^2,3^, Enrique Navas-Pérez^4^, Carlos Herrera-Úbeda^4^, José Miguel Lizcano^5^, Jordi Garcia-Fernàndez^4^, Hector Escrivà^6^, Àlex Bayés^2,3^ and Nerea Roher^1*^**

*** Correspondence:** Corresponding Author: nerea.roher@uab.cat.

**Supplementary Table 1. Primers used for RT-PCR analysis**

| **Gene ID** | **Primer sequence (5’-3’)** | | **T_m_ (ºC)** | **Amplicon size (bp)** |
| --- | --- | --- | --- | --- |
| GAPDH | For | CCCCACTGGCCAAGGTCATCA | 73.5 | 154 |
|  | Rev | GCTGGGATGATATTCTGGTGGGC | 71.3 |  |
| BlTLR | For | TCACACGCTTTCTACGGCTT | 64.8 | 122 |
|  | Rev | A AGGCTTAGGTCCAGTACGGT | 61.5 |  |
| Bl10262 | For | CCACCAATGAAAGAGCTGCG | 68.0 | 245 |
|  | Rev | GATGGCAGACCACCAAATGC | 67.7 |  |
| Bl09440 | For | AAACCGCTTATCCTCCGTGG | 67.1 | 200 |
|  | Rev | TTGCATATCTCCGTGGCGTT | 67.5 |  |
| Bl22164 | For | AGAACTGCACGGACGACATT | 64.7 | 217 |
|  | Rev | GTAGAACTGAACACGGGCGA | 65.2 |  |
| Bl05337 | For | GACACTGCCGACTCTCACAT | 63.1 | 173 |
|  | Rev | GTGGTAGAGTCACTTGCGCT | 62.1 |  |
| Bl07821 | For | CCCGAGAAAACGTACGACAT | 63.8 | 191 |
|  | Rev | AGTGTTCTCCTGCTGCTGGT | 64.1 |  |
| Bl52875d | For | GTAGGTGGTGTGCCGATCTT | 63.9 | 169 |
|  | Rev | GAACCAGGCTCACTCTACGC | 63.9 |  |
| Bl52875c | For | GTCCTGGGCTGATGATCACC | 67.0 | 159 |
|  | Rev | AAAGAGGTCGTTATGGCGCA | 66.7 |  |
| Bl52875b | For | TGGTTCATATCCTGGGCTGC | 67.1 | 167 |
|  | Rev | AAAGAGGTCGTTATGGCGCA | 66.7 |  |
| Bl52875a | For | TGCGTGAACTAACTCTGTCGG | 65.3 | 209 |
|  | Rev | AAGTGCCTCTTGCAAGTTTGG | 65.4 |  |
| Bl72403 | For | ACTACGAAATGCGAGCGTCA | 65.8 | 183 |
|  | Rev | ATAGCGGCCTACCCTTCTCA | 64.7 |  |
| Bl19440 | For | TCTTGGAGAAGATTCCTCGGC | 66.7 | 188 |
|  | Rev | TTCGTCTCCCATTCCACCATC | 68.2 |  |
| Bl21810 | For | GGGAAGCGTCTCATTCTCGT | 65.6 | 274 |
|  | Rev | TGGGGCCATGGATCTGTACT | 66.6 |  |
| Bl12652 | For | CCACTTTTCTCGCAGTCGGT | 66.5 | 215 |
|  | Rev | ACTCAGATCCAGGGTGACGA | 64.9 |  |
| Bl56664 | For | CAGCCATCGAAGAGAACGGA | 67.7 | 256 |
|  | Rev | AATCGTACAAGAGGCCGGAG | 65.2 |  |
| Bl20861 | For | AAACGTCAAGAAGGGCTCGT | 64.9 | 190 |
|  | Rev | CAAACCTCGCAGACTCCACA | 66.5 |  |
| Bl48785 | For | AGAATTTCGTGGACAGCGAGT | 64.9 | 263 |
|  | Rev | GTCAATGTGCTCAGAGTCGGT | 64.3 |  |
| Bl24356 | For | AGTTGAACTTGACCCCAGGC | 65.3 | 195 |
|  | Rev | AGGTGCCGTAAGTGTTCTGG | 64.0 |  |
| Bl18798a | For | AGTGCAAATCCACAGGTTGG | 64.9 | 286 |
|  | Rev | ATCCCGTAGGTTCAGCAATGTT | 65.5 |  |
| Bl18798b | For | GGCGTTTGTATCGATTCCG | 65.2 | 254 |
|  | Rev | AGACCGCGAGTTTATGCAGT | 63.7 |  |
| Bl24343 | For | AATTGTAGCCAGACGAGCCC | 65.3 | 190 |
|  | Rev | ATGCTGGGAGGATGTCGAAC | 66.4 |  |
| Bl19922 | For | ACATAATCACTCGGGCTCGG | 66.1 | 260 |
|  | Rev | TGCAGCTACAGTCAAAGGGG | 65.3 |  |
| Bl17405 | For | CTGGGTCATTCTGCTGGGAT | 66.3 | 178 |
|  | Rev | TGGCTAGTTTGCGAATCCTGT | 65.7 |  |
| Bl04519a | For | GGAGACCGAAGATTGGTGAA | 64.0 | 160 |
|  | Rev | GCAAAGTCCCGTTCGTGTAT | 63.8 |  |
| Bl04519b | For | GAGGACGTCATGTGGGTTGT | 65.0 | 194 |
|  | Rev | AACTCGTACTCGCACCACTG | 63.0 |  |
| Bl08928a | For | GACGTGAGGAAACGACCGC | 68.2 | 244 |
|  | Rev | GACCATCTTCACCATCTCCAAAC | 65.6 |  |
| Bl08928b | For | AGCATGTTCTTCGGCAAGGA | 67.0 | 250 |
|  | Rev | TGACGTCCCGGTTGTTGTAG | 65.9 |  |
| Bl08928c | For | CGTCTGTTCGGGTTGAGGAA | 67.4 | 189 |
|  | Rev | CCAGTTCGCTACCATGTCGT | 65.1 |  |
| Bl75614 | For | ATGTTTGAGCAAGGGGGAGG | 67.3 | 193 |
|  | Rev | GAGGCAAAGGTTCACCCAGA | 66.4 |  |
| Bl30396 | For | TTCTTACAACGACGCCTGCT | 64.8 | 226 |
|  | Rev | GGGCTATCGGGGTGTGTATG | 66.2 |  |

**Supplementary Table 2. Vertebrate and invertebrate protein sequences used in the phylogenetic analysis.**

| **Gene name** | **Species** | **GenBank ID/NCBI reference** |
| --- | --- | --- |
| TLR1 | *Homo sapiens* | AAC34137.1 |
|  | *Mus musculus* | AAG35062.1 |
|  | *Danio rerio* | AAI63271.1 |
|  | *Scophthalmus maximus* | ANS71060.1 |
|  | *Takifugu rubripes* | AAW69368.1 |
|  | *Miichthys miiuy* | AKJ66261.1 |
|  | *Epinephelus coioides* | AEB32452.1 |
| TLR2 | *Danio rerio* | AAQ90474.1 |
|  | *Homo sapiens* | AAC34133.1 |
|  | *Mus musculus* | AAF04277.1 |
|  | *Takifugu rubripes* | AAW69370.1 |
|  | *Labeo rohita* | ADQ74644.1 |
| TLR3 | *Homo sapiens* | AAC34134.1 |
|  | *Mus musculus* | AAK26117.1 |
|  | *Salmo salar* | AKE14222.1 |
|  | *Danio rerio* | AAI07956.1 |
|  | *Bos taurus* | CAH19227.1 |
|  | *Sus scrofa* | ABB92547.1 |
|  | *Ictalurus punctatus* | AEI59664.1 |
|  | *Takifugu rubripes* | AAW69373.1 |
|  | *Oncorhynchus mykiss* | NP_001118050.1 |
| TLR4 | *Homo sapiens* | AAC34135.1 |
|  | *Gallus gallus* | AJR32867.1 |
|  | *Mus musculus* | AAD29272.1 |
|  | *Labeo rohita* | AOM81178.1 |
|  | *Megalobrama amblycephala* | AMH41158.1 |
|  | *Ctenopharyngodon idella* | ACT68334.1 |
| TLR4a | *Danio rerio* | NP_001315534.1 |
| TLR5 | *Homo sapiens* | ACM69034.1 |
|  | *Rattus norvegicus* | ACN60145.1 |
|  | *Cirrhinus mrigala* | AHI59128.1 |
|  | *Larimichthys crocea* | KKF22099.1 |
|  | *Takifugu rubripes* | AAW69374.1 |
| TLR6 | *Homo sapiens* | ABY67133.1 |
|  | *Mus musculus* | AAG38563.1 |
|  | *Danio rerio* | NP_001124065.1 |
|  | *Chiloscyllium griseum* | CDO19210.1 |
| TLR7 | *Gallus gallus* | ACR26243.1 |
|  | *Homo sapiens* | AAF78035.1 |
|  | *Xenopus tropicalis* | NP_001120883.1 |
|  | *Danio rerio* | XP_003199309.2 |
|  | *Takifugu rubripes* | AAW69375.1 |
|  | *Salmo salar* | CCX35457.1 |
|  | *Coregonus maraena* | CEF90218.1 |
| TLR8 | *Homo sapiens* | AAF64061.1 |
|  | *Mus musculus* | AAK62677.1 |
|  | *Danio rerio* | XP_001920594.4 |
|  | *Epinephelus coioides* | AIS23538.1 |
|  | *Takifugu rubripes* | AAW69376.1 |
|  | *Miichthys miiuy* | ALJ55569.1 |
|  | *Scophthalmus maximus* | AQU15238.1 |
| TLR9 | *Homo sapiens* | NP_059138.1 |
|  | *Danio rerio* | NP_001124066.1 |
|  | *Mus musculus* | AAK28488.1 |
|  | *Takifugu rubripes* | AAW69377.1 |
|  | *Coregonus maraena* | CEF90220.1 |
|  | *Dicentrarchus labrax* | APM84342.1 |
| TLR10 | *Homo sapiens* | AAK26744.1 |
|  | *Rattus norvegicus* | ACN78428.1 |
| TLR11 | *Mus musculus* | AAS37672.1 |
|  | *Rattus norvegicus* | ACL80330.1 |
| TLR12 | *Mus musculus* | AAS37673.1 |
| TLR13 | *Mus musculus* | AAS37674.1 |
|  | *Salmo salar* | NP_001133860.1 |
|  | *Ictalurus_punctatus* | AHH40139.1 |
|  | *Scophthalmus_maximus* | AST16086.1 |
|  | *Miichthys_miiuy* | ALJ55571.1 |
| TLR14 | *Paralichthys olivaceus* | BAJ78225.1 |
|  | *Miichthys miiuy* | ALJ55572.1 |
|  | *Takifugu rubripes* | XP_003970412.2 |
| TLR15 | *Gallus gallus* | ABB71177.1 |
|  | *Coturnix coturnix* | ADL14379.1 |
| TLR16 | *Gallus gallus* | ABQ85926.1 |
| TLR18 | *Denio rerio* | NP_001082819.1 |
|  | *Cyprinus carpio* | ATW66458.1 |
|  | *Ctenopharyngodon idella* | AIB55030.1 |
|  | *Salmo salar* | CDK60413.1 |
|  | *Ictalurus punctatus* | AEI59674.1 |
| TLR19 | *Cyprinus carpio* | BAU98390.1 |
|  | *Salmo salar* | CDH93609.2 |
|  | *Danio rerio* | F1Q6F4 |
| TLR20 | *Ctenopharyngodon idella* | AHN49762.1 |
|  | *Cyprinus carpio* | AHH85805.1 |
| TLR21 | *Danio rerio* | AAI63075.1 |
|  | *Gallus gallus* | NP_001025729.1 |
|  | *Takifugu rubripes* | AAW69371.1 |
|  | *Coregonus maraena* | CEF90221.1 |
|  | *Epinephelus coioides* | ADM34974.2 |
| TLR22 | *Labeo rohita* | AGW43270.1 |
|  | *Epinephelus coioides* | AGA84053.1 |
|  | *Danio rerio* | NP_001122147.2 |
|  | *Takifugu rubripes* | AAW69372.1 |
|  | *Miichthys miiuy* | ALJ55574.1 |
|  | *Scophthalmus maximus* | AIC75881.1 |
|  | *Lates calcarifer* | AOV82293.1 |
|  | *Ictalurus punctatus* | AEI59679.1 |
|  | *Squaliobarbus curriculus* | ANT46160.1 |
|  | *Catla catla* | AGW43269.2 |
| TLR23 | *Takifugu_rubripes* | AAW70378.1 |
|  | *Miichthys miiuy* | ALJ55575.1 |
| BbtTLR1 | *Branchiostoma belcheri* | ABD58972.2 |
| Toll C | *Drosophila melanogaster* | NP_001262995.1 |
| Toll D | *Drosophila melanogaster* | NP_733166.1 |

**Supplementary Table 3.** **TLR ligands used in this study.**

| **Ligands** | **Working concentrations** | **Species** | **TLR agonist** |
| --- | --- | --- | --- |
| Pam3CSK4, synthetic tripalmitoylated lipopeptide | 1 µg/ml | Human | TLR1/2 |
| HKLM, heat-killed *Listeria monocytogenes* | 10^8^ cells/ml |  | TLR2 |
| Poly (I:C), synthetic analog of double-stranded RNA, 1.5-8 kb | 10 µg/ml |  | TLR3 |
| Poly (I:C) LMW, synthetic analog of double-stranded RNA, 0.2-1 kb | 10 µg/ml |  | TLR3 |
| LPS, Lipopolysaccharide from *E. coli* K12 | 10 µg/ml |  | TLR4 |
| Flagellin from *S. typhimurium* | 1 µg/ml |  | TLR5 |
| FSL-1, synthetic lipoprotein | 1 µg/ml |  | TLR6/2 |
| Imiquimod, imidazoquinoline amine analogue to guanosine | 1 µg/ml |  | TLR7 |
| ssRNA40, single-stranded RNA oligonucleotide | 1 µg/ml |  | TLR8 |
| ODN2006, synthetic oligonucleotides containing unmethylated CpG dinucleotides | 5 µM |  | TLR9 |
| ORN Sa19, *S. aureus* 23S rRNA derived oligoribonucleotide | 1 µg/ml | Mouse | TLR13 |

**Supplementary Table 4. TLRs in *B. lanceolatum***

| **Gene ID in database** | **LRRs** | **sccTLR/mccTLR** | **Domain structure** | **First hit of annotated sequence by blastp in NCBI** | |
| --- | --- | --- | --- | --- | --- |
| BlTLR22 | 21 | sccTLR | LRR+TM+TIR | TLR22 *Siniperca chuatsi* |  |
| Bl10262 | 8 | sccTLR | LRR+TM+TIR | TLR13 *Saccoglossus kowalevskii* |  |
| Bl09440 | 16 | sccTLR | LRR+TM+TIR | TLR3 *Saccoglossus kowalevskii* |  |
| Bl22164 | 14 | sccTLR | LRR+TM+TIR | TLR13 *Saccoglossus kowalevskii* |  |
| Bl05337 | 5 | mccTLR | LRR+TM+TIR | TLR *Portunus trituberculatus* |  |
| Bl07821 | 9 | mccTLR | LRR+TM+TIR | TLR *Portunus trituberculatus* |  |
| Bl52875d | 16 | sccTLR | LRR+TM+TIR | TLR3 *Chelonia mydas* |  |
| Bl52875c | 15 | sccTLR | LRR+TM+TIR | TLR3 *Saccoglossus kowalevskii* |  |
| Bl52875b | 19 | sccTLR | LRR+TM+TIR | TLR3 *Chelonia mydas* |  |
| Bl52875a | 4 | sccTLR | LRR+TM+TIR | TLR3 *Saccoglossus kowalevskii* |  |
| Bl72403 | 20 | sccTLR | LRR+TM+TIR | TLR3 *Chiloscyllium griseum* |  |
| Bl19440 | 20 | sccTLR | LRR+TM+TIR | TLR22 *Danio rerio* |  |
| Bl21810 | 7 | sccTLR | LRR+TM+TIR | TLR13 *Rhincodon typus* |  |
| Bl12652 | 18 | sccTLR | LRR+TM+TIR | TLR22 *Siniperca chuatsi* |  |
| Bl56664 | 13 | mccTLR | LRR+TM+TIR | TLR *Portunus trituberculatus* |  |
| Bl20861 | 17 | sccTLR | LRR+TM+TIR | TLR13 *Poecilia formosa* |  |
| Bl48785 | 14 | mccTLR | LRR+TM+TIR | TLR *Biomphalaria glabrata* |  |
| Bl24356 | 15 | mccTLR | LRR+TM+TIR | TLR *Portunus trituberculatus* |  |
| Bl18798a | 18 | sccTLR | LRR+TM+TIR | TLR22 *Epinephelus coioides* |  |
| Bl18798b | 20 | sccTLR | LRR+TM+TIR | TLR3 *Paralichthys olivaceus* |  |
| Bl24343 | 16 | mccTLR | LRR+TM+TIR | TLR *Portunus trituberculatus* |  |
| Bl19922 | 10 | sccTLR | LRR+TM+TIR | TLR13 *Xiphophorus maculatus* |  |
| Bl17405 | 15 | sccTLR | LRR+TM+TIR | TLR22 *Siniperca chuatsi* |  |
| Bl04519a | 15 | sccTLR | LRR+TM+TIR | TLR22 T*akifugu rubripes* |  |
| Bl04519b | 13 | sccTLR | LRR+TM+TIR | TLR13 *Astyanax mexicanus* |  |
| Bl08928a | 6 | sccTLR | LRR+TM+TIR | TLR13 *Astyanax mexicanus* |  |
| Bl08928b | 17 | sccTLR | LRR+TM+TIR | TLR9 *Poecilia mexicana* |  |
| Bl08928c | 15 | sccTLR | LRR+TM+TIR | TLR9 *Poecilia mexicana* |  |
| Bl75614 | 16 | sccTLR | LRR+TM+TIR | TLR13 isoform X2 *Xiphophorus maculatus* |  |
| Bl30396 | 17 | sccTLR | LRR+TM+TIR | TLR13 *Poecilia mexicana* |  |

**Supplementary Table 5.** **TLRs in *B. floridae***

| **Gene ID in database** | **LRRs** | **sccTLR/ mccTLR** | **Domain structure** | **First hit of annotated sequence by blastp in NCBI** |
| --- | --- | --- | --- | --- |
| Bf68489 | 19 | sccTLR | LRR+TM+TIR | TLR 22 *Seriola lalandi* |
| Bf88412 | 13 | sccTLR | LRR+TM+TIR | TLR 22 *Epinephelus coioides* |
| Bf89467 | 25 | sccTLR | LRR+TM+TIR | TLR 22 *Megalobrama amblycephala* |
| Bf89468 | 14 | sccTLR | LRR+TM+TIR | TLR 22 *Megalobrama amblycephala* |
| Bf89511a | 6 | sccTLR | LRR+TM+TIR | TLR 1 *Branchiostoma belcheri* |
| Bf89511b | 5 | sccTLR | LRR+TM+TIR | TLR 22 *Megalobrama amblycephala* |
| Bf89513 | 14 | sccTLR | LRR+TM+TIR | TLR 22 *Danio rerio* |
| Bf89514 | 4 | sccTLR | LRR+TM+TIR | TLR 22 *Danio rerio* |
| Bf92915 | 3 | mccTLR | LRR+TM+TIR | TLR *Portunus trituberculatus* |
| Bf94576 | 16 | sccTLR | LRR+TM+TIR | TLR 13 *Salmo salar* |
| Bf97448 | 16 | sccTLR | LRR+TM+TIR | TLR 3 *Anas platyrhynchos* |
| Bf105256 | 18 | sccTLR | LRR+TM+TIR | TLR 3 *Anas platyrhynchos* |
| Bf126412 | 16 | sccTLR | LRR+TM+TIR | TLR 22 *Danio rerio* |
| Bf100709 | 12 | sccTLR | LRR+TM+TIR | TLR 21 *Lissotriton montandoni* |
| Bf213613 | 13 | sccTLR | LRR+TM+TIR | TLR 3 *Dicentrarchus labrax* |
| Bf236291 | 14 | sccTLR | LRR+TM+TIR | TLR 13 *Tupaia chinensis* |
| Bf73275 | 5 | mccTLR | LRR+TM+TIR | Toll *Apostichopus japonicus* |
| Bf82252 | 12 | sccTLR | LRR+TM+TIR | TLR 22 *Ctenopharyngodon idella* |
| Bf82677 | 12 | sccTLR | LRR+TM+TIR | TLR 21 *Miichthys miiuy* |
| Bf85671 | 7 | mccTLR | LRR+TM+TIR | TLR *Portunus trituberculatus* |
| Bf88496 | 16 | sccTLR | LRR+TM+TIR | TLR 3 *Haliaeetus albicilla* |
| Bf99056 | 7 | sccTLR | LRR+TM+TIR | TLR 4 *Leopoldamys sabanus* |

**Supplementary Table 6. TLRs in *B. belcheri***

| **Gene ID in database** | **LRRs** | **sccTLR/mccTLR** | **Domain structure** | **First hit of annotated sequence by blastp in NCBI** |
| --- | --- | --- | --- | --- |
| Bb304600F | 14 | sccTLR | LRR+TM+TIR | TLR22 *Danio rerio* |
| Bb294010R | 4 | sccTLR | LRR+TM+TIR | TLRII *Oncorhynchus mykiss* |
| Bb115530R | 2 | sccTLR | LRR+TM+TIR | TLR13 *Larimichthys crocea* |
| Bb308500F | 4 | sccTLR | LRR+TM+TIR | TLR22 *Ictalurus punctatus* |
| Bb008380R | 2 | sccTLR | LRR+TM+TIR | TLR22 *Megalobrama amblycephala* |
| Bb292100F | 9 | sccTLR | LRR+TM+TIR | TLR22 *Danio rerio* |
| Bb020090R | 5 | sccTLR | LRR+TM+TIR | TLR22 *Scleropages formosus* |
| Bb024630R | 7 | sccTLR | LRR+TM+TIR | TLR13 *Myotis brandtii* |
| Bb320220F | 5 | sccTLR | LRR+TM+TIR | TLR22 *Ictalurus punctatus* |
| Bb112940R | 4 | sccTLR | LRR+TM+TIR | TLR22 *Ictalurus punctatus* |
| Bb207490F | 7 | sccTLR | LRR+TM+TIR | TLR22 *Scleropages formosus* |
| Bb020250R | 7 | sccTLR | LRR+TM+TIR | TLR22 *Scleropages formosus* |
| Bb020150F | 4 | sccTLR | LRR+TM+TIR | TLR22 *C**yprinus carpio* |
| Bb088530F | 1 | sccTLR | LRR+TM+TIR | TLR22 *Cyprinus carpio* |
| Bb091830F | 4 | sccTLR | LRR+TM+TIR | TLR22 *Cyprinus carpio* |
| Bb020140F | 7 | sccTLR | LRR+TM+TIR | TLR22 *Danio rerio* |
| Bb112920R | 7 | sccTLR | LRR+TM+TIR | TLR22 *Scleropages formosus* |
| Bb010410F_t1 | 6 | sccTLR | LRR+TM+TIR | TLR22 *Ictalurus punctatus* |
| Bb010410F_t2 | 7 | sccTLR | LRR+TM+TIR | TLR22 *Ictalurus. punctatus* |
| Bb032660R | 5 | sccTLR | LRR+TM+TIR | TLR22 *Megalobrama amblycephala* |
| Bb134830R | 4 | sccTLR | LRR+TM+TIR | TLR22A *Lissotriton montandoni* |
| Bb300960R | 5 | sccTLR | LRR+TM+TIR | TLR22 *Megalobrama amblycephala* |
| Bb044020R | 16 | sccTLR | LRR+TM+TIR | TLR3 *Chiloscyllium griseum* |
| Bb202930F | 21 | sccTLR | LRR+TM+TIR | TLR22 *Scophthalmus maximus* |
| Bb122860F | 2 | sccTLR | LRR+TM+TIR | TLR21 *Epinephelus lanceolatus* |
| Bb112820F | 6 | sccTLR | LRR+TM+TIR | TLR22 *Scleropages formosus* |
| Bb210680R | 8 | sccTLR | LRR+TM+TIR | TLR22d *Gadus morhua* |
| Bb205510F | 4 | sccTLR | LRR+TM+TIR | TLR *Carassius auratus* |
| Bb131620F | 13 | sccTLR | LRR+TM+TIR | TLR13 *Myotis davidii* |
| Bb210690F_a | 9 | sccTLR | LRR+TM+TIR | TLR22 *Siniperca chuatsi* |
| Bb210690F_b | 1 | sccTLR | LRR+TM+TIR | TLR13 *Rhincodon typus* |
| Bb267200F | 4 | sccTLR | LRR+TM+TIR | TLR13 *Larimichthys crocea* |
| Bb025770R | 6 | mccTLR | LRR+TM+TIR | TLR *Portunus trituberculatus* |
| Bb025760F | 6 | mccTLR | LRR+TM+TIR | TLR *Portunus trituberculatus* |
| Bb041610R | 5 | mccTLR | LRR+TM+TIR | TLR *Portunus trituberculatus* |
| Bb113680F | 1 | mccTLR | LRR+TM+TIR | TLR *Anthopleura buddemeieri* |
| Bb081670R | 6 | mccTLR | LRR+TM+TIR | TLR *Biomphalaria* *glabrata* |

**Supplementary Table 7. Protein sequence identity of BlTLR and fish TLR22.**

| **Species** | **Accession number** | **Identity (%)** |
| --- | --- | --- |
| *Branchiostoma lanceolatum* | MG437061 | 100.0 |
| *Oncorhynchus mykiss* | NP_001117884.1 | 29.7 |
| *Takifugu rubripes* | AAW69372.1 | 27.8 |
| *Miichthys miiuy* | ALJ55574.1 | 30.6 |
| *Epinephelus coioides* | AGA84053.1 | 30.7 |
| *Scophthalmus maximus* | AIC75881.1 | 30.8 |
| *Lates calcarifer* | AOV82293.1 | 30.3 |
| *Ictalurus punctatus* | AEI59679.1 | 29.7 |
| *Carassius auratus* | AQX43082.1 | 30.2 |
| *Squaliobarbus curriculus* | ANT46160.1 | 28.9 |
| *Catla catla* | AGW43269.2 | 29.4 |
| *Labeo rohita* | AGW43270.1 | 29.2 |
| *Danio rerio* | NP_001122147.2 | 29.7 |

**Supplementary Table 8. Ectodomain architecture of vertebrate TLRs and BlTLR.**

| **Family name** | **TLRs** | **LRRs** | **Architecture** | **Ligands** |
| --- | --- | --- | --- | --- |
| TLR1 Family | TLR1, 2, 6, 10, 14, 18, 24, 25 | 19 | Three-domain | Hydrophobic: lipids and lipoprotein |
|  | TLR15 | 19 | Single-domain | Virulence-associated fungal and bacterial proteases |
| TLR3 family | TLR3 | 23 | Single-domain | dsRNA |
| TLR4 family | TLR4 | 21 | Three-domain | LPS |
| TLR5 family | TLR5 | 21 | Single-domain | Bacterial Flagellin |
| TLR7 family | TLR7, 8, 9 | 25 | Single-domain | ssRNA, CpG-DNA |
| TLR11 family | TLR11, 12, 16, 19, 20, 26 (TLR11 subfamily) | 23, 24 | Trans-three-domain | Profilin from *T. gondii* |
|  | TLR13, 21, 22, 23 (TLR13 subfamily) | 25 | Single-domain | ssRNA, CpG-DNA, dsRNA |
|  | BlTLR | 21 | Single-domain | dsRNA |
