# Supplementary material for: Characterization of the TLR Family in Branchiostoma lanceolatum and Discovery of a Novel TLR22-Like Involved in dsRNA Recognition in Amphioxus
Source: Front Immunol. 2018 Nov 2;9:2525. doi: 10.3389/fimmu.2018.02525 (PMC6224433; doi:10.3389/fimmu.2018.02525)
Supplement: Supplementary Data 1 — TLR sequences of L. variegatus and S. kowalevskii used in the phylogenetic analysis. The TIR domain of each TLR is highlighted in yellow. [file Data_Sheet_1.PDF]

Supplementary Data 1

Characterization of the TLR family in *Branchiostoma lanceolatum* and discovery of a novel TLR22-like involved in dsRNA recognition in amphioxus

Jie Ji[1], David Ramos-Vicente[2,3], Enrique Navas-Pérez[4], Carlos Herrera-Úbeda[4], José Miguel Lizcano[5], Jordi Garcia-Fernàndez[4], Hector Escrivà[6], Àlex Bayés[2,3] and Nerea Roher[1*]

*** Correspondence:** Corresponding Author: nerea.roher@uab.cat.

Lytechinus variegatus TLRs

>LVA_016441_RA
MYDVSRRNGSLNWSSVSASGCQLTYTSQGVNASCTNLGLLSVPEGLPHNTIVLDLSRNVIETLDNKS
FTYLPDIIKLDLSVNKITLIEGGTFTPLGSLTELDLGRNQIKSLPAGLTSLKSLDLSDNYISSISGD
IFPRQSQLSVLNISNNKLDIIPKILFNRHPYLQSLFIQHNKISSIEHGTIIPKRFTIDVSDNPLACT
CDLRWFVDWLGSSNIEFHHPNHTICSHSSIKDMVELPILSFDPDKYCGINIILITSLSFAALLVVAL
SLLAYWKRWWFNYKVFLLRLAICGYKEMVQDFEDQDYEFQLNLMYQEEDQEWVDNVMKPVLQERFPH
LDRVVFGDNGLHLGMFYINALHYAVENSFKTALLLSNNAVHEAWFITKVRIALEEVNDSRLDKVILF
FLEEIDDDDLPYLVRLFLSKVKPYMLWTDDEDGQELFWAHFEKSMRGNRELNSIIPV

>LVA_011338_RA_Scaffold1242_prot
LDPETRGGLALIGQGHKECTSYTGNNNYVIIGGEMIDPLMSLLFITAKEKQRQAVIYLKRIALIMTL
KRGRILLHFAITVNQIAAITFYPKKTFLDHTTRRCGEDLIEKNADCSGLGFEYVPQHLPGDLQGLNL
ERNNITSLPNSSFTRYPLITELNLSKNDIRIIESTAFQPLKFLGKLSISSNLNLVFAGKGLLKWARK
LLYLDLSESRMSSFPNDILAWSANVDTLNLKYNLLKVINISSCGRVKSVDLSYNKIERLTKDVFHFP
CISGTLDLSSNLIMSVDSDVVKSLHVKSLVLKFNIPPMAIPTAKEMFIGISRSKINTFIYSYSADLT
TIPLDLFDPLRDSSLTTLDLSMNAFRYPYAFIFSNLTSLKRFKLTLNQFLSVEPIFFEGMHNLRVLN
VALNSLKYINSLEIPWKLDLTQLDVSSNHVTEITNNTFIGLHKLQILKLNSNPISRMHAESFLALES
IETIDLAYTRIVIFRMVIPNLISLHLTDMTMEYDPTECLKDVPSLQNLQLSNSALGIRNLWNSDKNI
SLFDRVTNLTSLDLNRNYFSVKYNVEKILPPGILQPLSALRNLSLQKCELSNLHPEVFIGLKSLEVL
LLSENNLEQLPTILFKKLNQITKIDLSDNILTELDRDIFVSNRRLETLLISDNKLTRVDQNTFKPIH
SSLFLIDLSNNPIDCNCDLKLDRDIFVSNRRLETLLISDNKLTRVDQNTFKPIHSSLFLIDLSNNPI
DCNCDLSWLIDFLDKSLSLENGNMTRCHAASLEPLRGKLLIHFDPEEYCSMNSTIVCLPPLAIICLI
FIIALVYYNRWQLRYKVFLIKLAVIGYKEMRDARDHNDYEYDLNVIFYDDDEDWIREHLRPAIAERL
PQFERNVFGDDELILGMHYLDSVDYVVSHSYKTVVILSRAAVRDRWFILKFRTAMDHVSDTQTEFVV
VVFLEDIPDNEMPFLVRLYLSDGRPYIHWTEDIRGQDYFFEELTKHLTINLRTDDRIPIE

>Scaffold1434_prot
NTTELTRKQFNKNTLIYFSWFRELDLLRHEFNKELSFLELNAFSGLSSIKYIDLSGTNVYDLQLNIP
TLRILSLNNILEPPNWTPSRSPFRYLQSLVDFSLKKTSISLVHLFSNATNASLFDGLLNLHCLDLSG
NDFGGTIFFDNTLPPGIFRQLFALQELIIDDSKIEKVHPHFFTGLKSLQTLSLKGNNIKSLDERLLW
ILPQLGSINLEGNQISYLERYIFLNNAKLTNLSLADNKLTSLNQSTFKPIASSISLLDLSNNPIACN
CDIQWLIDLLNENIDLDNKDNTICTIASIEPLRLKHLLDFDPNQFCTGNIGLISLISLTIVCFIVIS
IITYHNRWYLKYKIFLLKLAAVGYREMQDAREHDDYEFDLNIIFYDDDEVWVREHFRPALVEHLPQF
RRNVFGDEDLVLGMHYLDAVDYVVTHSYKTIIVLSKAAVKDRWFVLKFRTAMDHVSDTLTEFVMVVF
LEDIHDDEMPFLVRLYLRDGRPYIHWTEDVRGQEYFWNKLTKYLTINLRTNDILPNE

>LVA_002063_RA
MEVNHILHFLGFSSLFVRHVDAHVGFEATTSSTLLTSKSTHGCNQDMDFRMVDCSHRDLKEVPQDLS
KDIVLLDLSNNRIKMLLNSSFDVYPLITSLYISNNDLGVIESTSFHPLHGLKILNLSCNPRLVLPVK
DVFKMSPQLITLYLEEANLMTLPNDTLQSNQNLHRLLLSKNKLSFLNISSCGKVDKVYMDGNQIQRI
AKGWFTFVCHSDFLDLTDNPIQFVDPDDFASLNIRSLKLGFYPLSEEVLINTTLGISGAFPEGLFDP

LSAVGYREMQDAREHDDYEFDLNIIFYDDDEVWVREHFRPALVEHLPQFRRNVFGDEDLVLGMHYLD
AVDYVVTHSYKTIIVLSKAAVKDRWFVLKFRTAMDHVSDTLTEFVMVVFLEDIHDDEMPFLVRLYLR
DGRPYIHWTEDVRGQEYFWNKLTKYLTINLRTNDILPNE

>LVA_027441_RA_Scaffold15379_prot
MDIKHPLLIWHIVMTLGQSFQSVATIVPSHSHLNDKSPRCYIDLKAKHAKCTRLGLNTVPQYLPGEI
EGLNLQRNNITSLLNSSFQRYPHIAYLDLSFNYIRVIESSAFHPLKGLIKLALSWNSNLVVQDAGLF
KWSRRLSYLTFLNSNLRSIPDDIIAWSRNMDKLELRGNLLTTVNITSCGRAKTVQLSQNNIASITKD
AFNFFCNCDTLYLERNPIISVDPNLISSLLIRSLVLGHRELNIQALKNLSVGISRSAISSFSFCGAD
LSLIPMDLFDPLRNSSLRALNLSNNNIHILHRSIFSNLTTISHFNLNRNSIVKVEPFFFEGMDELRV
LDLRLCEIEDINTYNLTWTIDLTELDLSSNLLTEINHNTFRGDVFLNNKMLRILTLSDNKLARLDRG
TFQPIYSSLLSIDISLNPIECNCDLGWFLDWLSKSLSLINWQKTHCRETSLKPLWDQYLTDFDHKAY
CHVNIVQVCLPSFAIICLIFIIALVYPNRWLLKHKVFLLKLAAIGYNEVRDARDHKDYEYDLNVIFY
DDDENWIRENFRPAVVERLPQFQRNVFGDADLVPGMYYLDAVDYVVSHSYKTVIVLSRVAIRDRWFI
LKFRTAMDHVSDTGTEFVVVIFLEDIPEDEMPFLMRLYLSDGRPYIYWTENVRGREYFFDELTKQLT
VNLRTNDRIPYD

>LVA_027180_RA_Scaffold19048_prot
MANGSRIYFLLLACLPVTLSLQLSNVHDRHLPTRSPTGSSKEHGCDQSFTLKTASCRNRGLNSVPQN
LTQDIEVLDLGHNNITRLLNVSFELYPLINNLDISSNNIRIIQSAAFYPLKDLTSLYLDYNPDLVLP
ETGTSSLDLKGNPIQTVDPDVISSLHVRSLEHGDYPLSDDVVTNCILGVSKSSIKQLKILRGSLGAF
PVGFFDPLLDYPMYVLDLGKNHLTGLHPMVFSNLTQLMELRIYENALSIPEIQPDFFTGMKALTVLV
LPENQIRQINPHNQTWSLKLSEIYLMGNLISEISAYTFRGLKNLTTLDMSSNKYLSVFELTDESGLD
SIHTVSLSESRISVLALKTPSLKSLSMNYIRYDFPPLRPGESFQHLQSLNKLNMADSAINQINIWDA
TTNTSLFDGLLNLNYLILSNNPFLSLVDIPPGVFQRLSALRELSLANCFIKTLHPLVFSGLGSLQKL
DLHGNNIQHLNGGVLYMLEKLESIDLYGNQISYIEEVTFSNNGKLHSLSLANNRLTRLNQSTFKPLL
SSISSLDLSKNPINCNCDLKWLVDLVNKRLHLINKDKTICSSASLAHLREKNLLDFDADKLCTINIG
IISIIPLAVVCFSVVTILVCRNKWQLKYKIFLLRLAVLGYKEKQDAREHDDYDYDMNVIFYDGDEEW
IQDHLRPALEEHLPQFQRNLFGDEDLVPGMHYLDSVDYVVTHSYKTVIVLSRAAVRDHWFILKFRTA
MDHVSHTQTEFVLVVFLEDIPDDELPFLVRLYLSDGRPYIHWTEDERGQGYFWNELSKNLTVNLRMN
DMIPNE

>Scaffold7016_prot
WPDLVYSLGSPCFHYHHPLGLSQQMAVEVQNLSLKLAALGYKEMHDARDHNDYVFDINIIFFDGDEE
WIREHLRPALELHLPWYQRNVFGDEDLLLGMHYLDSVDYVVTHSYKTRCQNNNNNNQMPKYIKDSLS
SHRPTRPNLRSSKDPYLLAIPKTRTKSGDHTFSVTASKEWNIIVLSRAAVREHWFILKFRTAMDHVS
DTLTEFVLVVFLEDIPDDEMPFLVRLYLSDGRPYIHWTEDLRGQEFFWNELTTNLTVNLRTNDLIPN
E

>Scaffold7830_prot
KLAHELVKQITYPPEFQKTICASDSLEPLQNQHLINFDPKDFCSIDIVPILLPSLAIICLILIVGIT
YHNRWQLRYKLFLVKLAAIGYKEMRDARDHNDYEFDLNVIFYDDDEDWIREHLQPAMAERLPQFQRN
VFGDDELVLGMYYLDAVDYVVSHSYKTVVILSRAAVRDRWFILKFRTAMDHVSDTGTEFVVVIFRED
ISDDEMPFLVRLFLSDGRPYIYWTDDVRGQEYFFKELTKHLTINLRTNDRLPVE

>LVA_028064_RA_Scaffold7839_prot
MERCLIYSLMVVLITVQCSFGYDRDPVKISSFPMKGSLQGCNENLQTKTVACIGKGLKRVPRNLPED
IYVLNLAYNNITMLLNASFEEYPLIKDLYIYFNDLRSIEPAAFHPIRDLRYLDLSYNTRLVVLPVKG
VFMKSPKLFSLNLSRLNLRSIPNDTLKWSPRLRLVNLSSNKLSFINLSSCGTVKEVNLSRNLIQRLT
MEYFIFPCRTDHLDLTNNPIQFVDSDDIASLQVRSLKLGQYNLRNETLLNIILGISKSNIQELSISN
GFVGEFPEGLFEPLRNSSLSVLKFNGNSIISHDLSVFSNVTKIEQFDFSYNIPFIDEIQPVFFDGMR
ALKVLTIDGNEVTKLNPFNQNWTVNLTHLSLQDNKLRAIFAPAFSGLESLISLDMAWNKYLFVFMAT
SGLDNIQYLDLRGSSITFLEFNTPTLRFLFLNSVLDHPHLAPGDSFKHLQSLVELNMRDSRIYDSNL
WGGVDESLFDGLLNLTYLDLSRNDFKYDLTPGIFRELFGLIELNLEECSMTGIHPLLFVGLESLQKV
NLKGNSIKHLRGDLLSVLGQIIDINLEGNLINFLDDVMFINNKKLTNLSLANNKLTRLNQSTFKPIF
SSISTIDISMNPIVCNCDLKWLIDWLKGSIELNHINNTVCSPASVKPVRLKTLFHFHPDELCSIRTG
LVSVIPLAVVGVLVLVVVVFHNRWHLKYKIFLLKLAIFGYKEMQDARDHNDYEFDMNIMFYDDDEEW

IRDHLRPALEEHLPQFQRNVFGDEGLVLGMHYLDAVDHAVTHSYKTIIVLSRAAVRDHWFILKFRTA
MDHVSDTLTEFVLVIFLEDIQDDEMPFLVRLYLSDGRPYINWTDDVRGHEYFWNELSKRLTINLRTN
DNIPNE

>LVA_017961_RA_Scaffold8340_prot
MAVYNLLYECYVPFLAQIDLEERSYHHGNFNCVIHPLLLVIITSSLQSVHGIVRNSPTVFTEKPLHG
CDYDMVKKEVSCNYKQLDDVPQNLPRDTKYLDLSYNQITILLNSSFNVYPLISHLIIVQNDVRSIDS
AALYPLKFLRYLDLSKNFRLLLPVTDIFLVSSKLSHLDLIGSNLTSFPNNMLKSTTHLRLLRLTANY
LSFINVSSCSKVDAVYLSNNRIQRLTAEDFIFPCQTDSLSLVGNPIQFVESTVIASLKVHSLRLGGY
PLSNEVLSNIILGISMSKNIKELGIFGGSIGTFPKYLFDSLCNYSLSALTFKENHLKNLHPFVFSNL
TTLKQLIIIDNYVSIDNIQPNFFDGMHALEVLNMSNNGILQIYPSNQNWTLNVTHINLRKNALRTLF
VSAFHGLRNLILLDLRENNLVELTLTAFSRLRSLYLNNIENPYGVLRPGESFLTLKSLVDLRLRHGY
INGAILFNTEEKVSLFDGLSNLRFLDLSENQPLGIVLPHDVFKQLSALQELTLDDCHIRHINPLLFL
GLRSLQKLSLKENRITQLSYEFMFLFHLTDINLNGNVISYLGPSTFSTNKKLSIISLAHNQLTNLDQ
RTIKPIIFSIVSLDLSNNPIICDCDLNWLVGLLNKNLHLAQEDNTHCSQASLKPFRLKPLTDFDPND
ICTNINVFSLLAPLSVCCLLFIILVVFRNRWHLKYKIFLLKLAVFGYKEIRDARDHNDFEYDINIMF
YDDDEEWIREHLRPALEENLPQFQRNVFGDEDLVLGMHYLDAVDHAVTHSYKTIIVLSRAAVRDHWF
ILKFRTAMDHVSDTLTEFVLVIFLEDIPDDEVPFLVRLYLSDGRPYINWTDDVRGHEYFWNELSKRL
TINLRTNDMIPNE

>Scaffold9304_prot_a
FIMAHFSLLLHVSIILIQYSQSLEAQVNLGPRSCHEDLKEKTAMCGRMGFKSVPQHLPDGIEVLQLE
YNNISSLLNSSFTRYPLIAKLDLSTNDLRVIETAAFHPLKNLTRLILCGNLNLLIQGTGIFKYTKKI
SYFDMSDSLMGSIPYDMLAWSQNVDTLNLESNLLTSINMSLCGRAKTVDLTDNKIIRLTSNTFNFSC
NTDTLDLSWNPIRSVDPNVIASLHVKSLRFQTDVITTQTLSSIFLGISNSSTLSIKSLQISRGTKIN
FSSPGLFDPLRDGSLETLDLSQNNIRSLHPYIFSNLTSVRKFILTYNDIIAVQPEFFFGMQMLRVLE
MKYTQLKHIDNQFIPRPWSLGLSELCLSNNELTIIYRDMFTWLQNLTNLNLSSNKLLAFIEEDSFID
LKTIEVIDLSECKIMKFHSIVVPSLKSLYLNNNIVWLFTLGPTRPFKRLHLLEHLEMVNAEYFDNDL
WDPGMNTSLFDGLFNLKTLNLSKSQLSNWHDGDNLPPEVLRQLSVLQNLSLEDCDLSDLHPGAFSGL
KSLQVLILRNNNLEKLSAVLFKQLLNITIIDLSDNLLMDLDRGIFSSNRELKTLLLSNNKLTHLDQS
AFKPIQTSLLSIDISTNPINCNCDISWLMNWLSRSLILQNSNKTICASDSLVPLQNQHLINFDPKEY
CSIDIVLICLPSLAIICLILIIGLTYHNRWQLRYKLFLVKLAAIGYKEMRDARDHNDYEFDLNVIFY
DDDEDWIREHLRPALAERLPRFQRNVFGDDELVLGMYYLDAVDYVVSHSYKTVVILSRAAVRDRWFI
LKFRTAMDHVSDTGTEFVVVIFHEDIPDDEMPFLVRLFLSDGRPYIYWTDDVRGHEYFFEELTKHLT
INLRTNDRLPV

>Scaffold9304_prot_b
HIPRNFKFVNNSLEIYRGTLNLSSPGLFDPLHNCSLEILKLSENSVHGLSPYTFSNLTKLRKFIFTY
NNIDYIKPIFFFGMQNLRVLDVTRNVIQHIGKYPKQCSWTLELNELYLSNNHLFGIDKYTFWGLHNL
TNLYLSSNKWLNYIAEESFVDLQSIEVIDLSECNIVDFRNIVIPGLKSLYLNDMISLFGLQLRYPFK
RIQHLENLEMMNAGIYYYDLWNPVSNASLFGGLSNLRTLNLSKNRLSNTEGGDNLPPGVLQQLSALE
NLNLGNCDLSDIHPGAFTGLKSLQVLILRSNKFEQLPVILFQKLIQITTIDLNDNFLTAFERDHFLS
NRQLKVLLLSNNKLTRFDQSAFKPIYSSLVSIDVSMNPISCNCDLSWLLEWLSRSLILQNSNKTICA
SDSLEPLRNQHLIKFDPKDFCSIDIVPILLPSLAIIRLILIVGLTYHNRWQLRYKLFLVKLVAIGYK
EMRDARDHNDYEFDLNVIFYDDDEDWIREHLRPALAEQLPQFQRNVFGDDELVLGMYYLDAVDYVVS
HSYKTVVILSRAAVRDRWFILKFRTAMDHVSDTGTEFVVVIFREDIPDDEMPFLVRLFLSDGRPYIY
WTDDVRGHEYFFEELTKHLTINLLTNDRLPV

>LVA_016920_RA_Scaffold8843_prot
MAFKHLTSFPSKSVLACFIFPLVFLSKLMLSSGLLTGNAGHGCHQDFELRKAHCSNKGLDSIPQNLA
EDIEELDLSENNITVLENISFERYTQISTLDLSANDIRVIEPTAFWPLKNLTTLLMILNSNLVLPET
GLFRWTNEMTFLDLTGSNLKSIPNDILKWNKHLGRLDLGYNHLTFINISTCGSLEYAGLYDNHFEYI
TRESFMFSCQTDTLDLTGNPIKLIDPVAVAALHTRSLLIGIAPESQSWTPEMLINLTIGISLSTTNE
LHIRDVGLEFVPKGVFSSFQQVSFNLLDISYNKLNTLYPFVFSNLTLLSKLVIENNKLTSIEPSFFD
GMQELRVLRLNFNSIRTINPNNQTWTINLVELYLSTNSLTEISEFAFLGLQNLTIIDFRWNSQLAVL
HMTSFTGVDNIQVIDLSDCSITRLELYTPALVSFRMNRNSRGIFPLSPLEPGESFENAKNLVWLEIQ
DSEIWSSNLWHSFRGVSLFGGLSNLTTLDMSNNPDIGKSDLAPGTFLQLSALQELGLSGCGISNLNP
LLFSDLVSLKKLDLSGNNLMQIRSGLFAKLEQVQSIKLDGNILAHLDEKAFSNNPNLAALSLARNKL

TGLNESTFNPVFSTLSVFDLSQNPLVCTCDLEWLLDWLNGTGNLTLVNKEQTICAPPSLMSLRERPV
LDFNAADICQLNYAIFSSIPIVIASVIAITVFVYHKRWQLKYKLFLLKLAIVGYRERLDARDHMEYE
FDVNIIFYDDDDDEGWIRENLKPTMEEQLPRFQRNVFGDADLILGMHYLDAVDYVVTRSYKTIIILS
RAAVRDRWFMLKFRTAMDHVSDTQTEFVVVVFLEDIPDDELPFLVRLYLNDGRPYLHWTPDVRGQEY
FWNELAKNLTINLLTNDLIPNE

>LVA_000678_RA_Scaffold356_prot
MALDRPVLLVAINLLIVLLLQQTPYANGNALPYKNKEGSSPTCPFDVPSKTADCKQLGLTNVPQDLP
DGVEILLLSRNSITELFNSSFERYIHITKLDLYKNAISKVHPSTFHPLRLLTELQISNNKGLILQHE
GLLRWATELTAIDVSHSGLKSLPDDFLKWSSKLKYVNFYGNHLRSINISLSGKIDVFDLSGNQMYQL
NNNTFQITCEIDILKLEENRIISVDPDVIASLQIRSLHIGKSRISTQTLKYIFLGVSRSKMKRLFVI
DLLHRWTPSYLFDSLRNSTISELILFDNSIKLHPDIFFSLTNITFFDFTFNTNRNIEPSFFHGMGKL
MYLYLRNNEIQTINLKNEIWQNDLIEELDLSYNDIIEIQPSTFQGLQNLRYLNLDANYGLVSLEPTS
FVEMKRLETVILTDTNILKLHLVTPYLRNLLLNKMRSFRPGFSFNQSSFLVNLEIYDSLLSVDDLWD
PMTNVSLFDGLFNLTFLKLEISVKVQDPLRNKIFRSLSNLQNLTFKSVGMSNIPSKAFAGLESLKNL
DLSRNYIAKLHPDSLNGLSKLESVDLSENRLLYLDSDVFSNNQNISRLILSRNQLTFFNQNTFYPIR
SSLLYIDLSWNQIACDCELSWLVRRLILSRNQLTFFNQNTFYPIRSSLLYIDLSWNQIACDCELSWL
VRWVISTPTLKLLQGDNTVCGIASLEPFRDKPFLDFNPTKLCGFNVNLVCLPLIVVISLVATIILVY
NFRWQLRRWLFLLKLAALGYIEARDPREHDHYEYDLNIIFHEDDEAWVREHLRVVIEERLPQFQRKV
YGDGDLVPGMYYLEAVDYAVTRSYKTVVVLSRAAIKDRWFMLKFRTAIDHVTDTQTEFVLLLFLEDI
PGEELPFLVRLCLSDGRPYVHWPEDIRGRDYFCDELIAKLTINLKTNDLIPIE

>LVA_000679_RA_Scaffold356_prot
MLRVAINLSIVMLLQQAPYADSNALPYKEKDGSAHTCDFDVPSKTADCTELGLTTVPQDLPDGLNDA
TFHLTCEIDILKLDDNHIISVDHDVIASLQVRSFKIGGPRFQSKTLKNIFIGNLTIRMASISSIHSN
AFTGLKSLKFLDLSGNYLTKIHPDSLKGLWTLETVDLFENSLVYLDSGVFSNNQNLSRLILSQNRLI
FFNQSTFDPVISSLLYVDISQNQIACDCDLRWLVNWVISTPRVKLLHEDKTVCGMASLEPFRDKPFL
DFNPTELCGFNVYIVCLPSIAVISLIVTIILVYHFRWQLGRWLFLLKLAALGYMEVRDPRDHDDYEY
DLNVIFHEDDEEWVQEHLRVAIEERLPQFQRKVYGDADLVAGMHYLEAIDYAVTRSYKTVVVLSRAA
IKDRWFMLKFRTAIDHVTDTQTEFVLLLFLEDIPGEELPFLVRLCLSDGRPYVHWPEDIRGREYFFD
ELTAKLTVNLKTNDLIPNE

>Scaffold5411_prot
YDLSLEVTSLYSFILQTFFIMVLDRRTFLVSINLLIVILLQPAAYADVNALPYRDKEGSAPTCDLDV
ASKTADCARLRLTTVPQNLPDGVEFLLLILNRITELFNCSFERYLHITKLDLRFNAIRKVHPSTFRP
LKLLTDLMLSHNKGLLLPHEGLLRWAKELSFIEMSYSCLNSLPDDFLRWNSKLKVVNLGGNELSFIN
ISLCGTIDAFSLSGNRINHLNHDTFRLTCKNESAEMNSLQIDNLHLSENPIVSVDPDVIASLQVRSL
NIGQSRIPTPTLKDIFKGVSHSKIKELTVDNVLNGSISSDLFDSLRNYTIFNLNLAANSIELYYDIF
SSLTNVTLLDLNWNLNKIIEPNFFHGMGKLINLYLGHNEIKTINLNSDVWENDLIEHLDLSYNSLIK
IKKSTFQGLQNLRYLGLDANYDLVSLEPASFVEMKRLETLKLTGTHIMKLKLVTPFLLCLFMNDVRY
FMLRPGSSFNQSRNLINLEISNSMYLSVLWDHTLNVSLFDGLSNLKILRLDGNKMFRDQLQNPIFRS
LSSLQILSLRSSSIQSIPSKAFPGLGSLKELDLSGNYIKKLHPDSLKGLWKLETVDLSANNLVYLDK
DMFSNTPNLSRLILSQNQLTFFNQSTFDPIRSSLSHIDISQNQITCNCDLSWLVRWVVGTPTITLLQ
EEATFCGIASLEPFRDKPFLDFDPTEQCGFNVDIICLPLIAVISLVATIVLVYHFRWQLRRWLFLLK
LAVLGYMEVRDPRDHNDYEYDLNVIFHEDDEEWVQEHLRVAIEERIPQFQRKVFGDADLVAGMYYLE
AVDYAVTRSYKTVVVLSRAAIKDRWFMLKFRTAIDHVTDTQTEFVLVLFLEDIPGEELPFLVRLCLS
DGRPYIHWPEDIRGREYFFDELTAKLTVNLKTNDLIPNE

>LVA_014807_RA_Scaffold605_prot
TMIDLLPLCSKSFSGLDNLEVINLSECSIFQMRLYTPSLRWLSMSNLVAPFSPLVPEETFKGTKSLV
SLDMLESELRNYNLWNAAMNVSIFDGLFNLKTLDLSDNTDLGKSLPKYTFRELSALEELRIEYCHIS
TVHYLTFQGLVSLQKLSLRGNIIQTVHPWLFQGLVQVTSINLKGNKLEYLDKGIFLTNANLTTLSLA
DNKLTSLNQSTFQHIKTSLSSLDLSVNPINCICDLTWLLSWLSNSLNLVNKDETICSLASLEGTRQK
PLLDFDIEELCRINIVLVCVLPLVAICSVAGVGIVYYNRWQLRFKLFLLKLAALGYKEIRDARDHNE
FEFDLNIIFYEDDEEWIREHLQPALEEKLPQFERMVFGDKDLIPGMHYLDSVDYVVSRSYKTLIVLS
RAAVQDRWFMLKLRIAMDHVSDTRTEFVVAIFLEDIPDAEIPFLARLYLSDGRPYLQWPISERGQEC
FWNELSKGLTINLRTNDLIPNE

>Scaffold10504_prot_a
RFVCQKMAAGIPCLLACLFLWCVCLGVQTDEENRRHLHLPCASITKTTANCSNQNRTSVPQSLPNTL
EVLDLSYNNLTSLHNESFELYNLIRILNIAHNFIHFIESGSFRNLQGMERITLNNNVIGELSAQFHM
NNSENTTMILSHNEIREIPHLNGICFFVQGNTREKESRNITLIDFSHNNLTSVKEKDFASFSNCSIY
QFKIGNNKITSLPMKVFSGLSWVGKLDMNCITLEHFEVSAFLGIESIIEITLKGSHIRSIVPLNDST
FISSGQFTKLKILKMKDNKIEQVPDYAFQAFKYLDILDLGTNRIFLLTNKSFCGMRMLKVLNLSKNK
NRSLPRGAFACLGRLEYINLAQNNFFILEPAWFAGCKSLRNLDLFRSNINEITHGSWITTNLQILNL
QYNKLTFTERLMFTGLSHLLILRLAGNGQLYNIAGDTFCDMEALKILQMDNIHRLVLNSTFAELTSL
TFLDISYIPTYGLQSVSVHQFKHTNALQTLNMSYSHVHSHDLVNKKTKESLFYGLTSLRTLKLTGNE
LVRLPNVSWMFSPLQQMEYLDLSQCQLHSLTAEVFKNLTRLRELRLANNELVDIPERAFQNLHNLQN
LLLQFNKLKSIGKNLFKGTANLKQLYLQGNQISTVAPNTIIPIQLNVFNIAGNRLTCNCQLTWFMHW
LRTTNISLGIQNETLCSINSLNGLQNKPVWTFHPKKYCGINIILITGVSIGIIAVLFFSLLVYYKRW
WINHKIFLLKLAVIGYDEITEDADPDDYEYQLNIMFHDDDTEWVNENLKPALEERMPHLQNVAFGDE
ALHPGMYYLNALYHNLDNSFKTALLISNKSVDDAWFLTKLRMAVEHVNDTKLDKIILIFLEDIQEAN
LPYLVRLLLSRNKPYLLVTEDEDGQELFWAQFEKEMRTSKVINGVIP

>Scaffold10504_prot_b
RFEENPLINHKIFLLKLAVIGYDEYTEDADPDDYEYQLNIMFHDDDTEWVNENLKPALEERMPHLQN
VAFGDEALHPGMYYLNALYHNLDNSFKTALLISNKSVDDAWFLTKLRMAVEHVNDTKLDKIILIFLE
DIQEANLPYLVRLLLSRNKPYLLVTEDEDGQELFWAQFEKEMRTSKVINGVIP

>Scaffold11734_prot
RLTFSQTIFCKLDHIVLIILKTRMMWLVLKMATCVLYLMFILVTRSVAIDAIGHLKCAPSNTTAICS
HNISTLILSHNKFWKIPYLHGLCYNRTQIENTGLQELRSVTLIDFSYNNLTSVGERDFASFSKCSIS
QFNLGYNKLASLPKRVFSKFALVKIFDGKHINLDKFEVSSFLGTKNIIQMILQGSQIKFLIPLNDSA
GILQEQFPKIVKLCLNNNRIEHVPNNAFLGFKYLKILDLRDNMIVVASNKSFCKMTMLKALHLSYNK
FKELSNGAFACLKNLEHITLANNKLYKFDPAWFTGCKSLTTLELAHSNINEIEYGSWITTNLQTLDL
SSNNLQSIERFMFAGLSRLMILRLERNIQLQDIAYDTFKEMKALKTLTMHNIGRFILNGTLADMRHL
TLFDCSFTKYGVKFASTHQFTHTSALIKLNMSHSHISSHDIINEETKESLFEGLISLRTLRLQGNNL
RGLPNVSSIFSPLWQIQTLDLCSCNLQRIPSHVFKNMTGLHELRLNSNDIEYIHEHAFQNMRKLESL
LLQFNRINSISKNLFKGTTNLRWLYLHENKITTLAPGTVMPTHFLRVDISGNKLACNCELAWFVNWA
HTTNNVSFAHQNKTLCSSNSFSDLENKPLWSFHPEEYCGINIILVTGVCIAITAMLFLSLLVYYKRW
WFNHKIFLLKLAVTGYNEITEDADPDDYEYQLNIMFHDDDTQWVNENLKPALEERMPHLQNVAFGDE
ALHPGMYYLNALYHNLDNSFKTALLVSNKSVDDAWFITKLRMAVEHVNDTKLDKIILIFLEDIQEAN
LPYLVRLLLSRNKPYLLVTEDEDGQELFWAQLEKK

>Scaffold1406_prot
EKVKGIAFAKNKIRSLQDGAFSCLGNVEYITLAQNNLFILKPAWFAHCKSLKILDLFRSNINEIKDD
SWITPKLQILDLSYNKLISIGRLMFTGLSHLDILKLTGNQLYIIKGDTFRNMTALRSLEMDNIARIV
LNDTFAELRSLIYLDFSFLSSHGAQIASVHQFTHTSALQTLNMSYCSIHSHDLVNTKTKGTLFDGLT
SLRTLRMVGNNLVGLPNVSWIFSPLQRMQILDLGHCQLHSLTSEIFKNLSSLRELRLTNELVDISEN
AFQNLHNLHNLLLQFNKINSIGKNLFKGTANLQRLLLNSNQISTVAPNTIIPSHLNVFNIAGNQLIC
NCQLTWFMQWLRSTNVWLGPHNETLCSIHSFGELKNKPVWTFHPEKYCGINTLLLTGVSFAIIAVSL
LCLLVYYKRWWFDHKIFLLKLAIIGYDEITEDADPDDYEYQLNIMFHDDDTEWVNENLRPALEERMP
HLQKVAFGDEALHPGMYYLNALNHNLDNSFKTALLISNKSVGDAWFMTKLRMAVEHVNDTKLDKIIL
IFLEDIKQANLPYLVRLLLSRNKPYLLVTEDEDGQELFWAQFEKEMRTNKVLNSVIPT

>Scaffold183328_prot
ITEDADPDDYEYQLNIMFHDDDTEWVNENLKPALEERMPHLQNVAFGDEDLHPGMYYLNALYHNLDN
SFKTALLISNKSVDDAWFLTKLRMAVEHVNDTKLDKIILIFLEDIQEANLPYLVRLLLSRNKPYLLV
TEDEDGQELFWAQFEKEMRTSKVINGVIP

>Scaffold4828_prot_a
EYIIVLIIFKILMMFKMAGCRLCLVICLAVWNIGLGENDLGDKRCAFVTKTTAICSHLNLTSVPHSL
PKTLEVFDLSYNNITVLHNKSFDKYNLIRILNISHNVIHAIESSSFGNLCRLEKLILKSNAISKIAK
NLFSNNPNISTLILSHNKLWKIPHLYGFSNTKCKGATGVICTHLHLTSINQSLPNTLETLDLSYNKI

TVLHNESFQKYYLLRVLNISHNSINTIEGNTFGNLHRLETVVLENNVINEVPPSLFVNNPNISTLIL
AHNNIRRIPHLNGLCLNKTPKGITRFQKNLNLIDFSYNNLTSVDEKDFASFSNCSIHEFRLGYNELT
SLPKRVFTEFSSVGTFDGRHVNLSKFEVSSFLGIKSMIEMTLEGSQIKFLVPLNDSTRILRDQLPKI
KKLQLSINKIEQVPDYAFLAFEYLQVLDLSVNGIYNISNKSFCNMTIQNELNLSQNKINGHLYGAFA
CLRNLEHINLAQNRLYKFDPAWFTGCKSLKILNVSRNNINYMDYGLWNTTNLQILDLSANNLESIER
SMFTGLSRLRILRLGRNRELQKIAQDTFSGMTALETLIMNDTGRCVLNGTIADLRKLTFFDCSYTSY
KMNISSIHQFTHTSSLKTLIFYFSRISSAHALVNEETKESLFHGLASLHTLKLRGNHLQDLPDVPFM
FSPLQQMKTLDLVRCDLQRITSQVFKDLGGLEELLLNSNNIVYISEYAFQNLRNLSSLLLQYNRLNS
IGRNLFKGTTNLKRLYLQENKITTIAPGTVFPPRLIRLNIAKNILICNCELAWFVHWTRTATNVSFR
PQNETLCSNNSFIELQNKPIWSFHPEEYCGINIMLVTGVSIAIIIVMFLSLLVYHKRWWFNHKMFLL
KLAILGYDEITEDADPSDYQYQLNIMFHDDDTEWVNENLKPALEERMPHLQNIAFGDKDLHLGMYYI
NAVYHNLDNSFKTVLLISNKSEDDAWFMTKLRMAVEHVNDTKLDKIILIFLEDIQESNLPYLVRLLL
SRNKPYLMVTEDEDGQELFWAHFEKEMKVNKVINSVIPI

>Scaffold4828_prot_b
ELILKGSQINSIIPLNDSRAGFSAVKILELRDNNIQQVPDYAFQGFKFLKLLALNSNSIRDMSTRSF
CQLGMLKMLILGKNKITGLPHGAFACIENLEYLNLAQNSLFILDPAWFTGCTSLRNLKLFQSNINSI
GKTSWNTTNLQIIDLSYNKLTSLEKIMFTGLKQMKILRLTANVLLYKIASDSFNDMKGLEILRLEDI
GTFFINNTLAEMRNLTFLDCSFTKPGVKFGSTHEFMHNSALKTLNMSYSYINPQDLVDEKTKESLFD
GLITLHTLRLAGNDLVSLPNVSWLFSPLQQMKYLELSSCGLNSLNSEVFKNLSNLRHLRLINNKIVK
ISENAFQNMQNLNDLLLQFNRIHSIGKNLLNGTTNLKRLYLQGNKITTIAPGTVFPPRLIRLNISQN
ILICNCELAWFLHWTRTATNISFGPQNETLCSTNSFSELQNQPIWSFHPEEYCGINIILVTCVSIAI
IIVMFLYILVYHKRWWFNHKMFLLKLAILGYDEITEDADPDDYQYQLNIMFHDDDTEWVNENLKPAL
EERMPHLQNIAFGDEDLHLGMYYINAVYHNLDNSFKTVLLISNKSVDDAWFMTKLRMAVEHVNDTKL
DKIILIFLEDIHEANLPYLVRLLLSRNKPVLLVTEDGQELFWAQFEKEMRANKVINSVIP

>Scaffold665_prot
MVIPTRKQDNDNCSWYSNANTLLRVDISGNKLACNCELAWFVNWAHTSNNVSFAHQNKTLCSSNSFS
DLENKPLWSFHPEEYCGINIILVTGVCIAITAMLFLSLLVYYKRWWFNHKIFLLKLAVTGYDEITED
ADPDDYEYQLNIMFHEDDTEWVNENLKPALEERMPHLQNVAFGDEALHPGMYYLNALYHNLDNKLQD
NLLISNKSVDDAWFMTKLRMAVEHVNDTKLDKIILIFLEDIQEANLPYLVRLLLSRNKPYLLVTEDE
DGQELFWAQLEKEMRANKVINSVIP

>LVA_009949_RA_Scaffold947_prot
MDICTLCLFACLILWNNCVGVNAIRTPKCASITKTVATCSHLNLTSVPRSLPNTLEVLDLSYNNLTI
LHNESLGRYNLLRILNISHNFIHVVDSGTFTKLSRLEKIILNNNALSNVSENLILNSPSISTLILSH
NYFWKIPHLNGLCDDKSWTGSSGLSKLRNITLIDFSHNRLTSVEETDFESFSNCSIYQFNLGDNELT
HFPKRVFSRFSWIEKLDVSHINLGKFQVSSFLGIKNTRLSVQRSQIVSIIPLNDSTNVSSKQFPKTK
ILNLRYNNIEKVPDFAFLGFKYLEFLALSTNRIYDMSNKSFCGMIMLKVLHLSQNKIKGLLHGAFTC
LGNLKYIDLARNSLYTLDPAWFAGCTSLRTVDLFRSNINEIEHGSWNTTHLQILNLSFNNLYSIVRL
MFTGLSRLEILRLEGNSQLNNIANDTFREMTALTTLTMEDISGVFLDDTLAELRNLVFLDCSYTSYG
VKFASMHQFTHTPALKILNVSHSNIEYHDLVNKETKESLFDGLISLHTLRLSGNNLLVTLPNVSWMF
SPLQQMKLLDLTSCSLATITSLVFKNLTGLEELRLNSNKIVNISKHAFQNLHHLHDLLLQYNRIRSI
DKDLFKGTRSLKRLYLQNNQIFTITADMTMPPSLISLPISGNPFTCNCQLSWFMNWLRTTNVSLGHE
DEILCSSNSFSGLHNKPVWSFHPEEYCDINTILVSGVSIALVVVTFLCLVVYYKRWWFNHKMFLLKL
AVIGYDEITDDADPDDYEYQLNIMFHDDHTQWVNEILRPALEERMPHLQKVAFGDEALHPGMYYLNA
LYHNLDNSFKTALLISNESVEDAWFMTKLRMAVEHVNDTKLDKIVLIFLEDIQEASLPYLVRLLLSR
NKPYLLVTEDEDGQELFWAQFKKEMRANKFINSVI

>LVA_006843_RA_Scaffold2031_prot
MATFSAASCSTCIFLFIFTSASAFLELELTESLVGSTDLSTGSCKLQNKTKGIKATCSHLGLTSIPP
QLPSNTITLDVSYNQIRALQNDSFEGLLELSSLSLKENHISDLEVGTFLPLKKLRSLDMTGNLLTHL
QPNLFNSNQMLSNLMLFSNNFSSVPTTSLSRLPLLRNIFLGNNSIRVADFTAFSHSRTISQINFANN
QISTIEASDFKPLQNRSLQSISLEDNDLHSLPARVFSHLNNINLLVLDRNALHDFNSSSFLGSMNID
SMSVRGCRIYNIVKLDNSSHDNASFPVIRSIDMSGNIIHYIPPYAFRGFNQTRTLNIQRNKITTMYE
DSFCGLDSLINLDLSDNHIYSIIPGMFSCITKLQELIISRNEIARLNIDSFRGLYALHTLNLSHNIL
KHIDDRQQWDNPALRNLNLSNNRFLTLHPRFFSGNLPNISVVDVSFNKISDYSPDTFNNIPSVKELY
LIAEQNQYLPRVFSQMKNLIKLDISHTRTEWTSLDQFTETSSLQDLRMCYNNLHHYDLFEENMTQSS

LFEGLVSLKKLNLRGNDLNGLDPGVFTPLIKLYTLDLSLCHIAHLTPGVFQGLTSLNNLYLSENVII
TTSAGIFKGLDYLTTLFFRNSRLQIIDKDLFARTPHLKHLYLSGNHISKVQKETFFPTNHTLRIDMS
NNWFSCNCKLSWFINFLRDNDVVLIRPDQTKCYNTSFKAMIGKPITSFTPADFCRVDALLISGLSLL
ALTLIVSLVTAYNKRWWLNHKFFLLKLAIFGYEEMEEDFIANRHEFHLNLMYSEAEDEWVLQVMKPA
LEERLPHLQRVIYGDGDLPLGMFYIEAINDVIDNSFKTILLVSNQSIADPWFMTKLRLAFEHVNDTQ
IDKVILIFIEDIEDENLPYLVRLFLSRNKPYMQWTDDEDGQELFWAQFDKSMRANKAINNNIPL

>LVA_006378_RA_Scaffold2373_prot
CQILLLKYQKIQLSNSETWHNCRNLRWRLAQVRDIDLYDNRKNQSLFAGLCSLRLLRLQNNHLWNLE
SRVFQNLSKLHYLDMSNSKIIMLSSGIFEPLSSLQLLFLEGNQLQKIPEDTLNGLYHLTVISIPDNS
LHDLDTNTFTQNPRLHDLHLAGNQITNIKPGTVFPMNTSFNLDLSRNPLTCSCSLEWFRRWLESNNI
ELKHANQTKCSETSLKGLSGQPILSFHPEDHCGINIVPIVVLSFSGVLVGMIIVLAYTKRWWLNHKF
FLLKLAVVGYNEMAEEFNDVNYYHHLNIMFQESEQEWVDQVMKPGLEERMPHLQNIIFGDEDLHLGM
YYINAIFDALDNSFKTVLLLSNESINDAWTITKLRMALEHINDTGLDKIILIFVEDIEDENMPYLVR
LFLSRNKPYMLWTDDEDGQELFWAQFGNSMRANKAINNAIPL

>LVA_000161_RA_Scaffold4_prot
MLDISHNAVSSIGQERFNGLANLEVLNITDNNINYYSYKAFTNILNLKELYLKNEASTYLKDAFSQL
HTLFILDISNAPIRVSLQTQEQFSNMSSLGELRMENAQLENTTLYDKDKNQSLFAGLSSLYKLRIKD
NYLHDLDIRVFQNLSRLVDLDMTNSRIYTLRSGLLSPLISLRYLYLKQFVELPGDVFNGLFNLKVLY
FQNNILSSLDPQTFAQTLELTDLYLPGNQISTIKPGTVLPGNNSLRLDISSNPFSCTCSLKWFRQWL
DTANIDFKHADKTLCSGTSLKGFKKQPILSFHPEDYCGVNIFLIAVVSFAGIFFCLLTILAYHRRWW
LNHKIFLLKLAVLGYKEMAEDFEADHYEFHLNLMFHEDEEEWVDRVMRPALEERLPHLRNIIYGDKD
LHLGMFYINAIFHALDNSFKTVLLISNRSVDDAWCMTKLRMALEHINDTGLDKIVLLFVEEIDDENL
PYLVRLFLSKNKPYMLWTDDEDGQELFWAQFRR

>LVA_001232_RA_Scaffold603_prot
VHQGFSGIITVISEPRNMATNLLLVALCFHVHVALLAGGQSLTSPHAARSQVVGSASTLISYSNHTK
CHLRNSSLGLIADCGHLKLRDIPQDLPTDVQALDMTFNKVEVLWNESFQNVPHLVTLNMEHNQLMLI
ETKALWPLTELRNLILKSNNLVTLSPMLFIKNSFLTKLFLNNNKLINIPCKALAFLPRLTRMYVQNN
KIKRLNFDDCSEWSHLSNIYVSNNEIVEIHQRDFLPLQSLPLSYLFINGNKINMFPREAFYHLNLLR
LIKLDSNTIESFDIQPFLGMDFIARLSVKDGKISKLLPPQNTTNKGDHLYPTIISLDIANNLIESVP
PNSFWGFPKLQILNLTRNKISILVNKSFCQMQSLFELNLSSNKIMFLPTNTFACLSCLKILNLMNNL
LQSIYPESFNGLPRIQSVLLSHNSIKYLNRGRQAWTLRTLQSIDFSFNDITSIGKDSFTGLINLKDL
ILSYNQIYYFYQTAFSELFKLSRLHLTNERKIFLEKTFKQLHSLTFLDLSNSPIKISRYQTEQFRNL
TRLQELRMEMAQLSNTDLYNSQRNESLFTGLCSLQKLNLKDNSLMNLDRRVFQALSNLRYLDMTNSR
IKVLKSGIFSPLSSLRGLYLGSNELQKIPGDIFYGLHDLTVVKIQNNRIFDLDPRTFAQNPRLDILY
LSGNQITNIKPGTVLPSNSPFNLDLSRNPITCSCSLAWFRKWLELSNVNFKNANQAICSGTSLKGLA
SKPILSFHPDDHCGINIVLILVLSFSGVLVGIMIIIAYTKRWWLNHKFFLLKLAIVGYKEMAEEFND
VNYRHHLNIMFQESEQEWIDRVMKPGLEARMPHLQNIIFGDEDLHIGMYYINAIFDALDNSFKTVLL
LSNESINDAWTMTKVRMALEYINDTGLDKIILIFVEDIEDENMPYLVRLFLSRNKPYMLWTDDEDGQ
ELFWAQFEKSMRANKAINNAIP

>Scaffold654_prot
DILDCLKRVRTTVITMAVTCKRRDAKSSFWTVLFSFIFWLVCLFTAATAEEPVGQSSMVQPKKCMIT
YKIYGVKASCSNMSLTSIPHDPPPDTITLIMDNNLIVHLKNNSFDNLTQLATLNIRLNLISKLDTGA
FSPLTRLEILDLTGNMLTQLPDGLFDSNSFLSRLVLTSNCFKTVPSNTLMTHLDQLRYLSLGRNAIM
EVNFTAFQFLKNLSVIDFSGNNIASLRPSDFEHLHKLSLNSINLASNELSGEKLPDRIFSFLNQVVS
LNLYGNQLRVFRLNPFLGNVTISILSLSNCGIFLFEPFNSSTLPTVTLPTIYDIDMKQNSIDSIPSN
AFQGLNQTMSLNLAENKIKYMTNQTFCGMNSLVILDLSKNRIENFTNGIFLCTPRLQILTVAYNDIL
VLSPRIFQRLSVLHRLDLSHNAMRYVFPGNWNNSGLQILDMSHNLFEKTYRTLFRGWLPNLKVLDMS
FNLIETFYANTFDNVPSLREIYLGNMLTSVITLNGVFNHTKNLVKLSISSTRITSLRPSHQFTGSVS
LEELSMCENDVGIENLFNSATNSSLFKGLTSLRKLTLRENHLMHIVPGVFRPLIALLSLDLSNSKIE
TLEPGSFRGLTSLKSLYLNDNCIKRISADTFSGLGNLVSLFLRKSKIVALEPGSFQGLASLRSIYLS
DNRITSISTNTLSGLDNLISLYIKNNLLRFLDKRIFAATPNLHILSLSNNVIRTVQKDTFFPKSNIT
FIIDISGNPLSCNCELSWFRTWLNEHTTSIHHLQETVCSKGSFETVFGKPILEFDPTYVCGFNTIPI
TSLTLFTCIVIYLSLLVYHKRSWLRIKFVFLKQAIRGSGRIVENFKDENYDFHLNIMFHDGEEEWLD
RVMKPVLAERFPNIKKIIYGDGELRREMFYINAIYDAIENSFKTVLLISNRSLRDIWCITKFRLAVE

HVSDTGLDKFILIFIEDIEEDDMPSLIQVFLSENKPHMWWTRDEDEQEMFWAQFHRSMKANRAVINA
IPLYL

>LVA_000603_RA_Scaffold695_prot
MANCAPCFISVLAAVEFFLMHILPLTNNAPAEDYRWNPRNCSFINSSHGLSVNCKNKGLSDVPPHLP
EEATLLDLSKNNLTILHNSSFEDVPNLVSLIASGNPISFIETKTMWPLTKLRSLLLISCHLQTMSPL
LFIKNNLLTRLQLKRNHLNVIPSETLSVITRMSAMELSYNRIHGLNFDGYPIHHNLKRINLLVNSIE
EVHPSDFLTLQNSSISFLSLASNKIKKLHRYAFHHLHSIKSLHLEGNQLNSFDIQPFLGMTSIGELS
VYGCEIYQLLTLGNASYQNESIPSVSILQMRGNMIKNVPTGSFWGFTRLQTLVLRENKIRTFGNQSF
CLLRSLRELDISNNDIESIPAQTFACLPRLMKLNVSHNFIRTLSPLSFNGIPRITFIGLSHNSIVDL
NSDKTVWTLETLLKLDISNNDICSISQSIFKGLTYLTELNLSFNPCYFYSAKAFKDLPNLERLLLFN
QKKIYLQTTFQQLHTVLYLDISNAQVKASRYSIEQFVNMTHLHDLHIKRAQLTSGDLYDDISNQSLF
TGLYSLKKLHLRENYLHSLDSRVFNNLPNLFYLDMKNCRILELRPTLFHSLPSLAILYLSGNKLVKI
SEDTFHGLFYLRVLYIQNNSLHGLETTTFAQNSRLTDLYLPGNQISVIKPGTVLPSNNSLRLDVSRN
PFTCGCSLTWFRQWLDTADIDFRHPNQTVCSSTSLKELNNKPILSFQPKDHCGVNIVLIAVLSFSGL
LVVMLAMLAYHKRWWLNHKLFLLKLAVVGYEEMEEEFNADNYHYHLNLMFQETEQEWVNQIMKPVLE
ERLPHLQNIIFGDEDLHLGMYYVNALYDAIDNSFKTVLLISNQSVKDAWTMTKLRMALEHVNDTAFD
KVILIFIEEIEDENMPYLVRLFLSRNKPYMLWTDDADGQELFWAQFERSMSTNRAINNAIP

>LVA_006686_RA_Scaffold804_prot
MLSGNKIETVPVGAFWDFTTLEVLSLRLNKIKLLTNRSLCHLESLKELDISYNKITTFSNATFACLQ
NLKVLNASGNLLQALLPGYFNGLRSILTITVSSNRIEELNNGKHLWTVKTLCMLNISHNALISISQD
RFNGLANLENLSRLVHLDMTNSRIHTLRSGLLSPFSSLRYLYLSENSLVELPGDVFNGLFNLKVLYF
QNNILSSLDLQTFAQTLELTDLYLPGNQISTIKPGTVLPGNNLLRLDISNNPFSCTCSLKWFRQWLH
TANINFKHADKMLCSGTSLKGFKKQPIPSFHHEDYCGVNIFLIAGVSFAGIFICLLMILAYHRRWWL
NHKIFLLKFAVVGYKEMAEDFEADHHEFHLNLMFHEDEEEWVDRVMRSSLEERLPHLRNIIYGDKDL
HLGMFYINAIFHALDNSFKTVLLISNQSVDDAWCMTKLRMALEHINNIGLDKIILLFVEIDDENKPY
MLWTDDEDGQELFWVQFEKSMRSNKAINNAIP

>LVA_009873_RA_Scaffold2711_prot
QAVLFNCDIFKITIMRAVDTTKRVYACLWILLFAMYMLLAVSPAKNVLGSSSTESTRGCTLKNTSLG
TKAICTHLHLKSVPQNLPSNTVIFDISFNMISTLFNSSFLYLPSIDTLGLEHNILSKIEHDAFEPLS
HLRNLSLTGNRLVSLPSGLFKANHFLSRLILTGNRLIHFPNNALPESKSISKLDVSGNKISFIDSCD
FEPLQNCSLEILNLKSNALHSLQFNIFSYLHSVRLLQLTANTFKNFTPSMVLGRTNITHLDVSGCNI
QHIIPWNKSNVSLENYGKISELILNSNKIRYIPDFAFWGFSQTEIVLLHNSQVSKLSNKSFCGLDKL
IILDLSYNHLTTLSWNTFSCMEMLSKLKLNNNQITSVSIDLVSGLSSLSHLNLAHNSINDIERSNVT
IPSVEYIDLSFNKFKGIRRLLMRSFPNLKILNMSNNGITNQYSPHSFLNLRHLQELYLVNENHQDIN
YAFRYLVHLLVLDLSSAPLRFFNLSQFTNTSSLQRLAMRDNSLRSADIYHAETNRTLFWGLSSLETL
DLRKNKLDMLAPGTFNPLTNLKILYLSKCTITVLSCGVFDNLTALRTLDLRDNDIMKVPESLLQRQH
HLAVLFLGNNRLESIPRILFKETTSLHSLFIQQNRIAIIEPMTSFPTNTTLRIHAAGNPFSCTCRLS
WFVKWLRSDNAELWRPKQTLCSLTSLEAEVNSPILTFNPDKYCGIDIVMITCVTLSGLLVLVIGWVA
YKQRWWLNYKLFLLKLAIFGYEEIKQEFDEQEYEYQLNIMYNEEDQEWVDRIMKPVLQERFPHLQKV
AFGDNDLNIGMFYLNALHYVVDNSFKTVLLISYNCINDAWFLTKVRIALEQINDTKLDMVILIFLED
IQDEDLPYLVRLFLSKNKPYMLWTDDEDGQELFWAQFEKSIRSNKAINSVIPV

>LVA_015343_RA_Scaffold2840_prot
MASTPLYTFLCFGITWVLFFLSGGLSVNDVSGRNGSPNWSSVSTGGCQLTHTSRGIEARCKRLSLHF
VPKGLPYNTIVLDLSRNVIETLYNRSFSYLPDIIKLDLSFNDITLIEDGTFKPLCNLTELNLGGNHI
ESLPAGLFHSNKLLSVFKGFYNQLSSFPREALPTSNHMKTLDLTHNPKISSIKSLDFAPLQNSSLMT
LDISLCNLINLPTNTFSALKSIKYLNLDFNHHMEEFHISAMLGINEINDVRLSSCNIKSIIPLNKSF
HNPHGSLKIHQIGLYWNDLISFPDFVFWGLNQTNVLRLDHNKISTLSNCTFCGLDQLTELDLSHNII
RYLPQDMFFCNKQLKKLRLNQNLIESWSGSTVSNLCSLNHLDLSHNKIQSIGSTTQTHPSLEYLDLS
FNKFEQVHAFFLQSYPNLTLLDMSYNDMYWISPQVFSTLSNLKELYLTNEQFNMKVFSIAFQNIRNL
HTLDLSSAMQYKTFDAWLLTNFTSLRRLLLCKNNLQSRHLFNTVTTQSQFISLETLLTLDLKENDLD
MLAPGTFDPLKKLEILLLSQSSIKVLVSGVFEGLTSLKTLDLSENYISSYFGDIFPKQSQLSLLNLS
NNNVNLLPDTLFNTNPQLQSLYIQQNQITTIKQGTMFPKNFTIDASGNPFVCTCDLRWFLKWLSSSN

VEVIHPNDTNCSQSSFKDMVKSPILSFNPDKYCGINILLITSVSFAALLVAAFSLLAYWKRWWFNYK
VFLLRLAICGYKELVQDFEDQDYEYQLNLMYQEEDEEWVDDIMKPVLQERFPQLERVAFGDNDLHLG
MFYINALHYAVENSFKTVILLSKNSVREAWFITKVRIALEELNDSRLDKIILFFLEDIEDDDLPYLV
RLFLSKNKPYMLWTDDEDGQELFWAQFEKSMRGNRQLNSVIPV

>LVA_020042_RA_Scaffold3469_prot
MAVDTTMNASRVSSRLGILLFTMSLLLAEWPVKDVSGSLSSESTEGCILKNTHLGTKAICTHLHLKS
VPQDLPRNAVTVDLSFNTIPTLFNGSFAYLPNITSLGLEHNVLLTIEHGAFEPLSHLRKLSLKHNRL
DSLPSGLFRVNLFLSILILGKNRLVSFPRSALPWSNSITTLDVSQNRIAFLDALDFEPLQNCSLERL
YLGRLYALYTLPFKVFSTLNTVKWMSLSKNNFQNFTPSAVLGRSAITHLDLSSCNIENIISQNKSHI
ALVNGPISMLLMKGNKISHLPDYAFWGFNQTKTILLHNSRVASLSSKSFFGLDNLIDLDLSFNLLTA
LSLKTFSCLHMLSTLKLNGNQIARIFIDLVSGLSSLSHLNLAHNNIEEIPRSNVTIPSVEYIDLSFN
KFKTIRRFFMWSFTNLQILNMSNNDISQSYSPYSFNNLRHLQELHLTNEYQQLINSAFRYLGHLQVL
DLSFAPLKMTTLRQFTNASSLNRLIMRDCSLKSADIHNAKKNRTLFWGLNSLKVLDLRQNQFDILAP
GTFNPMKKLRVLYLSQCTISVLSCGVFDSLTALTTLDVRDNAITKVPESLLQRQHYLAVLFLGNNKL
ETIPGTLFKETTSLHSLFIQQNRITTIEPMTSFPTNTTLRIDAAGNPFSCTCQLSWFVKWLRSGNIE
LRRPKQTLCSLTSIKAEVDSPILTFNPDKYCGLDFVMITSRWWLNYKFFLLKLAIFGYEEINHEFDT
QDYEYQLNIMYNEDDQEWVDGILKPVLQERFPHLQKVAFGDNDLNIAMFYINALHYVVDNSFKTVLL
ISYNCINDAWFLTKLRIALEQINDTKLDMVILIFLEDIRDADLPYLVRLFLSKNKPYMLWTDDEDGQ
ELFWAQFEKSMRSNRAINSVIPV

>Scaffold4000_prot
IINNNHVDIIPKTLFNTSPHLQSLYIQQNKITTIKEGTIFPKNFTIDASGNPFSCNCDLRWFVKWLR
LSNVEVIHPNDTLCSQSSIKDMVDSPLLSFNLAKYCGINIFLITSVSLTVLLVVSLSLLAYWKRWWF
NYKVFLLRLAICGYKEMVQYQLNLMYQEEDQEWVDDIMKPVLQERFPHLERVVFGDNDLHLGMFYIN
ALHYAVENSFKTVLLLSNNSVREAWFITKVRIALEELNDSRLDKVILFFLEDIDDEDLPYLVRLFLS
KNKPYMLWTDDEDGQELFWAQFEKSMRSNKELNSVIP

>Scaffold4549_prot
LNLMYQEEDQEWVEDVMDLHLGMFYINALHYAVENSFKTVLLLSNNSVREAWFITKVRIALEELNDS
RLDKIILFFLEDINDDDLPYLVRLFLSRDKPYMLWTDDEDGQELFWAQFEKSMRANRELNSVIPI

>Scaffold888_prot
QSWYYFHDPIQYKPRFNNLGIISMTLFNINPDFQSLYIQQNKITTIKRGTIIPKNITIDASGNPFSC
TCDLRWFVKWLRLSNVEVIHPNDTLCSHSSIKDMVASPILSFNLAKYCGINIFLITSVSLTMLLVVS
LSLLAYWKRWWFNYKVFLLRLAICGYKEMVQDFEDQDYEYQLNLMYQEEDQEWVDDIMKPVLEERFP
HLERVVFGDNDLHLGMFYINALHYAVENSFKTVLLLSNNSVREAWFITKVRIALEELNDSKLDKVIL
FFLEDIDDEDLPYLVRLFLSKNKPYMLWTDDEDGQELFWAQFEKSMRSNKELNSVIP

>Scaffold1985_prot
CMMALYQTLQVIFMLLHGLLVGVRVDAGMDDINTRLTHTCSLNLTEHLANCSYRDLPSIPQNLPHDI
RSLNVSNNNISALLDTSFTNYSLIHTLDCSYNSIDFIHNKTFHGLTYLQILKLQYNKIGFLPISLLE
ENIHISLLIFHHNRLTEISPVFRQSIHSVGEDGDVGCKNVSRFDLSFNRITAVEKEDFEGVQDCYID
SFYLNANRIKSLPRGVFTYLPAVNLLINRIELHEFITSSFIGNKAIVKATMTGSGIRSIIPMNISNI
PNNILPGVNKLYLQSNKLSTIPSYALQGFEHLQFLDIGSNRITSVHEDSFCGLKSLVSLRLAVNKMK
TLPRNSFACAQKLERIDLSHNDFFELDPAWFNGSQRLRTLIVYKSNIKDIHPVPWNVSNLQSLTLSN
NDVRSINRNSFIGLSSLRVLDLTRNREPFDISIDAFEQNYNLERIIMEDLIKITLNGCFSNMHRLNF
LDLSYSASRLDISSRDQFKNTSALRTLNLSRTYLQTEDLVQFNGRMPLFSGLVSLIIILNIQHNHFND
FHSAPYAFTPLYNLQELDLTDCRIERIDSRMFRNLSSLRKLSLAVNYLKDIPEESFHDLTNLRILRL
EFNAVAVIGKRLFSRTSHLKLLSVKNNQISTIEPFTVFPINLQTLVIANNPLTCTCQLAWFREWIEK
VNVDFYEKNDTRCSSTSLGTLNNQTLWSFNPKDYCGVDVFLIVGVSLAVVTVLSVGILVYIKRWWLN
YKMFLLKLAIVGYQEFIEDRTADDYEYQLNLMFHEDNEEWVNDSMKPFLEERMPHLEHIVFGDRDLH
PGFFYLDAICDVIENSHKTVLLLSNQSVKDPWFMTKLRMSVEHMNDTKLEKVILIFLEDIENDRLPY
LVRLLLSRNKPYLLWVDDDEDGQELFWAKFQKRLKANREMNNVIPV

>Scaffold256_prot
VKDNKHFFFIQSGYPALLRDTMFMHSPRKRSVVPSKMQFIVILLISALKGGTSSVAFAEEVGHYCHL
STAISGVKADCSHLSLSGIPTDLPHETTTLDASFNELEVLHNSSFTGLHQLITLKVVKSHIYHLEVG

TFTPLTRLQTLVLTGNSLARLPDNIFESKQFLTTVLLSRNNLSSVPTRALNSLQLRKIRLDYNLIKK
VDFTAFSHTNNRANITLKGNSISSMQPIDFEPLQNVSLHKLDLSSNQLRNISKLIFSYLNNVYDLID
LNSNKLREFNLDSFLGNVTIGTLSLADCGIYLIIPLKNYSFINVTFPKIDQVILRGNTIREIPSSAF
WGLSQTRKLNLASNKISKVNNESFCGLDSLTNLYLSYNRIYNLPNQMFSCNPELQILLIAHNSIHKL
NPIFFSGLLALQQLDISRNGAEVLGTKWNNPALCILDISGNEITRLNDKKFEGNLSSLQKLYLSGNK
LDKLSPSTFQNVQLLQEIYLGSQHGGKLHLNGVFSKMKSLIKLDLSKTNITLTSTCQFNETMNLEEL
HLNFTRLNSTNLYDIERQSSLFDGLVSLSKLYLRGNNLNNLEQGTFKSLSKLSALDMSKARIVVIKS
GLFHGLSSLTWLNLDSNNINKISANVLSGLNYLKFLYFGYNRIEVIEKDSFKMTPNLHKLSLSSNRL
TKVEKDTFFPTNRTLNLDIHDNPFSCTCDLAWFISWLGESNVHLKNPSQTVCYSTPIDKFVGLPILS
FDPKLCAANFELLIIPLSGLMVGFLIVLAYINRNRVKDKLSPLKLAIIDYLQVVESLDADDHAFHLN
LMFHESQEEWVDQILKPAIEERLPHLQNIVYGDKDLCHGMFYVNAIQHAIENSFKTVLLVSNSSVDD
VWIRTKLRMALEHVNDTGFEKVLLIFLEDIEEDQLPYLARLFVRRNRPHIFWTEDEDGQKLFWATFK
RSMRVNKAINSSIPF

>LVA_007966_RA_Scaffold1160_prot
MVICIVVYLSLCVGLLPQCTKGSPTSGIKPFPKECIFVSDTFIDCRHRDLRTIPSGFPETVETLLLS
YNSIHVITNESFHGLVNLVTLELHHNSISKLQSDFFKDQAKLRYLSLSHNQIGDKLPDDAFEHLPAL
EVLDLSYMNHGFTVLPLALQQLIHLRSLNVSSNRLQSAGFTPGVAFPALLELRLGKNQIQSLQNEDF
KALLNCNLDFLDLTENQISAIDSGVFHPIASIAELDFSKGLRPAAIPSLAEALTDYQVDALYLREIG
LEIWNSNNFKYLRNSSLEKLDISFNNITTLDSGFSGLSNISSLIIQSSHVAHISNTAFSGLELVVTL
DLSGNDIAAITDGMFSSLSNTRLKTLYLDNNKIKRIDASGGFSGLSNLHYLKISKNKIEQNFIGDEF
KDLDSLDVLDMGVNRNISLSPDAFRLLKNLQTLYLNLANLKNITVVPSPFNKLSSLKKLDLSNNNMA
ALHKDTLSNLSNLETVYLQHNNLYNMWNETVYVPFLKYLRRLKHLNLCFNGFQNIPNDSLSSLPELE
TLLLCHNKISQLQDGILDGLPLTTLDLGYNEINLINQTLIEPLQGSLQILAVSGNPFSCGCNLQWFR
NWLDVTKVHVNDSSHMKCASPPEMRGKLVMDFHPESLNCDHLLPFYIWIIIGVGSGMVFVIVGLSVK
FRFHINYCWNLVNARRRKYQRIKGEDRPFLYDAFVSFSSKDEDWVKNELVQNLEGEHGFRLCLHDRD
FILGRKILDSIVEAVDSSRFTLCIMSTNYLDSHWCKMEREFAMANLIDRDVLIIIALGEIPEKKLTK
YYKLHRLMMKRTYLAWPDEPGVRRNDFWVKLRTVLREPELRVNNNVSI

>LVA_005397_RA_Scaffold3166_prot
MGGSGLTRLLPVLSIAVWAFADVPYQYETSGGNRSDHPGVTSVNCSLGSSMKNISCAGLGLQDVPQD
LIPDVEDLDLSFNHIRYLSNSSFSPYRRLRILSLYSNHLRKMSIGCFYAIPNLEMLDLGNNDLRTVR
SDMFKKSKNLVSLSFYKNMALEFIPNDILSWLPKLRTLNLGLTFISRINLSSCSSKTPELDINLENT
WIANITDDTLAINCEINSLSIKQKLIPTSVTKPYTIASLHSRIFSFSSIMVSVEQWKPFFRGIANSK
IEELVLQDSRIEEIDQSYFEPLQNKELQILDLSHNSFRNITNSGFLELPLVHTLILDYCQIKEIHPE
DFTKMKGLRVLHLNFNWINYINDYYGNVTWDIDLQELYLAFNSLHSIHLKSFEGLNSLKILDLKGNE
NLEQVFIAPSTGLWNLQSLDLSQTSLDIMTIYAPNLQSFISNDCEEYFSIYLSELGMLFNRSKLFLE
NVYLENNDLWDLGYSIDGSFVSVFQGLRKLKLLDVKKNPLKYLKAGLFTDLQYLQILDLSYCELSYL
ESKAFEGLQRLKNLHLQGNNLHELPSRLFENTGELAMIHLEENILRYLDSDLFVNSSNLRNLTLQRN
RLSGFNRSTFAPLFDHLYSIDISKNEVVCTCSPASLEELKEKPLMSFKPAELCVQDIGLYCSLPIVT
IWIFMVLAFAYHYRWFLKYKLFLMKLAVIGYRQIQDARDFDKYEFHLNVMFAEEDEAWVRERLRPVL
EEMLPDHNRNIFGDNDLPLGMHYNEAVDYAVENSYKTIILVSRAAIQDNWFVIKFRIAADQVNDSQL
ESVVVVFLEDIPGDELPFLVRLYLSVRQPYLGWEEDERFQEYFWQTLIKMLTVNLRSNNAIPS

>Scaffold3980_prot
MLLSKIMAELRSIQLGLILYLTSITIVMMVMAKEESFQDVLQLKRQKHLNLASRNCSSDPLRRRISC
SNKGFQNVPQDLFSFVEELDLSYNNIRFLLNMSFSHYRCLRNLSLYGNQISNIALGSFHALSDLEHL
NLGRNLMLTSIASDMFRNCTKLSSLSFAEDSLESIPGNILKWVPNLQILDLRFTHIRTVNVTSCSSE
IVPKLEINLEGTRITAIANDTFRIDCKLHHLDLSSLKNITAIDQKSVSLIRSYSLSFSRISMAPDLW
DNFLKGLANTETEQLFLQANDFGNTDVHDFELLKRLKLLDLTGSTSHINHIGLFNSPQLLTLILEDC
SIKDNELTATYFSLMKSLSNLNLASNGIIDINPAYSKFRWYNLDLQEVDLQYNELNTIHEWSFRGLT
NLTSLYLRGNTALAYVNVSAFTDLIHLRFLDLSGCSVIKEVSVYLPHLETFIFNNCTQPSWISVKTI
FVQTLKSLKHVYMGNASMNSLWDGRNSGSQSSLFQGMRNLLTLDLKENPIIELRSGLFLDLIFLEEL
NLCDCRLRWIESNTFEGLQSLKSLYLQNNELKTLPFEVLQSMGQLQTLNLDGNKLKYIIGDLFINSS
KLNSLILANNSLSVLNRSTFEPILNSLGLIDISDNILACTCNLKWLPEWLRGPIVVLNEARTNCSLA
SLEELKGKPLLTFDPAEYCGPNMTVLLMSSLLPLVIAVIAAVMIVAHRYRWFLRYKLFLLKLAVIGF
REVEDARDFNDFEFHFNIMFAEEDERWVMERFRPVLEELLPEYDRNVYGDGDLPLGMYYCDAVHYVV

ERSFKTIVLVSRPAIQDNWFVIKFRTAADQVNDTQVENMVVIFLEDIPDDELPFLVRLYLSDRQPYL
GWEEDERFHEYFWQILSKMLTINLRCNNVVPPE

>Scaffold9761_prot
AMACQIRLCWFLVFVLRVTSRRNEMMTTKEPENDPTSTRIAWAFTSEKCDFDVIAKRADCRQRGLAK
IPDDLPTDALHLDLSLNNIRSLNNVSFIRYTLLHFLDLSKNDITTIQPEAFKPLENLATLDLSDNPS
LSSIPYLQWLHKLELLLLNGCNFTSLPDEVLGISSKFLNLDMSYNNLASVNVTICSDLVLPDLNIGY
NGIESITPETFAILCPIDTIDMQNPITSIDPAAIAPLNAQNLVVGAFPTTNAVLTQLFQGAAISSIK
EISIIGSGLDAIIPDLFSPLSNASLFGLDLSFNDFISLNHSIFSNLTTLHRLSLSGNYISEIEPEHF
AGMKELRILDLGYNEVQTINPKNSTWEINLHELYLLDNSLIHIASFSFSGLNTLVLLDLSNNEDLSS
LEIGAFDALEKLKILDVPGCKLLDMSLWAPLLTSFNIRQRVPYFGDDSFIPGETFKTAPLLEFIDMT
GSIMLVNSLWDSSKNLSLFSGLTYLRTLFLDNNPLGGFPVGVFSNLTSLQKLSVVKCEIPFLEMGLF
SDLRSLKELHLQQNYLTHLSSDLLQGLTNLLALHVDDNHLSYFSEDAFTKIPLLTTLILTNNDLTAF
NSSSFDPVMSTLTAISLINNPIVCSCKIRWMVEWLRGDIHLINADQTYCSSASLDPLKQKPLLTFQP
DEFCSPNTTLISLLILTGIGVIIVALMMYSNRWALKYKIFLLKLAILGYDEMEDARGHDEFTYDINI
MCDDGDDQWMKDHFKPLIEEKLPDLNRNIYGDEDLIIGMHYLDSVHYIVERSYKTVLLLSRNAFRNN
WFLVKFRIALDHVNESQIENMVVVFLEDIPDAELPFLVRLFLGDHGTYLTWSENEEEQEYFWTKFVK
LMKVNRRSNHIIPP

>Scaffold10111_prot
NYTFRTLGQVNSSYCRKGGINPIHNVLISLGAITGVIAVVFMCFVIYKKNETLIKLMLLEYFPEDMS
DEDANKPFDVFISYSQLDDEYVLRYLVPLLENEEEASYSICLHHRHFIPGDAIANNIVSAVAQSRRV
ILVLSKNFLESDWCMYEFRMAHLQALQDRRNTLLIITLGDICEDNLDPDLKAHLRTTTYLESSDSKF
KYKLFLALKRRRSKRVTSNNQMGLIDLEGL

>Scaffold2440_prot
EPISNQDQRMDIVRVQACLMFLGVLLSLSSIVADNLISKMKNVNRRHHESARMARSYPISCNETDLP
CLCNGPTQETGPLIEFTSISCFLSSNWNVTLQRIPRTRSLTLNCYSHGNRTNDPADLKVDLFDNLSG
VLQELTMIGCKIGSFPSEIFADMALLQKLLLVSADLDGDRLEAIGGIKTNASVKLSFNHLHSLDAHS
FGFQLSNISTLDLSFNNIAEIGIDTFMVFPSLRKLFLGNNSVSVVTGDGFRGLQSLTELNIRANNNL
FNDESRCLFQNTASLITVDLSSTGLTNASHFNCAPLLHLRRLIIHDNNLPSLDGYVFVHMPNVTFID
LSNNALEYIHPSAFHGGLNDLKYVNMSGNDLQEFPVFAFESTPNIRLINISHSHLRVIRMGTFSSLP
SLQTIDLSFNRLQTIEVLGIVNLDSLTMIDLRYNDFVLFPDNLVWPFDIRPPHIPIDTLLQGNRFTC
GCPVMLFIRRGDFEDTYPFFHLPDNSSWTCNAPSPVAAKSMMTLPLEDFWCPYEDNTCKQGSCECYR
RDIDRANIFFCPDNSLHSLPNIPAETLIFDCNGCLIDDHYTLRAEDFQTSAKLAVLALRDIGLETIL
SDALLGFPNLQQLNLSNNVLNRFDVDILTDLTDLREIDLSMNGVNSLSSRTFKRNPNLTTINIHSNS
LQSLEDGIFNSTSHLKVLTLHDNPFICNCSLFWLKQWLQSHIDVVPRLYDVECYVNSSDNAMYPIIQ
VADQDFGCSSPDVLTIQEYNAVIATSTLTLVFLMVMAVIFRHRRAIRVILYTRYGFHVLHDDDDDDV
VLDNIRWEYDAYIAYSDEDIEYVLKNIIPILEEDINLPYKLCIRHRDFPPGGCIATTIVTSLEASRR
SIIVISRSFLQDEWRLLEFKTAHQRVLQDKRNKNLILVFLEDLDRDEMDEDMRYYVTANAYLSTTNR
LFRENLLYEMPRHPLAEIHGDGDHQ

>Scaffold5432_prot
LCFNSIEYNLQNSILLSKKFVMMGVYVEIKFSSMMFSMLLCFCEALVNSSVFTPYVVDCPIVPLPEG
NSFCQCGPPNITVTSVDLLPKFDYVTCKLTQDLISETNPNPFYFPRSRKVYVDCSWNNSLSYHDLLK
SVEKESGDIAEEVQAVGDIDCFTPFALTADTYEHLPGLQKLNFFFEDVTIGRNAFYPISNLEVLSLI
HLDLKSLHPEVFRGLTHLRQLSLWDNDLRELPDGIFNDLQDLQDLSLTSNNIVHISRNLLRPLEFLN
NLFLNNNNISYIHPDAFRPLRSLEEVDLSFNNLDGNLRLSFLSTRKLSLSHNSLTSFTEDTIVGGRG
HTASLDLSYNLITSITEKVFHTNYLLSPLLSVHLNNNRLESLPKNILRWGRRLTVIDLSNNSLETLP
NGLFDIWSNPLGEQRLAGNQLVIKLSGNPFTCDCRLSWFRLYDGQDVWISDRDDIKCFSPPNLQGLS
LFSVNPNQFECPLADSVCPKPCKCYKTVSIKGIDRTPHTMIFVDCKYANLSHIPLGVPMNTTSLEFT
GNTLKTLRETMLNTRLSMLKNLTFSMCNIEKIEGGSFRSMDSVLSLKLDGNNFNVTKGIFQGLTQLN
TLYLNHSSIRTITEGVFLDTPSLTQVYLHGNLLTVLPAFHSLPKSLEIMSLQHNPLVCSCNLIAYQG
LTRYTPGVVGNATCKQKGNAFVTLGQVNSSYCRKGGINPIHNVLISLGAITGVIAVVFMCFVIYKKN
ETLIKLMLLEYFPEDMSEEDANKPFDVFISYSQLDDEFVLRYLVPLLENEEEASYSICLHHRHFIPG
DTIANNIVSAVAQSRRVILVLSKNFLESDWCMYEFRMAHLQALQDRRNTLLIITLGDISEDSLDPDL
KAHLRTTTYLESSDSKFKYKLYLALKRGRLRRRTSNDMKLADLHA
>Scaffold1584_prot

TLFRITMDITKRICLWFSFLLYFGSCNSMNFCEYCECDSTEIFCVDVNFNEALNHSVPSAVSSRAKY
LNIGPSDDRVILANDTLNVFPALHSLRIHTCPVAFVGQFVFQKLYHLSDISLEMNGLHEIPERALSQ
LKASLQELTLAGQHFRTIPSDAFGELSNLRALTITYNTNHLYISPDAFTGLVKLTGLYLSACKIGHI
HNTTFWGLTKLQELDLSYNLLVEIPPAILSLNSLLKLDLSYCTHLVNPMDLIILKNATRLQVLQLGY
CAISEISPTAVAKLKGTPSLTVANFDGNPFNCTLDLCSFASWYVSLLVPAITTRGPDFIPFITLSPP
PGKGPYQCESNGQSLQEFFGETCIPDPGPSTLPPNEMQLHLVILCVALIIVLFVVILVSFVVWKFRL
ISIHHRRLGNAFQRQANYGAVEHVDREYVFDAYVSHHEDDKPFVEDEMLPRLEDESGFDLCVSFRNF
RLGSHLLDNVSSAQDVSRAIIFIINERFMQNGQCKLELEMASTRMLEDEVGPGGQRIILIMMEVLAP
ELVNNTLRMLLNHVAYLEWDPVAEDRCWGQLIATLHALVPDRNEENGGSVDQGSDDEIEVVENEHDG
NQI
>Scaffold1935_prot
FWFGFIIFINNHLCCCLADIIIFREGFVTYQISPLLHCRISTVTETSRTSYVMKMMNNGNRIFYLGI
SCFLLFLGRVGSVSFCDGLCECYLSRISCFGVDFIKALNHTLPDEIANSTTSLQISYSETLRLDNDT
FKRFHTLDTLDIAYVTVTFLSEYVFEDLDGLQHLRLAYTGLKTIPSTELSHLSSSLAKLDLMFQKFS
NIPANSFVQLGNLREMTIEYNLYSLDVSANAFAGLANLSYLSLCNSGIRSLHDTTFMGLTSLQEFDL
SHNLIIAVPTALDALKSLIKLDLSYNHLLQNTSNLEFLVEMPALQTLQMGYCAISEMSPDAVHNLKA
SSLTVANFDGNPFSCTEYICSFVIWYVGMAELSTTTRSPFYIPFITLSPPPGKGPYRCETSGLTFEE
FQKESCLPPVDPLPTISYPVNYPTSLRGIAFGVICIVFLLVIVSVVIWKHRLKRNRGFHFGFNFQRR
ADYGAVQENDNEYMFDAYVSHHEDDRPFVQDEMLPRLEDENGFDLCVSFRNFRLGSNLLENVSSAQD
VSRAIIFVINERFMQNGQCRLELEMASRRMLEDDEENGRRLLVLIMMDVLAPELVNNTLRMLLNHVA
YLEWDPVAEERCWGQLVATLRAMVPMRNDQNEERIADELNADREGGNER

>LVA_007716_RA_Scaffold1935_prot
MEFINFGKILLFVSLLPFIRRMKCEYFFCGEFCNCHPDIHMIHCSGVDFIEALSHNYSDEITNNTKS
LEISYFDQSLRLDNDTLKRFYALEKLAIVHYGVTFISEYVFQGLYELQNIRLTFNSLKSIPIVQLSH
LNSSLEELHLLQQKFTNIANYSFIQFGNLREMTIKYNQYFLEISAYAFTGLANLTYLCLSENGIMRL
HSTTFKGLTNLQELDLSHNLLVTVPPALNALKTLIKLDLSYNHLLEYTGNFQFLAETLTLQTLQMGY
CAISTISPEAVDNLNASNLNVANFDGNPFNCTEGLCSFVICPPLGKGPYRCKTSGLSFKEFSNESCS
PTPRIFSSTVSYPVGSSTSWHGHVIIVTVSITIVAFVVATLSFIIWKFRLVKRYHHHFRIHYQRHAD
YRARQEEDNGYMFDAYVSHHEDDRPFVQDEMLPRLEDENGFDLCVSFRNFRLGSNLLENVSSAQDVS
RAIIFVINERFMQNGQCKLELEMASRRMLEDEEENGRRRLILIMKDVLAPDLMNNTLRMLLNHVAYL
EWDAVAEERCWGQLVATLHAMGPARNDPNEQEADIGENNDGEAPNEIQPI

>Scaffold6509_prot
PGTKITSFSTKGVCLKMTPIHVLVMLVMLSMLCVKPTTTTEIAYSSAADDGDYTCPVCSCPYDGTSV
VCSMDQLNTSYPFANINASNATSLFITQGGADEDRSLYLRDGMLGRAFPKLHSLEFSTCSIGLIEDA
AFEKMPYLQELTFYKNGLQNIPTALNSIANSLQKLEISYQRRTTSVDFNGMNDFCNLTSLDLSSNSI
LEIGRYSFSGLIKLQTLTLQNNNIRTINDGVFDPIENLQSLDLSLNKLHVIPSTMKSLIHLVELDLS
YNNISDLRNFDFIAFMPNLTSLKLQGNAINTIDNISLDVLYNSSNLVNIRLCGNPFRCNKALCDFMF
YANLYLSFSSYDLLDNTWGSSIKCTYSCGYPFRYLGKTFDSVYGDLCVRKLDLPTEVPVVIQKNRKS
VRVISTVLGVLSSGGVVFGLIVFFAFRRLRHLHRGFIFAGHGFVNFNNRRAPDIRFDALVYNHVRES
NFVDDRLRPRLEDPPNDFHLCLPLTRDFRLGAKKLNNLRESMIASRCAIFVISEAFVQDARCKQALE
VACEFLHRDDLGPAHLKQTGLILIIILDPVLLDELPETLRVLVDRLVTLEWENLNEERCWRRLERALE
QFREPDGI

>Scaffold11175_prot
SERECYHNLPKETAVLDLSGNTIIALYNSSFKGLPNLVVLELTNNNITTIEGGALLCLLNLKQLLLS
HNKLRKLQDNLFRNNNELDTLVLTNNQFFNNAMSAISPLKKLVILNMEWNSIKCPNFTGFESGNLSL
LNLNKNNIADINSQIFYPLENKSIGILSLNYNEVKILPQYAFCYLKNVRNFLLSANNIDKFSLMPFL
EMKSLINLTINDNNISHIISVTNATEIRLSIPKLRMLFLARNRLIRVIPSKAFDGLEELEYLYIYEC
QITHLHNNSFTGLNSLKLLNLARNKIREIHGRIFTSFSNLQTLQLSNNKLSLQSAVQITGLRTLQTL
FLDGNDIQLSTPLVWDLPSLSSLQLSNNGILLLSANSFLGLDNLTHLTLSKNQISAIRNHAFGGLNH
LQKLDLSAGGNIGSFASPFQNLQQLIEIDMSGTSFHPIQQIFLGLNSLRGLSLSRCSLTRDSLWDSV
KNASIFATVTQLEFLYLSWNSLNVMHGSFHPMSSLTTLKLDHCAISVLHPDIFRNLSSLEYLYIEY
NYIKVISLQHLLELHSLMALYAQNNDIKQINKEAFSEKKHFLTLNIANNGISWIEEGTVLPRRYLDI
SNNPLACVCKLMWFRMELDKRNLTLGKSNDTTCSQTSIPSLVGQPLLSFNPKHSCAPNIALYIGLSI
LVVCMLFWTVTTYQKRWWISYKCFHLKLWIIGYEEFQDGREHLDFTYDVNIIFDEDDEEWVKNILKP

GIQEHLPHLNRVLLGDRDLPLDMFYMDAVIYVIENSYKTVLVVSENAIRDHNFVTKLRQTVDHMNEE
ETEKALLVFKDDIRQGKLPYLVRLLMSKNKPYLLWHEDEYAQRLFWEKFTKNMLVNKKMNDLLP

>Scaffold126_prot
FSILFLIIFWQGGEAIGKYCSGDFINIMTTMTEFCLLLSIGSLLYQWTDIHAASCPQDLSLKAAFCS
SMGFTSVPTDLLPDLRKLRLDDNLITMIKNETFTRYPNLRALTLGQNNISCIEEDAFEPLKSLVSLL
ITDNHHLPAFKRSMFSSNSLSLLNAASSNLETFPGDVVELLTSSTILSLSSNRIRNINWTSSNPLNS
LYLDSNSFRQISNESFVLNNPLDTLDLSTNPLEFIEATVIASLNIKRIYLMNTHIALNQIRNFFHGI
SLSTYIEELNLSDTELHAIQAGLFTPLKGKSLAKVNIELNDINVIEDGGFDGMAGVNQLQLGWNNLK
LIKPNIFKGMIALRYLSLDKNRIVTLNEDAVPWNVSLIELNLASNEIISINSSAFHGLKTLRKLDLS
ENHKLQLIESDSFASCPFIAELDLSNTHIFHLHFPYLPFLTSLILQYSFCPDKLIRPGNLETKAPSL
STLNLIDNILGSEKLWDSSTNKSSFYGLQNLSRLELSSNPLLSIPPGIFKNLSNLQILRLDDCLLVV
LETGIFSDLSHLILLSMQSNHLRAISTGLFDKLQDLQYLFLPGNEFTYLDGNVFQYLSNLVFIDISK
NRISGLNHSTFRPLSNLRKLYLSDNPIVCNCDLKWFPVWLKGGNVELIDSFETICLDTVATLKQFRG
KQLITFNPTGDCDANIVLYSCLAVVGMVSIFTLGLIYYQRWWIRYRLFLLKLCFVGYEVIHDDADRE
EYQYDLAVMLHEADDEWVDQHLRPALIERLPDFNRIVCGDEELMLGMYYLDAVHYATERSFKTIFVI
SRAALQDQWFLMKFRTVLDHVNDVGTEKMVIVFVEDIGEDELPFLIRLFLSDHRPYLIWPGDERGQY
YFWEELTKGLTVNIKCNQLIPP

>Scaffold1439_prot
FLVFFSFSLPFFIIIIFSLLYVFYFSFFFSPSSSSSSLFFPHSCSFAFNITLGPSMVNLSGIRMFNL
LPITPTIFFILVFTLTFNHGKASGLSKSCEQDWNLKIAYCSNLHLSSVPTYLVPDLRELYFSGNLLG
RLRNDTFLNYPSLRVLKLSSNNLSVVDHGAFTPLKNLVELDIGYNTYLTTLKRSMFDSNSFSTLLAG
SVNMDSFGGDLLACFARDAIVNLQENSIRQINWTSYNAFFDVNLNQNKFRQLSDENFVVNSKINTLH
LSGNPVEYITPSFVSSLKVSVLYLRETRLAVSEFSNFFSGVKLSHYIQEIGLSDSSLHLIDPGFFSP
LKGKRLRTLDISLSKIGSVQNNSLTGLDGLTQLLLGFNYLGSVEPSYFNGMVALTKLSLEENNINVI
NSQNESWDLSLTHLNIARNQFREINSSAFQGLHTLQFLDLSGNIELRVIDKNTFANVPFLKMLDLSG
TSITDLHAIHHLPFLTSLILQSAYCPSHLIQPGNLGNETPSLAELNLVDAELFTSKLWDPNSKTSSF
LGLYRLSRLELSKNALFPLPVGLFKNLSNLNILRLDDCKIHELNIGVLEDLRNLSQLDLANNLFVFL
PRDVFDKIKNLKELILDGNQLSYLDSNLFKFNMDLTYLDISNNNLAGLNKSTFESLDRLRVLDLAYN
PLVCNCDLKWLHSWLKGSVEIVNSLDTTCLDTPATLAPFGGKQLITFDPSKDCAVNIALYCGLSVVV
VMLLLILGLAYYHRWWIGYQLFLLKLCFVGYNEIREDVPRGNFLYDIAIMLHEDDGEWVDEHLRPAL
AERLPDYDRIVCGDNELMIGMHYLDAVHYAVEKSFKTIFVISRTALRDQWYLLKFRTALEYINDVAT
EKVTLLFVEDIGHEELPFLIRLFLSDHRPYLVWSDNERGQHYLWEELVKVVTVNLRCNNLVPP

>Scaffold3741_prot
YSHHIAIPSGLFDGLYELQYLFLLGNKLTYLDVNVFKYLSRLVYLDASENGLSGLNRSTFEPLSSLT
EADLSHNPFVCNCDLKWLPSWMKGTSVELLESRDTTCLDSKATLEPFRGKQLITFDPTGDCDANIVL
CSSLTVVAMVVILTLGLIYYQRWWIRYRLFLLKLCFVGYEEIHDDADREEYQYDLAVMLHEADDEWV
DQHLRPALVERLPDFNRIVCGDEELMLGMYYLDAVHYATEQSFKTICVISRDALRDQWFLMKFRTVL
DHVNDVGTEKMIIVFVEDIAEEELPFLIRLFLSDHRPYLVWPDDERGQYYFWEELVKDLTINLRCNH
LVPPK
>Scaffold436_prot
IYIFCLFILSIIQGGNSVVNCPKQIQRIPCGITFIRWHSRCRSFFSYLILSLVSVLDSEFIPSPMRS
PRCRLSVTSDGLEADCSWLGLQSVPLELPGGVTELNIEGNGIQSLNRSSFSHLPLLFKLNLHRNKLA
SINEGTFDSLPLLQELSLSQNSLAELPTTIFQKNQKLELVDFTQNLFSSVPILPLRGATNLESVILK
NNRIKSVNFTGFRSQDVEVIDLTGNNITCVRENHFSPFKNISTRSFRLSYNKLTSLPDIVFGKLHMT
KDLVLSHNGLQNFSLYPFLGMESLINLSLSSNPLESVYPFNETTPRADLPPLEHVILSGNHLFTLPS
RAFVGMGSVTRLDLGKGKLKSIENDSFAGMFALEFLDLSSNRLATLRREIFEPMTRLHTLILAKNKF
EYLPTTVFQDLRSLKVLDFTKNKIANIGTTWDIPSLLTLDLTSNLIFRINDGNFYGLSNLTELTISS
NPINLIKNNAFSGVENLQTLTMQSLTTIGSGSSRPFRNLVQLISLDLSGSNMNPSTIFFKGLVFLKT
LKLRETLLTPLKLWDNLRNASVLDTFFGLEYLSLSSNTLNGLIPGTFQNLTSLTRLLLEKCGIFQLP
AGLFENLASLRYLYLTRNNIKVLSASHFLGLHSLGTLLLENNAIKEIEVDLFKHTPKISYVYFPHNL
ISVVHEGTVLPTVMLDISNNPLSCVCEVQWFLSYLDNPTNNLILVNPNDTICSPSSIQRFIGKRLLS
SRPDSSDCGPDIIILACAPVFVLGLLVVLAATYYYRWKINYKLFHLKLAVLGFNQFEDAREHQDFRR
DLNVIYHDDDEEWVERVFRPGIDEALPDFQRIAYGDRDLAFGMFYMDAIIDLMENSYKVAFIVTNTA
IFDHSFINKFHMAVDHMNEVGFEKLVLIFVEDIPDNHLPYLMRLFLSKNKPYFLWSDNVDWQALFWA
KFAKLMKANKQINDTLP

>Scaffold5772_prot
LNKTDNKMCNICFLWVVIVVVPTLVCGNSRQQNLTGIPCSYERSDEGLRIKCSHRSLTAVPGNLTKD
ATIFELNDNLLSKLSNRSFSGLPNLVSLNLQSNIVGDNMEKGVFCSLPKLNAINLANNGITSVPSGL
FSKNRRLQKVDLANNKLKSFPIDVLESLDTLQFVDVTDNLITNLHFQGLKTKNVSLIFNANRLSVLH
EDDFLPLNDTRIEFLSFAANNLSSLQSKIFSHLSGVRKLALAANHFRNFSLLPFIGMTSLWKLTMEN
NGISTILPLASSFNESHLLPPVKVLYLEGNNMYTMPSGAFRGLNKLTELRLQQSRIKKLQNDTFEGL
DSLEILDLTGNPISYVASDMFTSFCPKLRSLILPTNHLSQLNPTQFAGIRTLGRLNVARGLIRYIVI
RPTGWSLPSLQSLDISNNRISRLNKHSFYGMTNLTVLNISKNPINILENSIFASNDKLQILRLEYLK
GFGSVWTPFTNLQNLNSLHLSHTPITQLTYKVFTGISNLHTLKMDDCALSFVSFWDPKMNVSVLSNL
STLKELSLKDNNLNDLLPGTFRGLQNLEGLEMQRSNIKSLHEGIFMNLTTLVDLKLDRNSIKELTSQ
HFKELTSLKIISIKGNEIKEIPVDLFSGNPNLNQLTISHNHLTTIKEGTFLPKIALDVSYNPFGCFC
DLKWFVDWINMKTVDTLNPDQTNCSQASLAKFKNKPILKFDPSRVCGPKVVVYIISTFVIVTCTVMI
IVAYQRRWLINYKCFHFKLLFIGKTGDRDGRNRLDYENDVNLVFDDDDEKWVREVLKPGIEERLLNF
DRIVCGDDDLPLGMFYIDAITEVIENSFKTILIVSNRAVDNHGFISKLRLAVDHMNEVQLEKTILVF
KEDIPDGHLPYLVRLFLSKNKPHLRWSEDEYGQRLMWEKLVQELEWNKKMNDVLPI
>Scaffold8782_prot
FKGEIFSFWLRGQRASVMHFFNLFWLTCILLLASGAVKDSGGADGEDPNREPCDYRVTPDGLQAICT
HRNLTEVPTDIVEDIAMLDLSHNQLTILRNASFNRLPHLRLLSLQSNEMTSIEMGVFDSLPELYQIT
FSNNNLTCLPTNVFSQNRKLQIVYLSVNQFVNFPGNAINHIKSLTLLNMEKNSLSHVDFTGFESMNM
TLLSLKYNNLSSFSEDDFLPLRNANIKQFTMTKNFLTSLPYGLFRHLAGVQEMILTNNQIRNFSLGS
FLGMSSLKTLRLASNMISAIEPLAPLPNQTNVIPSLTLLDIQGNRIPSLPSRSFWGLANLIRLDIHQ
SRIKTLQSDSFEGLASLEILDLTGNHLKYVNKETFLACPRLQSLILQSNPFTGLTPAQFADLVSLES
LNLARCQINKLYLQQGGWNLPNLKFLDISYNRLYRLEKYSFYGMANLITLNISHNQLATIENGAFVS
LDHLQSLGLSGCPLGQIHSPFANLNELSILDMSYTSVELMYELFTGLNNLTRLNMRGSGIDKESLWD
SPTNPPVLSALSTLERLYLKGNKLDGLIPGTFRGLESLQHLELDNSDITFLHEDIFVNLTSLQTLTF
DGNQIGELSPRHLSNLTSLYGISIQRNEIKVLASDVFTNNPHLSYLYISHNHLTTIQEGTVLPRKTL
DVSNNPFTCNCEFGWFVNWLDQDQVSIIHPEQTNCSTVSPAPFKNRPILSFDPREVCGPKVWVYVIT
TFSIVTCLMVCIVAYQRRWLINYRLFHFKILLLGRRDGHDGRERLDYEYDINIAFDDDDEKWVRTVL
KPGLEERLPTFDRIVCGDDDLPLGMFYIDAITEVVEHSYKSVLIVSNRSVDNHAFINKLRLAVDHMN
EVELEKIILIFKEDIPDGRLPYLVRLFLSKNKPYFRWSEDKYGQKLMWGKLMRELRYNKKMNDLLPI
>Scaffold8774_prot
RQNLIVSILPGMFWKLRALEILDLGTCGITEIDSDAFHGLASLHTLILENNEIQQLPHDLLFTLKGL
QLYNLKGNKIYYLDNAIFANTTNLTEVVISENQFAALNQSTFLPLYQTLTSLDISQNQLDCNCKIAW
LLDWLQSSSHLKLVAESKTICSMASLEKLRGKPLLTFDPKHYCGPDIGLLCSLPLAIVGVFAMMFAM
HRYRWLLSYKLFLLKLAVVGYQEVVDARNHGDFEYDLNIMFLEDDEHWVQEHLRPVLEERLPNFIRI
VFGDEDLVIGMHYFDAVFHAVEKSFKTVLLFSRAAFQDNWFMIKFRVAFEQVNDARMENVVVIFIED
IPDAELPYLVRLYLSERRTYLWWVEDDRHQEYFWNELILTLQSDNMRWNNMIPL
>Scaffold4622_prot
VKLGDCIFVGIERKAELTSGSGNQSSRGEEAKPTFLINALRLLSHFCLNNLEGIDEGVSFDVSFDVN
QSKLFAYTMAIKCLLLLIFLSDVVQSIDYNGRRSYDEDDELSDYDEWSFVNWPTSSPSAMPMDATDV
CDFQKWQKFVNCSERNLTAVPYNIPADTQILHLGFNLLFRLRNHSFTNLTQLRELYVESAELTNIES
SVFVDLGELEILDLHGNSFSDLPGDLFVNNSNLIELNLGNNRFDSIPCETLMPLTRLRKLLLFLNNI
NWPYCEHFPNLASGAIVSLSRNDIPVMRRGNFQFLENCTLSSLYLGGNRITAIDYRAVVSLKTQALD
MSYNPLSEIGIASLFYGFRMNDMLRSIELRSSLINSNDPSTFIFLKNKTIDYFGVSSNKFTSIPLWS
NFLNRVRNLSLADNSIFRISNRVFLEMTALEEINLENNQILRLETLTNETRWSATQLRTFKLAQNRL
DNIPDNVFLGLENLTSLDLHSNPITYLTVESLNGLQQLETLDLSETRLTSIASGTFQFVPNLKILNF
RNADLRQANPRYFLIDIPNLESIDLSGNNLQTLDLWNGYFQVSIFSGNTRLKEIRLDNNPFIVDIPA
RTFENLTSLSILALNVCGLKSIQANIFTTLVNLTSLSLNMNTISQLPTNLLWNLKSLETFSAAENSF
TTLDSSIFKHTLSLRYISLPNNAITFIAGDSMSHLSELRTFDLHGNPIACTCALEPFSDWLKVTNAS
LIGEERTLCSPSSFESVVGKSILDFEPTTFCGPNIALLVSIPALAVVLIVSVALSFQYRYWIRYKLF
MLKLSLFGYRRYEERDVDDYEYHINVMFAADDQPWVDDYLKPIIMGHFPEKDIHNDMVFGDEDLIPG
MYYLDAVHYSVIHSYKTLLLLSYAAIRDEWFTTKFRLALEHVNETQVEKVVIIFLEHIDDKDLPFLV
RVFLSKNKPYLRWTDDEAGREYFKESLVKSVKVNMRCDNTIP

Saccoglossus kowalevskii TLRs

>Sakowv30039278m
MTVMNASICLLIVHIYMFGIVNLYMLPNPEPICKYKELTVDCSHKTLMTIPHGLPRNTKRLVLCHNS
IARLTAISFKGLPKLEFLDLSYNSLSHIDDAAFASMPRLKELLLGHNEIGKSLPPATFRMLTELTRL
DLSHNLIDSSKTQLLFGLNKLSYLNLAQNKLKSVNLGQHLNLTDLKMLNLSRNIIDGLRRDDFAGLN
GSSLNTLDISCNNITVIEDDVFQGCTIKSFNISESFAKNSSVLVKSLVGNLKGKHLQELKLQNLNLT
RLNNGTFEGWDTETLSVLDLSLNKITVIENQDTFHGLGKLKYLNLDANGLTGFNNKIFLELKSLEQL
SLNQNNITILKKNMFSEGIPLPITYLYLRNSNINKIETGAFSGFDRLENLKMYNNSIDGTLNNSCFQ
GLVNIKLLDLGKNKHIHLSPGTFSIMPTLKYLYLNLNLLTIDPSKLSPFTNLTLLKTLDLSNNNMDT
VPFFDDLKSLETLYLQHNNLVLLWKKTHSGGPVFMLRNLTKLQNLHLGWNGLDNIPPMAFQDLHSLK
YVSLIYNKLNRLPDELFNNTRKLQNLYLHNNMFTLVNQSNLAYVLPSLKVLSMNFNPFSCSCDLEWF
RLWIGKTDIDIVDVEKYECFSPPSMSKQPVLSYMPSRWTCDGIPPTWFWGIIIAIAVVVMILIATGI
RYRWHIKFRFLLIKARFRNYTPLEGQDAEYRYDAFVSHSSKDEEWIIQKLLPELEENGSPKFKMCYH
ERDFTGGKAIVDNIIECIAESRKTICVISEHFLESEWCKYEQTMALHRLFDDHKDVLILVLLKKVPD
KKLTKYEKMHKLMKRKTYLEWPGNSPEKQQVFWQKLRNALMCGVKPSKNIDL

>Sakowv30024275m
MVDSLPGYVCFCALICVSFGVLPPWKVYNGTHIEYTGAYADLTALPDSLTHLKYINTANVSWNVSNE
TFSQCNPKCLPLQKLDLGGNAITSVSRHAFSHLSSLLNLSLAQNRHLKQYDINCSWSLELNVDVFAG
LHNLMYLDLTLIGMRDLNPEMLRPLKKLETLAIGKNCLAELQDETFNGLQELKVLDLSYTTIKSLPS
MVFSKLNKLETLLLYRTRLMNIPNVALAPLVNLRYLIISGYFTTIQFGEQFKNITRLQMVSLRTYYY
NNTEEEHTEMNITNTSFEHLQTEAFEIHQDQGVGANGVKNLLRYPPDTKNLSLNWVHGLSSRSLNRT
YFNVSKSRVVDFVSLDLSHTNLAGIKNETFIDLPNLQVLNLSYNILSFFDVYVHAFKGLEKLEILDL
SSNSLIWVPSLKDFKNGSLKVLYLNDNELDRRIFSNVFSIVPTLRELHLRRTRLGNSYLYWLYNLTN
LRVLDLSENYFKNFETLTTALSNLTLLEELNLARNPLQFLNKKVYLKKLKKLKSLNLSGTFFDFYWL
IAFNLTSLEELYLENLENGDFFDITWQKMNLSLENLKIFSLAGSLVRTLSPQFFSSMPALKHLSLQA
NGLDFLNKDMLKNLKHLEYLDLSLNSITKITRFDFPDSLVTLILSYNHLSIIEREELNSGELLNLKT
LVLSQNSLECSCPARWFRNWIHSNKMVNLVNYDKYICLSPSGGRAFFTAFNFDNLKCESLSYIHLAI
SLSCVAVIIFAVVCIAVYNRWYIRYACFLLRLKTRGYRELVDLDEKTYDAFISYNSADQNWVLRNLV
PCLESDKYRFNICVDYKNFIPGKFIVDNIMDSIQESRKTILVLSENFVNSEWCYFEMNMALHRLFDD
GRDVVVMILLEPIVGKKLPRILRKVFTKKTYIEWPQDHSTTAKELFWAKLENALKAPSRVDRVHKV

>Sakowv30024236m
MSLWSLILLHFLFCDVLLGDSIPRWKIYNGTYLKYTTKWPSAAVNSTVLKTIPRDANITHLYIVDSG
LVNITQESFKGLIHLKELDLSHNPVMSMENNSFADQGYLLNLSLARNAIFLESGGDCSRCSFNPHPN
VFKGLTNLTRLDLTLTGVCNVTKELFTHLRSLQSLLLGSNCYNEFTEDTFDHLRNLRLLNLSATLIE
SLPSGIFSKLTNLRALYISPSKLKVIPNDALAPVPLNKLYIGGRFTHVEFDDNFSNKILQTVAINAC
MTSNTPFAHTCGLLNITQTSFRHLTTKRFIHHQLLGLDSVGIKTLFAYPPRATYVMVNYGHGLAGKF
LTPDLFKESADIMTKMETFDLGHLNIIGIKNNTFLNFSNLRILTLANNDLISINIESNAFMGLSKLE
KLYLDSNSLSTVPTLQGMSSLKKLHLSGNQLNPVIKNSTFKDVPNLEELSLANSHIGDSTLNGLVNL
SSLISLYLSGNNLNYLPKVVHSLSTMTKLEILELSENAFIRTPENVNISLSSLKHLTKVLLGGYAVD
LKLLINVTSLKELHLKNPPYFDIVNSWLKYNLCLPKLQILHIIYSSMVLNSNFFHTMPNLQELHLPN
NRIDSLEKNIFSSLNDLRLLDLSRNDIKLVSTDAWNQPKLKTLLLTDNKISIVSEELIGRKYLPSLR
TFDVSKNSLDCTCDLIWFRNWLYIDCTVDVIDYYLYVCATAGGRDVTLLQQFDSDTLQCASYGHISL
IIALCCIAVIVFIIVCVAVYNRWYIRYACFLLRLKTKGYRELVDLEEKTYDAFISYNSADQNWVLRN
LVPCLESDEYKFNICVDYKNFIPGKCIIDNIMDSIQESRKTILVLSENFVNSEWCYFEMNMALHRLF
DDGRDVVVMILLEPIVGNKLPRILRKVFTKKTYIEWPQDDSTTAKKLFWAKLENALKAPSRAITHIQ
SS

>Sakowv30021123m
MITSTKATTHKMNISLLKSVLIIMMADITIQLECPRGCTCDVNESNIYQFDNILCGWGSFSARGDTT
SIAATTKLSMHCDSKPLINAVQFTLHHLQNKSVLTEVEFTFCPVPILTNRSIPFLPNLRILNITNSD
LNSITDNALQNLPSLSVINLTNNKLSVIPDTIPGHSHNTTVASKIQELYLDGNSLQNICNQTFKEFT
NLKVLTMAMTGQHETLPEGLFMNLHHLKTLDLSRNTIPHISPTLFDNTTDLEVLLLYSNALQVISTG
TFNNLRKLRILDLSLNIIHTLSVDVFNGMESLQHLNLRYNMFLDLPSNLFHGLHNLQHIDLSEIEFS
WSRVVLPDDLFIDIPMLKTLDISKANITSLRNMTFAGSVALEEINFSTNHIKLIPKDAVLFSHAINL
RTVDFSYNEISSIESHAFQDSQLRSIDLSHNNITIIQKHTFYKLSQLNTLDLSHNYIYFIHAVGLFQ

LPNLKELRLNNNLLTNFQFFLYARRGALVQPLPALPDFLHFDDPMLVYMANNPFNCDCQMFAKLFNT
SGHARYEHILWPGTDQLINQRFMPDPSSLVCYRPMRLRGRSIMSLKKNEYWCETTSFCPDNCTCYMV
PVENSLIVNCSYRGFTEIPDNLWFGISYLLLEGNYITRITNNKLNHFTRLWELYLNNNAIQIIDDKA
FHNLTSLITLDLSYNLITKITNEFTTMSNLENLYLNFNNIDYISTNSFVSGGRLKELMLDNNMLESI
DVDMFNNTNSLTMLTLHNNLYQCDCQTPWVDFKHWLQQPSINAIVAFQYNITCSTNNTLEDVPILQA
NENDLNCTTSTDSQVINVIQNRIIGAVLGLAVIAMIIGVTVFKFRNIIRVIIYNRTGWRLHHNKDYE
DAKIYDAFLSFSSEDLPWVKNSLLKKLENHVPPYKICIHHRDFIIGECIATNILDAVEKSRRIIVIL
SNNFLRSEWCAYEFNQAHLQVVRDKSCRLIVILMEKIPQQDIDKEIKMYLKTNTYLEMNDPMFWEKL
YYVMPDVHGRKDENADEEQLVENI

>Sakowv30033406m
MMSLAKVLVFMLILNTRLRVTRSCIPATNVTCPRLCECTLCGKALYLNVYCWNVKSASMLNDTVQLM
PLETDTVGIGWNKLSQIFNRTFLRLSNLTRLFVDHSKLDGNLIQLGAFHGLNKLTHLNMGNNPRPGI
IQLHSEWLKPLPLLSILSFEYSNLNYIPEDVFANNSKLYLIKLYNNNLKSIAASTFHNLPELERLFL
QHNSLTSLPADMFNGTREVKELNIAHNQLTTISPDTGLQNLASLYKLHVYGNPLDCGCDLIWFRNWI
DTTDVVWNINNVNCSDGKNILKFNPERLQCGFPVLITVMSTLSVVILCTGIVLVILNRWQLRYGIFL
CRRACRREYAEIERCNDEFEYDVFLSHSSKDVDWVTNVLRPTLENPPYNYKLCLDYRDFIVGDTIAD
NIIDAVQKSRKTAFILTKSFIESEWCYFELEMVRQQMFDEHRDLAILIMKENVSTGDMPGLLKYLMR
KGNYIEWSENKYGNKLFWNKLDNALNYNKENMV

>Sakowv30024277m
MLSEWAFSVECQILEVSSRPCKVYNLTYLDCRFQNIIDVPSDLAKNLTSLDLLVNNITSIRKDSFLA
LSQLVYLELSRNNIVEIEPYSFSRLERLEYLGLLRTGFAFKGGFHITTQTFHGLGQLRTLDLTLAGL
NDVSKEYFFNTPNLRTLRLSLNFIYVLPEDTFEYVSKLELLDLGGIFITTIAPWTFATLADLRELYL
NWTRITTMSLDPISPLLNLRMLFIEGPFTIVDFNSTLTNLRSLSEVETNVCNFTDLNNLENAYCYDT
NFSHAPFQELHTNSIILNEVVIEDENINKVLAGPFSPSTRELYLELVTGLSIKHINGEILNGVLNSS
IEIIDLSRNDIVGIEPGTFHNMTNIKHLNLSSNAFCLLDKPAFEGLENLKILDLTSSHLFSVPYLRP
LTHLQVFNMSFNSLKNPINYHAFREAPNLEVLILKHTSLSDLAISNGCLHNLTNLQVLDLSENELIY
IPRIINENILTMPNLKKIDFSNNYHAHFMNNYRATLAPLTHLESADFSAVYPELFIFSNVTSLKELT
MNNNNGQAGQWGGNFNKDWVKYQIYLPRLEFLSLSNSAIRVLSLDVVSSIPAIKHLDLTHNLLKSFD
RSVFSTLSYLVTLDLSSNLIAEAINTWNQTSLHTILLHANKLTVIDEDWISEEYLPSLKTLDVSSNS
LKCNCDLVWFRMWIETDANVNLIDYGTYTCTSPTNNSKLIHLKDFNPNRLQCTSPFPLILTIVGSIL
FAMLFILIVISVYHRWYIRYGCFLLRLKVRGYREITDETEMQKSFDAFVSYNSKDLQWVLHTLVPTL
ETGDEPTFRLCTDYRHFIAGKSIVDNILDSILDSRKTILVLTPNFVTSEWCHFELEMAQHRLFEDDR
DVLIIIVLEDIPDKILPRRLRKLFCKKTFIKWPQEEEGRELFWARLKDALQKPSLVDRSVDV

>Sakowv30033399m
MTWKFFWTPILNVFFTILLSSRGETRCVSATNITCPEGFSCEFCAFSYESWGNLKLVYNLKSVSDLK
ELVSRVPSETVLLYLQSAGISGIKNGTFSKLGRLLKLYLHGNNLVWNLVELDAFQGLSSLQLLDLSV
QRGINQVRREWFLPLSKLVKIQLESNIIKSLPGGTFANNVQLETILLHQNKLTELPDDIMRDLPKLK
RAMFQTNEIKTLPGNLLGGSTQVTELNFAHNRLSTISSEIGFQKDTNLTSLHLYNNPIECNCDLIWF
RNWVSTVNVLVSVNDTMCLSGYRIVDFNPDTLECGFPYIKIIVLSVSGLLLISVLIAMGKNRWRIRY
SLHLVKARLLGYQPLVDGDDDEYEYDVFLSHSSKDEEWVSKVLHPTLENPPYNYKLCLDYRDFIVGE
TIADNIIDAVQKSRKTAFILTKSFVESEWCYFELEMVRQQMFDEHRDLAILIMKENVPTGDMPGLLK
YLMRKGNHIEWSDNKHGELLFWSKLDSALKCIILSLKNEKQIMKDCNPKVQLFCETLEKIYRKGLKQ
PGSIFGLIRRDYWCWIEALPHYTWNDKINPLFSLALETCQNSSKVRTHQGKGRCFLRLSLMKKIINV
PISQLLKNPKLTEYWYDPSTSIIANELLKESLMSMLFLVTDIDFELNIKNASFLDETWQIPVYKHYE
LVPCRDLGIIVRHIKNRIMLADVKPGSVAGEDDKLEPGDIIDELFGESMYGASKGKISKLMCDHEGW
PIYISVVKCHLLNGKIFSPIHERLAVLRKEYANFKEPKEREPIKKKIPSHAQLPRDALDEVPISSPE
GSAGYRVKYIDKVHVGKEGGVNLIEGSINSVIKQKEINPKDVWLELAETDIIIKNLETRNTELKHSY
TEISSCGRRTDALTYFAYIAGDTTCTISKSFVCYVFKSQTEEEHDVSRLPWELVYTQTCDGNKRDFG
SFHSSNVQDLIEISHEEYEAPKIPVELPTFTSHDTPHYWPVFGKEIRKKHFLLEDFTFLNHGAFGAA
LKDAVDAKHQWQYYIERQPVRFMDRDVLPHLVCITRKLAQFVGCDKADIVLVSNATTAMNSVIKSIK
FKPGDIIYCLNTTYGAVKKLLKFISEETGAVIQEETLEFPLSEESEIINKVKATLSPGTRLAVFDHI
PSNTPFIMPIKELVEICHARDVPVLVDGAHSLGSLSLNIVDIGADYYVTNAHKWFCAPKGCAFLYVR
KELQQTVRPLVVSHGFGSGFSAEYMWPGLIDFTSFLSLYTVLDFWNSVGPDKIRHYIHSLAKQAAEL
LLDKWKTKLIAPMHMFGSMVLVQLPSDLHKNKICNYDLAETIQNKLFHQFKIEVPIKAIQNELYVRI
SAHIYNDICEYEVLRDAVTQLTTN

>Sakowv30024388m
MDLKTFLFITVFLFNYVSCGSTISPWKVYNQSFVEINYSVDQVPSLLSNITHLSLTKLDLANITAGT
FNKLSSLEYLKVYLLSNSEHVHIEPKSFTMLVRLQYLYLSGSDVTAPACNRSIQPSFSPDTFHGLNG
LKVLSLTGMGITFLCEHLFKGVSNLHILNLQKNPIYNISKDAFNHLTKLQTLDLSHTFITTLNPETF
SQLQNLTTLHMVPSNLKEIPIALIKLTNLEVLFLGGNFSEIHFNDDFEKLKKLQEVKMVSIFSNDSL
CGEICRDMSLPYNAFWSLKTKHLTLHDGLPTCFFKVSRSMGHKFFKFYSDITEGLSLSGLDAPLNQS
FHSTLTNLNLSGSRVDHVKDYVFKHLNNLRTLNLSHNFITYFDEHAFVGLASLTRLDLSYNKLTLRG
LSALKTFSNSSYKLEELFLSNNTHLVTHNTHMLEDAWNLFLIPTLKKIVLHSINLKGDAFKFLIKVL
HKLPQLEHLNLYGNDLNTIPTKLISSYPLNSLKILDISYNCLAVLDQSEYEHGLSVLPLLQYVDLSG
RRVRLSLFKNATSIQQLILNNRRPMCPDLQKNYKWKNDFNVQWENSLLTFPNLIKLEISYSLITQLS
KIVFDGISNVKYLNLNNNKFDHLDLSLFSRLQYLIHLDLSKNEIHAINGSWRMPKLKKLMLSNNGLS
SIPHFMLSSEYLPSLVELDLSWNVLRCDCLLSWFRNWAQTDYSVKLVNFHKYQCANAHDNFEVDGDV
RNFDPAVLPCESFLHVHLSVSLSCGFAFLLTVSTVCFYYRYNIKYGWYVCRIKMKGYKPITEDRKYD
TFVYYNNADSDWVNDEFLPKFGEGDHPKFNICTSEKDFSSGYKVTNILDAIQESRKTILLLTSNFVN
CELCIYGMFLALQELFQEGRDVIILVLLKPIPNGEMPLELRKLFCNKPIIKWPKTDDPVARNLFWVK
LEDALKSPTIVDHVHTSVIR

>Sakowv30000609m
MPSITTVLNLEDNSITTDGFKFPKLPKLHYLTLAGNPIDDIKRTSFKTIPNLKTLSMYFTEIRHIEE
GAFSELKMLQSLTLSNIIIQHIPSKTFQGLERLEFLDLSSTYILESIAVDAFSDLHHLKYLDLHQNA
LTVIDTAVFQHLSTLQYLDLSNNKLSEIPDNICRYLPTLQTLVLSNNNKLSSFAIRETCANLTNIQL
DSTNITIVNQEDFDAVSGHTLDGVSFYQTPLQEIKVGTFAGFQINRFSFGHKNSDFVNVLRGLTDCN
MNQLAISGLEDNFPSLSTSTFHVAMETTLGETLQQLTISFNHIKAVEDFAFIAFPNLHELNLTLNHI
KTLGKDAFTNLVFLQTLNLKSNFISQIPDGIFDNLRNLRYLNLYDNQLAFLNKGVFDNLLKLQEFVF
GGQQVSMIDANPTVFLHLSNLHTISITENIINDELKLPMSSLTKLKRLNFKKISALQNDILHGCENG
TSLEDIEADESLWFVDHIPSINDCLLSVKRMSISGVDLMRGWKVCFWDLRPGIYLMQNLEELILSHD
QISDLGDNCFELMNKLIRLNLSNNKIQMLRSGLWKGLERLDMLDLRHNAITRIEQSAFQTLPSLKQL
YLGDNPFVCDCDLRWFQKQLSTNIWQLRPTEYQNMIYWLQDYQCKLPETLHGTYVTDYKPSDFACSL
MAVAGLIAASTMTLFAITVVVLRRYRYCIRYRWFRFKLRYFGYRALEGEDEEYLYDAYITHSEDIQD
IDWICDHFMPRLENEFNFRITFRHRDYIGGTNRIENSRESLQRSRHTVFVMTEKILESGIFMFELQF
AYQKLIEDKKDLIILVELEEIPNAELPDLLHLLFCSKDKIKWTENEQGQMLFWEKIRHQIQSDNRVD
ARK*

>Sakowv30006552m
MYRLLLLLSAAILNTAGCRGNIMCHCDYKNMLLVDCGGNSLTSVPDDVPNKESLTSLWLDKNNFRCV
QQDMFKGFQKLRNLWLDSSNVHELEVRAFWEIKGLHQLYLRDNNILNIDEDNFSGLEQLTSLDLRNN
PVQSIQNSAFKDLVNLNALNFSDCNLTTLHSGILRGLTKLEVLKFERCNISAIMNETFDALINLKIL
SLHGNQLRNIDGKLFHSLTIQTLDVSCNLLRGAIGNLCNNVQSVERLNYEKNLFTTIKFDESYSKCR
NLTYLNIANVANSISVMGNTFQGLKTINITELDFHGNKMKDLDPDALTSFENPILLNLGSTNIRTDF
TRTFETLLTKNKLKTHSLLLNDISHLSNILLQSHHNIVQNVWLRDLDLSDNQIPRLVERMFQWCPLI
KKLNLMSNSITNVAVNTFLGLEYLTELDLSDNYIWNIKADTFKNSQLIELNLSNNILVFVGFEGLKK
LKKLKLSDQGKVRIVKEQFIKLESLEELDISYNFWGSSFQLPLCNLTSLKKLNISMTTIENDMRNFS
IDDMSETCSTFVSELTHLDFSMSYVDNMHQIERYFYKNRKALINLKTLNISSLLHAESYKYFPWNIF
TELINLKELQLQNNHIGNHLLPRSDPFSKLTNLQTLDASGNRITNIAAWKGLLNIQKLGLHDNWFDV
VETETFSDMKNLESLYLHGSQFDCGCTIDRFHRFLKKHTTIWTSEAAAYDGRPFWFQNYNCSEPKMT
YVIDAYSSPWKCDKILIVLVTFAIITACMGLSFLLYKFCRYEIRYIWFVVRLKLNCVRHAEHTNLIN
LDEYDYDVHISYNITDENWVIDHLLPFLENHLQIRVHINDRDMRGGVNRLSYASNLILKRTRKIMFV
LSDNFVQDKYCMLELQLAFEKLFNDHENIMVFANLERINKNLQPLMLRLPICKKRQNKWYRYNEITR
DAFWNDLKESIAENGSVNHTVQLL
