# Supplementary material for: Characterization of the TLR Family in Branchiostoma lanceolatum and Discovery of a Novel TLR22-Like Involved in dsRNA Recognition in Amphioxus
Source: Front Immunol. 2018 Nov 2;9:2525. doi: 10.3389/fimmu.2018.02525 (PMC6224433; doi:10.3389/fimmu.2018.02525)
Supplement: Supplementary Data 2 — Identified DNA and putative protein sequences of TLRs in B. lanceolatum. The TIR domain of each TLR is highlighted in yellow. [file Data_Sheet_2.PDF]

# *Supplementary Data 2*

# Characterization of the TLR family in *Branchiostoma lanceolatum* and discovery of a novel TLR22-like involved in dsRNA recognition in amphioxus

**Jie Ji[1], David Ramos-Vicente[2,3], Enrique Navas-Pérez[4], Carlos Herrera-Úbeda[4], José Miguel Lizcano[5], Jordi Garcia-Fernàndez[4], Hector Escrivà[6], Àlex Bayés[2,3] and Nerea Roher[1*]**

*** Correspondence:** Corresponding Author: nerea.roher@uab.cat.

## DNA sequences

>BlTLR22
ATGGAAAACCCACCCAAGTCAACATCAACTTGTATCTACAGTTTACTGTGTCTGTGCTTGTTTCTACTGTCGGTCAAG
GGTGACCACGTAGCGAACCCTTATCAGTGTCAAGAGTGGACCACCCTGCACATTACATGTACCAAACTACAACTCAGC
AAAGTGCCAGACAATATTCCCCCATCTACTCTTCATTTAGACCTACATGACAACAGCATCACAGAGCTACAGCAAGAG
GACTTTAAGACATTGATCAACCTTCAGTACCTAGATCTGAGGTGGAACAAGATAGACCACATCGAAAATGCAACGTTT
GCTCCACTAGCCAACTTGAAGACACTAAACGTGTCTGGAAACAAAATCCATGTTTCTCTGCTGCCTCAATTAGTGGAT
TTTCTACCTTCTCTTGAACACCTTGAGATATCCGTCAACTGGAAATGGGACGATCCAGTCATGCTGGGGAATATGACA
AGCTTTAAAGGCTTGGGAAACCTGACTTCTTTGAACCTGGGAGGAAATGACATTGTTGACGTACAAGAGAACTCCTTT
GATGGACTGGACAAGCTACAGAGTCTCAATCTTAGGGACAATCTCATTTCAAACATCAACGAAGCATCCTTCTCCCCG
CTCAAAGAATTAGAACACTTGGTTCTCTCTAACAATTATCTTACCGACGACGTCCTACAAGTTGATAAACTTTGGTCA
CCAGTGGTGAAGCTGACATCTCTTTACTTGTCTGAAAACTTGCTGTCTTTCGCCCGCTTTCCGTCAGTGTTTCAAAAC
TTCTCCCTCCTCCACACCCTGGACCTCTCCAGAAATCAGCTAATAAACCTGACTACAGATGATTTTGCGTCACTGTTA
TTTACTCCACTGCAAATCCTTCAGTTAGAACGGAACTCTATCAGTCACATCGACCAAGGGTTGCTGGCATCTTTGGCG
AACCTCAAGTCTCTAAAACTGCAGTCCAATCCCATCCTGTTTTCCCAGCTGAAAGATAAACTGGTCGGACTACAGATT
GAAGAGCTGACACTGGGAGGAAGCCCTGATTTAGACATCATCCGTAGTGATACGTTTCCTTCACTTCCTTCTCTAAAA
CACCTAACAATGAGTCTTCTTTATGACTGGAACCCTTCAATCAAATCAAGCAGACTTATGGGAGGAAGCTTCCTCAAC
CTGCCAAATCTGACACAACTCAACTTGGAAGACTATTCCATCAGCTCAGTTGAACCATACACTTTTACTGGCTTGGAA
TATTTAGAAAGGCTAGAGTTGGGAGAAAATAACATAGCAGACTTCCCCACGCATGCCTTTGATGGTTTGTCATCACTC
ACACATCTAGACCTGGGCCACAACAGCCTCACAGCAGTCAAGTCACACTACTTTCACAGCCTGAAGAATCTGGTTTGG
CTGAACCTACAGAACAATGACATCTACCTCATTGAAGAAACAGCCTTCAAAGATCTTGAAAGCCTTCAGTTTCTTATC
TTGACGTCGAACCATCTCACCACAGTGGCAGGCTTACAGCTGGGTCTTTCTAACTTACGACACCTGGACTTGGAAAGA
AACAACTTCACGTCTATTAAAACAGGTTCCTTCAGCAGACTGGAGAGCCTAACACACCTGACTCTCGCTCATAACTGG
ATCAGAAAAATAGAGAAGGAAGCTTTCTCTGAACTCGCAAGGTTAAAGCGGCTAAATCTGGCGGATAACAGACTTACT
AATCTGACGTCCTGGGCATTCGATGGGCTGTCGGAATTAGAGGAAATAAAGTTACAACATAACCTGATTGTGGTGGTT
GAATCACACGCTTTCTACGGCTTAGAACAGATGACAAAACTCAACCTGAAAGGGTTCAGCATTGCAACAATCCCTGAC
AATGCCTTCATGGGTCTACACAACTTAACCGTACTGGACCTAAGCCTTAACCAAATCAAGACATTTGGGAAGAAAGCT
TTCAACGGTTTAGATAACCTAAGAGTTTTACAGCTGCAGAAAAATGAGATAACCTTCTTGGATGAAACTGTCTTCAAA
GAAGTCTTAGATCGTGTATGGAAAATGGATATACAGGACAATCCTTTATTCTGTGACTGTGATCTGCTATGGCTTGTT
TTCAAAGCAAACAGCCAACCAAAGAAGGTTGTAGGGTGGAACACAAGCTCCTTCAAATGTGCTGCACCCCCTAAGGAC
CAAGGGAAGTCTTTACAAATCCTTCCTTCCCAGTGTGAATACGTCTTTATGCCAAACTTATGGCTGGCCAGCTTGCTT
TCTTCTTTAGGGATCTTCCTCTTTGTGATGACCACGTTCTGTGTCAACTACTACACCTGGAAGTTACGTGACCTGTGG
TTTAGGATAAGGCACAGGGACAGGGTGGTGCAAGTCTGTGACAACGATCATAGGTTCGTCTTCGACGCCTTCATCGCT
CATCACAACGAAGACAGGCGATGGGTTGAGCGGGACCTTTGCCGGAATCTAGAATGTTCCGAGAACTGCCCAAACTAC
CGACTGTGTCTTCACCAGCGAGACTTCCAAGCTGGCGTTCCAATCATCACGAACATCCGTACAGCCGTGGACAGCAGC
AGGAAGATTGTCTGTGTCATCACCAGGAGCTTCCTGCGCAGCCGTTGGTGTCAGTTTGAGTTCCAGCTGGCCCAACAC
ACCATGGTGGAGGAGGGAGGGGGGATACGTCTCATCCTGGTGTTCCTGGAGGACATCCCGCGCCACCTGGTACGACAG
TACCGCCACCTGCAGGCCGTTGTGGACAGGGACACGTACCTGGAGTGGCCGGGGGACCCGAGGGAACGCCCCCTGTTC
TGGAGGAGGCTGAGAGCTGCGTTAGGACAGCCATTAGATCAGCAGCCTGACGATAAGGACTCTGAGCCTGACCAGCAT
GGCTTCATGGCATTGGTGGAGGTGTGA
>Bl10262
ATGCCCACCCCACCACCAATGAAAGAGCTGCGCTCACTGAAGACAATAAGCATGAAAAACAATGCCATACAGACACTG
AATCTTACCAGCGTCAAAGATTTGGCTGAAGTGACAAGCATCAAGTTTACCGGAAATGCATTGGAAAGGATTCCTTTC
CTTGCCTTTGCAAAGAGCACTCTTGCGAAGCTAGAAGATCTAATGTTAGACTCCAACAAGATCTCAGTAATACATGAA
GATGCATTTGGTGGTCTGCCATCTTTAAAAGTCCTAGATCTCAATGGCAACATGATCCAAGTATTGAACAAGGCAATC
CTACACCAGCTTGAAAAAGTTGAAAAACTTAACTTGTCGAACAATGCTATCCATACACTGGAGGCAGAAACGTTCGCA
GAGATGAAGGATTTGCACGTGCTTGACTTGACCCAGAACAATATGACCTTTACATCTGAAACATCCGCATCATTATTT
ACTGGTCCAGAAAATCTGACGAGTTTGTACCTGGCTCAAAACTTGATCAAAAGTTGCCCAGAGGACCTAGGAAAAGCG
CTACCTTACTTACGACATCTTGGTCTACAGAAAAACAACATTTCTGTCTTTTCAGAAACATTCTTGAAGGGATTCGGT

TCCTTGAAAGAGATTGACTTGTCTGGGAACCCTTTCTCGTGCACATGCGACGTGGAGTGGTTATTTGATCGGTTGAAA
GAAAACGGCACTGTAGCATTCATTAAATGGGAAGAATACGAGTGTTACTACCCACGACATTCACGTGGTGTCAACTTC
GAACATTTCAACCCGGAGGTTCTCAAGTGCGACCACACGGACCACACCCGTCTGGGACTGTCCCTGGGGATTTCTCTC
ACCCTGGCTGTACTGGTAATCGTGCTTGCAGCTGTGCTGTACTACCACTGCCGATGGCGCGTCAAGTACGGTTGGTTC
GTGCTATGCGGTAAAAAGGGCGAGCAAGTTGAACAGAAAGTTGAGGACGAGTACTTCAAGTACGAGGCTTTTCTGTCC
TACTGTAGTAACGATCGCTGGTGGGTCATTGACGAATTACTCCCCAAGGTGGAGAATTGTCCCCCGCCCACGTACAAG
ATGTGCCTGGACATTCGTGACTTTGAAGACGGACCGAAAGATCAGAACAACATCATCGCCGCAATGGATGAGAGCCGA
AAGACGATGTTTGTCATCTCCAGTAGTTTTCTGCGGAGCAAGCGCTGCCTGTGGGAACTGGAGATGTCCAGGAACAAG
CGCTACGGGAAGAGCAGGGATGACCTCATCTT
>Bl09440
ATGGCGGCTGGCAGACTGTGGCTTTTCGTCGCCACAACGTTGGTTTTGTGGTCGGTTAGTCAGCTGCAGCAAACGCCT
CTGGCCGGGCTAGGACACATCGGGGCACGTATCAAGGCTTGTCGGGTCTACAACCGAACCGTCGCCGACTGCAGCAGC
CGTGGCCTGCGGTCCGTTCCCGATGCCAGAGAGCTGCCCGGGACGATAAAAACTCTGTACCTTCTGAGCAACAATTTC
CGCGCCCTGCCACACTGTGCGTTCTGCGACCTCCCTCTCTTGGAGTTTGTAGACTTGAGTTTTTGCAACATCAGCGAC
ATTAACAGGACTGCTTTCGCCAAACTCGCACGTCTGAAGACCATTCTGCTGATAGGAAACAACCTCACCAGCCTGGAA
AGTGGAACATTCGACGAACAACACAGTGTTACCGAAGTCAACTTGGCCGACAACAAACTGACGGAGGTACCCTATGGA
GCGTTTCCTCTCAACAACTCACTGAAAACGTTGGACCTATCGGGCAACAACATTAACATACAGGAGGAGGATACCGAC
TGGGACCGTTTCAAATCTCTTCAGCACTTTTCCTTCCGACTGAACAATGTCAGCTCTTCCGCTGGTTGGATCTTTTCC
CGCGAAGATCTGAACGTCTCGCTCCAGACGTTAGACCTCTCGTACAACAGAATCTCCCAACTGGATTCCACGGCGTTC
AACGGCATTCGGAGGCTGGACCGTCTTGTGCTGTCGAGTAACGACATTCTGACCGTAGACACGGACTCTTTCAGCAGC
CTTTCACAAACGGGTCTAGAAACCTTAGTTCTCTTCAGAAATAACCTGACAACGCTAACAAACAATACGTTCTCGTCT
GTACCTTTGTTGAAGCGTTTGGATCTCGCTTACAACAACATTTCGAGTATTGAAGACAACGCTTTTGCAGGATTGAGA
TATTTGGAGGTTTTGACTTTGTACATGAACCCATTATGGACGAACGTCCCCATGACGGCATTGGAAAAACTAAGCCCA
AGTCTTCTCGAGCTTGATTTAAGATTCTGCTCCATACTGAACATCTATCCCGAGCAGTTTCGGTCCTTCGGCAATCTC
CGGAGTCTGAACATTTCCGACAATTACATCGGTGCCCTGGGCAGCGGGCATAGACTGACAGGGAAGGAGTTCGATGGA
CTGGGCAACCTTAGAGTGTTGGACATTGGGGGAGGACCGGGCTACAACAGGATGAAAATCACAAACGAATCCTTCAGC
CACCTCCCGAAGCTCCAGAAAGTGGTCATGACAGGACTTCTCGGTTTTAACGAGCGTTTCGACGGTTCTTTCCGAAAG
AACCCCGATCTGAGGCTGATTAACATGGGCAACATGAACATACGTTTTCTTGGCGTCGACCTCTTTCAAGGTCTAACG
AAGTTGACGGAGCTAGTACTGTCCAACAACAACTTCTACTTGATGCTGAACTCTGACCAAGGTAGTGCAAACTTCGCA
CGATTTTTTGGTGACCTCACGAACTTAGAGATCCTCCGCTTGCAAGGCAATCGCCTGGAGTCTCTCCAAAGTGACATA
TTCCAGAATTTGACACATTTGCGGTATCTGAACCTCGGACCAGGTCGATACGGCGACCACAACAATCAGCTATCTAAC
TTGCAGCCCGAACTTTTCCAATCACTGACTAGCCTGGAAACGTTACGAATCGATGGAAACCGCTTATCCTCCGTGGAA
CAGTCAGTGTTTGAGCCCATGTTTAAGTCTCTGAAAGAACTCTTCATCTACGGAAACCCGTTTGACTGCACGTGTGAG
AACCTGCGCTGGTTTCGGGACTGGGTAAACAACACCAGGGCCGACGTCATCGGGTTGGGAGAAGGCAGGCTGGTTTGC
TCAACGCCACGGAGATATGCAAATGAGAGCATCTTGTCGTTCCGTCCTGAGGTCGACTGCCAGTCGCAGGTGGGGGTG
ATCGTGTCCCTGACGGTGTGCTCGGTCCTGCTCGCGGCGGCGCTGGGGACGGTGGTGTGCCGGCGGTTTGGCTTGCAC
AAGTACGTGCAGTACGTCTGGGTCCTGGTCACCAGGCGGAAGGAAGGGTACGAGAAGCTACAGGGGGAAGACTTGGAG
GTCGAGTACGACGCCTACGTCTCACATACAGAGGAGGACTTGAGGTGGATCCAGAGAGTGCTCATCCCGAACCTTGAA
GAGAAGTCTCCGCAGCTGAAACTGTGCATCCCGGACAGGGACACCCCCCCGGGCGAGGCGATAATAGACAACGTCCAG
GACTACATCCGTCGCAGCAGGAAAACGCTGTGCCTCGTCACTCAACGGTACCTTGCCCAGGAGGCGAGTTGGCTGGAG
ATGCAGGTGGCGTCCTACCGGCTGTTCGACCAGGAGGACCGGCGGGACGTGGTCGTCATGGTGTTCCTGGAACCCATC
CCCAAAAACAAATTAACACATTTCCAGAATCTCCGGAAGCTGATGCGGCCGGGCATGTTCCTCCACTGGCCGGGGGAG
GAGGACACGGCGGCCCACCCGCTCTTCTGGCTCCTCCTGCGGGACGCGCTGGGCACCAGCAACAACCCCGTGCCGAGG
AGTAGGCCACAGGTTATATGA
>Bl22164
ATGTTTCTGCCGCTGTTACTTACTTTGAAACATTTGTTCACCGACGCCCCGATAGCCCCCGGCCTGCTGAAGTCACTC
ACACATCTGCAGACGTTAGGCCCCCGCTATGGGTATAGTCCCGACATTCTGGACGTCCTGCCTGAACTTCGACACACG
CAGATTCAGGAGTTGTCATTTAGCATGTTATTTGGTATGCTTTGGAGATCCAGAATTGAGAAAATAACACCGGATACA
CTTAGTCTAGTAATTAAAGGTCTGAAATATTTAAAAACATTGTTTTTAGATATTACAGACATTAAAACCCTAAAAGTT
AACGCCTTTGCAGGTTTGTCACATTTACAAAGGCTGGGTTTGACGGGGGGCTCCTTGAAGACTTTACACGACAAGACA
TTCAGCGGACTGTCCTCACTTACGCATCTAAACTTGACACAGAACAAAATTCCTGGTTTAACAGAGGGCGTGTTTCAA
GCACTGACCTCTCTCACTCATCTTAACTTGAGCTATAACAAAATTTCTACTTTACCAGCGGGCGTGTTTGAAGGGCTG
ACCTCTCTTAGCCATCTTGATTTGAGCCATAACCACCTGGTAGCTTCCCTAGCTCAGCTTCCAGAGACATTGGACTAT
CTTGATCTCAGTTTCAATCAATTTGACAGCAGAGACACGGGTCAGACACCATGTTACTCGACAATCTTTTCAATAATC
TTCAACTTCAATGGTTTAAAACAGATGAATTATCTGAATTTGAGTCATAATGCTATTACTTACGTTGACGGCCTTTGT
CTACCTCGGAATATTGCTGTGCTTGATTTGCAGCACAACCAGATTGCCTATGTACCCCGCATTCCGAGTGCACGGTAT
CTCGACCTCTCTAATAACTATGTCAAGTATTTTGTTGGTTCTGTACTGCCGCCTGGACAATCAGCCGTAGAAACACTG
CGGCTGGATAACAATGCTATAGTATTAAATGATATGGGAGGTCCAGATTTAGAAGATTGGCTTTCACAGAAATTGCTG
GTAAAACTTAAAACATTGATAGTTTCTCACAACCGCATCCAACGTATGGCAACAGACCAATTCCCAGGGTTGGGCCAG
CTAAAACATCTGGATCTCAGTCATAACGAGATCAACACTATCATGCCATCCGCCTTCCGTGGACTATCGCGGCTAGCA
ATCCTGGATATAAGTGACAACGAGATACAGTTTATAACAGAGATGACATGTGAAGGTTTAAGCAACCTTACAGATCTA
AACCTGGCAGCTAACAGGATAGCAGTTATTGGAAACGCCTTTCGTCGTCTGTACGGGTTGAGGAACCTGAATCTGAGG
AGCAACAGGTTGGCTGTGTTAAATCAAACAACATTCGGTCCTGTTGTCAGTTGGTTAGAAACTATCGACATTGGAGAC
AACCCGTTTTTGTGTGATTGCAAGTTAATGTGGTTTGTTGAGTGGGCCAATGACAAATACGACAGGGTGTCGGACTGG
CGAAACCCCTACCCTAACTTTGGTCGAGGCTACACATGTTCTCGACCAGCAGAACTGCACGGACGACATTTGATAGAC
GGGTTGACGCAGAAACAACAATTCAATGGAAGAAGGAATCCAGCAGAACGAAAGTTCTTTGACGCAGTGTGTAGTCAT
GGATTCCGGCCCAACCGCCTCCTGGCTTGTGTGCTCGCTTCTTCGGGCATCTTCGTCGCCATGATGACCATTTTCCTG
GTCGACTACCACATCGCCCGTGTTCAGTTCTACCTGTGGCAGTTGGCCAAGTGGAGGAGACCGAAGGTTGGAGAAGTA

GAGAATCAAGAGCCGCACAGATACACACACGATGCTTTCCTTGCCTACAACAACCGGGACGTCATGTGGGTTGTACAT
GAAGCAATAGAGAACCTGGAACCTGACTATAGTCTGGTCATACACGAAAGGGACTTTGCAGTCGGCGCTCCCATTGTG
GAAAACATCGCGGATGCCGTGGAAAACAGCCGGAGAACCGTCTGCCTCATCACCAGGAACTTCCTGAAGAGTCAGTGG
TGCGAGTACGAGTTCCAGATGGCCCAGTACCACATGTTTGAGAAGGGGGGAGGGAGGCGTCTCATACTGGTGTTTCTG
GAGAGGATTCCTATGGCATCATTTCCGTGTCAAAATAACGATGTCGAAAAAGCGAGATATACAGCATCCATACAGAGA
TTCATCGTGTGCCCCACGCTAGACGTGCAAGCCAAGCGTCCTTGCCACACGTGTCATACGTACGTTGTGTAG
>Bl05337
ATGATTAACTTGATTGGCTGTCCTTTCCCCACTATCAAATCAGGAACTTTCCGTCATCTGAAGAATCTCACATCATTG
GAAATACCACTACCGCGAACCATCGAAAAAGGAGCATTTGACTTCAAGAATCAAAGAAGCGTTCACATCTATTTGGTG
CCGGTTGACTTATCTTTTTATACCTCGTCATTTCCCAATGACTTCTCCCAACTCTTTAGCAATCAAACTTTGATGAAA
AAGTCTTCTATCCAGATAGACTTTGTCGGTGAATATATCTGTGATTGTAAGATGGCCACATTATCTGTATCCTATCGG
AGGAATCAAACCTTAACCAGACGGATAATGCCACGGGTTGACGGATTAAGCTGCATCTGGAAAAATGACGGGGCATCT
GTTGATTACAATCCCTCTAACAATATTGCCCTACGGTGCCCTGTGTCCGGGCCGTCGTGTCCAGAGCGATGTTACTGT
AGAACAAGCACGATTGTCGCGGAGGCGTTCTGTTCGGACCGCGGGCTTACAGAAGTGCCTGACCGCTTTCCTGTCGAA
ACATCCTTGGTGAGGCTTGACTTGAATTCTATACAGTATGTACAGCCACTAGCATTTGAAAGTGCACCAAATCTTCTG
ATTCTAAACCTGAGCACTAATGCTATTCGGGAGCTCAATGGTTCTGCATTTCTGGGATTGCGCCGTCTCGAAGTATTG
TACCTCGATGGAAACGAACTGTCTCACGTACCAATGGATCTTCTACAATCCCTTGTGAATCTCAGAGCCCTGTGGTTA
AACAACAACAACTTGTTGGATCTACCTCGTACTTTGTTTCATGAGCAAACGATATTTCTTCGTGTCTTGAGGCTTGAT
CACAACAATTTGACGACGTTGTCAACGGGGATTTTTTCAAACCGAACCTCGTTGACGAAGTTGTCCCTGGTCAATAAC
AAATTCGACTGTGACTGTCATCTGCTCTGGTTAAAGACGTGGATGCTTCAACACAGAAGGGTTATAGATGAGATCAAA
TCGGTGACGTGCCGATCGCGTGGAAACGCAAAGGACAAATACATCCTCAAACCAATCATAGAACTTCCAGATGACGCG
TTTATCTGCGGTGATGAGAGCACGTACAACCCTCTGGTCTGGGTGGCAGCGCCTGGAGGGCTTACAATTTTCCTACTC
CTGATTCTGGGAGTCTTTAAGTGCAGGAAGAACGTTCGCGTCTGGGTTTATGCCCGATATCGTAGAGGACTGAGGCAT
CTCGAACAGGAACCAGAGAAAACGTACGACATCTACGTCTCCTACTGCGTGGAGGACGAGGAGTTCGTAGACAGGGAG
GTGGTGAGAGTTCTGGAGGACATGGACCCACCATATAAGGTTTGCCTGAGGAATCGAGACTTCATCCCCGGCCACAAC
AACATCCAGCAGGCCGCGGACTCCATCACCAGCAGCAGGAGAACACTTCTGGTCCTGACGGAGAGGTTCCTGCAGGAC
AGGTGGTGCCTCTGGGAGTTCCAGGTCGCTCATCAACAAGCTGTCACCGACCAGGCGTACCGCCTCATCATCGTCATC
ATGGACGACCTTCCGCTGGAGGCCTGTGATGACGTCATGGACCTGAAGCAGTATCTAACGGCGAACAAGTACCTGTTG
TGGGGAGAGCTGCTATTCTGGGACAAGTTACGGCAGGCTGTACCACCTCCTGGTGTCCGGGGGGAGGTCGATGGCGTG
GAAGAAGAAGGAGAGGAAAATGATGATAGTGACGAATCAGACTATGAAGCTCATGTCAATATTTTTGTTCCGCGCTAA
>Bl07821
ATGTCCGATTTAGTCGTGCTTGATCTAAAAGCTACAGATTGGCCATTAACATTGGAGTCGCACGCTTTTAGGGGACTG
TCAAGAGTACAAAACGTAACAATAACGTCGTGGGAGGACATATTCGTTAAGCCATGCGCGTTTGATGGTATGGTGTCG
CTGCAAAGTCTTACAATGATAAGTGAATACCAGATTTTCCATTTGCCGGAGCAACCGTTTGCAGGTCTTCTCTCTTTA
AAAACTTTAACATTGCAAGCGGGTGACTGTAGTCAGCAGCCCTCTAATGTTTCACAGGTGACATTTCATGACATCGCT
CACACCATTCAAAACATCAGTCTGACCGGCATTTCTGTAACAGGCCCGGCATTTGCTGGTTGTACAAATCTCAGTTCT
GTTCAATGGTCTAACGGGTGCAGCTCTAGCTATAGCTCGCAGCCGGGTCCTAATATTTCTCCTCAAGCGTTTCAAGGA
GCACCGTCACTGGAGTCCATTTCACTTCACTCTATAAGTCTACAAAACATCGAAGGAAGGATGTTCGAAGGTCTTTAT
AATGTTGTTAACCTGTCCATAGAATTTGCTGACTTCTCTGGCATCCAATATCTTCCCAGCGACATGTTCGTCGGGCTT
TCGTCTGTACGATACATTTGGCTCGACGGGAGTCCCGAACTTCCAGGGAACATATTCCAAGGACTCATGTCACTCAGA
GAGATCAGAATTGAGAACATGGTCGCGCGAGCCATGGTGAACGGCTCGGGTAAATTCTGTTATACCGACTTTCAGCGC
TTGTTCTCTGGGCTACAGTCTTTGGAAATCGTCCGACTCATCGACAGCCCAATAGAATGTGAGACACTGCCAACTCTC
ACATTCAGTAACCTCCCGTCTTTAAAGGAAGTACATCTCATTGGCACTCGTCCCTTAAAGTCTTTGCCGATAGGATTT
TTCCACGACGTCCCCGTCCTACAAAAATTCAAATTTACAGAATGGTCACTTAGATCCACAGCACAAGTGACTTTACCA
CCGCTATTTTTTGCAGGGCTTAATATCGAGGTAATAGACCTACGTCGAAAGTCCTTTCTTGATATTTCGAGATATCTG
TTCCAAAACCTAACATCACTCCAGTCATTGTACATAGTTGACTGTGAATTTTCCGAAGAGTCTGCATCCGCAGTTGAG
ACTTTGTTCTCAGGGCTAACGTCGTTAAAACATTTAGATCTCAGTGATACAAAAATGCCATTGCTGAACAATATGTTT
AAAGACCTTGTAGCTCTAAAAACACTAAGGATGACTGACTGGACTTCATCATTACAAAGACGCCCAGTGCCTTCCATG
ATATTCAAAGGTTTACGCTCCTTAAAGGTGTTGTATCTTGAAGATTCCAGATCGTTCCATACCAGTGAAAGCGTGCAA
TTACCGCAAGATTTATTTGTCGATTTAGTCAATTTGGATTATTTGAATCTCGAAAATGTCCCAGTGCAGAGTTTGGAT
AAAGACATCTTCAAAAAGCTGACAAACCTTAGACAGTTGTATTTAAATAACACATTGCTGACACAGGAGGGACTGTCA
ACTTTACCCTTCTCTCATCTCAAAAGTTTAGAGGTACTATCTTTAAGAAGCGTGCCCTTGGACGAATTAAGTCCGGAT
CTGTTCATAGGTGCAAACAACCTCTCCATAATTGACTTGATTCGATGTCCTTTCCCGACTATTAAATCAGGAACTTTT
CGTCACTTGACAAATCTCACATCATTGGAAATACCACGACCGCAAACCATCGAAAAAGGAGCATTCGACTTTGAGAAT
CAAAGAGGCGTTCACATTGATTTGTTTCGTCCGGTTGACCCTTTATTTTCTCTCTCGTCCTTACCCGAAGACTTCGTC
GAACTCTTTAGCAATCAAAATTTGATGGAAAAGTCTTCTATCCAAATAGTAGACTTTAACGACGACTATAACTGTGAC
TGTAAGATGGCCACATTATCTGTCTCCTATCGGAGGAATCAAACCTTAACCAGACGGATAATGCCACGGGTTGACGGA
TTAAGCTGTATCTTGGAAAACGACGGGGCATCTATTGTGAACAATCCTTCTAACAACGCCCTACGGTGCCCTATCTCC
GGACCGTCGTGTCCAGAGCGATGTTACTGTAGAACAAGTACGATTGTCGCGGAGGTGTTCTGTTCGGACCGCGGGTTT
ACAGAAGTGCCTGACCGCTTTCCTGTCGATACCTCCTTGGTGAGGCTTGACTTGAATTCTATACAGTATGTACAGCCA
CTAGCGTTTGAAAGTGCACCAAATCTTCTGATTCTAAACCTGAGCACTAATGCTATTCGGGAGATCAATGGTTCTGCA
TTTCTGGGATTGCGCCGTCTCGCAGAATTGTACCTCGATGGAAACGAACTGTCTCACGTACCAATGGATCTTCTACAA
TCCCTTGTGAATCTCCGTGTCCTGTGGTTAAACAACAACAACTTGTTGGATCTACCAAGTACTTTGTTTCATGAACAA
ACGATCTTCCTTCGTGTCTTGAGGCTTGACCACAACAATTTGACGACGTTGTCAACGGGGATTTTTTCAAACCAAACC
TCGTTGACGAAGTTGTCCCTGGTCAATAACAAATTCGACTGTGACTGTCATCTGCTCTGGTTAAAATCCTGGATGCTT
CAACGCAGAAGAGTTATAGATCAGATCAAATCGGTGACGTGCCGATCGCGTGGAAACGCAAAGGACAAGTACATCCTC
AAACCAATAATAGATCTTCCAGATGACGCGTTTATCTGCGGTGACGAGAGCACGTACAACCCTCTGGTCTGGGTGGCA
GCGCCTGGAGGGCTGGCGATCTTACTACTCCTGATTCTGGGAGTCTTCAAGTGCAGGAAGAACGTCCGCGTGTGGGTT

TATGCCCGATATCGTAGAGGACTGAGGCATCTCGATCAGGAACCCGAGAAAACGTACGACATCTACGTCTCCTACTGC
GTGGAGGACGAGGAGTTCGTAGACAGGGAGGTGGTGAGAGTGCTGGAGGACATGGACCCACCATATAAGGTTTGCCTG
AGGAATCGAGACTTCATCCCCGGCCACAACAACATCCAGCAGGCCGCGGACTCCATCACCAGCAGCAGGAGAACACTT
CTCGTCCTGACGGAGAGGTTCCTGCAGGACAGGTGGTGCCTCTGGGAGTTCCAGGTCGCTCATCAACAAGCTGTCACC
GACCAGGCGAACCGCCTCATCATCGTCATCATGGACGACCTTCCGCTGGAGGCCTGTGATGACGTCATGGACCTGAAA
CAATATCTAACGGCGAACAAATACCTGCTGTGGGGAGAGCTGCTATTTTGGGACAAGTTACGGCAGGCGGTACCACCT
CCTGGTGTCCGGGGGGAGGTCGATGGAGAGGAAGAAGAAGAAGGAGGGGGAAATAATGATAGTGACGAATCAGACTGT
GAAGATCATGTTGATATTTTTCTACCTCGCTAA
>Bl52875d
ATGTTGTTCCTACAGAAGAATCGTTTGTCCTGTCTGCCACATGACCTTTTTGCCCAGCTTTCCACTCTGGAAAATCTT
GACCTCTCCTACAACAGTCTAAAGAACATACTTCAGGCTGACATTTGGAAAGGTTTAAATCTCCAGAAGCTAGACATT
TCCTACAACCAACTCATAAGCAGCAAATTTTCAAGTGACTTTGCTGCAATGTCTTCACTGCGTGAACTAATTCTGTCG
GGAAACAACATATCCTCTTTGAACTATTCTGACTTCCAACCTTTCCTTTTGCATGGGTTTGACTTACTTGACTTTTCT
GACAATCCGATAACTCACATAGACAAATCTTTCTTCACTCTGTTCAGTTTTTTACACATTTTGGACATATCTGGCCTT
CCTATTTTGTTTTCCAACTTGCAAGAAGCACTTGAAGGCTTGACTAGCTGTAACATAACAGCAATTTGGCTGGATAAT
TATAATGGTCTTCCTATCATTCAAGCTGGTTCCTTTGCTTGTCTACAGAACACAAGCTTGGAGATATTGACACTGGAG
AATTCAAATCTAAAGGAAATACAAGACAATGGTTTTTGTGGGTTGAACCATCTTAAGGATCTTGTCCTTGCAGGAACC
AAGCTTACAGAACTGCCAAGGCTAGCTTTTCATGGATTATCTAATTTACAGTTTTTGGATCTGTCAGTGCTTCCCCTG
ACACACATTCCAAAAGCTGCACTGTCAGAGGTAGCACAAACTCTGAGGGAACTACAAATGTCTACAACTGGTGTGGAA
ATTATCCAAACAGATGAGTTTAATGACCTATCAAACTTGAAGATCATAAATCTTGGTTATGGCATTCTTTCAGATATT
CAACCTTTTGCCTTTAGAGGACTTGGAAACTTAGAGAATCTACAGCTTCATGTAAATATTCTGAAAAATTTGTTGGAT
AATACTTTTGCTGGACTTGACAAATTAAAGATGTTGAATCTAAGTGGATGCAGTATAAGAAGTTTTTCAAATAAAATC
TTTGCTAATTTAACATCACTAGTAAAGCTGGATTTGCAACAAAATGATCTAGTACATGTACCAACTTTAGTGTTCTCT
GATTTAAGATCTCTTGAAGAGCTCAGTGTTGCTGGAAACCACATATCTCCATATGACTCAATAGACTTGACTAGTCTT
GCCAGCATTAAGCACATTGATCTGAGTTACAATGATCTCTTACTTCGAGAGACCACTTTTGACTTCCCTGCAAACACT
AGGTTAAAATCCCTTGACTTGTCCTATAATAAACTCTTTTCTTATTCATCCACATTACCATGTTTTGGGCCATGTACA
TTCTTTAAAAAATTGCAAAACTTAACAGAGCTAGATCTCCAAGGGAACAAGGTTACCCCACAGCGAGGTCGTGGGGTC
ATCTTTCATGGTCTTGTATCTTTGACCACCCTGATTATGAGAGAGACAGGAGAAATTTGGTCTAGTTTTTCAAATGAA
ACCATCTTATATGGCATGCCCAATCTGGAGGTCATTGACCTATCTGCATCTTCAATATCATACATCCCTGCTGAGTTG
TTCAAAGTTCATGATAACCTACGTTCTGTTGACTTGTCATCAAACAAAATAACGTATCTTCCACGGACTCTGTTCTCA
GCTAACTCCAAATTACAAACCCTATTCCTATCTCGGAACCTCATAACCTTGTTAAATGAGTCCAGTTTTGAGACTACT
TTACCAGGGCTTAAGAACTTGGCCATCAATGATAACCCCTTCTTTTGTGACTGTGAGATTGAGTGGTTTATATCCTGG
GCTGAGAATCACTCTTCTTTAGTGCAGGGTTGGTCTGATGGAAGGTACCAATGTAACACCCCTCCCGACCTTCACAGC
ACAGATCTGCGTAACTTTCATCCTGATTGTGCCTCTCACCGTGACCTCTACGCCTGCGCCATAACGACCTCTTTCCTT
CTCCTCTACATGCTCTTTGCTGTCCTTGTCAATTTCTGCAGCGGCTATTTTGCTTACTTGTGGTTTAGAGTTAGGCTC
CGTCTCCGTGGATATGAAGAGATCCCTGAACAACCTCAGCAGTTTAGATACGACGCCTTTGTGGCATATAGCAGCAAT
GATGAAGCCTGGGTTTCCCGCGTGTTGTCCCCCATGCTGGAACGGCGCCCTCCTCGCTACCGTCTGTGTATCGGAGAA
CGTGACTTTGTAGGTGGTGTGCCGATCTTACACAACATCAGCAATGCCGTGGAAACGAGCAGGAAAACGGTCTGCATC
ATAACGAGGAGCTTCCTACGCAGCAACTGGTGCAACTACGAGCTACAGATGTCCCAGGGCAGACATCACCTGTTCGAC
CCGCGTAGAGTGAGCCTGGTTCTCGTGTTTCTGGAGAACATCCCGGATCGTGTCTTAGAGCGCTACCCGCTGCTGAAT
AACATCGTTAACAGGGACACCTACCTGCGCTGGCCCAACAACCAGCAGCACCTACCTCTCTTCTGGGCCAGACTACTA
CAAGCACTGGGTCCACCTCTCGGGGATGACTTACAGGAGGATGATGGTATTGAGGAAGATGTAGTTTAA
>Bl52875c
ATGACTTCACTGCGTGAGCTACTTCTGTCTGGAAACGACATATCCTCTTTGAATTATTCTGATTTCCAACCTTTCCTC
TTAAATGAATTTGATTTGCTAGACTTTTCATACAACCCAATAACTCATATTGACAAAGCTTTCTTCACCCTGTTTAAA
GCTATAGAAACTTTGGACTTATCGAACATTCTAATTTCATTTTCCAACTTGCAAGCAGCACTTGAAGGCTTGAATGAC
TGTAACAAAATCAGCCTAGATTCTTATACAGGTCTTCCAGTAATTTCATATGGGTCTTTTGCCTGTCTGCAAAACACA
AGCTTGAAGTCATTGAAACTTTCACGTGCAGAAATCCAGGAAATACAAGACAATAGCTTTTATGGGTTGACCCATCTT
GAAAATCTTGGTCTACAATGGAACTTGCTAACTGAACTATCTGGGTTAGTTTTTCATGGATTATCAAGTTTACAGGTC
TTAGATCTGTCATTTGTCTCACTGACACATATTCCAACAGCTGCATTATCAGAAGTATCACATACTCTGAGGGAACTA
ACCATATTTTCAACTGAGATGAAAACAATCCATAAAGATGATTTCAATAACCTTCCAAACTTGAAAGTCTTATATCTG
AATGCAGACTTTAGTGGGGGACTTGAAGAGATTGAACCATTTGGTTTCAGAGGACTTCAAAACCTAGAGGTTCTTCAG
CTTGATGATAATGACCTGACACATTTAGTTGAGAACACTTTTGCTGGACTGAACAACTTGACAAAGTTGTCTCTAAAA
CGCTGTAATATAAAAAGACTTTCTGGTAGAATATTTGCTAATCTAACTTCCCTGGTAGAGCTGGATTTGAGTGTAAAT
AACCTAGTTCATTTACCAAAGATGCTTTTTTCAGACTTGATATCTCTTGAAGTTCTGATTATCAATAATAATTACAAT
CTATTTCCATATTTTGAATCAATAGACTTCACCGGTCTAGCCAGCATAAAGCACATTGACCTCAGTCACGATGGACTC
TTTCTGGATAACCAAACTTTAAACTTTCCTACAAACACAAGACTAGAAACCCTGGACTTGTCCTACAACCAACTTTTT
GTTGCATCACTACCTAATGAGCGTTCAGTCTTTGAAAAACTCAGATATCTAACTGAGTTGAATCTCAGTGGAAATAAG
CTGGCCAAAATACCTGCCTATGGGGCTATATTTCACGGTCTTTCATCTTTAATCAAACTGATCATGAAAGAAACAGGA
GAAATTTGGTCTAGTTTTTCAAATGACACCATCTTATATGGCATGCCTAATCTAGAGGTCATTGACCTATCCGCATCT
TCCATATCATACATCCCTGCTGAGTTGTTCAAAGTTCATGATAACCTACGTTCTGTTGACTTGTCATCAAACAAAATA
ACGTATCTTCCACAGACTCTGTTCTCAGCGAACTCCAAATTACAGAGCCTATTCCTATTTTCGAACCTCATAACCTTG
TTAAACGAGTCTACTTTTGAGACCATTTTACCAGGGCTGGAGAAATTAGCCATCCATGATAACCCCTTCTTCTGTGAC
TGTGAGATCAAGTGGTTCATGTCCTGGGCTGATGATCACCCTTCTGTAGTGCAGGGTTGGTTGGATGGAAGTTACCAA
TGTAACACCCCTCCCGACCTCCACGGCACAGACCTGCAGAACTTTCATCCCGATTGTGCCTCTCACCGTGACCTCTAC
GCCTGCGCCATAACGACCTCTTTCCTTCTCCTCTACATGCTCTTTGCTGTCCTTGTCAATTTCTGCAGCGGCTATTTT
GCTTACATGTGTTTTAGAGTTAGGCTCCGTCTCCGTGGATATGAAGAGATCCCTGAACAACCCCAGCAGTTTAGATAC
GACGCCTTTGTGGCGTACAGCAACAATGATGAAGCCTGGGTGGCCCGTGTGTTGTCCCCCATGCTGGAAGACCGCCCT

CCTCGCTACCGCCTGTGTATCGGAGAGCGTGACTTCATAGGTGGTGTGTTGATCTTACACAACATCAGTAACGCCGTG
GAAACGAGCAGGAAAACGGTCTGCATCATCACGAGGAGCTTCCTACGCAGTAACTGGTGCAACTACGAGCTACAGATG
TCCCAGGGCAGACATCACCTGTTCGACCCGCGTAGGGTGAGCCTGGTTCTCGTGTTTCTGGAGAACATCCCGGACCGT
GTCTTAGAGCGCTACCCACTGCTGAATAATATCGTTAACAGGGACACCTACCTACGCTGGCCCAACAACCAGCAGCAC
CTACCTCTCTTCTGGGCCAGACTACAACAAGCACTGGGTCCACCTCTCGGGGATGACTTACAGGAGGATGATGGCATT
GAGGAACATGTAGTTTGA
>Bl52875b
ATGTTTGAAGACCTTGCTGTGTTTTCAATATCAAAGTCAGTTGAAGTTCCCACACCTGCGATGGCCACTGGTGAGAAA
ATCAACAGCAACAGCTTGCGACTGTTTGGACTGAGGATGTTTTTAACACTAAGCACGGTTTTAGCGCTATGCACAAGA
ACACAAGGAACTCCATGCACCATTGTTAACAAGACAGCCACCTGTTCCAATGGCGGACTGACTCAGATTCCAAGCAAC
CTTCCTCACAATCTCACCAACCTCGACCTAAGCAACAACGACATCCAGGCAATACGCAACAACTCCTTTTCTTCCTTA
CACTTTCTGGAGGTTCTCAACTTTGGAAGAAACAATCTGTCAGTCATTGAACCAGCTGCCTTCTATAACCTCTCCAGT
CTGAAGACATTGTTGCTAAAGGAGAATCGTTTGTCCTATCTGCCACCTGGCCTTTTTGCCCCTCTTTCTACTCTGCAA
AATCTTTACCTTTCTGACAACAGTCTACAGAACATCCTTCAAGCTGATGTTTGGAAGGGACTGAATCTCAAAAAGTTA
GACGTTTCCTACAATCAAATCATTAGTGGCACATTTAGAAGTGATTTTGCTGCAATGACTTCACTACGTGAACTAATT
CTGTCGGGAAACAACATATCCTCTTTGAACTATTCTGACTTCCAACCTTTCCTTTTGCATGGGTTTGACTTACTGGAC
TTTTCTGACAATCCGATAACTCACATAGACAAATCTTTCTTCACCCTGTTCAGTTTTTTACACATTTTGGACATATCT
GGCCTTCCTATTTTGTTTTACAACTTGCAAGAAGCACTTGAAGGCTTGACTAGCTGTAACATAACAGCAATTTGGCTA
GATAATTATAATGGTCTTCCTATCATTCAAGCTGGTTCCTTTGCTTGTCTACAGAACACAAGCTTGGAGATACTGACA
CTGGAGAATTCAAATCTAAAGGAAATACAAGACAATGGTTTTCGTGGGTTGAACCATCTTAAGGATCTTGTCCTTGCA
GGAACCAAGCTACAAGAACTGCCAAGGCTAGCTTTTCATGGATTATCTAATTTACAGTTTTTGGATCTGTCAGTGCTT
CCCCTGACACACATTCCAAAAGCTGCACTGTCAGAGGTAGCACAAACTCTGAGGGAACTACAAATGTCTACAACTGGT
GTGGAAATTATCCAAACAGATGAGTTTAATGACCTATCAAACTTGAAGATCATAAATCTTGGTTATGGCATTCTTTCA
GATATTGAACCTTTTGCCTTTAGAGGACTTGGAAACTTAGAGAATCTACAGCTTCATGTAAATATTCTGACAAATTTG
TTGGATAATACTTTTGCTGGACTTGACAAATTAAAGATGTTGAATCTAAGTGGGTGCAGTATAAGAAGTTTTTCAAAT
AAAATCTTTGCTAATCTAACATCACTAGTAAAGCTGGATTTGCAACAAAACGATCTAGTACATGTACCAACTTTAGTG
TTCTCTGATTTAAGATCTCTTGAAGAGCTCAGTGTTGCTGGAAACCACATATCTCCATATGACTCAATAGACTTGACT
AGTCTTGCCAGCATTAAGCACATTGATCTGAGTTACAATGATCTCTTACTTCGAGAGACCACTTTTGACTTCCCTGCA
AACACTAGGTTAAAATCCCTTGACTTGTCCTNAACCCTGGACTTGTCCTACAATAAACTCTTTTCTTATTCATCCACA
TTACCATGTTTTGGGCCATGTACATTCTTTAAAAAATTGCAAAACTTAACAGAGCTAGATCTCCAAGGGAACAAGGTT
ACCCCACAGCGAGGTCGTGGGGTCATCTTTCATGGTCTTGCATCTTTGACCACCCTGATTATGAGAGAGACAGGAGAA
ATTTGGTCTAGTTTTTCAAATGACACCATCTTATACGGCATGCCCAATCTGGAGGTCATTGATTTATCCTTATCTTCC
ATATCATACATCCCTGCTGAGTTGTTCAAAGTTCATGATAACCTACGTTCTGTTGACTTGTCTTCAAACACAATTACG
TATCTTCCACAGACTCTGTTCTCAGCTAACTCCAAATTACAGAGCTTATTCCTATCTCGGAACCTCATAACCTTGTTA
AATGAGTCCAGTTTTGAGACTACTTTACCAGGGCTTAAGAACTTGGCCATCAATGATAACCCCTTCTTTTGTGACTGT
GAGATCGAGTGGTTCATATCCTGGGCTGCTGATCACTCTTCTTTAGTGCAGGGTTGGTCTGATGGAAGGTACCAATGT
AACACCCCTCCCGACCTTCACGACACAGATCTGCGTAACTTTCATCCTGATTGTGCCTCTCACCGCGACCTCTACGCC
TGCGCCATAACGACCTCTTTCCTTCTCCTCTACATGCTCTTTGCTGTCCTTGTCAATTTCTGCAGCGGCCATTTTGCT
TACATGTGGTTTAGAGTTAGGCTCCGTCTCCGTGGATATGAAGAGATCCCCGAACAACCCCAGCAGTTTAGATACGAC
GCCTTTGTGGCGTACAGCAGCAATGATGAAGCCTGGGTTTCCCGCGTGTTGTCCCCCATGCTGGAACGGCGCCCTCCT
CGCTACCGTCTGTGTATCGGAGAACGTGACTTTGTAGGTGGTGTGCCGATCTTACACAACATCAGCAATGCCGTGGAA
ACAAGCAGGAAAACGGTCTGCATCATAACGAGGAGCTTCCTACGCAGCAACTGGTGCAACTACGAGCTACAGATGTCT
CAGGGCAGACATCACCTGTTCGACCCGCGTAGAGTAAGCCTGGTTCTCGTGTTTCTGGAGAACATCCCGGACCGTGTC
TTAGAGCGCTACCCGCTGCTGAATAACATCGTTAACAGGGACACCTACCTACGCTGGCCCAACAACCAGCAGTATGTA
CCTCTCTTCTGGGCCAGACTACTACAAGCACTGGGTCCACCTCTCGGGGATGACTTACAGGAGGATGATGATATTGAG
GAAGATGAAGCGTAA
>Bl52875a
ATGATATTTCATGGTCTTGTATCTTTAACAACCTTGAAAATGAGAGAGACAGGAGAAATCTGGTCTAGTTTTTCAAAT
GACACCATCTTATATGGCATGCCCAATCTAGAGGTCATTGACCTATCCTTATCTTCCATATCATACATTCCTGCTGAG
TTGTTCAAAGTTCATGATAACCTACGTTCTGTTGACTTGTCTTCAAACACAATAATGTATCTTCCACAGACTCTGTTC
TCAGCTAACTCCAAATTACAAACCCTGTTCCTATCTTGGAACAGCATAACCCTGTTTAATGAGTCCAGTATTGAGACT
ACTTTACCAGGGCTTAAGAACCTGGCCATCCATGATAACCCCTTCTTTTGTGACTGTGAGATCGAGTGGTTCATGTCC
TGGGCTGCTGATCACCCTTCTGTAGTGCAGGAGTGGTCGGAGGGAAGGTACCGATGTAACACCCCTCCCGACCTCCAC
GGCACAGACCTGCGTAACTTTCATCCTGATTGTGTCTCTCACCGTGACCTCTACGCCTGCGCCATAACGACCTCTTTC
CTTCTCCTCTACATGCTCTTTGCTGTCCTTGTCAATTTCTGCAGCGGCCATTTTGCTTACATGTGGTTTAGAGTTAGG
CTCCGTCTTCGTGGATATGAAGAAATCCGCGAACAACCCCAGCAGTTTAGATACGACGCCTTTGTGGCGTACAGCAGC
AATGATGAAGCCTGGGTTTCCCGCGTATTGTCCCCCATGCTGGAACGGCGCCCTCCGCGCTACCGCCTGTGTATCGGA
GAGCGCAACTTTGTGGGTGGTGTGCTGATCTTACGCAACATCAGTAACGCCGTGGAAACGAGCAGGAAAACGGTCTGC
ATCATCACGAGGAGCTTCCTACGCAGCAACTGGTGCAACTACGAGCTACAGATGTCCCAGGGCAGACATCACCTATTC
GACCCGCGTAGGGTGAGCCTGGTTCTCGTGTTTCTGGAGAACATCCCAGATCGTGTCTTAGAGCGCTACCCGCTGCTG
AATAACATTGTGAACAGGGACACCTACCTACGCTGGCCCAACAACCAGCAGCACCTACCTCTCTTCTGGGCCAGACTA
CGACAAGCACTGGGTCCACCTCTTGGGGATGACTTACAGGAGGTTGAAGACATTGAAGAGGATGTTGTCTGA
>Bl72403
ATGCTGGTACTGCTGCAAGTTATATCCTGCCAAGGCTTGGCCATAAAAGTGCCACAGTGCAAAATGACAAACACCACA
GTTGACTGCTCTGACAGAGGATTCATCCAAGTCCCTCCCAACATACCTACCAACACAACAAGTCTGGACCTCAGCTCA
AACGACATACAGCAGTTGGACAACTACTCCTTTTCAAGACTACCTCTCTTGACATTGCTAGACCTAAGTTCCAACGAC
ATGCTCATCATCGAACCTGTTGCTTTTTACAATCTTTCCCAACTGGTGGAATTATCATTATCTATAAACAAACTCTCT
ACGCTTCCATCTACAGTATTTGAACCTCTGAAATCATTGGCGAGTCTTTCTCTCTATGACAATGAATTCTCTGACATA

CTAGGTTTTGATAAGATATGGGAGGGGTTGCCCTTAACAAAACTTGATTTGAGTAGGAACAAAATGATGAAAGCTGAG
TTTTCAAAAGCCTTTTCTGAAATGCACTCACTGAAGTTATTAGACCTGTCCTTAAATAGAATCTCGGTTCTTAAAATT
GCTGATTTTGAGCCCCTTGCTTCGCATAGTTTTCAGACTTTGGAATTATCAGCGAATCCTATAACTTTTGTAGAATCC
GGGTTCTTCGCACAGTTTCATAGAATAGAATATCTTGGTCTTTTGATAACAAGTTCAAACATTACGATTCTACGTGAT
GCATTTTCAGGTGTACACTCTGTAAAAGTGACTCAGTTAGCCCTAGGCATTGCAGACTTCTTCATTGTAAACGAGGAC
GCCTTTCAGTTTCTGAAAAACACTTCGTTACAAAATCTTGTATTAGGATGGGGAGATATCAAAGAACTTAAAGATTAT
GCTTTCCGTGGATTGGACGACCTAGAAACTCTTAATCTCCAAAACAATCAGATTGAAGAAGCATCTCCACAGGCATTT
GGGGGACTACATAACCTAGTTGTTCTGGATTTGAGAGAAAATCATTTTAAACAAGTTCCCTTCAAGGCACCTAGTGTC
GTATTTCCAGCTCTGAGGGAATTACACCTCGAGGACAACAAAATAAAAAACATTGTGCCAGAAAACTTCATAGGCCTT
CCAAACCTGCAGCTACTCAAACTGGACAATAACCTGGTAACAAACATTCCCGCAAATGTTTTCCAATGTCAAAGGAAT
CTAGACACACTGTCGCTACAAAATAACAAGATCAGTCTTCTTTCAAACAGGAGTTTTACGGGCTTGGAAAATGTGACT
TTGATGGACCTGTCTATGAACAAACTCCCTGTTCTTCCTGGCAGTGTCTTCTTTCCGCTGATAAAACTAAAAAAGCTT
GACCTATCATATAATTATCTTTCTCCTGAAATGGAAGATTTTTATGGCCTAGAAAAAATAGAAACACTAAAATTGCGT
CGAAATGACATAAGTCTAAAGTGGAACACATTTAAGCTAGTTTCAACTTTGACATATCTGATATTATCTGAAAACGAT
CTGTTTGGTGGCATTCCCAATCACAAGCCTTTTATAACTATGAGGAATCTTGTGACTCTTGACATAAGCAACAACCAA
ATCAAATTTCAGAGCGACTCCATGAGAGAATTTTTTGAAGGCCTGGTGTCTTTACGGAGTTTACAAATGTATCGCACA
GGAGATATTTGGGCAAGGTTTCAGAACGCGACTATTCTGTATAGCATGCCAAATCTAGAAATGCTTGATATGAGAGAA
TGCAAGATACAGTATATCCCTCCTAATGCATTTAGCATCCATGTAAACCTACAGTATGTAGACTTCAGTGATAATGCT
ATTTCTAGTTTACCTGACAACCTATTCTTGAATGTCCGGTCTCTAAAAGCACTATATCTGGAAACAAATGCCATTTCT
TACTTAAACGAGTCAAGCTTTGCAACTGTCTTGCCGACTCTCGAAAGCTTAGGCCTGGATCTAGGTGAAAATCCCTTC
TACTGTGATTGTAGGGTAACATGGTTTATCAGCTGGGCTGACGCTAACCCCTCAAAAGTTCTTTTCTGGGAGAAAGAT
GGGTACTATCTCTGCAACACCCCTGCATCTCTGCACAACAGAGATTTACGGACTTTCCATCCGGATTGCGATTCTCAC
TTGAACTTGTACATGTGTGTCGTCACGACTTCCCTCCTAGTGCTGTACATGTTTACTGTGTTTGTGGTAACTCAGTAC
AGTTTATATGTCTGTTACATCTGGTATCTGTTACGGGCCTGGTTAAGAGGATATGAAGAAATTCCGGACATGGGGGTA
CAGCGGTTTGAATACGACGCGTTTGTGGCGTACAGCAGCAAGGACAAGAGATGGGTGTCACAAGTGCTACGTCCGCAA
CTTGAGGACCGTCCACCGCACTATCGACTCTGCTTCGGCGAGCGAGACTTTCGTCCGGGTGTGCCGATAACGAAAAAC
ATTGGCCGAGCCGTCCGAGCCAGTCGCAAAACCATCTGTCTGATTACAAGGAGCTTTGTGAGGAGTAACTGGTGTAAC
TACGAAATGCGAGCGTCAGAAGGACGCTACCATCTCTTCGATCCGCGTAGGGTCAACCTGGTTCTCGTGTTCTTAGAG
GAAATTCCAGACCGCGACATGGAACGCCATAAACACTTGAGAGACGTTGTGATGAGAGACACTTACATAAAGTGGCCC
AGAAATGAGAAGGGTAGGCCGCTATTCTGGGCTAGACTGAGAGAAGCTTTAGGACAACCTCTACCTGTCAATCAAGGT
GGAAACAGGGACATTTTTGAGGACAGTGTATAG
>Bl19440
ATGACTCTTAGGCTCGTGGCCCTTGTCTGTCTGTCGACGTGGTTCCTGTCGGGAGCACTATCTGCTACAAATGGAGCT
GATCCACTCGGATGTCAGGTATGGAGTCTTACCGATGTTACATGCGCGAATACGCTCATCCTGAAGGATGTCCCACCA
GACCTGCCTGCGTCCATTCTCCGATTGGATCTACAGGGCAACGACATAACACAGCTCAACAGACAACCACTAAATGCT
CTCAAGAACTTGCAGTACTTGGATTTGAGCTGGAACCGTATCGAGTATGTTGAGAATGGAACATTTGATTCGATGACA
AATTTACGGATTCTGAACTTATCCAGAAATGGTCTAGGAGACTCGCAGTGGTCCAACATAACCTCGTATCTTCCCTCT
CTACAAATTCTCATCCTTTCGAGAAACCGTCTGCAGGACTTGAGTAGAGGGTCTTTCATGGGTTTACACAACCTGACC
TTCTTGGACCTTTGGTCTAATGCCATCATTGTCGGAGAGTGTGCGTTTTGTGGACAGCAAAGACTACAAAGGCTCGAC
ATGACCGGTAACGAGATAAGTACCCTGCCCAACAGGCTTTTTCATCCTCAATCAGAAATTCAGGTATTGGATCTGTCG
AACAATAGACTTCAAGATGATGTGCTAGAAAAGGAGGAACTCTGGTCTGGGCTAGGGAAACTCAGAACACTGGACGTA
AGACGAAACTACTTCTCCATTCCTAAATTTGCACAGTCCATTAGAAACCTGACATCCCTTCACAACATTTACATGTCC
AGCAACAAAATTCAGCAGTTCCTCTTTCAACACGTGGCTCCTCTGTTAAACGCACCGATCCATGAGTTCGTTTTGGGT
TACAATCCAATATCCATATTTGAAGAGGGCATCCTGCTACGGCTACGGCATTTAAAAACATTAGAACTTGGCCAAACG
TCAATTCCGTCTTCAGAATTGCAAAAGATATTACCAGAATTGCAGAATACGAAGATACGAAAGCTAGAATTGGGAACA
GCCCCAGGCAACTTTGCCATAGATAACACAACTTTTGGACAACTGCCAGGTTTAGAGGAACTAAGTATTTACGGCTCA
GACTTGAGTGTGTTAAAAGCAGAAGTCTTACGACATTCAAAAAACCTAACGGATTTGGCCCTGAACAGCAATAACATT
ACTTTCATTGATGAGCGTGCATTTATAGGATTAAAAAGCCTAGAAAAGCTACAGCTGCAACGAAACAATATCCAAACT
TTGCCAGACAAAGTTTTCGACCCGCTGTTTGCCCTTACTTATCTGGATTTGAGCTCAAATAGTTTGAACACCATTTCG
CCTGCACTTTTTAAAGGGCTCCTAAATCTCCAGTGGCTAGATCTCTCTAGTAATGGTATTACCAAAATTGATAACACT
TCTTTCATAGGACTAAGTTCTCTAACCCATCTCGATTTATCCTACAATCGGCTTACCACAGCTGGACTGCCTGTTGTA
GGGCCAAGCGTCAAGTCTATTGATTTGAAGGCAAATCAAATTGAAATTCTAAAAGCAAATGAATTCTTAGGTTTTGAG
AACTTAGGCTCGTTGTGCCTAGAAAGTAACAATATCAGATCAATTGAACCTCATGCATTCCAAGGTCTAGGAAAATTA
GAGAAACTTGATCTAGGTGGCAATAAACTTAGAACGGTGATAGGTAGGATGTTTGAAGGCCTTGAAAATGTGACGGTG
TTGGATTTTGATTCAAACACTCTACTTGAAATTGAAATTGAACCGTATGGTTTCTATGGTTTAAGGCGTTTAAAAAGG
CTTGAAATTGGTTTCACATTGAGAGCAATTCCGTCATACGCTTTTGTGGGCCTAGATAGTCTAACTTTCTTGGACCTC
AGTCATAACCAGATTGCTAAAGTAGGAAGGCATGCATTCGACGGTCTACCTAACATGAAAATATTTCTTTTCGTAAAA
AATAAAATCACCGTCTTCGAAGAGGAGACATTTGGACGCGTCGTTCGGCAAGCTTCTCGTATTCAGTTTGAACAGAAT
CCATTCTATTGTGATTGTAGACTTCTTTGGTTTGTGGAATGGGCGAGAAAGAACCCTGATAAACTCCATTTCTTTACT
TTCGGCTGGTACAAGTGTGCTGGACCTCCAGAGTACGCGGACACGAGATTGGTTAACTTCTACATCAACTGTACGTCG
CCCGAGGACAACGTATTTCAGCCTAACAGGCCATTAGCCTGTGCATTAGTATCTTCAGCAATTCTGTTCTACATGTTT
GCGGTGTTTCTGGTCAGCTATCACCACTGGAAGATTAAGTACTTAATGTTTTTGCTTAGACGCAGAAATGAGGACGAT
GAACCAATGCGTGGCCGTAGGTTTGTGTACGATGCCTTCGTAGCACATAACAGTGAGGACATTAGGTGGGTGGTGCAC
GAACTCTGCCACAACTTAGAAAATGTCCAAGACCAACCCAGGTACAAACTCTGTATCCATCAGAGAAACTTCTTACCC
GGCGCCCCCATTGTAAACAACATCGTCAAAGCCATCGAAACCAGCCGGAAAACCATCTGCGTGTTGACCAGGAGCTTC
CTGTGGAGCGGCTGGTGTGAGTTCGAGCTGCAGCTGGCTCAGACTCCGGACAACTTGTTCGGCAAGGGCGGCAGTTGT
CGACTGATTCTCGTCTTCTTGGAGAAGATTCCTCGGCCGCTGCTGAAGAAGTACAGACACTTGGAGGCCGTGATGGAC
CGGGACACGTACCTGGAGTGGCCGGGGGATGCGCGGGGCAGGCCGCTGTTCTGGAGACGCTTGCGGGCCGCCTTGGGG

AAACCCGTGAACGTTGAACAGGCGGGAGATGGTGGAATGGGAGACGAAGACGACGAACTTGAACCGTTGTTAAGAATG
TAA
>Bl21810
ATGGAGTCCAAACCCGCTCTTCTCGCAGTCTGCTTGGCTGTGGTTTCCGCGTTCATCGCCCAACGTGTTAGAGGAGAA
CTCCTCCCGCCTGTTTGTCAGATCTGGAACTCAACAACTGTGGTGTGTACACGCGGTGTATGGGACGATCCAAACCGC
GCGCCCCTTACCCAAGTACCGCCCGGCATCCCCGAGTCTGTCATCGCCTTAGATCTCAGTCACAACAACATCACTGAA
CTGCACAACGGCTCGTTTAGTGGGTTAAGAAACCTGAAGTCCTTAGACTTGTCTCACAATAGTCTACAAATCATCGAA
GTCGGTACGTTCGCTGGGTTGGAGAATTTAGAACGTCTGGATATAGGTAACGTTACATTGATGCCGCAACAGGAACCC
ATGTATGCTCTTCAAAGTGGACTTTTTCGAGATTTGCGAAATCTGAACTATATTGTCGTGCAGACATGGACCGATACA
GTCAGTGAGGGAGTGTTCACGGGGTTAGTTAAACTAAGACATTTAGATCTGAGTGTAAAAAACATCAGCAATTTACCA
GATCACGTTTTTGATTCTTTGACTTCATTGGAAAGTCTGACCATAGAGGAACTCGCGATCCGCGATGACATAGACGAG
GACCAACATACCGCCCCTACAAGAGTCCACAGTGGAAGTGGGTTCTTACAACGACGCCTACTGTGGGAACCGCTGTAC
AACCTTCACAAACTGAGTCTCTACTATCTACCAAGAGCCAGCGACCTCTACTTCGGACCTGTATTTAGAAACCTGTCT
AACTTAGAGACTTTAGAAATAAGTTTACTCCACGACTACTCCCTCAGTGTTCAAATGTTCCGGCCGCTGTTGCTAACT
CTGAGACATTTACACATACACGCCCCGATAGCACCCGGCCTGCTGAAGTCACTCACACATCTGCAGACGTTGGACGTC
GACCTTGTTCCATTCCCCGCCATTGTGGACGTTCTGCCTGAACTCCGACACACAGAGATTCAGGAGTTGTCCTTTGAC
ATGCTGGGATTCGGAATGGACAAAATAACATCTGATACTCTAGCGGGATTAAGAGGTCTGAAACATCTAAAAGGTTTG
TTTTTATATCTAGATATTTCAAAAATTGAAACTGTACAATCTAACGCCTTTGTGGGTTTCTCACATGTACAAAGGCTG
GATTTGACTAACGGTTTCCTCAAGACCTTACAAGACAAGACGTTCCGTGGACTGTCTTCACTCACTCATCTTAACTTG
ACACATAACACAATTTCTACTTTACCTGCGGGCGTGTTTGAAGGACTGACCTCTCTTACTCATCTTGATTTTAGCAAA
AACAACTTGATAGCTTCCCAGGCTCAGCTTCCAGATACCTTGGACTATCTTGATCTCAGTAACAATAGGTTGAACAGC
AAATTCACAGGTCAAATTCCATGTGCCTACCCATTATCATTCAACTTCAGTGGTTTAACAAGCGTGAatttctgaac
tnccacatacacaccgacacaccaactacgacaacaaaccatccaggcgttctcgacttattctgttcactaacagac
acacagacaagcacCGTCGCATAACCTTCTCGACGAAACCATTCGCCGCGAAGGTAACAATCGAACGACAGGGACAAT
CCAACAGAAAGAACATTCTTTGGCATAGACTGTAGTCATGGTTTCCGACCCAACCGCCTCTTGGCTTGTGTGCTCGCC
TCTTCCGGCATCTTCGTCGCCATGATGACCATTTTCCTGGTCGACTACAACATCGGCCATGTTCAGTACTACCTGTGG
ACGTGGGCCAAGTGGAGGGGACCGAAGATTGGCGAAGAAAAGAACCAAGAGCCGTCCAAATACACAAACGATGCTTTT
ATTGCTTACAACAACCAGGACGTCATGTGGGTTGTAAATGAAGCTATAGAGAATCTGGAACCTGACTTCAGTCTGGTC
ATACACGAAAGGGACTTTGCAGTCGGTGCTCCCATTGTGGAAAACATCGCGGATGCCGTGGAAAACAGCCGGAGAACC
GTCTGCCTCATCACCAGGAACTTTCTGAAGAGTAAGTGGTGCGAGTACGAGTTCCAGTTGGCCCAATACCACATGTTT
GAGGCTGGAGGAGGGAAGCGTCTCATTCTCGTGTTTCTGGAGTGGATTCCTGACAGAATGCTGAAACGGTTCCGCCAT
CTGAACGCCGTAATGAAGAGGGACACGTACCTGGTGTGGCCGGGCGACGTGCGGAAAAGGCCGCTGTTCTGGAAGCGG
CTGCGACACGCTCTGGGCGACCCTCTACCCCGGGACCCAGAGCCTCAGCAGCAGGTACACAATCTGGAGCGAAACATT
CCGGAACAAGATCCGGAAAGGAACATTGTGGAAGTACAGATCCATGGCCCCATGTGGAACATTCCGGAACAGCATGCA
CTGGCAATACAAGTACATGTTCCAATTAATGGGAACATTCCGGAACAGGAGCTGCAAGCTCCGGAACGGAACATTCCG
GAACAGGATGATGGAATTCTATTACCAGAGCCAGACGATCAATGGTTTTGTGGCAGGGAAGATGGTCCCCTCCTACCA
ATGTAG
>Bl12652
ATGGAGTCTAAAACCACTTTTCTCGCAGTCGGTTTAGCCATCTTTTCGGCTCTCTTGATCCCGCACCAAGGACGGGTT
ATAGGACAACCCCTCCCACCCGTCTGTCAGATCTGGAACTCAACAACTGTGGTGTGTAGAGGGGAGTATCCATGGGGG
AAACTAACCCAGTCGACGCTAACCCAAGTACCGCCCGCCATTCCCAAGTCTGTCGTCACCCTGGATCTGAGTCTAAAC
GAAATCAGAACACTTCATAACGGTTCGTTCAACGGGCTAAGAAACCTGACGTATTTAGATCTGTCTGATACGGACCTT
CACACCATCGAGATCGGTGCCTTTGCTGAACTGGAGAACTTGGAACGACTGATCATCTCCAAGGCAACTACGGAATAC
CCGTTTTCTCTTCAGAATGGACAGGTGTTCAAAGGTCTATACAATCTGAGGTATCTAGATCTTAGTGTGAACTTTGTA
AGCAACTTGCCCGATCATAGCTTTGATTACTTGACGTCATTGGAAGAAGCGAAGATAGTTGAATTCGGTTGGCCAAAT
GAGGATCGGAATACAACAATAGAAAGAACCCGCGATGGAAGTGGTTTCTTACAACGACGCCTGCTGTGGGCACCACTT
AAAAAACTGAAAACTTTACGTCTCTATTATCTAAAAAGAGCCAGTGACCTCTACTTTGGACCTGTATTTGGAAACCTG
ACAAACTTTGACACAATACAAATAAATTCGTCTGACACGGTCCATTTCGATATCCAAATGTTCCAGCCACTGTTATCA
ACTCTGAAACATTTATCTTTGCAAATGAATAGAATTACTCTACACATACAGCCAGAGCTCCTGGCGTCACTCACAAAT
TTGCAGACCTTGGAATTCCCCAAGTGGGTCCCATTTTCCTACATTTCGGACCTACTTCCTGAACTCAACGCCACACAA
ATACAGGAATTGGCCTTTGCTACTCGAGGGATCGACACAATAACACCCGATAGTCTGGAGGCAATAAAGGGTTTGAGA
GATTTGAAAGCTTTGTCTTTACGTTCTGTGCAAGCTATAAGAGCCAACTCCTTTACGGCTTTTTCATACTTACAGAGG
CTGCGTCTGTCGGAAGGATCCTTAGAGACTTTACCAGACAGAGCGTTCAGTGGTTTGTCGTCACTCACCCATCTAAAC
ATGCACGATAACCACATTTCTACTTTACCTCTGGGTGCGTTTGAAGGACTGTCGTCCCTTGACCACCTTGATCTGAGC
AAAAATCGCCTGGAAACCTCCGAAGCTCAGCTCCCAGAGACCTTGGACTACTTGGATCTGAGTCACAATGATTTGAAC
AACGACGTCTCGAGTTGGCATCTTCGCCGATGTTCCAACCCCATATCATTCAACTTTGAAGGCGTGAAAAGCGTCCGG
TATCTGAACATGAGTTATAACAAACTTGCCTTTGTGGACATTGAATGCTATCCTGGGAGCGTGGTTGTGCTCGATTTG
CAACACAACGGCATATATTACGTTGGTGGCATTGATATCAACCCTGTCTTTGTCATCAACAGTTTAAAGTATCTCGAC
CTTTCTAACAACGACATCAAGAACAGCTGGCCCGATGTAAGAATACCAACAACAACTCTAGAAACGCTCAAGATGGAA
AACAATGCCATCAAAAGTGTCAATTGGGGACATATGGGAGGGCTGGTTAGGTTGAAAATGTTGACTCTCTCTCACAAC
CACATCAGTTTTATTGGGAGAGGAGATTTCCAGTTTTTGGTCCAGCTGACACATCTGGATCTCAGTCATAACTACATC
AACACCATCAGATCATCCGCCTTCCGTGGACTATCGCGGCTACAATTCCTGGATCTCAGTAACAACCAAATACAGAAT
ATAACAATGATGAAATTTGAACATTTGGGCAACCTTACATATCTAAATCTGGCAGCCAACAGAATAGCGGTGATAGGA
GACGCCTTTCAACATCTTCTTGCGCTGAGGAACTTGAACCTAAGCAGCAATAGGTTATCCGTTTTGAACCAAACCATA
GTGGGTCCCATTGTCCAGCGGTTAGAAAATCTTGACGTTGCAGAAAATCCGTTTCTGTGTGACTGTAACTTATTGTGG
TTCGTTGAGTGGGCCCAGGACAGATACGACAGGGTGGTGAACTGGCACAATCCTTACAGTCTCCCTAATCGACGCTAC
ACGTGTTCACGCCCAGCTAAACTGAGCGGACAGCGCTTGATAGACGGAATGGCAAACAGAGAGGGCCATAACTACAGA
CAGGAGCCAGCAGAAACAAGGTTCTTTGACACAGTCTGCATTCATGGATTCCAGCCCAACCGCCTCCTGGCTTGTGTT

CTCGCCTCTTCTGGCATCTTCGTCGCCATGATGACCATTTTCCTAGTAGACTACAAAATTGCCCGTGTTCGGTACTAC
CTGTGGAAACTGGCCAAGTGGAGGAGACCGAAGATTGGTGAAGTAGAGAACGAAGATCCGCCAAGATATACACACGAT
GCTTTTATTGCCTACAACAACCAGGACGTCAGGTGGGTTATAAAGGAAGCAATAGAGAATCTGGAACCTGACTACAGT
CTGGTCATACACGATAGGGACTTTGCAGTCGGCGCCCCCATCGTGGAAAACATCGCGCATGCCGTGGAAAACAGCCGG
AGAACCGTCTGCCTCATCACCAGAAACTTCCTGAAGAGTAAGTGGTGCGAGTACGAGTTCCAATTGGCCCAGTACCAC
ATGTTTGAGGAAGGGGGAGGGAAGCGTCTCATCCTTGTGTTTTTGGAGAGGATTCCTGACAGAATGTTGAAACAGTTC
CGCCACCTGAACGCCGTAATGGCCAGGGACACGTACCTGACGTGGCCGGATGACGGGCGAGAAAGGCCGCTGTTCTGG
GGGAGGCTGCGAGACGCTCTGGGCGATCCTCTTCCTCGAGACCCAGGGCCTTATCAGCAAGAACAAGCTCCAGAACGG
AACATTCCGGAACGGCAGGCACAAG
>Bl56664
ATGAGGGGTATTCTGCTCTTCATGTTCATCTGTTATGTCGGCTGCCATGGCAACGCAAACGTTGCTACGACAACGGTA
TCGAACATACGACCTACCCTCAGCAGCAACGGTGACGTTCAAGTCTTTCGTAGTCAGTTGTGGGTTTGTGCACTTCAA
TATTCAATTTCAACGTACACATGCACGGGTTCAGATATAACTACCTTATACATCGCTGACGTTGACTTCTTCGATGCA
ACAGTCACTAAGCTGACTGTAAACTGTATATATAAAATCAACCACTCCAATTTTACTGTTTTTGACAATCTACCATCA
TACATCAAGACAGTTCGACTGTGGGATTGTTTCGAAGAGGAGATAGGAAAGGAGGTCCTGCTTGGGTTGGCACATGTT
GACTTTCTAGATATAGGAAACATCCTTTCTATATTCACCGATGGTTATGACCTCCACACTGAATTCTCTCGGATAAAA
GAACCCCTGGATCCAGTGAGGTTGGACCCTGACCTTTTTGCCTCTGTCCCAAAGTTAAAGAAACTGTCTATCCATTGG
CTATACATGGATGCATTTCCGGAAGCCATCTACCATCAAACGAATGATCAAGCTCATCTTAAAAACTTAGAGGAATTG
GACCTTTCTTTTAACGAAATACCAGAAATAAAGCCCGAGTACTTCAAGAATATGCCAAAGCTAAGATCACTCAATTTG
TCAGTCAATCGAATTAACAACTTATCTGACTCATTTTTGATGATGCAAAATCTACAAAGTCTTTATCTAACAGGTGCC
TTCATAGATAGTTTGGATGGTTCCCCCTTCAACGCCTTACCAGAACTAGGCACTCTAGTGCTGTCGCTAATGCGCCCT
ATTAGAAATGTCACATTACCAGGACCCGCAGGTCAAAAGTATCCTGTCAGAGGGTCTCTTCAGTTTATATATCCAAGT
TCGTTTGCTGGACTGTCAAATCTCAGGATACTTGATTTACATCACCATAGCATTCTGCTGATACAAAATGGAACGTTT
CTGGCTCTTGGCAGTCTTGAACAATTGGATCTATCCGTTGGACTCGTATCAGCCATCGAAGAGAACGGATTCGAAGGG
TTACAGTCGCTAACATCGTTGGACCTAAGCTATAACAATCTTTCACGGATAGGGACTTCACTCTTCCACAACTTGCCA
GCTTTGCTCTCTCTGAATCTTGAAGGGAACTACCTAGATTCACTGTCGAAGAACATGTTTCTTGGTTTACCCCTCCTA
ACGTCTTTAAATCTCGGCTATAATAATTTCATTGATATTCCCAGCGACGTCCTCCGGCCTCTTGTACGATTAAAATTG
TTATCAATCAATCATAACTACCTGAAGTCTGTCGAAGGCCTTCTGTCGGGTTTATCGTCTACATATTGTGAAGATATC
GACATTTCATTCAACAACATATCAACTCTAGACGCATCCATTTTGCCAACGCTCGCAGTAGCGGAGAGAATGATCACT
CTTAATCTGTCACACAACGCAATAACGACTGTGTATGCCACAAACTCCTACTTTGACATAAGTGCAAGCCAGTCATTG
AAGCAACTTAGACTCACACTAGATTTGCGTTGGAACAGACTTCTATCGATTCCTTTTCAGCTGGATAGATTCAGCTTT
TTCAGTTTGCCTAGGGTTGTTATAGACGGAAATGATCACTATAGGTATGCCAAAATCGGCCTGCATCATCAGTTGACG
CTTCAAATACGTCACAACCCCATCATATGCGATTGCACGATATATGAGCTCTTACAGAACATTGCAACCGCCCAAAGA
GGAGTTCTTTATGCAAATACAGATTTCAAAGAGCTGACTTGCCATGATCCTGAAGACTTGCGCGGAATAAAGCTCGAA
GATATCAGCCCATCTCAANGATGACCTAAGCCCATCTCAAACATGGTGTCAGAAGTGGGATTCGTCCGACTGTCCTTC
AGCTTGCGATTGCATGGTTCAGGGAGAACCAAATTCAACTATTTCACCTTGGAATGAACTTGTCGACTGCTCGGGAAA
GAACCTTTCCGTCATTCCCGACGGGATTCCAAATGCAGCAACGATCCTTCACTTAAGGAGGAACAACATCAGGAGAAT
TCAGCAAACCGCCTTCAGGAAAGCTCCTCTTACCAGGGAGCTGTATCTTAGCGATAACAACATTACAAAGATCGATTC
AGCAGCATTTATCCACATTCAGTCGTTAGAAATTCTCTATTTGGACGGGAATGATATTGAAGAAATCACAGGATATGA
GTTCGTGCATTTAGAAAACTTGAGAGAACTTTATCTGAATAGTTCAGAGGTTCGAGCTGTTAACAGGGACGCCTTTGG
TCATTTACCATTATTGGAAGTATTGAGTTTGAAAGACAACCTGTTGAATACCCTTCCCGACGGCTTGTTCTCTGCTTT
ATCGAACTTACAACACTTGTCCTTAAGCGAGAACAGTTTTCGATGCGACTGCGACATTCTGTGGTTCAAATACTGGAT
CCGGAACAAAGAATCGCTCCTAAAAGAAGAAAATATCACATGTACACACGAACATAACCAAGTCCAAGAAAACATCGA
AACTCTTTTGTCAGATAGCCTTGGTTGCGATCTTCAAAACACAGAGAGGAAGAGTAAAATAACCATTGGCTTTTCTAT
TGTGGTTGGTCTCCTTTCTTTAATTATACTAGTTGGATTCGTGCTGTATAGAAAGAAGGGAGACCTTGAAGTTTACCT
TTATTCTCGGTACGGATGGCGGTTCACAGAAGACAACGAAGACTTGGACAAACCTTACGATGCCTTCTTATCCTACAG
TCAACATGACCTTGACTTCATCCTCCAGCACATCCTACCTGGCCTTGAGAACAGGGAACCTCCGTTCCGAGTTTGTCT
TCACCACCGTGACTTTATCCCGGGTGTTCCTATCGCTGAGAACATTCTAAACGCTGTTGAGGAGAGCAGGCGAACCAT
TGTTGTTGTCTCCAGAAATTTCCTGGACAGCGATTGGTGCCAGCTGGAATTTCAAGCGGCGCATGCGCAGGTTTTGCG
AGAACGGGCCAATCGTTTGATCATGATTCTTTTGGAGGATATACCTGCTGACGACGCCCCTCCGGATATAAAACATTA
CCTACAGACGAACACGTATCTGAAGTGGGGGGACGAACGGTTTTGGGAACGGCTGGTCTACGTCATGCCCCGACCAAT
GCATTCCGAGGAACTGGTTCAAAACATGGAGCAAGAACCTTTAGAAATACCAGAGCCGAGTCAACAAACTACTACCGT
TTAA
>Bl20861
ATGCCTCTTTCCTCTCTCGCAGGTCCTGAGACGTTCCCGCGCCTTGACAACCTGAAACTCTTGGATCTCAGCAAGAAT
CGCATAAAGATCATTAAGTCAGGAAGTTTCAAGAACACCTACAGCCTGACAACCCTCCATCTGAACCACAACAACATC
TCGACCCTCCCGAACGGCGCATTCGACGGTCTCGCGTCGTTAGAAGTACTCGGGCTGGCTTTCACTCGCCTGTCGAAT
CTCAACAGCGTGGGGTTCCTCACACCCAATCTGAAATCACTCAACTTCACGTCATTCGCGGGAAACGGTGTCACTCAG
TGCAAACTCGGAGACGAGTTCAGAAATTTAAAAAGGCTGACGTACTTAGATCTGGCTTATAACAAGATCTCCGACCTT
GAACGGGACTGTTTTGCATCCCTGAACGAATCGCATGTGCAAACACTAGACCTGTCCTTCAACAACATCAAAGTTATC
AGTCATCCGGTTTTCTGGCCATTTCGTAACCTATCCTTCTTGTTTCTCGGCGGAAACAAATTTGACCTGACAGAGTTG
AACACGACCTTTGATAATATCAACAGCATTGACACTATGTCCTTGTATCTGTCATTCTATAACAGCACATTTCTAGCT
GGAGCCAAAACTCCTTTAAACACCACATTTGCTCGTTTGGCAGCCCTGCCAATCAAACATCTAGATCTGTCGTATGTA
GGGCTACGAAGGCTACCAGAAATAGGCTTGTTCTCCTTTTTCCCTCGACTGGAAAGTCTCCTCTTGGTTGGTAATAAC
ATAACCGAGTTGCCACCAAAGGCATTCATTGGCTTAGGTAGCCTGAAGACACTAGATATGAGCAAGAACAAATATTTC
CCAAACGTCAAGAAGGGCTCGTTTGCATCACTTGCCAACCTGACATCCCTCTCGCTAACTCTTGCAAAGGGTCGACAA
GACATACCCGGCAGCGTATTTCTCAACCTTCCCAATCTTCGCTCTTTAGCCATTTCGGGACCTAAGGGAAAGTTTGCC
GTAGGGAAACTCACCATGTGGAGTCTGCGAGGTTTGTCGTATCTGGAAAACATAATGATAGAATGGCACGATCTTCCT

GTTGTCCCAGGTCCTGCACTGGAACTTGTCAAAGGCACCCTGCAAAGACTGAGCTTTCTTCATGGACACATTTCCGAA
ATAGAACCAAGGGCGTTTCGCGGTTTCAAACATCTAAACACGCTTGACTTCAGATACAACAAGCTAGACGCTCTTCCA
GATTATGCATTTGATGGTCTTGAAAACCTGATCAGTCTAAAGCTGTCCTACAATTACATCGAGAAAATTAACAAAACG
GCGTTCTTCGGTCTTGTTAGATTGGAGATATTATACTTAGAAAGCAACCACCTCTGTTTCAGAGCAGAGACTGACTTC
CCTCCTCCATTCACTGCTTTACAATCCTTGATCTCCTTGCATCTGGAAGAGCAGAGCTCCAAGTGTCGTTTTTACGGA
GGAATTTTGTCTTTTCCCCCTGACTTCTTCTCAGGACTCGTCTCGATAAAGAGACTTCATCTGAGTAAAAATAAGCTA
AGAATGATGTTGTTCCGTGAGAAAACTAGCCGCCCGTTTGCTAACCTTACGACTCTAGAAGTGCTCGACTTATCTTAC
AACAACTTTGATACCATGACAGCCCTGCCATTTGAAAACCTTAAAAATCTAACAAGTCTATACCTTTCTTATAACCGT
ATTCTGAAAATCCCTGACAACCTATTCAAATCAGTGCCCAACCTGCGAAAGCTCAGGCTAAATTCTAACCGTAACTTT
GGAAAATTACCCAAAGACGTGATCTACCTTTTGCCTAAGCTTGAGCGGTTGGACTTGGAAGACAATCCCCTACAGTGC
ACCTGTGAAGAAGAGTGGTTTCACGATTGGGTGGTGAGTAACCGAACCAAGACACTGTTTTATAACTTATCAAACTAC
GGTTGTGTGTCGCCTGAACGTCTTGTGCACAAGACTATCTTAGACTTCGACGCTGAAGCCCAAGGGTGTAACGACAAG
ACGGGGCTTCACGTAGCAATATCCGGAGCGATCCTCCTTTGCGTTTTTCTCGTGGGTGTTGTTGTCGGATACCGTAAC
CGCTGGTACATCAAGTACGGCTGCTTTGTGATCAAGGCCCGCTACCACGGCTACCAGACATGGACAAATGAGAACTTA
CAAAAGAAGTTCGACGCCTTCGTGTCCTACAACCACAACGACCGCGCGTGGGTGATGAACGAGCTGGTACCGCATCTA
GAAGAGGACGGGGAAGAGTTCCGGCTCTGCCTGGACTATCGTGACTTTGTTCCAGGCGCGCCGATCACCGACAACATC
GTCAACTCCATCTACGACAGTCGCAAGACAGTCTGCCTGGTGACGGAGGAGTTCCTGAAGAGCGAGTGGTGCGAGATG
GAAGTACAGATGGCGACGTACCGACTGTTCGATGAGCAGATAGACGTGCTGATCCTGGTGTTCCTCGAGGACATCCCT
GACCGCGCCCTCCACCGCTACCATCGCCTGCGCCGCCTCATGTGCAAGAGGACGTACCTGGAGTGGCCCAAGGACCCA
CAGGAGAAAGCCCTGTTCTGGGAACGTCTTAAAGACGCACTGAAGACGGGAGACAGGCCGCCTATTGAGAACATCATC
TGA
>Bl48785
ATGGAGATAAAATATCCTGGTCTGATGCTGTCAGTAGGGTTGCTACTGCTACTCTTCAGCTGCAGCTGTAATACAGGT
GCTTCAGCTGAAACACCGTGCACCTGTGAGCTGAGGCATGGAGAGGGGGGAGTGCCACAGTCTTCAGCGTTCTGCCAG
TGTCCCACCATCAGTAGCCTGTCGTCACAGGACATCCCCAACTATGACAACATCACAACCATGGTGGTATCTTGTCAA
GCTGTAGACAGCCCAGCAGACCTATCACTGTCCGACCTAAGCTCTATCCAAAGCCTCTCTCTCTACAACTGTTTCTAT
GTGGTACTGAACTCGACCTTCAGGAGTCTACCCAGTCTGCGCAAGCTGGTGCTAGATGGCTGCGACCTTGACAATGAC
CCCTGTAACTACTTCTGGGTGCGGAGCGACAGTTTTTTGGGACTCCCAAACCTGGAAGAGCTCAAGCTCCTTGCCGCG
GCCGACGTGCCCAAACTCCGCGGACTCCCAAGTCTGGAATCCCTCATGTTGGCGTACATTTACCAGGGAGTACTAATC
AAGAACTATACACTGGTCATACAGTACCCTAACCTGTTACAGTTCACCCTTACTGGCAGTGTGTCAGAGACTCATAAA
GTGGACATACTACTGCTAGACATGCCCCTATTGGAAAGAGTGAATATCACATACAACCCTCAATCTCTCAATTGGATT
GTTCTTCCAGAAAACTTGTTCAAGCACACACCGGGGCTTAAGGAACTGTCTCTTACACATGATAGTATCCGTGTCTTA
AGTCAGTTTGTTCTCCATCCTGCAACGAACTTAACAAAAATCAACCTCTCGAATAACAACATCCGCTACGTCCCCCCT
CGTTTCTTTGAGGGACAAGACAAACTTGTTGAGGTTGACCTGAGTGACAACCAGATTTGGTACATCGCCCATAACACA
TTTTCCGACTTAGCTGCAATGGAGATCTTAGACCTTAGCAACAACGTTCTGACAAGCCTAGAGACAGGCACTCTTAAA
GGTCTCTTCAACCTTGGAATCATAGACATGTCAAAGAACTCCCTCGGACACCTACCAGCCGATATCTTTGCTGACTGC
ACGAATCTCACCGAGCTACAGCTAAGCAACAACAACATCTCCTCCCTGGATGACACAATCTTTCATCCCCTCACCAAA
CTCGAGGGCCTCTATCTGAACTACAATGAACTGCAGCACATCAGTGGGAGGCTGTTTCAGAACCAGACCAGCCTACAG
GTGCTCCAAGTAGGGGTGAACCGGCTACAGGCAGTTGACTTCGCCTGGTTTTCCCACATGTCCGCTTTAAACCAGCTG
CTTCTTCCTATCAACAACATACAGACTGTCTCGTCTTGGGGGGTTCTTAAGTCTTCCTTCCGTGGAAACTTACAAGTT
GACTTGGTAGCCAACAACATCAACCGTTTACCTTATCATGAAATCATACAGTTGGCATCTTTAGGTCAAAGTAAACCC
CAATTATCTCAAGTATTACTCAACTTCAATCCTTATAACTGTGACTGTGAGGTCCTACCGGTCAACAAAGTCCTATCG
TTACCCCGGGCAACGGAGACGTTCCCCGACTTGGTAAACATCTCATGTTGGAACCCACCTGAGCTAAGAAATCTTCAA
GTTTCACACCTGCCTCCCAGCTCCTTCAAGTGTTTCTTTAAGGAGCAGTGTCCCGAGTCTTGCTCCTGCTACAGTCAG
GGGATGAAATATACCGACGATGCTCTGCACAGAATTGTCAACTGTTCAGCTACAGGACTAACACAAGTCCCTTCGATG
TTTCCAGAAAACCTTACTTTGGTGGACCTTGGAGGTAACAGCTTTCCAAAATTGACTGTTCAAAGCTTTTTAAACTTT
ACGGACACCAAAACCATGATCTTATCTAAAAATGATATCTCAGCATTTGAGCCAGGAACCTTTAAGAACATGAACTCA
GTACGAATCCTTTACCTAGACGGAAACAACATTTCCACCATTGAAGAAGACACATTCCAAGGATTGGACTCACTCAAA
GTCTTGTTCCTGAATAACTCTGGTGTGACAAACGTCCACCCATCAAGCTTTTCACACCTGCGGCACCTGAAAGAACTT
CACCTGCAAGACAACGACTTAGAACACCTGTCAAAGCAAACCTTCAAAAGAGTATCAAGACTACAGGTGTTGTACATA
GGGCAGAATCCTTTGAAGTGTAACTGTGATCTGTTGGCATTTAAGGAGTGGGCGGAAACAAACGTCCAGTCTGTCTCC
TTCGAGTTTAACGTCACCTGCAGTAACCATGGCAACAAAGTGTTCACATCTGTAGCTCACGTTGTCAAGAGAGAGCTG
TCCTGTACACCTGGTAATCAGTATGTGTATGTCATCCTTGTACTAGGGATGGTAGTAGCACTGTTGGTTGTTGTACTG
TTGGTCTATCAGTATCGAGGCTTCCTACAGGTTTGGCTGTACATGAAGTGTGGTTGGCGTTTTGATCCAAAAGACGAC
GGCGACGACAAAACGTACGACGCCTTCATCTCGTACAGCAGCAGGGACGAACTTGTCGTGATACGGGAGCTCGCGCCT
GGACTGGAGGAGCGCGGGTTCAACCTCTGCCTGCACTACCGTGACTTCCCAGTAGGGGCGTGCATAGCAACAACCATC
ATAGAGACCGTGGAGACCAGCAGGCGGACTATCATCCTCCTCTCGCAGAATTTCGTGGACAGCGAGTGGTGTGCCTTG
GAGTTCAAGGCCGCACATCGACAGGTGCTGGAGGACCGACGGAACAGGATCGTGGTGATCGTCCTGGACGACCTTGAA
CTTCAGAACGTGGACAAAGACCTGCAGTTTTACTTGAAGACAAACACGTACCTGAAGTGGGGAGATCCCTGGTTTTGG
AGCAAATTGTGCTATGCCTTACCCAGGGTGGGGAGGGGGGCAGACAAACAGTCAACCGACTCTGAGCACATTGACATG
AAGGATGTCACAAGCCAAGACTCAGGCATAGAAATGACAGAGGTTTATGACAACCATTTATGA
>Bl24356
ATGTCAGGTGATTTGTCAGGTGGTAAAAGGCAATGGAGTTCTTGGCTTAGGGGAGGGGCAGCTGGTAAACACTGTGTT
TCTTGCAATAACTTGAATAAGAAAGGGCTATTTTTATCCTGGTATCAGTACACTGTGTTTCTGTGTTACAATCTTCAT
TTTCATAACCTTCGTTTTGTAACTAAACATTTTCACCTCATCTTCATTTGTAGGAGGCATCAAAAAACAAGAAACATG
TTCAGAGTTCTTCCTATCTTTGTGATCTTGTGCATTTGCGCCACCAGTGGTGGCATTACAACAGACACACCCAGAACT
GGTCCTATCCAAGGCAATGGCTCTCAGCCACCGCGATTGTGTGATTGCGTGGACGTAAGAGAACCTTCCTTGCCATTG
TTCCAGTTTACTTGCCTTTGCCCGGATATATTCAGCCTGAACAGTACTCAGGACCGTCTCTATGTGTCCAGATATCTC

ACCGCGGCTCCAGAATTTACTCACTTTACTGTACAGTGCTCAAACAGGTCCGTGATGTCTCCAAGTAGGCTGTTTGAC
ATGCTCCCAGCATCCATAAAGACAGTGGGGGTTAATGACTGTTTCCAGCATTATATCTCAAAAGATCTGTTCTTTGGA
ATGTCCCATGTGGAAGGGCTGTTCACAACGAACAATCCTTCGAATTTTTATGAAAAAGTTATAACAGGCATTTACAAG
ATATCCACTATAATACCAGTTGAACTTGACCCCAGGCTTTTTTTGCATGTGCCCCAGCTACAAGCACTTAACCTGGTG
TTGCTATTGCTTGATAGATTCCCTGAAGCTCTCTATCAAGGAGTAAATGGAACATATCCCTTGCAACGCTTAAGGGGT
CTTAACCTACAATTAAACAATATTGCATATCTTAAACCAGAACACTTACGGCACCTACCAAACTTAGAATCCTTGGAC
ATCAAATACAACAATATCAAGAATTGTTCAACATCATTTCCATTCCTGCCCAGTTTGCAAATGTTAATCCTAAATGTA
AACCAAATCAGAAGTTTGGACGGTTCACCCTTTCAAAATCTCAGTGCTCTACAGTCTCTAGACTTGTCCAGGCAACTT
GCAGTAGAAGGCCAAGCATCGAAAGTTATTTATTTATTGAATAGTATATATGTATCCAAGCATCTTAATTTTCATAGC
GAATTACCGGTAACCAATCTAACCTATAATGATGGCGTCATGGAGTCAATTTTCCCTACATCTTTTCTTGGACTATCG
GAATTGAAATTTCTTACATTGGCACAGAGCTACATTAAGTTGATTCCGAATGGTACCTTTAAAGATTTAGTAAAACTG
GAACAACTGAACATCTCTGATGGGCTTGTGGATCATATTGGAGAGTCAGCATTTCAAGGATTGGGTGTCTTAAAGATT
TTGGACTTAAGTTATAACAACTTATTAAATAGTACAATTGATCCTTCAATTTTTGAAAGTCTTAGATCGCTTCTCTCT
CTTTATCTTCAAGGCAATGTTCTTCAATCTCTTTCCCAGAAAACGTTCGAAAACACGCTTTATTTGGAAATCTTAAAC
TTGGGGAACAACCGTTTAACAACACTTGGACAATATACACTCCGTCCTCTACTGAACTTGAGAACATTTGATGTGCAT
GGAAACCAGCTAGTCTATCTTGAAGGCATGTTCCACGGTATATCAATTGGCGTTTGTGAGATAATTGATGCATCCTAT
AACAACGTGAAGGCATTGCAGAATCTTGGTGTTGCTGGTAAAACTGTAAATGTAGACCTATCCCACAACAACCTGGAG
ATTCTGTACACCACAAACTATTACATGGAAGAAAACAGACCATTTCTGTCCCTGAGACTTGATCTAAGGTGGAACATG
TTCACTTCGCTGCCGTTTGAACTAGCAGCCTACAGTTCGTTCAACAGATCCAGGATATCAATTCGGTTTCCAGATGAA
TCTCTTAACAGTTACAAAAAAGGTGGTGACATCTCTATCCTGATGAAGTCAAACCTCCTAGTCTGTGACTGTTTGCTG
TACGAGCTCATGCTGAACTTGGATGTCGCTAAACAAGGTGCCCTGTACACAAAAACAGATTTCCAAGACCTGGAATGC
GCATTTCCCGATGAACTTAGTGGTAGAAAAGTGATAGATGTCAGCCCATCCGAGCTGTGGTGCTCAGAGTTTTGCTTC
AAGCGACCATACTATCTTTGTTCCTGCTTTGAGCATGCAGGTGAGGTGCTCGCGTCAACGATACCCGGATGTTGGGTA
GAGCAGACAGTGTGTCCGTCAGAATGTTTTTGCTCGTACCAAGGAAAGCTGCATTCTTCTACTGCTCCTTACAATGAA
CTGGTAGACTGTGCCCAAAGGAACTTGTCTTCTATCCCAGGCAACATTACCAACATCACAACAATCCTTCATCTGGAG
GGGAATCACTTACGGGTCATCAGTCAAGATGTTCTCCCCCAACTTTTAATGATACGCGAACTTTACTTAAATGACAAC
AACATCTCATACATTGGCAATCAAGCATTCAACGACTTACTGTCACTTCAAATCCTTCGGCTCGATGGAAACAACATC
TCTGAGATAAACAGCACAGTGTTCAAGTCTCTCTCTAATCTACGCGAACTTTACATAAACCATTCCGGCATACGATAT
CTAGTAGCTGATACATTTCAGGACCTTGCAAGTTTACAAGAATTACACCTGGAAAACAACAGGCTGCAGTCTCTACCT
GAAAACATGTTTGCTGGTTTGAAAAACCTACGGTTACTCGGTATTCATGGCAATCCTCTCATCTGTGACTGTGACATT
CTGTGGTTCACGAACTGGCTCCGAAGTAGAGTATCCCTTCTGTCGCAGGCCCGTAACGTGAGCTGCTTTGCAAAGACA
AAAGTAAGACGGAACATTTTGAGCTTATCGTCGGCACAGCTGGACTGCGATGACCTGTTGGCAGCTCAAGCACGAACT
AGACTCATCGTGGGTCTTTCAATCCCGTTGGTTCTTGTGATGATCATTTTAGTTTGCGGTATCGTCCTCGTGAAGCGA
AAGGAAGCCATCCAAGTCTACCTGTATGCCAGGTACGGATGGCGGTTCAGGgaagaagaagaggaagacgaagaCAAG
GAGTACGATGCGTTCTTATCGTACAGCCAACACGACCTAGACGTGGTGATACATGACGTCTTGCCCGGACTCGAGAAC
AGGGATCCACCATTTCACGTCTGCTTGCACCACCGCGACTTTCTCCCGGGTATCCCAATAGCTGAGAACATCGTGAAC
GCCGTCAATGCAAGCAAGAGAACGATCATTCTACTTTCCAACAACTTTTTGGAGAGTGACTGGTGCCAGCTGGAGTTC
CAGGCCGCACACGCGCAGATGCTGCAGGACAGGGCCAACCGTGTGATCGTGGTCTTACTGGAGGACGTCCCCGAGGAA
AACGCCCCACCTGACATTCAGCATTACCTGAAGACGAACACGTACCTGACCTGGGGGGACGAACGGTTCTGGGAACGG
CTGGTTTACGCCATGCCGCGTCCAAGGCATGCGCAGCCTGTGCAAAATGAGGATGGAGATCAGCTGGCCTTGGTCGAA
TTGGACCACAATGCTTGA
>Bl18798a
ATGAGGACAACCACGCTGATTCTGCTATTGGTGCTGTATTCAGTGCAAATCCACAGGTTGGCATCAACAAGAAAAGGG
GAATGGTGCTCATACAAGAATTGGGAGTTCTCTTGTAAAAAGAAGGGATTAAGACATGTTCCTGTGTTAGTTCCTGTT
AGAGCAAGAGCCTTGGATCTTTCTATCAACTACATACCAGCACTTTACAATGACTCGTTTGCTGGTATGGATAAACTC
ACGATCTTGGTTGTGTCAAGGAACCTCATAATGGACATTGAAGCAGCAACTTTCTGGAACCTGAAAAGTCTAACATTG
CTGAACCTACGGGATAATAGGCTACAGTCATTACGTCCTCATGTGTTTAAAACTCTGTCCTCCCTGCAAGTGTTACAG
TTAGATCAAAATATGTTAAAAAGCACAGCAGCCATCAGTGCAGCATTACCCCCGTTAACTTCTTTATACAGCTTGGTT
TTAAGTAGCAACAACCTGTCACACGTCAAGCTAGAACCGGAGTTTGCCAGTTTAAACAAACTAACTGCTCTTCGCCTT
ATTGGAACCTCGATTTCATCTCTACAGACAGAGTCTTTCCAGGTGATGAAGAATCATTCCCTCAGTCATCTTAGCTTG
GGAGGTAACTATCTGGTCAACATACCGCGACAGGCATTAGATCCCTTTGCAAGCATCAAACAGATTGACCTTGCTAAC
AGTGACCTGACACCAAAAGACTTGTTTACTATTTTCAATAACACCAGGGGATTGGGTATAACAAATTGGACATTGAGT
GATAATAGCTTGACTGAATTGACAAATATCACATTTTCTCCGCTCATTGATGAAGAAGTCATCTACATTCGTCTAAAC
AGTAACAACATATCTCAGCTTACAGACTATCTCTTTGCTTATGTGCCTCAACTGCGAGTTCTTTCTTTTGGACAAAAC
CCAATTGAGAGGCTATCGGCAGGAGTGTTTGCTGGCTGTGACAAACTAGAAGAGCTGGGTTTAGACAATTGGGGTTTG
AAGGAAATTCCATCAGAATTATTCGAACCATTGAAAAACTTGACCAAGTTATACATGGATTCAAATCCAATACATAAA
GTCAGAGGCGACGCCTTCGTGTCATTGCCGAAGTTGAAGACGTTGCGACTGAATGCAAACAGGATAGAAACAATAGAG
AAAAATGCCTTCTATGGTCTACATCATCTTGAAGAGTTGAATTTAGGTTCCAACCAGTTAAGGCAAATTCCATATGTC
GCATTCAGTAAAGTTGGCCCGTCACTTAAGAAGCTAGACCTGTCGGCCAATCGCCGACTGGAAGCGATATCTCCGTTC
TCTTTCAACATGCTACAAAATCTGACAGACCTATCGCTTCAAAGCTGCAAGATAAAACGTTTAGGTACGAATGACTTC
GCAGGTCTCTACAACTTGCAAAATCTTGACTTGTCCTTTAATTTGATCCTGACAATTGACCCAAAAGCTTTTAATAAT
TTGTTGTCAATCAAAGTTCTCGATTTAAGCAAGAACCATCTTGGTCTATATATACGTGAAAATCCATCAATATCTCCA
TTTGTGAACCTAACTTCCCTTACAGAACTACATTTTAACTATCAAAAGTACACCGCAGGAGTATTTCCTGATGGCTAC
TTAGATGGTCTAACGTCCCTTCGGCTGCTTGCATTAATGTCACAGAAACTCGTATCATTGGAAAGCAAAACCCACAAA
ACAAGTATCCTGAAAAATCTGAAGAATCTTAGATACTTGCAACTTTCCCAGAACAAGCTCAGCAATTTCACAAGAGAT
GCCTTTGCAGGGTTGACAAATCTCAATTACCTTTATCTTACGTCAAACTCCATTAGAAATCTTCCACCTGGTATCTTT
AGGCACCAAGGTAGACTGCAATATCTAGATCTCAGTTACAATGGCATTGTTACTCTGAGTAGCATGGTATTTAGTCCC
TTGAAGTCACTTGTTGTACTTAACGTTTACAGCAACAGCTTTGCCTGTACCTGTGATATAGAATGGTTCCACGACTGG

ATAGCTTCAAGCATGAATGCTACTACAGGTGTACAAGGCGTCTACTTCGAAGCCTACACAAACTACACCTGTGCATCA
CCCAAAAGTTTGAGAAACAAACCTCTGATTGACGTCAACTTTGACAAACTTGGCTGCAAGTCCAAACTCGAAATTTAC
TTAGCAATAGGACTGAGTGTCACGTTTTTCATCTTGATGGTCAGCATCATACTGATGTACCGATATCATTGGTATGGA
CGATACGCCATGTTCCTACTGCGGGCCAAGTTCAACAAGTATGAAATCATCAGAGAAGAGGAGGAGAACCCCAAGACG
TACGATGCCTTTGTGGCACACAACAGCCACGACAGCGCCTGGGTCATCCGTCAGCTCCTTCCACAGTTAGAACGAGGA
GATCCGCCGGAGTTTCGTCTCTGTCTGGGTGATCGTGACTTTCAACCAGGGGCTCCTATCGTGGACAACATCGCCGAG
TCGATCTACGAAAGCCGCAAAACCATCTGTGTCATCACACGGAACTTTCTGGAGAGTGACTGGTGCAGGTTTGAGATG
CAGATGGCGACGTATCGTCTGTTTGAGGAGCACGTGGACTGTTTGATTGTGGTTTTCTTGGAACAGATTCCTGCCCAA
CGACTGGCCAAGTACCACTCCCTGAGGCGCGTGATGTGTAGGAACACCTACCTGGAGTGGCCCGAGGATCCGGAGGCT
AGGGATCTGTTCTGGGAACGACTTCGTGTGGCACTGCGAACACACAGGCCTCTTAATCATGATTTCAATTAA
>Bl18798b
ATGATGAAGAAGGCCAGACTGACGATGGTGTTGGTGATGTGTTCACTGGCAGTCTGCATGTCTGCTGCTACAAGGGGG
GTGCAATGGTGCTCGTACAAAACTGGGGTCCTCTCTTGTGTTAAAATGGGCCTCAGACATGTTCCTATATTAGTTCCC
CGACAAACAGAAGAATTGCATATTTCTATCAACCGCATACCGGCACTATATAATGATTCATTTGTTGGTATGGGTCAA
CTCGAAGTCTTGGATGTGTCTAGGAATCTCATAACTGATATTGAGGAGGGAACGTTTTGGAACCTGAAATCGTTAAGA
CTGTTGAATCTGGGAGGAAATAGGCTGACGGTATTAAGGTCACATGTGTTTAAGACCCTGTCCTCCCTGCAAAGGTTG
CAGTTAGACCAAAACATGTTTAAAAGCACCGCAGCCATTAGTTCAGCATTGCTGCCATTGGTCTCATTGAATGTCTTA
ACATTCAACAAAAACAAACTGTCACGTATCAAGCTAGAACCAGAGTTTGCAAAGCTGAACAAACTGGATATTTTGGAT
CTTGCTGAAATGTCGATTCCATCCCTGCAGGCTGAGTCTTTCCAGGTGATGAAGAATCACTCCCTCACTCATTTTAGC
TTGGGAGGTAACCGTCTGGTAGATATACCACAAAAAGCCTTGAAGCCATTCGTGAGTATCAAACAGCTAGATCTTGGT
TACAACAACCTGGCACCAAAAGTATTGGCTACCATCTTTAATAACACCAGGGGACTGGGGATTACAAATTGGACTTTG
ATGAACAATGCCTTGACTGAATTGACAAATGCCACATTCTTTCCACTCTTTGATGAAGAAGTCATCTACATTCGTTTA
GACGGTAACAGTATATCTCAGCTTACAGACTATCTCTTTGCATACGTGCCTCAACTGCGCGTTCTTTCCTTAAAGAAC
AACCCGATTGAGAGGCTATCAGCAAGAGTGTTTGCTGGTTGTGACAGATTACTACATCTAGGCTTAAACAGTTTGGAT
TTGAGTGAAATTCCGTCAGGATCGTTCGAACCATTGAAAAACTTGACCAAATTAAACCTGGATTCAAATATGATACAT
GAAGTCAGAGACAGGGCGTTTGTATCGATTCCGAAGTTGAGGATCCTGTGGATGAATGGAAACAGGATAGAAATGATT
TGGAAAAATGCCTTCTCTGGTTTACATCATCTTGAAGATCTGAACTTGGGTGCAAATAACCTAAGGAACATTCCCTCT
CGTGCACTAAGAGTAATCGGCCCATCACTAAAGAAGCTGGACCTTTCCGCCAATCGCAACTTGAAAACGATTTCACCA
AACTCCTTTAACAGACTGCATAAACTCGCGGTCTTAAAGCTTCAAAGTTGCCGCATAAGAAGTATAGGTAACAGTACA
TTTGCAGGTCTTTATAACTTACAGCGACTAGACCTGTCCTATAACAAGATTACGACAATTGATTCAGGTTCTTTCCAT
GATTTCTTGTCAATCAAAGTGCTTAATCTAAACGACAACGTTCTTGGAAGTCATGAAAGTTCATCTCCTTTTCGGAAT
CTGATTTCTCTTATCGAACTGCATTCTGATTATCAAAAGGACACGGCTGGAATATTTCCTGATGGTTACTTAGACGGT
CTGACATCCCTTCGGCTGCTCTCATTGAAGTCAGAGAAACTGATAACATTGGAAACCAAGACAGACAAAACCAGTATC
TTTAAAAAACTTAAAGACCTGAGATACTTGCTGCTGACCCAGAACAAAATCAGCCATCTCACGAGAGACACCTTTGCA
GGGTTGACTAACCTCACGTACCTGTACCTCGGTTCTAACTCCATCAGGGCTCTCCCGTTAGGTGTATTTAGAGATCAA
GGTAGACTGCAATATCTGGAGCTTAGATACAATGGAATTGTTACCCTGGCCCCCGAGGTCTTCAGTCCCTTGCAGTCA
CTAGTTATGCTCAATGTTTACAGCAACAGTCTGGCCTGTTCTTGTGCTATAGAATGGTTCAAAGACTGGATAGCTTCA
AGCATGAATGTTACTACAGGTGTACAAGGTGTCTACTTCGAAGCCTATAAAAACTACACCTGTGCATCACCAAAAAGC
TTAAGAAACAAACCTCTGATTGACATCAACTTTGACATACTTGGCTGCAAGTCCAGATTGAACTTTTACATGGCAATA
GGATCGAGCTGTACCATCTTGATCTTGATGATGACCATCACATTGACGTATCGATACCGTTGGTACGGACGATACGCC
ATGTTCCTACTCCGAGCCAAGTTCAACAAGTATGAACTCATCAGAGAAGAGGAGGAGAACCCCAAGACGTACGATGCC
TTTGTGGCACACAACGACAACATCGCCGAGTCAATCTACGAAAGCCGCAAAACCATCTGTGTCATCACACGGAACTTT
CTGGAGAGCGACTGGTGCAGGTTTGAGATGCAGATGGCGACGTATCGTCTGTTTGAGGAGCACGTGGACTGTCTGATT
GTGGTTTTCTTGGAACAGATTCCGGCCCAACGACTGGCCAAGTACCACTCCCTGAGGCGCGTGATGTGTAGGAACACC
TACCTGGAGTGGCCCGAGGACCCGGAGGCCAGGGATCTGTTCTGGGAACGACTTCGTGTGGCACTGCGAACACAAATG
CCTCTCGATCACGAAGTTAATGCCTAA
>Bl24343
ATGGCTAGATTTCACCCTGTCCTCTTATTGATAATTTACGCAATCATCCGTGACGTTGCCATGGACACGGCTGAAAGT
AGTCCGGACGGGGGAGACAGTTCACCTTTCGTGTGGCAGACTTTGTGCAATTGTAGCCAGACGAGCCCGGTTTCTTTT
GGATGCCTCTGTCCGGATCTGTCCAGTCTGACCAACGTGCAGGATCGTCTAGTTGTGTCTGGATATCTTTCTGCCTTT
CCGGAAATCACCGAATTCGGTGTGACGTGCAAACAGAAGTCATCAAGGATCACCAGCGGGCTGTTCGACATCCTCCCA
GCATCTGTTAAGAAACTGACGCTCGGTGATTGTTTCCTACACTATATAGGGAAGGAAGTTTTCCACGGTATCCCCAAT
GTAGAGGACCTCGTTATCGAGAACTTGAAATTGGGCGGGAACGATTCAGAATCCAGTCAGTCTTTACCACATGTTGAA
CTTGACCCACAGCTATTCGTGTCCCTACCACAACTACAAAAGCTCACCCTGGACTATCTTCTTCTCTTTAGATTCCCA
AAGGCGCTCTATCAAGAGGTCAATGGTTCATATCCCTTGCCAAACCTAAAGAGTCTCACTCTCCAAGGAAACCAAATT
CCATACCTAAAACCAGAATACTTACGGCACTTGACAAACTTAAGATCATTTGATATTGAATATAACCGTATTTCAAAC
TTATCAGCATCATTTCCATTCTTACCTGGCTTACGAATATTGAACATGGCTTTCAACCGAATCATCTCCTTGGTCGGT
TCCCCATTTCAAAACCTCAATAATCTACAGTCTTTGGATTTGTCTCAACAATATTCATTTGACAATGATACTAGTATC
ACTATAATCAATATAGGTGCTTTGGAATTCATTTTTCCCACATCTTTTCTTGGACTCTCAAAACTGAAATCTCTTCGC
TTGGCATGGAGCAAGGTTCGGTCTATTCCGAATGGCACATTTAAGGGGCTAGTTGAACTCGAAAAATTGGACATCTCT
TATGGGCTTGTAGATCATATTGGGCAGTCAGCTTTTCAAGGATTGAGTCTCTTAACATCTTTGGACTTAAGTTATAAC
AACTTAAGTTCAATTCATCCTTCAGTTTTCAATAGTCTAAGCTCACTTCTCTCTCTTTATCTGCAAGACAATGTTCTT
CACTCTCTCTCTCTTAAAACGTTTGAAAAGTTGCCTTATCTGTCATACTTGAACTTGGGGAACAACCGTTTAATCACA
TTAACACGTGAAATATTCAGTCCTCTACTGAACTTGAGAACGTTAGATGTGCACGGAAACCAGCTTAACAATATTGAG
GAGGTGTTTGCCGGGATAAATTCTGACTTTTGCGAGAAAATTGACGCATCCCACAACAAGTTGGAGTCATTGGATACC
TCGTCATTGCAACTTCTTGGCATCGCTGGCAAAACTGTAGAAGTCGACTTATCCCACAACAACCTGAGGGTTTTTTAT
TCAGCATATTACAGTTCTTCAGACGTGATACACCTGTCCCTTGCACTCGATCTAACATGGAACCAGTTTACTTCGCTA
CCGTTTATGCTATGGATATACGAGTCTTTTAATAGATCTAAGATATCAATTGCAAATGGTTTAGGCGGGGAATATGGT

GATATCTCCCTCCAGATGACTCATAATCCTTTAACATGTGACTGTCGGCTGTACGAGCTGATGTTGAACTTGGCTGTC
GCTAAACAAGGTGTCCTGTTTACAAAGACGGATTTCCGAAACATGGAATGTCGATACCCTGATACCCTCAGGGGTAGA
AGGGTGGTAGATCTCAACCCTTCTGAGCTGTGGTGCCCAGGGGAGTGTTACAACCGACCGGGCTATTGTTTCTGCTTT
GAGTATGAAGGTGAGATAATCAAGTCATCGTTTGCCAAATGTCAGCCGGAACGTAAGGCGTGTCCGTCAGAATGCTCG
TGCTCGTTCCAAGGACAGCTGCATTCTTCTACCGCTCCCTATAATGAGAAGGTGAATTGTGCCCGGAGGAACTTGTCT
TCTATCCCAGATGACATTTCGAACGCCACAACAATCCTTCATTTGGAAGGGAATCACTTAAGAGTCATCAACCAAACT
GTCCTCCCCCAACTTTTGATGATACGAGAACTTTACTTAAATGACAATAACATCTCAATTGTGGGCGCTATGGCATTC
AGAAACTTAACCCCACTGGAAATCCTTAGGCTTGATGGAAACAACATCTCCAAGATAAACAGCAAAGTTTTCAAGTCC
CTCTCTAATCTCCGAGAACTTTACCTCAATCATTCCGGTGTACGATATCTAGCAGATGATCAGTTCGACGACCTTACA
AGTCTACTGGAGCTACACTTGGAGAACAACAGGCTCCAGTCTCTTCCAGAAAACATGTTTGCTGGTTTGAAAAAGCTG
CGGTTACTCGGCATTCATGGCAATCCGCTCAACTGTGACTGTGATGTTCTGTGGTTCGCGAACTGGCTCCGGAGTAGA
ACTGGCTACCTTATGTCAAAAGGACGTAACGTGAGCTGCTTAACAAATACACAAGTAAGGCGGGATATTTTGAGTTTG
TCGTCAGCACAACTTGGCTGCGATGACCTACAGGCAGCTCAAGCGCGAACTAGGCGCACCATAGGTCTTTCAATTGCA
CTGGTTCTTGTGACGATCCTTCTCGTTTGCGTGATCGTTACCGTGAAACGGAAAGAAGACATCCAAGTCTACCTGTAC
GCCAGGTACGGATGGAAGTTCCGAGAAGAAGAGGAAGACGAAGACAAGGAGTATGACGCCTTCTTATCGTACAGTCAA
CACGACTTAGACGTGGTGCTACATGACGTCTTGCCCGCACTCGAGAACAGGGAGCCGCCGTTTCACGTCTGCTTGCAC
CACCGCGATTTTCTCCCGGGTGTCCCAATAGCCGAGAACATCCTGAACGCCGTCAGCGCGAGCAAGAGAACGGTCATT
CTGCTGTCCAACAACTTTTTGGAGAGTGACTGGTGTCAGCTGGAATTCCAGGCCGCACATGCGCAAATGCTGCAGGAC
AGGGCTAATCGTGTGATCGTGATTTTACTGGATGACGTCCCCGCCGAAAACACCCCTCCGGACATTCAGCATTACCTA
GACACGAACACGTACCTGAAGTGGGGGGACGAACGTTTCTGGGAACGGCTGATTTACGCCATGCCGCGACCAAGGCCG
CATGCGCAAAACGTGGACGGAGATCAGCTGGCCATGGTCGAACTGGATCACAATCGTTAA
>Bl19922
ATGCGTACCCGGCCAGTACTTCTCCTGTGCGTCTTCGTTGCGGTAGCTCCACAAGTAGTCCGACAGGTTACCGGTACT
ACACTTGTGCTCACGCCTGATGAAGCCACAGCTGCAAGTTGTACAGAGTGGGAGCCAAACGCCGACAATGTTACGGGA
TTAGCTACCGACTGCTCTCGGAGAAATTTCTCTGCTGTGCCGTCCAACGTCTCCTACTTCGTCGTGGTTTTGGACCTG
GCGCACAACCGGCTGTTCTTCCTAAACCGGGACAGCTTCAGCGGTCTCGCCCGTCTAAAGGTACTGAGTTTATCTTAT
AACCAAATACGCACAATCGAGCATGGTTCATTTTCGGATCTGTCTGAACTTTCCGAACTGTACCTAGATCACAACGGC
ATATCATCGATAGCAAATGGCTTTTTTACGGGTCCGTCTTGGTTGGAAAACATTGGTCTGTCTTTCAATCGTATCGCC
TACATCGAAGACTCTGCCCTCGACGAAGTTGGCACTACCTTGAAACAACTCAAGCTAGGAAACAATTATCTTGGCAGG
CTGTTATCCTTAGAAAAATCCTACCGAATATTTCAACAACTTGAAAACCTCACACACCTTGATCTAAGCAACAATGGA
ATCTATGACTTTAGGTCAGACGTATTACGATCTCAATCAAATATCTGGTCGTTGAATTTGTCAGGAAATGCTCTTCGC
GATAAGAGCCTTTTCATTGAAGTTCAAAATGTACATAATCACTCGGGCTCGGGTGAATACATGTATGCACAGCGAACA
TCAGTATTCCACGGTTTATCAAGTCTGAAATCTCTAGACATCAGCCACAATAGGTTTAAACTTTGCTGTGAGGCATAT
AACGGACTAACCAATTTAGCATATCTAGATCTACGGTCAAATGATGTATCGTACATTGAACCGGAAAACATAACAAAG
GCCTTACCGAATCTGAATGAATTACATCTCAGGGGAAACCCCTTTGACTGTAGCTGCAGGGCTGTGCCATTCTTTATT
AGGTGGGTTGATGACTCAGTTGTTGACACGGACATTTTGAACTGGACTTGTCAACGACCACCATCTGTGGCAGAGGCA
AAAGTTGCTGAGTTGGATATCCGTGGTTGCGGTCAAGACTTGAAGTATAATGTTTTGTTAACAGCATTCATTCCAATT
TGCGTAATTGCAATTTTTGCCATTGGAGCAGTTATCTTCTATCGACGAGCAAAAAGGGCTAATTTTTCAAAAATCGAA
GCGTTCGAAAACATTGAACTTGAAGACCGTAAATACGACGTCTTCATCTGCTACAGTTCGCACGACGCAGATTGGGTT
CACCGTTCACTACTCCCACATCTAGACTCGCTTCATGTTGAGTATTGTATCCATCTTCGTGATTTTGCACCTGGCGAA
ACTATAGTTGAAAACATCGTGTACGCTATAGAACAGAGTTGCCAAGTTATCGTTGTGATTTCGGGAAACTTTCTTAAT
AGCGAGTGGTGCACGTTTGAACTCCAAATGGCAGAGCACAGGCATTTCACCACTGGGGAAAAGTACATCATTCCCATC
CTACTAGATGAAGTACCCGTTAACACGATGCCAAAAAGTCTGCGATATTTGCTGGCAACAAAAACGTACATCGAATGG
AAAGGCAGAGGAGAAGAAGAAGACCTGTTCTGGCGTAGACTTGGGAAAGCTTTAAAGTCGAGGTTACTGATCAAAAGG
ACCAACAACGACGCCATTTACATGGAAGAAGAAGCCACAATCATGCCTGAGTCACCTACATCAACGCCAAAGCATCGT
TCTGACACCGTAAATCTCTGTTGGGTTAAGGCAGCCCAGGCTCGAAAATTCGTGTTCGAGTTCCGGCCGCGTGCCTGG
CCAGCGGCGCGGCCGAACTCGAACATGGTTCGAAATGTTCCACTACGGAGCATTTCGGGGAGCGCAAGGTCTATTACA
CAACACAATCATATGTATCATCTCAGGTGTACGTATAAGCACAAAATTGCTTTATAG
>Bl17405
ATGTCCTGGCTCTTAGTTTGTTTCCTGCTGGGTCATTCTGCTGGGATGTACAGCGTGTCAGCGATCAGAAATGACGCC
TTCCCCTGCTGGAGTTTAAACACAACCTTTGTGGACTGTAGTGCGCTGTACCTGGAACATGTCCCTCCACTGCCACAG
ACAACAACATCACTGGACTTCAGTGACAACAGGATTCGCAAACTAGCCAACAACTCATTCTATGGACTGGACAACCTC
CTGCAACTCAAGCTTTGCACCAATGGCATAACCTCCATGGAAGAACAGGCATTTGCAAACCTACAGCAACTGGAGGAA
CTGAATCTAAAAGAAAATCCACTGGTGTACATCCACCCAGAGGTTTTCCTTCCTCTCACGTCCCTAAGGAAGTTGGAT
CTATCCACAGGAAGGCTAACAGCAATTCCAGAGGCATTAAGAATGCTTCATAACTTGCAAGATGTCAATCTTGCAAGG
AACTACATCACATCAGCCAATCTTGACATTTTCAGAGGTATGTCTAAGATACAAAAGATAGATTTTCAGGGCAACCTT
ATAGTAAACATTTCAGCAATTGACTTCAGGATGGTTTCAAACAGTTCCCTGCGTGCACTGTCTCTCTCAGCAACTGTG
AAGAGTTTGTCTCACATACAGGAAGGAGCTCTGGCTGCTTTAAGGGAAGTACAGGAACTGAATCTTAGTGACATTGAT
GTCGAAGGAAGGATGTCTTTGTTTTTAAAGTATACTTGTGAGTTGACTTATGGCACAGTCAAGTTTCTGAACTTGATC
TATATGAGAATGTACATATATCGCAGTGGTTTCTTTGATTGTCTTCCACGTACAATCCAGACACTGTGGCTAGATCTT
AATATCATCGAAAATTTACACAAGAATTTATTTGTGAGACTCAAGAATTTGCAAGTTCTCAGGCTTTCTCAATGTTGG
ATGTCTAGCATAGAGATGGGTGCTTTTGATGGCTTAGAAAGTCTAAAAGAACTGCACCTCAGTGGGAACAAGTTGACA
GCTTTAGACCCACATGTACTTTCTCCTGTTTCCGCAAGTCTTGAGGTACTTAATCTGGGAAATAACCTGAACTTCCAA
CTACAACCGGGACATTTTAAAAACCTGACTCTCTTGAAAATTCTGGCACTACAAAACAATGGGATTCGTTCTTTCGAA
AGTGACCACTTTAAAGGTCTTGGTAATTTGATACAGCTGCAAATCGGGTATAACCAGGCAAACTACAAACCAACCAAA
GAAGGCATTTTCAGATATATTCCCAAGTTAGAGGTCTTTTTGGCAAAGAAAAATGATGCAGCTGAGTTTGCACTTGAT
GCTGTCTTATCTGCAGGTCTGGATTCACTAAGGGTTCTTGATCTAGACAGTGGGAGTGTTAACATTCTCAACGATAGA
GGCAGAAGCATAAATCTTTCAGATCTACAAGTTTTGAGAGTGGGAAATTCGGCTGATGACATTACAATAACATATGCT

TACCACTACAGGGCTGACCTATTATGGAATCTCACTGAGTTACAAGAGATAGATCTGTCTCAGAATGGGTTTCAAAAC
ATTAAAAAGGAAGCATTCTGGCATTTGAAGAAACTGGACACTCTGAACTTAGCAGGAAACTACCTGGCATCCTTGCAC
CCAAGCATGTTTCGTGATGCACACAGCCTGAGAGTCTTGGATCTTAGTTACAACCGCATTACTGCAATATCACCAACA
CTGTTTCAAAATGTAAATTATTTGCGACAGCTTAGCATGCAAAAAAACCTGATCACCTCCATAGGCCCACAAACACTG
ACGTATTGGAACAAGTTCAACACATTGTACGAGTTAGACCTGTCAGGCAATCCTTTTTCCTGCACCTGTCAACTCCTA
GATTTTGTAGAATGGGCAAGGAACAACACATCAGTCAGGATTCTGTATTATTCCTCTACTGGTGGTTACAAGTATTAC
AAGTGTTCCTCTCCTCCAGACCTGAAAAACCTTCCCCTTCTCGACTACAAGCCAGACTGTAAGTCCTACCTTGGATAC
TACACATGCATAGTCATGTCCACTTTGATTTTTCTGTACATAACCATGACATACGTGATGGTGAAATATCATGCGTAC
ATACGCTATCTGTATCAGTATGCGAGGGGGAAGCTGCGTGGATACCAAGCCATTCCGCACAGGCACAGATACGAGTAT
GATGTGTTTGTGTCGTACAACAACGAAGACATCCCCTGGGTCCAACAGGAACTGATTCCCCACCTAGAGGAAGTGGAG
CCACACTACAGACTGTGTATTGGCGATCGCGACTTCCTCGCTGGACGCGACATCACGACAAACATCGTGGAAGCGATC
CAGGGCAGTAGAAAGACGCTGTGCCTGCTGACGCAAAGGTTCGTACGCAGCGGCTGGTGCACGCTGGAGTTCAAGATC
GCCCAACACCGTCTGTTCGAGGAGGGAGAGGATGTGTTGGTTCTGGTTCTGCTGGAGGACATTCCGGCTCATGTGGTG
CAGCGCTACAACCGCCTGCGCCAGCTCATGTCCAGGAAAACATACCTCGTGTGGCCCGAGGACGAACGAGCACGGCCG
TTGTTCTGGGTTCGACTCAGGAAAGCCCTTGGGGTAGGAAATGTCTTACCATATGAAGAAGATGTGTAA
>Bl04519a
ATGGAGTCTAAACCCGTTCTTCTCGCAGTCTGCTTAGCTGTGGTTTCCGCGTTCATCACCCAACATGGACGGGTTGGA
GGATTAACCCCCGCACCTTGGCAACTCGACCCACCTGTGTGTCAGACCTGGAACTCAACAACTGTGGCGTGTACAGGG
GAGAATACATGGTCTCCAACCCGCGCACTTCTAACCCAAGTACCGCCCGGCATCCCCGAGTCTGTCATCACGGTGGAT
CTCCGTTGGAACAACATAACTGGACTGTACAACGACTCGTTTAGTGGGTTAAGAAACCTGAAATCCTTAGACCTGTCT
GGAAATGTTCTACAGACCATTGAGATCGGTGCGTTTGCTGGGTTGAAGAATTTAGAACGTATTAATATGAGTATAAGG
GGATGGGATGATATAGATGGATTGATATGGTTAAGGGAATTGCGACCCATGTATACTCTTCGAAATGGACTTTTCCAA
GATCTGCGAAACCTGAAATATCTGTGGGTGACGACTTACACTGATACAATCAGTGCGGGAGTGTTCACGGGGTTATCT
AACCTGAGGCATTTAGAGCTAAGGGTGAGGGATGTCAACATTTTACCCGATCACATGTTCGATTCTTTGACCTCATTG
GAAATTCTGACGATAAGGGAACACAGCAATGTCATGAGGGAGGATCACACCACCACCACTAGAAGTGGGTTCTTACAG
CGACCTCTGCTGTGGGCACCGCTGTACAACCTGAAAAAAATGAGTCTCCATTTACGGCCAAGAATCAGTAACTTCTAC
TTTGGACCTGTGTTTAGAAACCTATCTAACTTAGAGAGTATAGAGATAAAGGCATGGACATCTAACGACTATGCTCTC
AGTGTTCAAATGTTACGGCCTCTGTTACTTAGTTTAAAACATTTACACACTGACTTCCCGATAGCCCCCGGCCTGCTG
AAGTCACTCACACATCTGCAGACGTTGAACCTCGACCCTTTACATCCCTGGCTCATTCTGGACGTCCTGCCTGAACTC
CGACACACGCAGATTCAGGAGTTGACATTTCAGGCTGGGGTATCGGGAATGGACGTCTTTGCGGGAATCACTCACCTT
GCGGGAATAAGAGGTCTAAAAAAATTGCAAACTCTGTCTTTAACAGGATCCATATCTAAGTTTGACGGTTTTATGAAT
TTCTCACATCTACAAAGGCTTGACTTGGCGCAGAGCTCCTTGAATTATTTAGAAGACAAGATGTTCAGCGGACTGCCC
TCACTCACTCATCTTAACGTGACAGATAACGCTATTTCTACTTTACCAGAGAGCGTGTTCCAAGGACTAACAACCTCC
CTTACCCATCTTGATTTGAGCCATAACCGCTTGGAAACCTTCCTAGCTCAACTTCCAAGGACCTTGGACTATCTTGAT
CTCAGTTACAATAAGTTAAACAACAGATACATTGGTCAAACACCGCGGCCATATAAATGTCTTCCCCCTTTAGCATTT
GACTTCAGTGGTTTGAAAAGCGTGAATCATCTGAATTTGAGTCATAATGCGATTACTGATGTTAACGGCAATTGTCTA
CCTCGGAATATTGATGTGCTGGATTTGCAGCACAACAACATAGGGCAGATCTCGATCCTCTGCACTACGAGATATCTC
GACCTTTCTAACAATCATATCCACTCTTTTGTTGATCAAGAAGAATGTGAGTTTAACAATGCGTTAGAAACACTGAGG
CTGGATAACAATAATTTGGAAGATATATATGTACCGTTACCGTGGTTAATACACCTGAAAACGTGGACTCTTTCTCAC
AACCACATCAAACGTATTAAAACAGGACAATTCACAGTGATGGACCAGCTGGAACATCTAGATCTTAGTCATAACGAG
ATCAAAACAATTCAAAAATTCGCCTTCCATGGACTATCGCGGCTAAGATTCCTGGACCTCAGTGACAACAAGATACAG
TTTATAACGGAGATGACATTTGAAGGTTTAGGCAACCTTGCACATCTAAAGCTGGCATCCAATAAGATTGCAGTGATT
GGGAATGCCTTTCGCCGTCTGTATGAGTTGAGGAATCTTAATCTGAGGAGCAACAGATTAACTGTGTTAAATCAAACA
ACATTGGGTCCTGTTGTCAACCGGTTAGAGACTATTGATATTGCAGACAACCCGTTTTTGTGTGACTGTAACTTAATG
TGGTTTGTTGAGTGGGCCAACGACAAATACGACAGGGTGCCGAACTTGTTCAACCCTTACCCTGATTTTGCTCGAGGC
TACACGTGTGCTCGTCCAGTACAACTGCTCCGACGGCGTTTGATAGACGGGTTGACGCAGGAACAACAATCCAAAGTC
AAAAATGATCCATCAGAACGAAAGTTCTTTGACACGGTCTGTAGTCATGGATTCCGGCCCAACCGCCTCCTGGCTTGT
GTGGTCGCCTCTTCGAGCATCTTCGTCGCCATGATGACCATTTTCCTGGTCGACTACCACATCGCCCGTGTTCAGTAC
TACCTGTGGCAGTTGGCCAAGTGGAGGAGACCGAAGATTGGTGAAGTTGAGAACCAAGAGGCAAACAGATACACGCAC
GATGCTTTTATTGCCTACAACAACCAGGACGTCATGTGGGTTGTACATGAAGCAATAAAGAATCTGGAACCTGACTAT
AGTCTGGTCATACACGAACGGGACTTTGCAGTCGGCGCTCCCATTGTGGAAAACATCGCGGATGCCGTGGAAAACAGT
CGAAGAACCGTCTGCCTCATCACCAGGAACTTCCTGAAGAGTCAGTGGTGCGAGTACGAGTTCCAGATGGCCCAATAC
CACATGTTTGAGAAGGGGGGGGAAGAGGCGTCTCATTCTAGTCTTTCTGGAGAGGATTCCTGACAGACTGCTGAAACGG
TTCCGCCATCTGAACGCCGTAATGAAGAGAGACACGTACCTGACGTGGCCGGGCGACGTGAAGAAACGGCCGCTATTC
TGGGATCGGCTGCGACACGCTCTTGGCGATCCTCTACCCCGGGACCCAGAGCCTCAGCAGCAGGTACAAGATCCGGAA
CGGAACATTCCGGAACAGCAAGAACAGGCTCCGGTAAGGGACATTGTGGCAGTGCAGGTACACAAACCCATGCGGAAC
ATTCCGGAACATCAAATCCTATTACCAGAGCCAGACGACCTGTGGTTTGGAGATGAGGACGGCGTTCCTCTCCTACCC
CTGTAG
>BL04519b
ATGGAGTCTAAACCCGCTCTTCTCGCAGTCTGCTTAGCTGTGGTTTCCGCGTTCATCACCCAACATGGACGGGTTAGA
GGACAACTCCCCCCACCTGTGTGCCAGACCTGGAACTCAACAACTGTTGTATGTTCAGGAAAGGAATCAAGGTGGATA
CAATGGGAGCAGAAACCAACCCGCGTGCGTCTAACCCAAGTACCGCCCGGCATCCCCAAGTCTGTCATCAAGGTGGCT
CTCCTTTGGAACAAATTAACAGTATTGTACAACGACTCGTTTAGTGGGTTAAGAAACCTGAAATCCTTAGACCTGTCT
GGAAATGCTCTAGAGACCATTGAGATCGGTGCGTTTGCTGGGTTGAAGAATTTAGAACGTATTAGTATGGGTGTAGCG
GGATGGGATGATGTGATAAGTGGATCGCTATGGACAAGGGAATTGAGACCCAGATATACTCTGACACCCATATATACT
CTTCCAAATGGACTTTTCCAAGATCTGCGAAACCTGAACTATCTGCGGGTGACAATTTTCACCGATGCAGTCAGTGCG
GGAGTGTTCACGGGGCTATCTAAACTCAAGCATTTAGAACTTAGTGTGAGGGATGTCAACCTTTTACCCGATCACATT
TTTGATTCTCTGACCTCATTGGAAATTCTGACGATAAGGGAACACAGCAATGCCACGGGGGAGGATCAAAACACCACC

GCTAGAAGAGTCAACAGCGGAAGTGGGGTCTTACAACGATCTCTGCTGTGGGCACCGCTGTACAACCTCAAAAAAATG
AGTCTCTATTTACTGCCGAGAACCAGTAACCTCTACTTTGGACCTGTGTTTAGAAACCTGTCTAACTTAGAGAGTATA
GAAATGAATTCAGGGATATCTAATGCCCTCAGTGTTCAAATGTTCCGGCCTCTGTTACTCAATTTGAAACATTTACAC
ACTGACTGCCTGATAGCCCCCGGCCTGCTTAAGTCGCTCACTCATCTACAGACGTTGGACCTCGACTCTGCATATGAC
TTGCGCATTTCGGATGTTCTGCCTGAACTCCGACACACGCAAATTCAGGAGTTAACATTTCTGGTGGGGCTATCGGGA
ATGGACGTCCTTGGGGGAATAAGAGGTCTAAAAAAATTGAAAACTCGAACCCAGTGGCATACAATTGAACGGTTTTAT
GAATTTCTCACATCTACAAAGGCTTGACTTGACGCAGAGCCCGCAAAATGAATTAGAAGACAAGATGTTCAGCGGACT
GCCCTCACTCACTCATATTAACTTGACAGAAAATTATATTTATACTTTATCACGAGGGGCGTTTGAAGGACTAACCTT
ACTTACTCATCTTGATTTGAGCTATAACAACTTGAAAACCTCCCAGGCTCAACTTCCAGAGACCTTGGACTATCTTGA
TCTCAGTTTCAATAACTTAAACAACAGAGACATAGATCAAATACCACGGGCACGGGAATGTTTCTACACTTTATCATT
TGACTTCAGTGGTTTGAAAAGCATGAATTATCTGAATTTAAGTCATAATGAGATTACTGATGTTGACAGCTCTTGTCT
ACCTCGGAATATCACTGTGCTTGATTTGCAGCACAACAATATTGACGATGTACACAAGACTCCGAGGGCACGGTATCT
CGACCTTTCTAACAACAATATCAATTCTTTAAATTTATACTCAAGTAATGAACTATCGATTGTAGAAACACTTAGGCT
AGATAACAATGTTCTGATAGCGATAGATTCATTATGGATTCCACAAACTCTGAAAACGTTAACTCTTTCTCACAACCA
CATTCAGAGTATCAAAACAGAACCATTCCAATGGCCGAACCGGCTGGAACATCTAGATCTCAGTCATAACGATATCAG
CACAGTCATGCCATCCGCCTTCCGTGGGCTATCGCGGCTAAGATTCCTGGATCTTAGTGACAACAAGATTCAGTTTAT
AACGGAAATGACATTTGAAGGTTTGGGCAACGTGACACACTTAAACCTGGCGTTCAACGGGATAGCGGTGATAGGGGA
CGCCTTTCTCCGTCTGTACGGGTTAAAGGATTTGAACATGAGGGGAAATAGCTTAGCTGTCTTAAACCAAACAGCGCT
GGGTCCTGTTGTTAACCGACTAAAGAGGCTGGAAGTTGCAGGCAACCCGTTTCTGTGCGATTGCAGCTTACGGTGGTT
TGTTGAGTGGGCGGTGGGCAAATACGACAGGGTGCCAAACTTGTACAACCCTTACCCTGACGTCGGGAGAGGCTACAC
ATGTTCTCGTCCAGCACAACTGCGCGGACGTCGCTTGATAGACGGGTTGATACAGAAACAACAATTCAACGACAGAAA
GGATCCAGCAGAACGAAAGTTCTTTGAAGCAGTCTGTAGTCATGGGTTCTGGCCCAACCGCCTCCTGGCTTGTGTGCT
CGCCTCTTCGGGCATCTTCGTCGCCATGATGACCATTTTCCTGGTCAACTACCACATCGCCCGTGTTCAGTACTACAT
GTGGCAGTTGGCCAAGTGGAGGAGACCGAAGATTGGCGAAGTAGAGAACCAAGAGCGGCTCAGATACACGCACGATGC
CTTTATTGCGTACAACAACGAGGACGTCATGTGGGTTGTACATGAAGCAATAGAGAATCTGGAACCTGACTACAGTCT
GGTTATACACGAAAGGGACTTTGCCGTCGGTGCTCCCATTGTGGAAAACATCGCGGATGCCGTGGAAAACAGCCGGAG
AACCGTCTGCCTCATCACCAGGAACTTCCTGAAGAGTCAGTGGTGCGAGTACGAGTTCCAGATGGCCCAGTACCACAT
GTTTGAGAAGGGGGGGAAGCAGGCGTCTCATCCTGGTGTTTCTTGAGAGGATTCCTGACAGACTGCTGAAACGGTTCCG
CCATCTGAACGCCGTGATGAAGAGGGACACGTACCTGGTGTGGCCGGGCGACGTGAGGAAACGGCCGCTATTCTGGGA
GCGGCTGCGACACGCCCTGGGCGATCCTCTACCCCGGGACCCAGAGCCTCAGCAGCAGGTACAAGATCCGGAATGGAA
CATTCCGGAACAGCAGGAACAGGCCCCGGAACGGAACATTCCGGAACAGGCTCCAGTTAGGAACATTGTGGAAGTGCA
GGTGCATGGCCCCATGCGGAACATTCCGGAACAGCAAGATGAAATTGTATTACTAGAGCCAGACGACCAGTGGTTTGG
AGATGAGGATGACGTGCCTCTCCTACCACTGTAG
>Bl08928a
ATGTTCGTATTGTCCACAGGTTCACAGAGAGGGATTAAAAAAGAAATTGACATGGGGCCTAAACCCGCTCTTCTCGCA
GTCTGCTTGGCTGTGGTTTCCGCGTTCATCACCCAACATGGACGGGTTACAGGGCAAATCACCCCAGCTCTCGGCCCA
CCTGTGTGTCCGGTCTGGAACTCAACAACTGTGTGGTGCGAAGGGGACGACTGGTTACCAAACCGCGCTCGTCTAACC
CAAGTACCGCCCGGCATCCCCGAGTCTGTCATCACGGTGCATCTCAATAATAACAACATAACCAAACTGTACAACGGC
TCGTTTAGTGAATTAACAAACCTGAAATCTTTAGACCTGTCTTATAATGATCTACATACCATCGAGGTCGGTGCGTTC
GCTGGGTTGGAGAATTTAGAGCGTCTTGATCTAAGTGGAGTGGAAGTGGGATGGGGATCCAAACTCAAGTATTCCCTT
CAAAATGACCTTTTCCGAGATCTTCAAAACCTGAACTATCTGGCCGTGACGACTTCCACCGAGACAGTCAGTGCGGGA
GTGTTCACGGGGTTGTCTAAACTGAGACATTTAGAACTCACAGTGAAAAATGTCAGCCTTTTACCCGATCATATGTTT
GATTCTTTGACTTCATTGGAAAGTCTGAAGATAAAGGAAGAGATCTTTACTATCAATAGTAGTGTCCACAGCGGGAGT
GGGTTCTTACAACGNCTACGATTCCTTGATCTTAGTTACAATCAAATACAAACCATAGCAGAGAAAATGTTAGAAGGT
TTGGGCAACCTCACACACTTAAACTTGGCATCAAACAGAATAGCGGTGATAGGGGACGCCTTTCTCCGTCTCTACGGG
TTAAAGGATTTGAATATGAGGGGAAATAGTTTAGCTGTTTTAAACCAAACAATACTGAGTCCTGTTGTTAACCGACTA
GAGAGGCTGGACGTTGCAGGGAACCCGTTTTTGTGTGACTGTAACTTGATGTGGTTTGTTGAGTGGGCCAACGACAAA
TATGACAGGGTATCGAACTGGCAAAACCCTTACTATGGCGTCGGTCTAGGCTACACATGTTCCCGTCCAGCACAACTG
CGCGGACGACTTTTGATAGAAGGGTTGACGCAGAAAAAACGATCCAAAGACAGAAAGGACCCAACAGAAAGCAGATTC
TTCGGCACGGACTGTAACCAGGGATTTAGACCAAACCGCCTCCTGGCTTGTGTGCTCGCATCTTCGGGCATCTTCGTC
GCCATGATGACCATTTTCCTGGTCGACTACCACATCGCCCGTGTTCAGTACTACCTGTGGCAGTTGGCCAAGTGGAGG
AGACCGAAGATTGGTGAAGTAGAGAACAATGAGCCGCTCAGATACACGCACGATGCTTTTCTTGCCTACAACAACCGG
GACGTCATGTGGGTTGTACATGAAGCAATAGAGAATCTGGAACCTGACTACAGCCTGGTCATACATGAAAGGGACTTT
GCCGTCGGTGCTCCCATTGTGGAAAACATCGCGGATGCCGTGGAAAACAGTCGGAGAACCGTCTGCCTCATCACCAGG
AACTTCCTGAAGAGTAAGTGGTGCGAGTACGAGTTCCAGATGGCCCAGTACCACATGTTTGAGAAGGGGGGAGGGAGG
CGTCTCATCCTGGTGTTTCTGGAGAGGATTCCTGACAGACTGCTGAAACGGTTCCGCCATCTGAACGCCGTAATGAAG
AGGGACACGTACCTGGTGTGGCCAGGCGACGTGAGGAAACGACCGCTGTTCTGGGAGCGGCTGCGACACGCTCTGGGC
GATCCTCTACCCCGGGACCCAGAGCCTCAGCAGCAGGTACAAGATCAGGAACGGAACATCCCGGAACAGGCTCCGGTA
AGGAACATTGTGGAAGTGCAGATACATGGCCCCATGCGGAATATTCCGGAACAGCAAGATGACATTCTCTTACCAGAG
CCTGACGACCAGTGGTTTGGAGATGGTGAAGATGGTCCTCTCCTACCACTGTAG
>Bl08928b
ATGGAGTCTAAACCCGCTCTTCTCGCAGTCTGCTTAGCTGTGGTTTCCGCGTTCATCACCCAACATGGACGGGTTAGA
GGAGAATACAGACCACCTGTGTGTCAGACCTGGAACTCTACAACTGTGGCGTGTGCAGGGGACAATTCATGGTTAACA
ACTAGCGCGCTTCTATCCCAAGTACCGCCCGGCATCCCCGAGTCTGTCACCACCTTAATTCTAGATCATAACAACATC
ACTGAATTGTACAACGGCTCGTTTAGTGGGTTGAGAAACCTGAGATCCTTAGACCTGTCTTATAATTATCTACAGACC
ATCGAGGTCGGTACGTTCGCTGAGTTGGAGAATTTAGAACGTCTTGATCTAAGTGGAGTGGCGGGGGGATGGGGGCTG
AACCTCAATTATTCTCTTCAAAATGACCTTTTTCGAGATCTTCAAACCCTTAACTATCTGGCCGTGACGACTTCCACG
GAGACAATCAGTGCGGGAGTGTTCACGGGGTTATCTAAACTCAGGCATTTAGATCTCGCAGTGAAAAATGTCAGCCTT

TTACCCGATCACATTTTTGATTCTTTGACTTCAGTGGAAAGTCTGAAGATAGAGGATTCTTCCACTTCATCTTATAGT
CTATCTGAGGAAAAGGTAGGAGAGGACGAAGATAATATCACTAGAAGGGTTCACAACGGACGTGGGTTCTTAGAACGA
CCCCTGCTGTGGGCACCGCTGTACAACCTTAACAAATTGAGTCTCGGTGGTCAACTTAGAGCCAGTGACCTCTACTTT
GGAACTGTATTTAGAAACATGACTAAGCTAGAGACTATAGAAATTACTATAGACAGGACTGAAATATTCGATCCGGAT
CCCCGCCCCCTCAGTGTTGAAATGTTCCGGCCGCTGTTACTGACTCTGAAACATCTAGTCACTGAGGAACCTATAGCC
CCCGGCCTGCTGAAGTCACTCACACATCTGCAGACGTTGAACGTCAACTACCTCACCAATGTGGACGTTCTACCTGAA
CTCAACCACACGGAGATTCAGGAGCTGTCATTAGATATGTGGAGAGTGTACAAAATAACATCGGATACTCTATCGGGA
ATAAAAGGTCTTAAATATCTAAAAGATCTGTCTGGCGGATTCCTTGCATATATTAGAACCATAGAAGCTAACGCCTTT
GTGGGTTTGTCACATTTACAAAGGCTGGATTTGACGGCGGTCTCCTTGAAGAATTTACACAACAAGACGTTCAGCGGA
CTGTGCTCACTCACGCATCTTAACTTAAAACGAAACGCAATTTCTACTTTACCTGTGGACGTGTTTGAAGGACTGACC
TCTCTTAGATATCTGCATTTGAAAAGTAACAACTTGGAAACCTTTCGAGTTCAGCTTCCAGAGACTTTGGACTATCTT
GATCTCAGTTATAACAACTTATACACTAGCGGATACACAAGTCAACCATGTGTCAACATTTCATCATTTACCTTCAGT
AGGTTGAAAAGCGTGAATTATCTGGATTTGAGTCATAACACAATTGGTCTTATTGACTGCAGTTGTCTACCGTCGAAT
ATTACTGTGCTTAATTTGCAGTCCAACAAAATAACAGAGGTCAATACAAACGTGTACTGCATTGCAAGTTTACGGTAT
CTCGACCTTTCGAATAATAACATCAACGTTTGGAGTGACGAACAGCAATGTGAGCAACCTAATTTAGAAATACTACGA
CTGGATAACAATCCTTCAGGGTTTATTGCAGAAGATTTCAATATTGGTTCGGCATCAATATCGCTGAAATACTTGAAA
ACGTTGACTCTTTCTCGCTGCAGCATTCCGAGTATCTCTCCAAAAGGATTTAGAGGATTGTACCACTTGACATATCTG
GATCTCAGTCATAACGAGATCGACACTGTGAAACAATCCGTCTTTCGTGAACTATCGCGGCTACGATTCCTGGATCTG
AGTAACAACCGAATACAAAACATGGCAGAGAGAACATTTGAAGGTTTGGGCAACCTGACATACTTAAATCTGGCGTTC
AACAATATAGCGGTGATTGGGAACGCCTTTCTGCGTCTGTACGGATTAAATGATTTGAATATGAGGGGAAATAGCTTA
ACTGTTTTAAATCAAACTGTACTGAGTCGTGTTGTTAATCGGCTGAGGGGGCTGGACGTTGCAGGTAACCCGTTTCTG
TGCGATTGTAATTTGATGTGGTTTGTTGAGTGGGCCACCGATAAATACGACAGGGTATCGAACTGGCAAAACCCTTAC
TATGGCGTTGGTCAAGGCTACACATGTTCTCGTCCAGCAAGACTGAATGGACGACGTTTGACAGACGGGTTAACGTTG
ACGCAGAATAAACGATCCAATGACAGAAAGGACCCAACAGAAAGCATGTTCTTCGGCAAGGATTGTAGCCAAGGATTC
CGGCCCAACCGCCTCCTGGCTTGTGTGCTCGCATCTTCGGGCATCTTCGTCGCCATGATGACCATTTTCCTGGTCAAC
TACCACATCGCCCGTGTTCAGTACTACCTGTGGCAGTTGGCCAAGTGGAGGAGACCGAAAATTGGCGAAGTAGAGAAC
CATGAGCCGCTCAGATACACGCACGATGCCTTTATTGCCTACAACAACCGGGACGTCATGTGGGTTGTACATGAAGCA
ATAGAGAATCTGGAACCTGACTACAGCCTGGTCATACACGAAAGGGACTTTGCCGTCGGCGCTCCCATTGTGGAAAAC
ATCGCGGATGCCGTGGAAAACAGCCGGAGAACCGTCTGCCTCATCACCAGGAACTTCCTGAAGAGTCAGTGGTGCGAG
TACGAGTTCCAGATGGCCCAGTACCACATGTTTGAGAAGGGGGGAGGGAGGCGTCTCATCCTGGTGTTTCTGGAGAGG
ATTCCTGACAGACTGCTGAAACGGTTCCGCCATCTGAACGCCGTAATGAAGAGAGACACGTACCTGGTGTGGCCGGGC
GACGTGAAGAAACGGCCGCTGTTCTGGGAACGGCTGCGACACGCTCTGGGCGATCCTCTACCCCGGGACCCAGAGCCT
CAGCAGCAGGTACAAGATCCGGAACGGAATATTCCGGAACAACAGGCACAGGCCCCGGAACAGAACATTCCGGTACAG
CAAGAACAGGCTCCGGTGAGGAACATTGTGGAAGTGCAAGTACATGGCCCCATGCGGAACATTCCGGAACAGCAAGAT
GAAATTCTATTGCCAGACGACCCGTGGTTTGGAGATGGGGATGACGTGCCTCTCCTACCACTGTAG
>Bl08928c
ATGAAGTCTAAGCCCGCTCTTCTCGCAGTCTGCATAGCTGTGGTTTCCGCGTTCATCACCCAACATGGACGGGTTAGA
GGACAACTACCCCCACCTGTGTGTCAGACCTGGAACTCTAAAACTGTGGAGTGTGCAGGAAAGACGTATGATAGTCCA
ACGGGTTTGCAACTAATGAAGGTACCTCTAACCCAAGTACCGTCCGGCATCCCCGAGTCCGTCATCACAGTGGATCTC
AGAAATAACAACATAACTAGACTGTACAACGGCTCGTTTAGTGGGTTAAGAAACCTGAAATCCTTAAACCTGTATCAT
AACAATCTACAGAACATCGAGGTCGGTACGTTCGCTGGGCTGGAGAATCTAGAACGTCTTGATCTATATGGATTGATA
GAGAGACGGGGACTCAAAATCACGTATTCTCTTCAAAATGGACTTTTTCTTGATCTTGAAAACCTGAACTATCTACGC
GTGAAGACTTTCACTGAGAAAATCAGTGAGGGAGTGTTCACGGGGTTATCTAAACTGAGGCATTTAGATCTCACAGTG
AAATATGTCAACCTTTTACCCGATCACATTTTTGATTCTTTGACTTCATTGGAAAGTCTGAAGATAAAGGAAAACGTC
TTTGCTATCGCTGGAAGAGTCCACAGCGGAAGTAGGTTCTTACAACGACCCCTGTTGTGGGCACCGCTGTACAACCTT
AACAAGTTGAGTCTGTATTGTCTAGTAAGAGCAATTGATCTCAACTTTGGTCCTGTATTTAGAAACATGACTAGGTTA
GAAACTATAGAAATTACTATAGTAAGAACTGCAGACTTCCATCCGGCTCTCCGCCCCCTCAGTGTTGAAATGTTCCAG
CCACTGCTATTAACTCTAAAACATCTTCTCACTAACGCCCCTATAGCCCCCGGCCTGCTGAAGTCACTCACACATCTA
CAGACGTTGAGCGTCATTTCATACCGTTACCCCACCACTGTGCACGTTCTGCGTGAACTCATCCACACGGAGATTCAG
GAGTTGTCAATTGACATTTCGAGAGATAGAACGTACAAAATAACATCTCATACTCTAGCAGGAATAAAAAGTCTGAAA
TATCTAAAACATCTGTTTTTTAAAAAACTAGACGTTACAAATATCAGAACCATAGAAGCTAATGCCTTTGCGGGTTTG
TCACATTTACAAAGACTGAATTTGATGAGGGGCTCCTTAAAGACTTTACAAGATAAGACGTTCAGCGGACTGTCCTCA
CTCACTCATCTTAACCTGGCAGGTAACAGAATTTCTACTTTACCAGCGGGCGTGTTTGAAGGACTGGCCTCTCTTAGA
CATCTCGATTTGAGAGGTAACAACTTGCAAACCTTTCGAGTTCAGCTTCCAGAGACTTTGGACTATCTCGGTCTCGCT
GGTAACAACTTAGACACTAGAAGACACATAGGTCTGACTCAACCATCATGTGAGAATTCACCATTTAACCTCCGAGGT
TTAAAAAGCGTGAATCATCTGGATTTGAGGTATAACGCAAATAAAGCTATTAACGGCCAGTGCCTACCGTTGGATATT
ACTGTGCTTAATTTGCAGTACAACTATATATCACAGTTCAATACAGACGTGTACTGTATTGCGAGTTTACGGCATCTC
GACCTTTCGAACAATCATATCACCATGGTCTTAGGTTTCAAACAGCAATGTCTGCCTACTTTAGAAGTATTACGACTT
GGTAACAATCCTGCAAGTCTAATTCATCTATACAATATTGATAAGTCGCCATCACTGAAAAATCTGAAAGCAATGAAC
CTTTCTCACAGCAGCATCCAAAGTATCTCTGCGGAACAATTCACAGGATTGTACCACTTGGCATATCTGGATCTCAGT
CATAACATTATTAACACTGTGAAACAATCCGTATTTCGTGAACTATCGCGGTTAAGATCCCTGGATCTTAGTAACAAC
CAAATACAAAACATAGCAGAGAATATGTTCGAAGGTTNATAGTTGAGAAAACGTTCGAAGGTTTGGGCAACCTCACAC
ACTTAAACCTGGCGTTCAACAGAATAGCAGCGGTTGGGAACGCCTTTCACCGTCTGTTCGGGTTGAGGAACCTGAATC
TGAGGAGCAACAAGTTAGCTGTGTTAAATCAAACAACATTCGGTCCTGTTTTCAGCCGGTTAGAGACTATCGACATTG
CAGACAACCCGTTTCTGTGTGACTGTAGGTTAATTTGGTTCGTTAAGTGGGCAAACGACAAATACGACATGGTAGCGA
ACTGGTACAACCCTTACCCTGTCGTTGGTCGAGGCTACACATGTTCCATTCCAGTACATTTGCGCGGACAACGTTTGA
TAGACGGGTTGACGCAGAATAAACGATCCAACGACAGAAATGACCCAACAGAAAGCACGTTCTTTGACATGGACTGTA
GTCACGGATTCCGGCCCAACCGCCTCCTGGCTTCTGTGTTCGCCTCTTCGGGCATCTTCGTCGCCATGATGACCATTT

TCCTGGTCAACTACCACATCGCCCGTGTTCAGTACTACCTGTGGCAGTTGGCCAAGTGGAGGAGACCGAAGATTGGTG
AAGTAGATAACCAAGAGCCGCTCAGATACACGCACGATGCTTTTATGGCCTACAACAACCGGGACGTCATGTGGGTTG
TACATGAAGCTATAGAGAATCTGGAACCTGACTACAGCCTGGTCATACACGAAAGGGACTTTGCCGTCGGTGCTCCCA
TTGTGGAAAACATCGCGGATGCAGTGGAAAACAGCCGGAGAACCGTCTGCCTCATCACCAGGAACTTTCTGAAGAGTC
AGTGGTGCGAGTACGAGTTCCAGATGGCCCAGGCCACATGTTTGAGAAGGGGGGAGGGAGGCGTCTCATCCTGGTGTT
TCTGGAGAGGATTCCTGACAGAATGCTGA
>BL75614
ATGGAGACCAAAATCACTCTCCTTGCGGTCTGCTTAACCACGATATTGGCGTTTATCACACAAGTGTGGGCAGGACAG
CTGCCCCCTGACTGTCAGGTCTGGAACTCCACAGCCGTGGTGTGTACAGGACAGGGCGGCCCATGGGAGTACCCAACC
CGCGCAACTCTAACCCAAGTACCGCCCGGCATTCCCGAGTCTGTCATCAGCTTGGACCTCAGCAGAAACAACATCACC
GAACTGTACAAGGACTCTTTCGCAGGGTTGACTACTCTAAGGTACTTAGCTCTGCGCGAAAACAATATAGCCAAAATA
CACAACGGCTCTTTTCGTGGATTAAGAAATCTGAGGCACTTGGATTTGTCGGACACACGTATTCAGGCAATCGAGGTC
GGCGCATTCGCTGGGCTAGAGAACTTAGAACGTCTGGACATGTGTTGTCCAACTTCGCAATCTACGTTTGCTCTTCAA
ACTGGGCTGTTCATAGATCTGCGAAACCTGAATTATCTGACAGTTGAGACCGCTACCGAGAGGGTAGATGTAGACGTG
TTCAAAGATTTATCTAGACTGAGGTATCTATATCTCGGTTTGATCAATATAAGAAGTTTACCTCATCACATTTTTGAT
TCCTTGACTTTGCTGAAAAACCTGACAATTGCAGAACTGGACATCCAAAGCGGAAGTGAATTCCTACAGCGACCCTTG
CTGTGGTCACCTCTCCACAAACTTAAAACATTTCAACTCTTCGTCCAAAGAGCCGACGATTTAAATTTCGGGTCTGTA
TTTGGGAACCTGTCGAACTTGGAAGAAATACAACTAGGCTCTGTAGGCCCAGTTAACTGCAATGTTCAAATGTTCCTT
CCGCTATTGTCAACTCTGAAACGGTTAGACCTGCGTCTTAGCGCTCACGTCTATACACCAGCTGACCATCTAACCATA
GAGCCCGGCCTGCTGACGTCACTCACACATCTACAGGCGCTGGACATTCCTTGGACAACTTTCTCCTCTATCGCAGAG
GTTCTGCCTGAACTTCGACACACACATGTACAGGAGCTGTCGTTTGCCATGGGCGGGATTAACACAATAACAGCTGAT
ACGCTGGCGATAATGAAAGGCTTGGACGAGCTGAAAACATTGTCTATAATTCTACCTGACACCCACAGTCATTCAAGCA
AACTCATTCACAACTTTTCCAAATTTACAAAGGTTGAATCTGGATGTAGGGATTCTAAACACGGTAGAAAACAAGGCG
TTCAGTGGTTTGCCCTCCCTCAGTCATCTTGACTTAAGCAATCATCTAATTTCTACTCTACCACAAGACGTGTTTGAA
GGACTGTCATCTCTTATCTGTCTTGATCTGAGCTATAACAACTTGGAAACATCCCAAGCTCGGCTTCCAGAGACCTTG
GACTATCTCGACTTGAGTCATAACAATTTAAACAGCTACTTTCAAATGGAATCAGGCTACATCGCCTTATCGTTCAAC
TTCACAGATTTGAAAAGCGTCAATCATCTGAACTTGAGCCATAACAGACTGCAAGGTGTTGAAATGTTGCCAGCGAAC
GTGACCATGCTCGACTTGCAGCATAACCAAATAGGATATTACTTGCCCTATTCTGTATTCTCCATTGCAGGTCTGAAA
TTTCTTGATCTTTCTCACAACGACATCAGAACTACGTCCTACACAACAGTGCAGTCATCAACGTTAGAAACACTCAGA
CTGGATAGCAATGGTTTAGTAGGGATAGATGGGACGTTTATGAAAGGACTGACGAACCTTACAATATTGACTCTTTCG
CACAACCACATCCGTGTCATAGAGGCAGGGAACTTCCAGTGGTTGGTCCAACTGCTTCAACTCGATCTCAGTCATAAC
GAAATTAGCACTTTGAAGTCGTTCGCCTTTCGCGGACTATCACGGCTAAGATCGCTGGATCTTAGTAACAATCAAATA
CAGAACATGACAGCAAAGACATTTGAAGGTTTGGGCGACCTAAAAATCTTAAACCTTGCAGACAACAGAATAGCAGTG
ATTTGGAACGCCTTTGATCGTTTATACGGACTGAGGGATTTGATTCTTAGCAACAACAGGTTGAGTGTCATAAACCAA
ACCTCGTTGGGACNCAACAGGTTGAGTGTTATAAACCAAACCTCGCTCGGACCTGTTGTCAACCGCCTGGAGAGACTT
GACGTTGCAAACAATCCATTTCTGTGTGATTGTGACTTACGGTGGTTTGTTGAGTGGGCACAGGACAAATACGACACG
GTGTCGAACTGGAATAACCCCTATCCTCTCTCTTATCGAAGCTACAAATGTTTCCGTCCAGCTAAACTGTATGGGCAA
CGTTTGACTGACGGGATCACACAGCAACAGAACTATAACGAAGGAAAAGCACAATCAGAGAACATGTTCTTTAAGATG
GATTGTAGTCATGAATTCCGGCCCAACCGTCTCCTTGCTTGTGTGCTCGCTTCTTCGGGAATCTTCGTCGCCATGATG
ACTGTTTTTCTTGTCGACTACAACATCGGGCATGTTCACCACTACCTGTGGCTGTGGGCCAAGTGGAGAAAACCGAGG
ATCGGAGAAGAGGAAATCAATGAACAGCACGCCTACACACACGATGCTTTTATTGCATACAACAATGAAGACATCCGA
TGGGTTATAAAGGAAGCAATAGAGAACCTGGAACCTGACTACAGTCTAGTCATACATGAGAGGGACTTTGCAGTCGGC
GCCCCCATCGTGGAGAACATCGCCGACGCCGTGGAAAACAGTCGGAGAACCGTCTGCCTCATCACCAGGAACTTCCTG
AAGAGTAAGTGGTGCGAGTACGAGTTCCAACTGGCCCAGTACCACATGTTTGAGCAAGGGGGAGGGGTGCGTCTCATC
CTGGTCTTTTTGGAAGAAATTCCGAACAGATTGTTGAAACGATTCCGCCACCTGAACGCTGTAATGAAGAGGGACACG
TACCTGATGTGGTCGGAGGACGTGAAACACAGGCCGCTGTTCTGGAAGCGGCTGCGACACGCTCTGGGTGAACCTTTG
CCTCGGGACCCAGAGCCAGAACAGATACCAATGACGGAACCTCATATTCCGGAACAGATCGATCAAACTGCCGTCGGA
AATATTCCGGAATGGCAGGACCATTCTCCGGGGCGAAAAGTTCCGGAACAGAAGGGACAAGCTCCAATAAAGAACTGT
CCTGAACTCCAGACGCAAGCTCCGACAAGGAACACTCCGGAATATCAGCTATTGCATCCGCTCATGCATATTCCGGAA
CAACAAAAAACGACAACAACTCCGGAATGTGCCATACACGGTCCGGAAGATAGAATTGTAGAATCAGAGTCAGAAGAT
GATTGGTGTGGACCTAGAGATGCTGACATCATTCTTCTGCCACTGTGA
>Bl30396
ATGGCGTCTAAACCCGTTCTTCTCGCAGTCTGCTTAGCTGCGGTTTCCGCGTTCATCGCCCAACATGGACGGGTTAGA
GGACTCCCCCCACCTGTGTGTCAGACCTGGAACTCAACAACTGTGGTGTGCACACGTGGAATATTTGATTTTCCACTC
CGGGCACCTCTTACCCAAGTACCGCCCGGCATCCCCGAGTCTGTTATCACTGTGGATCTCAATAATAACAACATAACC
GAACTGAACAACGGCTCGTTTTGTGGGTTAAGAAACCTGAAATCCTTAGACCTGTCTTATAATGATCTACAGACCATC
GAGGTCGGTACGTTCGCTGGGTTAAGAAACCTGAGATCTTTAGACCTGTCTTATAACAATCTACAGAAAATCGAGGTC
GGTACGTTCGCTGGGTTGGAGAATCTAGAATGTCTTGATATGTGCGTGCCAAGACGAATGCAAGAACCAATGTATGCT
CTTCGAAATGGACTTTTCCAAGATCTGCGAAACCTGAACCATCTGCGGGTGATGACTTACACCAATACAGTCAGTGAG
GGCGTGTTAACGGGGTTATCTAAACTAAAACATTTAGAACTTAATGTGAAAAATGTCAGCAATTTTCCCGATCACATT
TTCGATTCTCTAACTGCATTGGAAAGTCTGAAGATAGTGGACCTCTTAATGGATCTCGCGATCCACGGTGCCATAGAA
GAGGACAGAAATGCCACCACTAGAAGAGTCCACGGCCAACGAGGGTTCTTACAACGACGCCTGCTGTGGGCACCCCCTG
TACAACCTTAAAGAATTGAATCTCTTTTTTCTACCAAGATCCATTGACTTCTACTTTGGACCTGTATTCAGAAACCTA
TCTAACTTAAAGACTGTAGAAATAATTTCACTTCACAACTACCCCGTCAGTGTTCAAATGTTCCGGCCGCTGTTACTG
ACGATAAAACATTTACACATACACACCCCGATAGCCCCCGGCCTGCTGAAGTTGCTCACACATCTGCAGACGTTGGAC
TTTGAACATGTCTTCTTTCCCACCATTGTGAACGTTTTGACTGAACTCCGACACATTCATATTAAAGAGCTATCGTTT
AGCATGTTGGGATACGGAGGGCCCATAACAACAAGTTTTACTCAAGCCAGATTAGGGACCGTAATAGCAAATCATACT
CTGGCCGGACTAAAAGGTCTGAAATATTTAAAAGGTCTGATTCTGGATTTACAGAATTCAATAATTCAAGTCGTACAA

GCTAACGCCTTTGCGGGTTTTTCACATGTACAAAGGCTTGATCTGACTAATGGTCTCTACCTAAAGACTTTACAAGAC
AAGACGTTCAGTGAACTGTCCTCACTCACTCATCTTAACTTGACACAAAACACAATTGCTACTTTACCAGCGGACGTA
TTTGAAGGATTGACCTCGCTCACTCATCTTGATTTGAGCCATAACAACTTGGTAACCTCCCTAGCTCAGCTTCCAAGG
ACATTGGACTATCTTGATCTCAGTTACAATAACTTGTACAGCAAACCATATCAACCTCCATGTCACAACCCTTTATCA
TTTAACTTCGGTGATTTAAAAACCGTAAATCATCTGGATTTGAGTCATAATAATATCATTGTCGTTGACAACACTTGT
CTACCTCGGAATATTACTGTGCTTGATTTGCAGCACAACAAGATAGAATCGGCCGATGCTACATACTGCATGGAAAGT
TTACGGTATCTCGACCTGTCTAACAATGATATCATGACGTTCAGCCCTAATGTTTATTATTCAAGATGTGCGGGATTT
ACCTTACTAGAAACACTTAGGCTGAGTAACAATGCTATAGAATATATACATCTACCGGGATGGTTTCCAAACCTCCAA
ACGTTGACTATTTCTCTCAACCACATCAAGCGTATCGAAACAGACCAATTCCCAGGTTTGGACCGGTTGGAACATCTG
GATCTCAGCCATAACGACATCAACACCATCATGCCATCCGCCTTCCGTGGACTATCGCGGCTAAGATTCCTGGATCTC
AGTGACAACAAGATACAGTTTATAACAGAGATGACATTTGAAGGTTTGGGCAACCTTAAACATCTAAACCTGGCAGCC
AACAAGATAGCGGTGATAGGGAACGCTTCTCTTCATCTGTACGGGTTGAGGAACTTGAACCTGAGGAGCAACAGGTTC
GCTGTGTTAAATCAAACAACATTCGGTCCTGTTGTCAACCGGTTAGATACTATCGACATTGCTGACAACCCGTTTAGG
TGCGACTGTAACTTGATGTGGTTTGTTGAGTGGGCCCAGGACAAATACGACAGAATAACAAACTGGCGAAACCCTTAT
CCTGGCGTTGGTCGAGGCTACACATGTTCTCGTCCAGCACAACTGCGCGGACGACGTTTGATAGACGGGTTGACGCAG
AAGCAACACTACAACGGCAGAAAGGACCCAACAGAAAGCACGTTCTTCGACATTGACTGTAGNAGCATGTTCTTCGAC
ATTGACTGTAGTCAAGGATTCCGGCCCAACCGCCTCCTAGCTTGTGTGCTCGCCTCTTCGGGCATCTTCGTCGCCATG
ATGACCATTTTCCTGGTCAACTACCACATCGCCCGTGTTCAGTACTACCTGTGGCAGTTGGCTAAGAGGAGGAGACTG
AAGATTGGCGAAGAAGAGAACCAAAAGCCGCACAAATACACGCACGATGCTTTTCTTGCCTACAACAACAAGGACGTC
ATGTGGGTTATACATGAAGCTATAGAGAATCTGGAACCTGACTACAGTCTGGTCATACACGAAAGAGACTTTGCAGTC
GGCGCTCCCATTGTGGAAAACATCGCGGATGCCGTGGAAAACAGCCGGAGAACCGTCTGCCTCATTACCAGGAACTTC
CTGAAGAGTCAGTGGTGCGAGTACGAGTTCCAGATGGCCCAGTACCACATGTTTGAGAAGGGTGGAAGGAGGCGTCTC
ATCCTGGTGTTTCTGGAGATGATTCCTGACAAAATGCTGAAACGGTTCCGCCATCTAAACGCCGTAATGAACAGAGAC
ACGTACCTGGTGTGGCCAGGCGACGTGCGGGAACGGCCGCTGTTCTGGGAGCGGCTGCGACACGCTCTGGGCGATCCT
CTACCCCGGGACCCAGAGCCTCAGCAGCAGGTACAAGATCAGGAACGGAACATTCCGGAACAACAAGAACAGGCTCCG
GTAAGGAACATTCCGGAAGAGCAAGAACAGGCTCCGTTAAGGAACATTGTGGCAGTTCAGGTACATGGCCCCATGCGG
AACATTCCGGAACAGCAAGAACAGGCTCCGGTAAGGAACATTGAAGAAGTGCAGGTGCATGCCCCCATGCGGAACATT
CCGGAACAGCAAGATGAAATTCTAGAGCCAGACGACCCGTGGTTTGGAGATGGGGATGACGTTCCTCTCCTATCCCTG
TAG

## Protein sequences

>BlTLR22

MENPPKSTSTCIYSLLCLCLFLLSVKGDHVANPYQCQEWTTLHITCTKLQLSKVPDNIPPSTLHLDLHDNSITELQQE
DFKTLINLQYLDLRWNKIDHIENATFAPLANLKTLNVSGNKIHVSLLPQLVDFLPSLEHLEISVNWKWDDPVMLGNMT
SFKGLGNLTSLNLGGNDIVDVQENSFDGLDKLQSLNLRDNLISNINEASFSPLKELEHLVLSNNYLTDDVLQVDKLWS
PVVKLTSLYLSENLLSFARFPSVFQNFSLLHTLDLSRNQLINLTTDDFASLLFTPLQILQLERNSISHIDQGLLASLA
NLKSLKLQSNPILFSQLKDKLVGLQIEELTLGGSPDLDIIRSDTFPSLPSLKHLTMSLLYDWNPSIKSSRLMGGSFLN
LPNLTQLNLEDYSISSVEPYTFTGLEYLERLELGENNIADFPTHAFDGLSSLTHLDLGHNSLTAVKSHYFHSLKNLVW
LNLQNNDIYLIEETAFKDLESLQFLILTSNHLTTVAGLQLGLSNLRHLDLERNNFTSIKTGSFSRLESLTHLTLAHNW
IRKIEKEAFSELARLKRLNLADNRLTNLTSWAFDGLSELEEIKLQHNLIVVVESHAFYGLEQMTKLNLKGFSIATIPD
NAFMGLHNLTVLDLSLNQIKTFGKKAFNGLDNLRVLQLQKNEITFLDETVFKEVLDRVWKMDIQDNPLFCDCDLLWLV
FKANSQPKKVVGWNTSSFKCAAPPKDQGKSLQILPSQCEYVFMPNLWLASLLSSLGIFLFVMTTFCVNYYTWKLRDLW
FRIRHRDRVVQVCDNDHRFVFDAFIAHHNEDRRWVERDLCRNLECSENCPNYRLCLHQRDFQAGVPIITNIRTAVDSS
RKIVCVITRSFLRSRWCQFEFQLAQHTMVEEGGGIRLILVFLEDIPRHLVRQYRHLQAVVDRDTYLEWPGDPRERPLF
WRRLRAALGQPLDQQPDDKDSEPDQHGFMALVEV

>Bl10262

MPTPPPMKELRSLKTISMKNNAIQTLNLTSVKDLAEVTSIKFTGNALERIPFLAFAKSTLAKLEDLMLDSNKISVIHE
DAFGGLPSLKVLDLNGNMIQVLNKAILHQLEKVEKLNLSNNAIHTLEAETFAEMKDLHVLDLTQNNMTFTSETSASLF
TGPENLTSLYLAQNLIKSCPEDLGKALPYLRHLGLQKNNISVFSETFLKGFGSLKEIDLSGNPFSCTCDVEWLFDRLK
ENGTVAFIKWEEYECYYPRHSRGVNFEHFNPEVLKCDHTDHTRLGLSLGISLTLAVLVIVLAAVLYYHCRWRVKYGWF
VLCGKKGEQVEQKVEDEYFKYEAFLSYCSNDRWWVIDELLPKVENCPPPTYKMCLDIRDFEDGPKDQNNIIAAMDESR
KTMFVISSSFLRSKRCLWELEMSRNKRYGKSRDDLI

>Bl09440

MAAGRLWLFVATTLVLWSVSQLQQTPLAGLGHIGARIKACRVYNRTVADCSSRGLRSVPDARELPGTIKTLYLLSNNF
RALPHCAFCDLPLLEFVDLSFCNISDINRTAFAKLARLKTILLIGNNLTSLESGTFDEQHSVTEVNLADNKLTEVPYG
AFPLNNSLKTLDLSGNNINIQEEDTDWDRFKSLQHFSFRLNNVSSSAGWIFSREDLNVSLQTLDLSYNRISQLDSTAF
NGIRRLDRLVLSSNDILTVDTDSFSSLSQTGLETLVLFRNNLTTLTNNTFSSVPLLKRLDLAYNNISSIEDNAFAGLR
YLEVLTLYMNPLWTNVPMTALEKLSPSLLELDLRFCSILNIYPEQFRSFGNLRSLNISDNYIGALGSGHRLTGKEFDG
LGNLRVLDIGGGPGYNRMKITNESFSHLPKLQKVVMTGLLGFNERFDGSFRKNPDLRLINMGNMNIRFLGVDLFQGLT
KLTELVLSNNNFYLMLNSDQGSANFARFFGDLTNLEILRLQGNRLESLQSDIFQNLTHLRYLNLGPGRYGDHNNQLSN
LQPELFQSLTSLETLRIDGNRLSSVEQSVFEPMFKSLKELFIYGNPFDCTCENLRWFRDWVNNTRADVIGLGEGRLVC
STPRRYANESILSFRPEVDCQSQVGVIVSLTVCSVLLAAALGTVVCRRFGLHKYVQYVWVLVTRRKEGYEKLQGEDLE
VEYDAYVSHTEEDLRWIQRVLIPNLEEKSPQLKLCIPDRDTPPGEAIIDNVQDYIRRSRKTLCLVTQRYLAQEASWLE

MQVASYRLFDQEDRRDVVVMVFLEPIPKNKLTHFQNLRKLMRPGMFLHWPGEEDTAAHPLFWLLLRDALGTSNNPVPR
SRPQVI

>Bl22164

MFLPLLLTLKHLFTDAPIAPGLLKSLTHLQTLGPRYGYSPDILDVLPELRHTQIQELSFSMLFGMLWRSRIEKITPDT
LSLVIKGLKYLKTLFLDITDIKTLKVNAFAGLSHLQRLGLTGGSLKTLHDKTFSGLSSLTHLNLTQNKIPGLTEGVFQ
ALTSLTHLNLSYNKISTLPAGVFEGLTSLSHLDLSHNHLVASLAQLPETLDYLDLSFNQFDSRDTGQTPCYSTIFSII
FNFNGLKQMNYLNLSHNAITYVDGLCLPRNIAVLDLQHNQIAYVPRIPSARYLDLSNNYVKYFVGSVLPPGQSAVETL
RLDNNAIVLNDMGGPDLEDWLSQKLLVKLKTLIVSHNRIQRMATDQFPGLGQLKHLDLSHNEINTIMPSAFRGLSRLA
ILDISDNEIQFITEMTCEGLSNLTDLNLAANRIAVIGNAFRRLYGLRNLNLRSNRLAVLNQTTFGPVVSWLETIDIGD
NPFLCDCKLMWFVEWANDKYDRVSDWRNPYPNFGRGYTCSRPAELHGRHLIDGLTQKQQFNGRRNPAERKFFDAVCSH
GFRPNRLLACVLASSGIFVAMMTIFLVDYHIARVQFYLWQLAKWRRPKVGEVENQEPHRYTHDAFLAYNNRDVMWVVH
EAIENLEPDYSLVIHERDFAVGAPIVENIADAVENSRRTVCLITRNFLKSQWCEYEFQMAQYHMFEKGGGRRLILVFL
ERIPMASFPCQNNDVEKARYTASIQRFIVCPTLDVQAKRPCHTCHTYVV

>Bl05337

MERKILISVISAIFICGVSMSPDISCDILGGTLDSTIYTYNCTYTLCPDNPFRYCLYRDYCRSTTLDSFFNRQKFSTL
VTCCADPTWAEDTLQNRSIAVEELYMWHCHDSNSTVSFLRSIRGLKKLSFHIHYSVDKITTNFFPDMSDLVVLDLTAF
GPLTLETNAFRGLSNVQNATIMCKEDIVVKPRTFDGMVSLQSLTMISENWILHLPEQPFTGLLSIKTLTLQTGDCSPP
PNVTQVTFYDIARTIQNINLTGISVTGPAFAGCTNLSSVEWSNKWSEYSCNSRSGPDISPQAFQGAPSLESISLHSIS
LQSIRGRMFEGLHDLVNLSIEFADFSGIKYLPSDMFVGLSSVRHIWLDGSPELPGNIFQGLTSLREIRIENMVAHAMV
NSSGKFCYTDFKHLFSGLESLEIVRLIDSPIXXLQSLEIVRLIDSPIECETLPTLTFSNLPSLKEVHLIGTRPLKFLP
IGFFHDVPVLHTFKYTERTPRSTAQVTLPPLFFAGLNIEAIDLSRQSFLNISRYLFQSLTSLQSLYMAGCLFDEESVS
AVETLFSGLTSLEHLDVSFESIEYRSYTNFPLLNNMFKDLVSLQTLRMTGWTSSLQRRPIPSMLFKGLFSLTVLQLPD
LRSSSARESVQLPQNLFDDLVNLEYLNFEYVPVQSLDKDTFKKLTNLRQLYLNNTLLTQKGLSTLPFSHLKSLEILSL
RSVPLDELSQDLFVGADNLSMINLIGCPFPTIKSGTFRHLKNLTSLEIPLPRTIEKGAFDFKNQRSVHIYLVPVDLSF
YTSSFPNDFSQLFSNQTLMKKSSIQIDFVGEYICDCKMATLSVSYRRNQTLTRRIMPRVDGLSCIWKNDGASVDYNPS
NNIALRCPVSGPSCPERCYCRTSTIVAEAFCSDRGLTEVPDRFPVETSLVRLDLNSIQYVQPLAFESAPNLLILNLST
NAIRELNGSAFLGLRRLEVLYLDGNELSHVPMDLLQSLVNLRALWLNNNNLLDLPRTLFHEQTIFLRVLRLDHNNLTT
LSTGIFSNRTSLTKLSLVNNKFDCDCHLLWLKTWMLQHRRVIDEIKSVTCRSRGNAKDKYILKPIIELPDDAFICGDE
STYNPLVWVAAPGGLTIFLLLILGVFKCRKNVRVWVYARYRRGLRHLEQEPEKTYDIYVSYCVEDEEFVDREVVRVLE
DMDPPYKVCLRNRDFIPGHNNIQQAADSITSSRRTLLVLTERFLQDRWCLWEFQVAHQQAVTDQAYRLIIVIMDDLPL
EACDDVMDLKQYLTANKYLLWGELLFWDKLRQAVPPPGVRGEVDGVEEEGEENDDSDESDYEAHVNIFVPR

>Bl07821

MSDLVVLDLKATDWPLTLESHAFRGLSRVQNVTITSWEDIFVKPCAFDGMVSLQSLTMISEYQIFHLPEQPFAGLLSL
KTLTLQAGDCSQQPSNVSQVTFHDIAHTIQNISLTGISVTGPAFAGCTNLSSVQWSNGCSSSYSSQPGPNISPQAFQG
APSLESISLHSISLQNIEGRMFEGLYNVVNLSIEFADFSGIQYLPSDMFVGLSSVRYIWLDGSPELPGNIFQGLMSLR
EIRIENMVARAMVNGSGKFCYTDFQRLFSGLQSLEIVRLIDSPIECETLPTLTFSNLPSLKEVHLIGTRPLKSLPIGF
FHDVPVLQKFKFTEWSLRSTAQVTLPPLFFAGLNIEVIDLRRKSFLDISRYLFQNLTSLQSLYIVDCEFSEESASAVE
TLFSGLTSLKHLDLSDTKMPLLNNMFKDLVALKTLRMTDWTSSLQRRPVPSMIFKGLRSLKVLYLEDSRSFHTSESVQ
LPQDLFVDLVNLDYLNLENVPVQSLDKDIFKKLTNLRQLYLNNTLLTQEGLSTLPFSHLKSLEVLSLRSVPLDELSPD
LFIGANNLSIIDLIRCPFPTIKSGTFRHLTNLTSLEIPRPQTIEKGAFDFENQRGVHIDLFRPVDPLFSLSSLPEDFV
ELFSNQNLMEKSSIQIVDFNDDYNCDCKMATLSVSYRRNQTLTRRIMPRVDGLSCILENDGASIVNNPSNNALRCPIS
GPSCPERCYCRTSTIVAEVFCSDRGFTEVPDRFPVDTSLVRLDLNSIQYVQPLAFESAPNLLILNLSTNAIREINGSA
FLGLRRLAELYLDGNELSHVPMDLLQSLVNLRVLWLNNNNLLDLPSTLFHEQTIFLRVLRLDHNNLTTLSTGIFSNQT
SLTKLSLVNNKFDCDCHLLWLKSWMLQRRRVIDQIKSVTCRSRGNAKDKYILKPIIDLPDDAFICGDESTYNPLVWVA
APGGLAILLLLILGVFKCRKNVRVWVYARYRRGLRHLDQEPEKTYDIYVSYCVEDEEFVDREVVRVLEDMDPPYKVCL
RNRDFIPGHNNIQQAADSITSSRRTLLVLTERFLQDRWCLWEFQVAHQQAVTDQANRLIIVIMDDLPLEACDDVMDLK
QYLTANKYLLWGELLFWDKLRQAVPPPGVRGEVDGEEEEEGGGNNDSDESDCEDHVDIFLPR

>Bl52875d

MLFLQKNRLSCLPHDLFAQLSTLENLDLSYNSLKNILQADIWKGLNLQKLDISYNQLISSKFSSDFAAMSSLRELILS
GNNISSLNYSDFQPFLLHGFDLLDFSDNPITHIDKSFFTLFSFLHILDISGLPILFSNLQEALEGLTSCNITAIWLDN
YNGLPIIQAGSFACLQNTSLEILTLENSNLKEIQDNGFCGLNHLKDLVLAGTKLTELPRLAFHGLSNLQFLDLSVLPL
THIPKAALSEVAQTLRELQMSTTGVEIIQTDEFNDLSNLKIINLGYGILSDIQPFAFRGLGNLENLQLHVNILKNLLD
NTFAGLDKLKMLNLSGCSIRSFSNKIFANLTSLVKLDLQQNDLVHVPTLVFSDLRSLEELSVAGNHISPYDSIDLTSL
ASIKHIDLSYNDLLLRETTFDFPANTRLKSLDLSYNKLFSYSSTLPCFGPCTFFKKLQNLTELDLQGNKVTPQRGRGV
IFHGLVSLTTLIMRETGEIWSSFSNETILYGMPNLEVIDLSASSISYIPAELFKVHDNLRSVDLSSNKITYLPRTLFS
ANSKLQTLFLSRNLITLLNESSFETTLPGLKNLAINDNPFFCDCEIEWFISWAENHSSLVQGWSDGRYQCNTPPDLHS
TDLRNFHPDCASHRDLYACAITTSFLLLYMLFAVLVNFCSGYFAYLWFRVRLRLRGYEEIPEQPQQFRYDAFVAYSSN
DEAWVSRVLSPMLERRPPRYRLCIGERDFVGGVPILHNISNAVETSRKTVCIITRSFLRSNWCNYELQMSQGRHHLFD
PRRVSLVLVFLENIPDRVLERYPLLNNIVNRDTYLRWPNNQQHLPLFWARLLQALGPPLGDDLQEDDGIEEDVV

>Bl52875c

MTSLRELLLSGNDISSLNYSDFQPFLLNEFDLLDFSYNPITHIDKAFFTLFKAIETLDLSNILISFSNLQAALEGLND
CNKISLDSYTGLPVISYGSFACLQNTSLKSLKLSRAEIQEIQDNSFYGLTHLENLGLQWNLLTELSGLVFHGLSSLQV
LDLSFVSLTHIPTAALSEVSHTLRELTIFSTEMKTIHKDDFNNLPNLKVLYLNADFSGGLEEIEPFGFRGLQNLEVLQ
LDDNDLTHLVENTFAGLNNLTKLSLKRCNIKRLSGRIFANLTSLVELDLSVNNLVHLPKMLFSDLISLEVLIINNNYN
LFPYFESIDFTGLASIKHIDLSHDGLFLDNQTLNFPTNTRLETLDLSYNQLFVASLPNERSVFEKLRYLTELNLSGNK
LAKIPAYGAIFHGLSSLIKLIMKETGEIWSSFSNDTILYGMPNLEVIDLSASSISYIPAELFKVHDNLRSVDLSSNKI
TYLPQTLFSANSKLQSLFLFSNLITLLNESTFETILPGLEKLAIHDNPFFCDCEIKWFMSWADDHPSVVQGWLDGSYQ
CNTPPDLHGTDLQNFHPDCASHRDLYACAITTSFLLLYMLFAVLVNFCSGYFAYMCFRVRLRLRGYEEIPEQPQQFRY
DAFVAYSNNDEAWVARVLSPMLEDRPPRYRLCIGERDFIGGVLILHNISNAVETSRKTVCIITRSFLRSNWCNYELQM
SQGRHHLFDPRRVSLVLVFLENIPDRVLERYPLLNNIVNRDTYLRWPNNQQHLPLFWARLQQALGPPLGDDLQEDDGI
EEHVV

>Bl52875b

MFEDLAVFSISKSVEVPTPAMATGEKINSNSLRLFGLRMFLTLSTVLALCTRTQGTPCTIVNKTATCSNGGLTQIPSN
LPHNLTNLDLSNNDIQAIRNNSFSSLHFLEVLNFGRNNLSVIEPAAFYNLSSLKTLLLKENRLSYLPPGLFAPLSTLQ
NLYLSDNSLQNILQADVWKGLNLKKLDVSYNQIISGTFRSDFAAMTSLRELILSGNNISSLNYSDFQPFLLHGFDLLD
FSDNPITHIDKSFFTLFSFLHILDISGLPILFYNLQEALEGLTSCNITAIWLDNYNGLPIIQAGSFACLQNTSLEILT
LENSNLKEIQDNGFRGLNHLKDLVLAGTKLQELPRLAFHGLSNLQFLDLSVLPLTHIPKAALSEVAQTLRELQMSTTG
VEIIQTDEFNDLSNLKIINLGYGILSDIEPFAFRGLGNLENLQLHVNILTNLLDNTFAGLDKLKMLNLSGCSIRSFSN
KIFANLTSLVKLDLQQNDLVHVPTLVFSDLRSLEELSVAGNHISPYDSIDLTSLASIKHIDLSYNDLLLRETTFDFPA
NTRLKSLDLSXTLDLSYNKLFSYSSTLPCFGPCTFFKKLQNLTELDLQGNKVTPQRGRGVIFHGLASLTTLIMRETGE
IWSSFSNDTILYGMPNLEVIDLSLSSISYIPAELFKVHDNLRSVDLSSNTITYLPQTLFSANSKLQSLFLSRNLITLL
NESSFETTLPGLKNLAINDNPFFCDCEIEWFISWAADHSSLVQGWSDGRYQCNTPPDLHDTDLRNFHPDCASHRDLYA
CAITTSFLLLYMLFAVLVNFCSGHFAYMWFRVRLRLRGYEEIPEQPQQFRYDAFVAYSSNDEAWVSRVLSPMLERRPP
RYRLCIGERDFVGGVPILHNISNAVETSRKTVCIITRSFLRSNWCNYELQMSQGRHHLFDPRRVSLVLVFLENIPDRV
LERYPLLNNIVNRDTYLRWPNNQQYVPLFWARLLQALGPPLGDDLQEDDDIEEDEA

>Bl52875a

MIFHGLVSLTTLKMRETGEIWSSFSNDTILYGMPNLEVIDLSLSSISYIPAELFKVHDNLRSVDLSSNTIMYLPQTLF
SANSKLQTLFLSWNSITLFNESSIETTLPGLKNLAIHDNPFFCDCEIEWFMSWAADHPSVVQEWSEGRYRCNTPPDLH
GTDLRNFHPDCVSHRDLYACAITTSFLLLYMLFAVLVNFCSGHFAYMWFRVRLRLRGYEEIREQPQQFRYDAFVAYSS
NDEAWVSRVLSPMLERRPPRYRLCIGERNFVGGVLILRNISNAVETSRKTVCIITRSFLRSNWCNYELQMSQGRHHLF
DPRRVSLVLVFLENIPDRVLERYPLLNNIVNRDTYLRWPNNQQHLPLFWARLRQALGPPLGDDLQEVEDIEEDVV

>Bl72403

MTNTTVDCSDRGFIQVPPNIPTNTTSLDLSSNDIQQLDNYSFSRLPLLTLLDLSSNDMLIIEPVAFYNLSQLVELSLS
INKLSTLPSTVFEPLKSLASLSLYDNEFSDILGFDKIWEGLPLTKLDLSRNKMMKAEFSKAFSEMHSLKLLDLSLNRI
SVLKIADFEPLASHSFQTLELSANPITFVESGFFAQFHRIEYLGLLITSSNITILRDAFSGVHSVKVTQLALGIADFF
IVNEDAFQFLKNTSLQNLVLGWGDIKELKDYAFRGLDDLETLNLQNNQIEEASPQAFGGLHNLVVLDLRENHFKQVPF
KAPSVVFPALRELHLEDNKIKNIVPENFIGLPNLQLLKLDNNLVTNIPANVFQCQRNLDTLSLQNNKISLLSNRSFTG
LENVTLMDLSMNKLPVLPGSVFFPLIKLKKLDLSYNYLSPEMEDFYGLEKIETLKLRRNDISLKWNTFKLVSTLTYLI
LSENDLFGGIPNHKPFITMRNLVTLDISNNQIKFQSDSMREFFEGLVSLRSLQMYRTGDIWARFQNATILYSMPNLEM
LDMRECKIQYIPPNAFSIHVNLQYVDFSDNAISSLPDNLFLNVRSLKALYLETNAISYLNESSFATVLPTLESLGLDL
GENPFYCDCRVTWFISWADANPSKVLFWEKDGYYLCNTPASLHNRDLRTFHPDCDSHLNLYMCVVTTSLLVLYMFTVF
VVTQYSLYVCYIWYLLRAWLRGYEEIPDMGVQRFEYDAFVAYSSKDKRWVSQVLRPQLEDRPPHYRLCFGERDFRPGV
PITKNIGRAVRASRKTICLITRSFVRSNWCNYEMRASEGRYHLFDPRRVNLVLVFLEEIPDRDMERHKHLRDVVMRDT
YIKWPRNEKGRPLFWARLREALGQPLPVNQGGNRDIFEDSV

>Bl19440

MTLRLVALVCLSTWFLSGALSATNGADPLGCQVWSLTDVTCANTLILKDVPPDLPASILRLDLQGNDITQLNRQPLNA
LKNLQYLDLSWNRIEYVENGTFDSMTNLRILNLSRNGLGDSQWSNITSYLPSLQILILSRNRLQDLSRGSFMGLHNLT
FLDLWSNAIIVGECAFCGQQRLQRLDMTGNEISTLPNRLFHPQSEIQVLDLSNNRLQDDVLEKEELWSGLGKLRTLDV
RRNYFSIPKFAQSIRNLTSLHNIYMSSNKIQQFLFQHVAPLLNAPIHEFVLGYNPISIFEEGILLRLRHLKTLELGQT
SIPSSELQKILPELQNTKIRKLELGTAPGNFAIDNTTFGQLPGLEELSIYGSDLSVLKAEVLRHSKNLTDLALNSNNI
TFIDERAFIGLKSLEKLQLQRNNIQTLPDKVFDPLFALTYLDLSSNSLNTISPALFKGLLNLQWLDLSSNGITKIDNT
SFIGLSSLTHLDLSYNRLTTAGLPVVGPSVKSIDLKANQIEILKANEFLGFENLGSLCLESNNIRSIEPHAFQGLGKL
EKLDLGGNKLRTVIGRMFEGLENVTVLDFDSNTLLEIEIEPYGFYGLRRLKRLEIGFTLRAIPSYAFVGLDSLTFLDL
SHNQIAKVGRHAFDGLPNMKIFLFVKNKITVFEEETFGRVVRQASRIQFEQNPFYCDCRLLWFVEWARKNPDKLHFFT
FGWYKCAGPPEYADTRLVNFYINCTSPEDNVFQPNRPLACALVSSAILFYMFAVFLVSYHHWKIKYLMFLLRRRNEDD
EPMRGRRFVYDAFVAHNSEDIRWVVHELCHNLENVQDQPRYKLCIHQRNFLPGAPIVNNIVKAIETSRKTICVLTRSF
LWSGWCEFELQLAQTPDNLFGKGGSCRLILVFLEKIPRPLLKKYRHLEAVMDRDTYLEWPGDARGRPLFWRRLRAALG
KPVNVEQAGDGGMGDEDDELEPLLRM

>Bl21810

MESKPALLAVCLAVVSAFIAQRVRGELLPPVCQIWNSTTVVCTRGVWDDPNRAPLTQVPPGIPESVIALDLSHNNITE
LHNGSFSGLRNLKSLDLSHNSLQIIEVGTFAGLENLERLDIGNVTLMPQQEPMYALQSGLFRDLRNLNYIVVQTWTDT
VSEGVFTGLVKLRHLDLSVKNISNLPDHVFDSLTSLESLTIEELAIRDDIDEDQHTAPTRVHSGSGFLQRRLLWEPLY
NLHKLSLYYLPRASDLYFGPVFRNLSNLETLEISLLHDYSLSVQMFRPLLLTLRHLHIHAPIAPGLLKSLTHLQTLDV
DLVPFPAIVDVLPELRHTEIQELSFDMLGFGMDKITSDTLAGLRGLKHLKGLFLYLDISKIETVQSNAFVGFSHVQRL
DLTNGFLKTLQDKTFRGLSSLTHLNLTHNTISTLPAGVFEGLTSLTHLDFSKNNLIASQAQLPDTLDYLDLSNNRLNS
KFTGQIPCAYPLSFNFSGLTSVNFLNXPHTHRHTNYDNKPSRRSRLILFTNRHTDKHRRITFSTKPFAAKVTIERQGQ
SNRKNILWHRLQSNDRDNPTERTFFGIDCSHGFRPNRLLACVLASSGIFVAMMTIFLVDYNIGHVQYYLWTWAKWRGP
KIGEEKNQEPSKYTNDAFIAYNNQDVMWVVNEAIENLEPDFSLVIHERDFAVGAPIVENIADAVENSRRTVCLITRNF
LKSKWCEYEFQLAQYHMFEAGGGKRLILVFLEWIPDRMLKRFRHLNAVMKRDTYLVWPGDVRKRPLFWKRLRHALGDP
LPRDPEPQQQVHNLERNIPEQDPERNIVEVQIHGPMWNIPEQHALAIQVHVPINGNIPEQELQAPERNIPEQDDGILL
PEPDDQWFCGREDGPLLPM

>B112652

MESKTTFLAVGLAIFSALLIPHQGRVIGQPLPPVCQIWNSTTVVCRGEYPWGKLTQSTLTQVPPAIPKSVVTLDLSLN
EIRTLHNGSFNGLRNLTYLDLSDTDLHTIEIGAFAELENLERLIISKATTEYPFSLQNGQVFKGLYNLRYLDLSVNFV
SNLPDHSFDYLTSLEEAKIVEFGWPNEDRNTTIERTRDGSGFLQRRLLWAPLKKLKTLRLYYLKRASDLYFGPVFGNL
TNFDTIQINSSDTVHFDIQMFQPLLSTLKHLSLQMNRITLHIQPELLASLTNLQTLEFPKWVPFSYISDLLPELNATQ
IQELAFATRGIDTITPDSLEAIKGLRDLKALSLRSVQAIRANSFTAFSYLQRLRLSEGSLETLPDRAFSGLSSLTHLN
MHDNHISTLPLGAFEGLSSLDHLDLSKNRLETSEAQLPETLDYLDLSHNDLNNDVSSWHLRRCSNPISFNFEGVKSVR
YLNMSYNKLAFVDIECYPGSVVVLDLQHNGIYYVGGIDINPVFVINSLKYLDLSNNDIKNSWPDVRIPTTTLETLKME
NNAIKSVNWGHMGGLVRLKMLTLSHNHISFIGRGDFQFLVQLTHLDLSHNYINTIRSSAFRGLSRLQFLDLSNNQIQN
ITMMKFEHLGNLTYLNLAANRIAVIGDAFQHLLALRNLNLSSNRLSVLNQTIVGPIVQRLENLDVAENPFLCDCNLLW
FVEWAQDRYDRVVNWHNPYSLPNRRYTCSRPAKLSGQRLIDGMANREGHNYRQEPAETRFFDTVCIHGFQPNRLLACV
LASSGIFVAMMTIFLVDYKIARVRYYLWKLAKWRRPKIGEVENEDPPRYTHDAFIAYNNQDVRWVIKEAIENLEPDYS
LVIHDRDFAVGAPIVENIAHAVENSRRTVCLITRNFLKSKWCEYEFQLAQYHMFEEGGGKRLILVFLERIPDRMLKQF
RHLNAVMARDTYLTWPDDGRERPLFWGRLRDALGDPLPRDPGPYQQEQAPERNIPER

>B156664

MRGILLFMFICYVGCHGNANVATTTVSNIRPTLSSNGDVQVFRSQLWVCALQYSISTYTCTGSDITTLYIADVDFFDA
TVTKLTVNCIYKINHSNFTVFDNLPSYIKTVRLWDCFEEEIGKEVLLGLAHVDFLDIGNILSIFTDGYDLHTEFSRIK
EPLDPVRLDPDLFASVPKLKKLSIHWLYMDAFPEAIYHQTNDQAHLKNLEELDLSFNEIPEIKPEYFKNMPKLRSLNL
SVNRINNLSDSFLMMQNLQSLYLTGAFIDSLDGSPFNALPELGTLVLSLMRPIRNVTLPGPAGQKYPVRGSLQFIYPS
SFAGLSNLRILDLHHHSILLIQNGTFLALGSLEQLDLSVGLVSAIEENGFEGLQSLTSLDLSYNNLSRIGTSLFHNLP
ALLSLNLEGNYLDSLSKNMFLGLPLLTSLNLGYNNFIDIPSDVLRPLVRLKLLSINHNYLKSVEGLLSGLSSTYCEDI
DISFNNISTLDASILPTLAVAERMITLNLSHNAITTVYATNSYFDISASQSLKQLRLTLDLRWNRLLSIPFQLDRFSF
FSLPRVVIDGNDHYRYAKIGLHHQLTLQIRHNPIICDCTIYELLQNIATAQRGVLYANTDFKELTCHDPEDLRGIKLE
DISPSQDDLSPSQTWCQKWDSSDCPSACDCMVQGEPNSTISPWNELVDCSGKNLSVIPDGIPNAATILHLRRNNIRRI
QQTAFRKAPLTRELYLSDNNITKIDSAAFIHIQSLEILYLDGNDIEEITGYEFVHLENLRELYLNSSEVRAVNRDAFG
HLPLLEVLSLKDNLLNTLPDGLFSALSNLQHLSLSENSFRCDCDILWFKYWIRNKESLLKEENITCTHEHNQVQENIE
TLLSDSLGCDLQNTERKSKITIGFSIVVGLLSLIILVGFVLYRKKGDLEVYLYSRYGWRFTEDNEDLDKPYDAFLSYS
QHDLDFILQHILPGLENREPPFRVCLHHRDFIPGVPIAENILNAVEESRRTIVVVSRNFLDSDWCQLEFQAAHAQVLR
ERANRLIMILLEDIPADDAPPDIKHYLQTNTYLKWGDERFWERLVYVMPRPMHSEELVQNMEQEPLEIPEPSQQTTTV

>B120861

MPLSSLAGPETFPRLDNLKLLDLSKNRIKIIKSGSFKNTYSLTTLHLNHNNISTLPNGAFDGLASLEVLGLAFTRLSN
LNSVGFLTPNLKSLNFTSFAGNGVTQCKLGDEFRNLKRLTYLDLAYNKISDLERDCFASLNESHVQTLDLSFNNIKVI
SHPVFWPFRNLSFLFLGGNKFDLTELNTTFDNINSIDTMSLYLSFYNSTFLAGAKTPLNTTFARLAALPIKHLDLSYV
GLRRLPEIGLFSFFPRLESLLLVGNNITELPPKAFIGLGSLKTLDMSKNKYFPNVKKGSFASLANLTSLSLTLAKGRQ
DIPGSVFLNLPNLRSLAISGPKGKFAVGKLTMWSLRGLSYLENIMIEWHDLPVVPGPALELVKGTLQRLSFLHGHISE
IEPRAFRGFKHLNTLDFRYNKLDALPDYAFDGLENLISLKLSYNYIEKINKTAFFGLVRLEILYLESNHLCFRAETDF
PPPFTALQSLISLHLEEQSSKCRFYGGILSFPPDFFSGLVSIKRLHLSKNKLRMMLFREKTSRPFANLTTLEVLDLSY
NNFDTMTALPFENLKNLTSLYLSYNRILKIPDNLFKSVPNLRKLRLNSNRNFGKLPKDVIYLLPKLERLDLEDNPLQC
TCEEEWFHDWVVSNRTKTLFYNLSNYGCVSPERLVHKTILDFDAEAQGCNDKTGLHVAISGAILLCVFLVGVVVGYRN
RWYIKYGCFVIKARYHGYQTWTNENLQKKFDAFVSYNHNDRAWVMNELVPHLEEDGEEFRLCLDYRDFVPGAPITDNI
VNSIYDSRKTVCLVTEEFLKSEWCEMEVQMATYRLFDEQIDVLILVFLEDIPDRALHRYHRLRRLMCKRTYLEWPKDP
QEKALFWERLKDALKTGDRPPIENII

>B148785

MEIKYPGLMLSVGLLLLLFSCSCNTGASAETPCTCELRHGEGGVPQSSAFCQCPTISSLSSQDIPNYDNITTMVVSCQ
AVDSPADLSLSDLSSIQSLSLYNCFYVVLNSTFRSLPSLRKLVLDGCDLDNDPCNYFWVRSDSFLGLPNLEELKLLAA
ADVPKLRGLPSLESLMLAYIYQGVLIKNYTLVIQYPNLLQFTLTGSVSETHKVDILLLDMPLLERVNITYNPQSLNWI
VLPENLFKHTPGLKELSLTHDSIRVLSQFVLHPATNLTKINLSNNNIRYVPPRFFEGQDKLVEVDLSDNQIWYIAHNT
FSDLAAMEILDLSNNVLTSLETGTLKGLFNLGIIDMSKNSLGHLPADIFADCTNLTELQLSNNNISSLDDTIFHPLTK
LEGLYLNYNELQHISGRLFQNQTSLQVLQVGVNRLQAVDFAWFSHMSALNQLLLPINNIQTVSSWGVLKSSFRGNLQV
DLVANNINRLPYHEIIQLASLGQSKPQLSQVLLNFNPYNCDCEVLPVNKVLSLPRATETFPDLVNISCWNPPELRNLQ

VSHLPPSSFKCFFKEQCPESCSCYSQGMKYTDDALHRIVNCSATGLTQVPSMFPENLTLVDLGGNSFPKLTVQSFLNF
TDTKTMILSKNDISAFEPGTFKNMNSVRILYLDGNNISTIEEDTFQGLDSLKVLFLNNSGVTNVHPSSFSHLRHLKEL
HLQDNDLEHLSKQTFKRVSRLQVLYIGQNPLKCNCDLLAFKEWAETNVQSVSFEFNVTCSNHGNKVFTSVAHVVKREL
SCTPGNQYVYVILVLGMVVALLVVVLLVYQYRGFLQVWLYMKCGWRFDPKDDGDDKTYDAFISYSSRDELVVIRELAP
GLEERGFNLCLHYRDFPVGACIATTIIETVETSRRTIILLSQNFVDSEWCALEFKAAHRQVLEDRRNRIVVIVLDDLE
LQNVDKDLQFYLKTNTYLKWGDPWFWSKLCYALPRVGRGADKQSTDSEHIDMKDVTSQDSGIEMTEVYDNHL

>Bl24356

MSGDLSGGKRQWSSWLRGGAAGKHCVSCNNLNKKGLFLSWYQYTVFLCYNLHFHNLRFVTKHFHLIFICRRHQKTRNM
FRVLPIFVILCICATSGGITTDTPRTGPIQGNGSQPPRLCDCVDVREPSLPLFQFTCLCPDIFSLNSTQDRLYVSRYL
TAAPEFTHFTVQCSNRSVMSPSRLFDMLPASIKTVGVNDCFQHYISKDLFFGMSHVEGLFTTNNPSNFYEKVITGIYK
ISTIIPVELDPRLFLHVPQLQALNLVLLLLDRFPEALYQGVNGTYPLQRLRGLNLQLNNIAYLKPEHLRHLPNLESLD
IKYNNIKNCSTSFPFLPSLQMLILNVNQIRSLDGSPFQNLSALQSLDLSRQLAVEGQASKVIYLLNSIYVSKHLNFHS
ELPVTNLTYNDGVMESIFPTSFLGLSELKFLTLAQSYIKLIPNGTFKDLVKLEQLNISDGLVDHIGESAFQGLGVLKI
LDLSYNNLLNSTIDPSIFESLRSLLSLYLQGNVLQSLSQKTFENTLYLEILNLGNNRLTTLGQYTLRPLLNLRTFDVH
GNQLVYLEGMFHGISIGVCEIIDASYNNVKALQNLGVAGKTVNVDLSHNNLEILYTTNYYMEENRPFLSLRLDLRWNM
FTSLPFELAAYSSFNRSRISIRFPDESLNSYKKGGDISILMKSNLLVCDCLLYELMLNLDVAKQGALYTKTDFQDLEC
AFPDELSGRKVIDVSPSELWCSEFCFKRPYYLCSCFEHAGEVLASTIPGCWVEQTVCPSECFCSYQGKLHSSTAPYNE
LVDCAQRNLSSIPGNITNITTILHLEGNHLRVISQDVLPQLLMIRELYLNDNNISYIGNQAFNDLLSLQILRLDGNNI
SEINSTVFKSLSNLRELYINHSGIRYLVADTFQDLASLQELHLENNRLQSLPENMFAGLKNLRLLGIHGNPLICDCDI
LWFTNWLRSRVSLLSQARNVSCFAKTKVRRNILSLSSAQLDCDDLLAAQARTRLIVGLSIPLVLVMIILVCGIVLVKR
KEAIQVYLYARYGWRFREEEEEDEDKEYDAFLSYSQHDLDVVIHDVLPGLENRDPPFHVCLHHRDFLPGIPIAENIVN
AVNASKRTIILLSNNFLESDWCQLEFQAAHAQMLQDRANRVIVVLLEDVPEENAPPDIQHYLKTNTYLTWGDERFWER
LVYAMPRPRHAQPVQNEDGDQLALVELDHNA

>Bl18798a

MRTTTLILLLVLYSVQIHRLASTRKGEWCSYKNWEFSCKKKGLRHVPVLVPVRARALDLSINYIPALYNDSFAGMDKL
TILVVSRNLIMDIEAATFWNLKSLTLLNLRDNRLQSLRPHVFKTLSSLQVLQLDQNMLKSTAAISAALPPLTSLYSLV
LSSNNLSHVKLEPEFASLNKLTALRLIGTSISSLQTESFQVMKNHSLSHLSLGGNYLVNIPRQALDPFASIKQIDLAN
SDLTPKDLFTIFNNTRGLGITNWTLSDNSLTELTNITFSPLIDEEVIYIRLNSNNISQLTDYLFAYVPQLRVLSFGQN
PIERLSAGVFAGCDKLEELGLDNWGLKEIPSELFEPLKNLTKLYMDSNPIHKVRGDAFVSLPKLKTLRLNANRIETIE
KNAFYGLHHLEELNLGSNQLRQIPYVAFSKVGPSLKKLDLSANRRLEAISPFSFNMLQNLTDLSLQSCKIKRLGTNDF
AGLYNLQNLDLSFNLILTIDPKAFNNLLSIKVLDLSKNHLGLYIRENPSISPFVNLTSLTELHFNYQKYTAGVFPDGY
LDGLTSLRLLALMSQKLVSLESKTHKTSILKNLKNLRYLQLSQNKLSNFTRDAFAGLTNLNYLYLTSNSIRNLPPGIF
RHQGRLQYLDLSYNGIVTLSSMVFSPLKSLVVLNVYSNSFACTCDIEWFHDWIASSMNATTGVQGVYFEAYTNYTCAS
PKSLRNKPLIDVNFDKLGCKSKLEIYLAIGLSVTFFILMVSIILMYRYHWYGRYAMFLLRAKFNKYEIIREEEENPKT
YDAFVAHNSHDSAWVIRQLLPQLERGDPPEFRLCLGDRDFQPGAPIVDNIAESIYESRKTICVITRNFLESDWCRFEM
QMATYRLFEEHVDCLIVVFLEQIPAQRLAKYHSLRRVMCRNTYLEWPEDPEARDLFWERLRVALRTHRPLNHDFN

>Bl18798b

MMKKARLTMVLVMCSLAVCMSAATRGVQWCSYKTGVLSCVKMGLRHVPILVPRQTEELHISINRIPALYNDSFVGMGQ
LEVLDVSRNLITDIEEGTFWNLKSLRLLNLGGNRLTVLRSHVFKTLSSLQRLQLDQNMFKSTAAISSALLPLVSLNVL
TFNKNKLSRIKLEPEFAKLNKLDILDLAEMSIPSLQAESFQVMKNHSLTHFSLGGNRLVDIPQKALKPFVSIKQLDLG
YNNLAPKVLATIFNNTRGLGITNWTLMNNALTELTNATFFPLFDEEVIYIRLDGNSISQLTDYLFAYVPQLRVLSLKN
NPIERLSARVFAGCDRLLHLGLNSLDLSEIPSGSFEPLKNLTKLNLDSNMIHEVRDRAFVSIPKLRILWMNGNRIEMI
WKNAFSGLHHLEDLNLGANNLRNIPSRALRVIGPSLKKLDLSANRNLKTISPNSFNRLHKLAVLKLQSCRIRSIGNST
FAGLYNLQRLDLSYNKITTIDSGSFHDFLSIKVLNLNDNVLGSHESSSPFRNLISLIELHSDYQKDTAGIFPDGYLDG
LTSLRLLSLKSEKLITLETKTDKTSIFKKLKDLRYLLLTQNKISHLTRDTFAGLTNLTYLYLGSNSIRALPLGVFRDQ
GRLQYLELRYNGIVTLAPEVFSPLQSLVMLNVYSNSLACSCAIEWFKDWIASSMNVTTGVQGVYFEAYKNYTCASPKS
LRNKPLIDINFDILGCKSRLNFYMAIGSSCTILILMMTITLTYRYRWYGRYAMFLLRAKFNKYELIREEEENPKTYDA
FVAHNDNIAESIYESRKTICVITRNFLESDWCRFEMQMATYRLFEEHVDCLIVVFLEQIPAQRLAKYHSLRRVMCRNT
YLEWPEDPEARDLFWERLRVALRTQMPLDHEVNA

>Bl24343

MARFHPVLLLIIYAIIRDVAMDTAESSPDGGDSSPFVWQTLCNCSQTSPVSFGCLCPDLSSLTNVQDRLVVSGYLSAF
PEITEFGVTCKQKSSRITSGLFDILPASVKKLTLGDCFLHYIGKEVFHGIPNVEDLVIENLKLGGNDSESSQSLPHVE
LDPQLFVSLPQLQKLTLDYLLLFRFPKALYQEVNGSYPLPNLKSLTLQGNQIPYLKPEYLRHLTNLRSFDIEYNRISN
LSASFPFLPGLRILNMAFNRIISLVGSPFQNLNNLQSLDLSQQYSFDNDTSITIINIGALEFIFPTSFLGLSKLKSLR
LAWSKVRSIPNGTFKGLVELEKLDISYGLVDHIGQSAFQGLSLLTSLDLSYNNLSSIHPSVFNSLSSLLSLYLQDNVL
HSLSLKTFEKLPYLSYLNLGNNRLITLTREIFSPLLNLRTLDVHGNQLNNIEEVFAGINSDFCEKIDASHNKLESLDT
SSLQLLGIAGKTVEVDLSHNNLRVFYSAYYSSSDVIHLSLALDLTWNQFTSLPFMLWIYESFNRSKISIANGLGGEYG
DISLQMTHNPLTCDCRLYELMLNLAVAKQGVLFTKTDFRNMECRYPDTLRGRRVVDLNPSELWCPGECYNRPGYCFCF
EYEGEIIKSSFAKCQPERKACPSECSCSFQGQLHSSTAPYNEKVNCARRNLSSIPDDISNATTILHLEGNHLRVINQT
VLPQLLMIRELYLNDNNISIVGAMAFRNLTPLEILRLDGNNISKINSKVFKSLSNLRELYLNHSGVRYLADDQFDDLT
SLLELHLENNRLQSLPENMFAGLKKLRLLGIHGNPLNCDCDVLWFANWLRSRTGYLMSKGRNVSCLTNTQVRRDILSL
SSAQLGCDDLQAAQARTRRTIGLSIALVLVTILLVCVIVTVKRKEDIQVYLYARYGWKFREEEEDEDKEYDAFLSYSQ

HDLDVVLHDVLPALENREPPFHVCLHHRDFLPGVPIAENILNAVSASKRTVILLSNNFLESDWCQLEFQAAHAQMLQD
RANRVIVILLDDVPAENTPPDIQHYLDTNTYLKWGDERFWERLIYAMPRPRPHAQNVDGDQLAMVELDHNR

>Bl19922

MRTRPVLLLCVFVAVAPQVVRQVTGTTLVLTPDEATAASCTEWEPNADNVTGLATDCSRRNFSAVPSNVSYFVVVLDL
AHNRLFFLNRDSFSGLARLKVLSLSYNQIRTIEHGSFSDLSELSELYLDHNGISSIANGFFTGPSWLENIGLSFNRIA
YIEDSALDEVGTTLKQLKLGNNYLGRLLSLEKSYRIFQQLENLTHLDLSNNGIYDFRSDVLRSQSNIWSLNLSGNALR
DKSLFIEVQNVHNHSGSGEYMYAQRTSVFHGLSSLKSLDISHNRFKLCCEAYNGLTNLAYLDLRSNDVSYIEPENITK
ALPNLNELHLRGNPFDCSCRAVPFFIRWVDDSVVDTDILNWTCQRPPSVAEAKVAELDIRGCGQDLKYNVLLTAFIPI
CVIAIFAIGAVIFYRRAKRANFSKIEAFENIELEDRKYDVFICYSSHDADWVHRSLLPHLDSLHVEYCIHLRDFAPGE
TIVENIVYAIEQSCQVIVVISGNFLNSEWCTFELQMAEHRHFTTGEKYIIPILLDEVPVNTMPKSLRYLLATKTYIEW
KGRGEEEDLFWRRLGKALKSRLLIKRTNNDAIYMEEEATIMPESPTSTPKHRSDTVNLCWVKAAQARKFVFEFRPRAW
PAARPNSNMVRNVPLRSISGSARSITQHNHMYHLRCTYKHKIAL

>Bl17405

MSWLLVCFLLGHSAGMYSVSAIRNDAFPCWSLNTTFVDCSALYLEHVPPLPQTTTSLDFSDNRIRKLANNSFYGLDNL
LQLKLCTNGITSMEEQAFANLQQLEELNLKENPLVYIHPEVFLPLTSLRKLDLSTGRLTAIPEALRMLHNLQDVNLAR
NYITSANLDIFRGMSKIQKIDFQGNLIVNISAIDFRMVSNSSLRALSLSATVKSLSHIQEGALAALREVQELNLSDID
VEGRMSLFLKYTCELTYGTVKFLNLIYMRMYIYRSGFFDCLPRTIQTLWLDLNIIENLHKNLFVRLKNLQVLRLSQCW
MSSIEMGAFDGLESLKELHLSGNKLTALDPHVLSPVSASLEVLNLGNNLNFQLQPGHFKNLTLLKILALQNNGIRSFE
SDHFKGLGNLIQLQIGYNQANYKPTKEGIFRYIPKLEVFLAKKNDAAEFALDAVLSAGLDSLRVLDLDSGSVNILNDR
GRSINLSDLQVLRVGNSADDITITYAYHYRADLLWNLTELQEIDLSQNGFQNIKKEAFWHLKKLDTLNLAGNYLASLH
PSMFRDAHSLRVLDLSYNRITAISPTLFQNVNYLRQLSMQKNLITSIGPQTLTYWNKFNTLYELDLSGNPFSCTCQLL
DFVEWARNNTSVRILYYSSTGGYKYYKCSSPPDLKNLPLLDYKPDCKSYLGYYTCIVMSTLIFLYITMTYVMVKYHAY
IRYLYQYARGKLRGYQAIPHRHRYEYDVFVSYNNEDIPWVQQELIPHLEEVEPHYRLCIGDRDFLAGRDITTNIVEAI
QGSRKTLCLLTQRFVRSGWCTLEFKIAQHRLFEEGEDVLVLVLLEDIPAHVVQRYNRLRQLMSRKTYLVWPEDERARP
LFWVRLRKALGVGNVLPYEEDV

>Bl04519a

MESKPVLLAVCLAVVSAFITQHGRVGGLTPAPWQLDPPVCQTWNSTTVACTGENTWSPTRALLTQVPPGIPESVITVD
LRWNNITGLYNDSFSGLRNLKSLDLSGNVLQTIEIGAFAGLKNLERINMSIRGWDDIDGLIWLRELRPMYTLRNGLFQ
DLRNLKYLWVTTYTDTISAGVFTGLSNLRHLELRVRDVNILPDHMFDSLTSLEILTIREHSNVMREDHTTTTRSGFLQ
RPLLWAPLYNLKKMSLHLRPRISNFYFGPVFRNLSNLESIEIKAWTSNDYALSVQMLRPLLLSLKHLHTDFPIAPGLL
KSLTHLQTLNLDPLHPWLILDVLPELRHTQIQELTFQAGVSGMDVFAGITHLAGIRGLKKLQTLSLTGSISKFDGFMN
FSHLQRLDLAQSSLNYLEDKMFSGLPSLTHLNVTDNAISTLPESVFQGLTTSLTHLDLSHNRLETFLAQLPRTLDYLD
LSYNKLNNRYIGQTPRPYKCLPPLAFDFSGLKSVNHLNLSHNAITDVNGNCLPRNIDVLDLQHNNIGQISILCTTRYL
DLSNNHIHSFVDQEECEFNNALETLRLDNNNLEDIYVPLPWLIHLKTWTLSHNHIKRIKTGQFTVMDQLEHLDLSHNE
IKTIQKFAFHGLSRLRFLDLSDNKIQFITEMTFEGLGNLAHLKLASNKIAVIGNAFRRLYELRNLNLRSNRLTVLNQT
TLGPVVNRLETIDIADNPFLCDCNLMWFVEWANDKYDRVPNLFNPYPDFARGYTCARPVQLLRRRLIDGLTQEQQSKV
KNDPSERKFFDTVCSHGFRPNRLLACVVASSSIFVAMMTIFLVDYHIARVQYYLWQLAKWRRPKIGEVENQEANRYTH
DAFIAYNNQDVMWVVHEAIKNLEPDYSLVIHERDFAVGAPIVENIADAVENSRRTVCLITRNFLKSQWCEYEFQMAQY
HMFEKGGKRRLILVFLERIPDRLLKRFRHLNAVMKRDTYLTWPGDVKKRPLFWDRLRHALGDPLPRDPEPQQQVQDPE
RNIPEQQEQAPVRDIVAVQVHKPMRNIPEHQILLPEPDDLWFGDEDGVPLLPL

>Bl04519b
MESKPALLAVCLAVVSAFITQHGRVRGQLPPPVCQTWNSTTVVCSGKESRWIQWEQKPTRVRLTQVPPGIPKSVIKVA
LLWNKLTVLYNDSFSGLRNLKSLDLSGNALETIEIGAFAGLKNLERISMGVAGWDDVISGSLWTRELRPRYTLTPIYT
LPNGLFQDLRNLNYLRVTIFTDAVSAGVFTGLSKLKHLELSVRDVNLLPDHIFDSLTSLEILTIREHSNATGEDQNTT
ARRVNSGSGVLQRSLLWAPLYNLKKMSLYLLPRTSNLYFGPVFRNLSNLESIEMNSGISNALSVQMFRPLLLNLKHLH
TDCLIAPGLLKSLTHLQTLDLDSAYDLRISDVLPELRHTQIQELTFLVGLSGMDVLGGIRGLKKLKTRTQWHTIERFY
EFLTSTKAMNFSHLQRLDLTQSPQNELEDKMFSGLPSLTHINLTENYIYTLSRGAFEGLTLLTHLDLSYNNLKTSQAQ
LPETLDYLDLSFNNLNNRDIDQIPRARECFYTLSFDFSGLKSMNYLNLSHNEITDVDSSCLPRNITVLDLQHNNIDDV
HKTPRARYLDLSNNNINSLNLYSSNELSIVETLRLDNNVLIAIDSLWIPQTLKTLTLSHNHIQSIKTEPFQWPNRLEH
LDLSHNDISTVMPSAFRGLSRLRFLDLSDNKIQFITEMTFEGLGNVTHLNLAFNGIAVIGDAFLRLYGLKDLNMRGNS
LAVLNQTALGPVVNRLKRLEVAGNPFLCDCSLRWFVEWAVGKYDRVPNLYNPYPDVGRGYTCSRPAQLRGRRLIDGLI
QKQQFNDRKDPAERKFFEAVCSHGFWPNRLLACVLASSGIFVAMMTIFLVNYHIARVQYYMWQLAKWRRPKIGEVENQ
ERLRYTHDAFIAYNNEDVMWVVHEAIENLEPDYSLVIHERDFAVGAPIVENIADAVENSRRTVCLITRNFLKSQWCEY
EFQMAQYHMFEKGGSRRLILVFLERIPDRLLKRFRHLNAVMKRDTYLVWPGDVRKRPLFWERLRHALGDPLPRDPEPQ
QQVQDPEWNIPEQQEQAPERNIPEQAPVRNIVEVQVHGPMRNIPEQQDEIVLLEPDDQWFGDEDDVPLLPL

>Bl08928a

MFVLSTGSQRGIKKEIDMGPKPALLAVCLAVVSAFITQHGRVTGQITPALGPPVCPVWNSTTVWCEGDDWLPNRARLT
QVPPGIPESVITVHLNNNNITKLYNGSFSELTNLKSLDLSYNDLHTIEVGAFAGLENLERLDLSGVEVGWGSKLKYSL
QNDLFRDLQNLNYLAVTTSTETVSAGVFTGLSKLRHLELTVKNVSLLPDHMFDSLTSLESLKIKEEIFTINSSVHSGS
GFLQXLRFLDLSYNQIQTIAEKMLEGLGNLTHLNLASNRIAVIGDAFLRLYGLKDLNMRGNSLAVLNQTILSPVVNRL

ERLDVAGNPFLCDCNLMWFVEWANDKYDRVSNWQNPYYGVGLGYTCSRPAQLRGRLLIEGLTQKKRSKDRKDPTESRF
FGTDCNQGFRPNRLLACVLASSGIFVAMMTIFLVDYHIARVQYYLWQLAKWRRPKIGEVENNEPLRYTHDAFLAYNNR
DVMWVVHEAIENLEPDYSLVIHERDFAVGAPIVENIADAVENSRRTVCLITRNFLKSKWCEYEFQMAQYHMFEKGGGR
RLILVFLERIPDRLLKRFRHLNAVMKRDTYLVWPGDVRKRPLFWERLRHALGDPLPRDPEPQQQVQDQERNIPEQAPV
RNIVEVQIHGPMRNIPEQQDDILLPEPDDQWFGDGEDGPLLPL

>Bl08928b

MESKPALLAVCLAVVSAFITQHGRVRGEYRPPVCQTWNSTTVACAGDNSWLTTSALLSQVPPGIPESVTTLILDHNNI
TELYNGSFSGLRNLRSLDLSYNYLQTIEVGTFAELENLERLDLSGVAGGWGLNLNYSLQNDLFRDLQTLNYLAVTTST
ETISAGVFTGLSKLRHLDLAVKNVSLLPDHIFDSLTSVESLKIEDSSTSSYSLSEEKVGEDEDNITRRVHNGRGFLER
PLLWAPLYNLNKLSLGGQLRASDLYFGTVFRNMTKLETIEITIDRTEIFDPDPRPLSVEMFRPLLLTLKHLVTEEPIA
PGLLKSLTHLQTLNVNYLTNVDVLPELNHTEIQELSLDMWRVYKITSDTLSGIKGLKYLKDLSGGFLAYIRTIEANAF
VGLSHLQRLDLTAVSLKNLHNKTFSGLCSLTHLNLKRNAISTLPVDVFEGLTSLRYLHLKSNNLETFRVQLPETLDYL
DLSYNNLYTSGYTSQPCVNISSFTFSRLKSVNYLDLSHNTIGLIDCSCLPSNITVLNLQSNKITEVNTNVYCIASLRY
LDLSNNNINVWSDEQQCEQPNLEILRLDNNPSGFIAEDFNIGSASISLKYLKTLTLSRCSIPSISPKGFRGLYHLTYL
DLSHNEIDTVKQSVFRELSRLRFLDLSNNRIQNMAERTFEGLGNLTYLNLAFNNIAVIGNAFLRLYGLNDLNMRGNSL
TVLNQTVLSRVVNRLRGLDVAGNPFLCDCNLMWFVEWATDKYDRVSNWQNPYYGVGQGYTCSRPARLNGRRLTDGLTL
TQNKRSNDRKDPTESMFFGKDCSQGFRPNRLLACVLASSGIFVAMMTIFLVNYHIARVQYYLWQLAKWRRPKIGEVEN
HEPLRYTHDAFIAYNNRDVMWVVHEAIENLEPDYSLVIHERDFAVGAPIVENIADAVENSRRTVCLITRNFLKSQWCE
YEFQMAQYHMFEKGGGRRLILVFLERIPDRLLKRFRHLNAVMKRDTYLVWPGDVKKRPLFWERLRHALGDPLPRDPEP
QQQVQDPERNIPEQQAQAPEQNIPVQQEQAPVRNIVEVQVHGPMRNIPEQQDEILLPDDPWFGDGDDVPLLPL

>Bl08928c

MKSKPALLAVCIAVVSAFITQHGRVRGQLPPPVCQTWNSKTVECAGKTYDSPTGLQLMKVPLTQVPSGIPESVITVDL
RNNNITRLYNGSFSGLRNLKSLNLYHNNLQNIEVGTFAGLENLERLDLYGLIERRGLKITYSLQNGLFLDLENLNYLR
VKTFTEKISEGVFTGLSKLRHLDLTVKYVNLLPDHIFDSLTSLESLKIKENVFAIAGRVHSGSRFLQRPLLWAPLYNL
NKLSLYCLVRAIDLNFGPVFRNMTRLETIEITIVRTADFHPALRPLSVEMFQPLLLTLKHLLTNAPIAPGLLKSLTHL
QTLSVISYRYPTTVHVLRELIHTEIQELSIDISRDRTYKITSHTLAGIKSLKYLKHLFFKKLDVTNIRTIEANAFAGL
SHLQRLNLMRGSLKTLQDKTFSGLSSLTHLNLAGNRISTLPAGVFEGLASLRHLDLRGNNLQTFRVQLPETLDYLGLA
GNNLDTRRHIGLTQPSCENSPFNLRGLKSVNHLDLRYNANKAINGQCLPLDITVLNLQYNYISQFNTDVYCIASLRHL
DLSNNHITMVLGFKQQCLPTLEVLRLGNNPASLIHLYNIDKSPSLKNLKAMNLSHSSIQSISAEQFTGLYHLAYLDLS
HNIINTVKQSVFRELSRLRSLDLSNNQIQNIAENMFEGXIVEKTFEGLGNLTHLNLAFNRIAAVGNAFHRLFGLRNLN
LRSNKLAVLNQTTFGPVFSRLETIDIADNPFLCDCRLIWFVKWANDKYDMVANWYNPYPVVGRGYTCSIPVHLRGQRL
IDGLTQNKRSNDRNDPTESTFFDMDCSHGFRPNRLLASVFASSGIFVAMMTIFLVNYHIARVQYYLWQLAKWRRPKIG
EVDNQEPLRYTHDAFMAYNNRDVMWVVHEAIENLEPDYSLVIHERDFAVGAPIVENIADAVENSRRTVCLITRNFLKS
QWCEYEFQMAQATCLRRGEGGVSSWCFWRGFLTEC

>Bl75614
METKITLLAVCLTTILAFITQVWAGQLPPDCQVWNSTAVVCTGQGGPWEYPTRATLTQVPPGIPESVISLDLSRNNIT
ELYKDSFAGLTTLRYLALRENNIAKIHNGSFRGLRNLRHLDLSDTRIQAIEVGAFAGLENLERLDMCCPTSQSTFALQ
TGLFIDLRNLNYLTVETATERVDVDVFKDLSRLRYLYLGLINIRSLPHHIFDSLTLLKNLTIAELDIQSGSEFLQRPL
LWSPLHKLKTFQLFVQRADDLNFGSVFGNLSNLEEIQLGSVGPVNCNVQMFLPLLSTLKRLDLRLSAHVYTPADHLTI
EPGLLTSLTHLQALDIPWTTFSSIAEVLPELRHTHVQELSFAMGGINTITADTLAIMKGLDELKTLSIILPDTTVIQA
NSFTTFPNLQRLNLDVGILNTVENKAFSGLPSLSHLDLSNHLISTLPQDVFEGLSSLICLDLSYNNLETSQARLPETL
DYLDLSHNNLNSYFQMESGYIALSFNFTDLKSVNHLNLSHNRLQGVEMLPANVTMLDLQHNQIGYYLPYSVFSIAGLK
FLDLSHNDIRTTSYTTVQSSTLETLRLDSNGLVGIDGTFMKGLTNLTILTLSHNHIRVIEAGNFQWLVQLLQLDLSHN
EISTLKSFAFRGLSRLRSLDLSNNQIQNMTAKTFEGLGDLKILNLADNRIAVIWNAFDRLYGLRDLILSNNRLSVINQ
TSLGXNRLSVINQTSLGPVVNRLERLDVANNPFLCDCDLRWFVEWAQDKYDTVSNWNNPYPLSYRSYKCFRPAKLYGQ
RLTDGITQQQNYNEGKAQSENMFFKMDCSHEFRPNRLLACVLASSGIFVAMMTVFLVDYNIGHVHHYLWLWAKWRKPR
IGEEEINEQHAYTHDAFIAYNNEDIRWVIKEAIENLEPDYSLVIHERDFAVGAPIVENIADAVENSRRTVCLITRNFL
KSKWCEYEFQLAQYHMFEQGGGVRLILVFLEEIPNRLLKRFRHLNAVMKRDTYLMWSEDVKHRPLFWKRLRHALGEPL
PRDPEPEQIPMTEPHIPEQIDQTAVGNIPEWQDHSPGRKVPEQKGQAPIKNCPELQTQAPTRNTPEYQLLHPLMHIPE
QQKTTTTPECAIHGPEDRIVESESEDDWCGPRDADIILLPL

>Bl30396

MASKPVLLAVCLAAVSAFIAQHGRVRGLPPPVCQTWNSTTVVCTRGIFDFPLRAPLTQVPPGIPESVITVDLNNNNIT
ELNNGSFCGLRNLKSLDLSYNDLQTIEVGTFAGLRNLRSLDLSYNNLQKIEVGTFAGLENLECLDMCVPRRMQEPMYA
LRNGLFQDLRNLNHLRVMTYTNTVSEGVLTGLSKLKHLELNVKNVSNFPDHIFDSLTALESLKIVDLLMDLAIHGAIE
EDRNATTRRVHGQRGFLQRRLLWAPLYNLKELNLFFLPRSIDFYFGPVFRNLSNLKTVEIISLHNYPVSVQMFRPLLL
TIKHLHIHTPIAPGLLKLLTHLQTLDFEHVFFPTIVNVLTELRHIHIKELSFSMLGYGGPITTSFTQARLGTVIANHT
LAGLKGLKYLKGLILDLQNSIIQVVQANAFAGFSHVQRLDLTNGLYLKTLQDKTFSELSSLTHLNLTQNTIATLPADV
FEGLTSLTHLDLSHNNLVTSLAQLPRTLDYLDLSYNNLYSKPYQPPCHNPLSFNFGDLKTVNHLDLSHNNIIVVDNTC
LPRNITVLDLQHNKIESADATYCMESLRYLDLSNNDIMTFSPNVYYSRCAGFTLLETLRLSNNAIEYIHLPGWFPNLQ
TLTISLNHIKRIETDQFPGLDRLEHLDLSHNDINTIMPSAFRGLSRLRFLDLSDNKIQFITEMTFEGLGNLKHLNLAA
NKIAVIGNASLHLYGLRNLNLRSNRFAVLNQTTFGPVVNRLDTIDIADNPFRCDCNLMWFVEWAQDKYDRITNWRNPY
PGVGRGYTCSRPAQLRGRRLIDGLTQKQHYNGRKDPTESTFFDIDCXSMFFDIDCSQGFRPNRLLACVLASSGIFVAM

MTIFLVNYHIARVQYYLWQLAKRRRLKIGEEENQKPHKYTHDAFLAYNNKDVMWVIHEAIENLEPDYSLVIHERDFAV
GAPIVENIADAVENSRRTVCLITRNFLKSQWCEYEFQMAQYHMFEKGGRRRLILVFLEMIPDKMLKRFRHLNAVMNRD
TYLVWPGDVRERPLFWERLRHALGDPLPRDPEPQQQVQDQERNIPEQQEQAPVRNIPEEQEQAPLRNIVAVQVHGPMR
NIPEQQEQAPVRNIEEVQVHAPMRNIPEQQDEILEPDDPWFGDGDDVPLLSL
